# Chiral Chalcogenyl‐Substituted Naphthyl‐ and Acenaphthyl‐Silanes and Their Cations

**DOI:** 10.1002/chem.202002977

**Published:** 2020-10-27

**Authors:** Sandra Künzler, Saskia Rathjen, Katherina Rüger, Marie S. Würdemann, Marcel Wernke, Patrik Tholen, Corinna Girschik, Marc Schmidtmann, Yannick Landais, Thomas Müller

**Affiliations:** ^1^ Institut für Chemie Carl von Ossietzky Universität Oldenburg Carl von Ossietzky-Str. 9–11 26129 Oldenburg Germany, European Union; ^2^ Institute of Molecular Sciences (ISM) University of Bordeaux, CNRS, UMR-5255 351 Cours de la libération 33400 Talence France, European Union

**Keywords:** chalcogens, chirality, Lewis acid, silicon, silyl cations

## Abstract

Cyclic silylated chalconium borates **13**[B(C_6_F_5_)_4_] and **14**[B(C_6_F_5_)_4_] with *peri*‐acenaphthyl and *peri*‐naphthyl skeletons were synthesized from unsymmetrically substituted silanes **3**, **4**, **6**, **7**, **9** and **10** using the standard Corey protocol (Chalcogen Ch=O, S, Se, Te). The configuration at the chalcogen atom is trigonal pyramidal for Ch=S, Se, Te, leading to the formation of *cis*‐ and *trans*‐isomers in the case of phenylmethylsilyl cations. With the bulkier *tert*‐butyl group at silicon, the configuration at the chalcogen atoms is predetermined to give almost exclusively the *trans*‐configurated cyclic silylchalconium ions. The barriers for the inversion of the configuration at the sulfur atoms of sulfonium ions **13 c** and **14 a** are substantial (72–74 kJ mol^−1^) as shown by variable temperature NMR spectroscopy. The neighboring group effect of the thiophenyl substituent is sufficiently strong to preserve chiral information at the silicon atom at low temperatures.

## Graphical abstract

**The Corey reaction** of acenaphthyl‐ and naphthyl‐based chiral silanes allows the synthesis of silylchalconium ions with control of the stereochemistry at the chalcogen atom (shown here for the sulfonium ion). The chalcogen neighboring group effect on the silyl cation is sufficiently strong to conserve the stereochemistry in a consecutive nucleophilic attack.

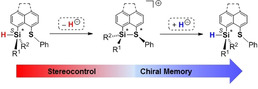

## Introduction

**Figure 1:**
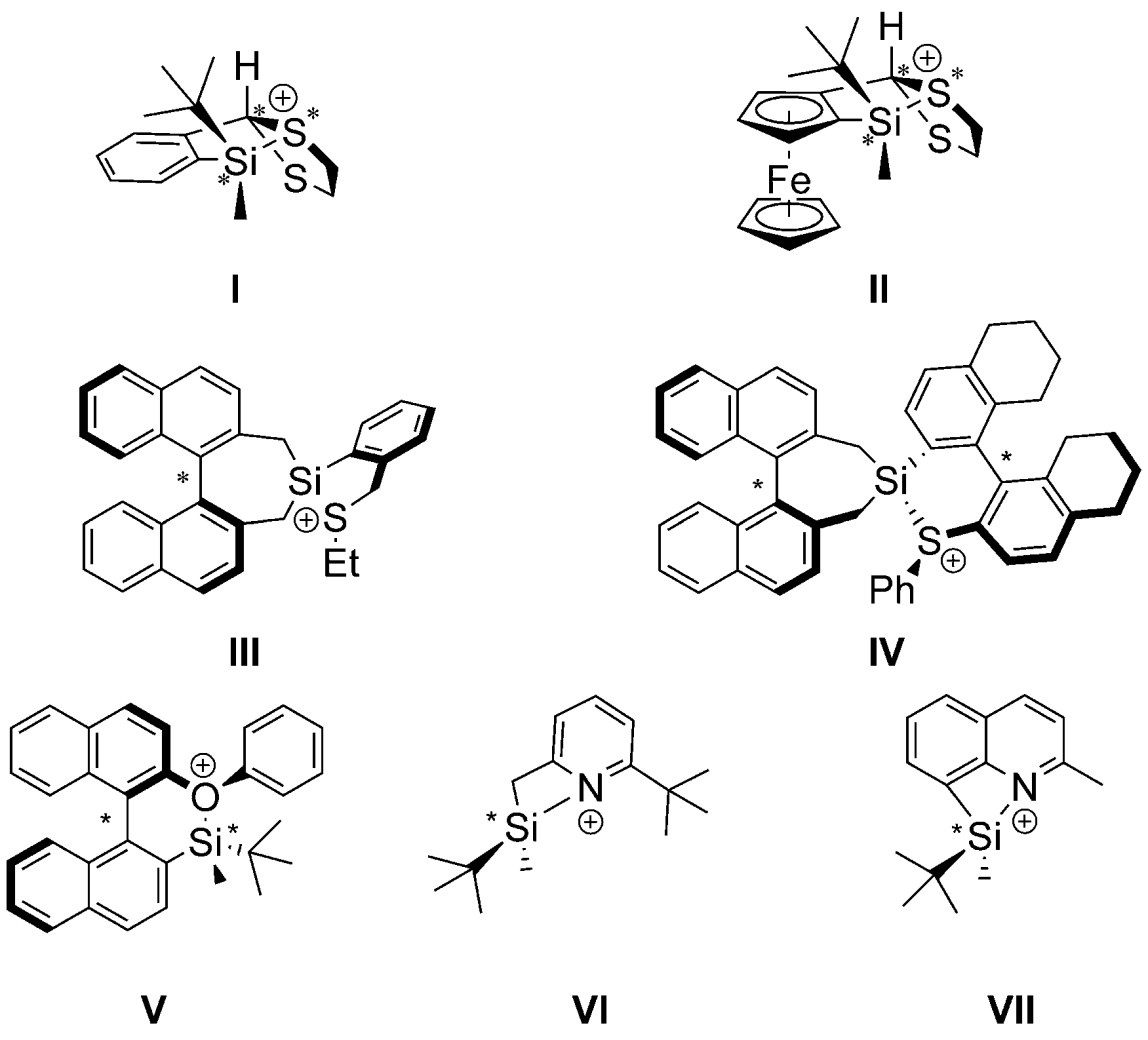
Chiral silyl cations synthesized by the groups of Oestreich and Landais.[^27^, ^28^, ^32^, ^36^, ^37^]

The last 25 years have witnessed the successful development of silyl cations from laboratory curiosities to valuable catalysts in organic transformations.[^1^, ^2^, ^3^, ^4^, ^5^, ^6^, ^7^, ^8^] By virtue of their high Lewis acidity, silyl cations have proven to be excellent catalysts in Diels–Alder reactions,[^9^, ^10^, ^11^, ^12^] hydrodefluorination[^13^, ^14^, ^15^, ^16^, ^17^, ^18^, ^19^, ^20^, ^21^] and CO‐activation reactions.[^22^, ^23^] In these applications, it is mandatory that the high Lewis acidity of the tricoordinate silylium ion is pacified by an intramolecular or intermolecular donor to control the reactivity of the silyl Lewis acid. For regeneration of the cationic catalysts, it is important that the donor/cation interaction is reversible. These partly contradicting requirements have to be addressed and to be compromised on the way to an efficient silyl cation‐based catalyst. Already in 1998, the quest for chiral silyl cation catalysts appeared on the agenda. Jørgensen and Helmchen designed a chiral cationic silyl Lewis acid by introducing the axially chiral *bis*‐naphthyl backbone.^[24]^ Later, Ghosez and co‐workers used non‐ionic chiral silyltriflimides derived from substituents from the chiral pool.[^25^, ^26^] In a series of investigations, Oestreich and co‐workers used different families of intramolecularly stabilized silyl cations to introduce chirality on these silyl Lewis acids.[^27^, ^28^, ^29^, ^30^, ^31^, ^32^, ^33^] Particularly interesting in the context of our study are silylsulfonium ions **I**–**IV** (Figure 1).[^28^, ^30^, ^31^, ^32^, ^33^] In these examples the neighboring group effect of the sulfur donor creates new centers of chirality that are controlled by the present stereo element (centered chirality at the silicon atom or axial chirality). Consequent structure modifications finally led to the silylsulfonium species **IV**, which catalyzes challenging Diels–Alder reactions at low temperatures in good yields and decent enantioselectivities (up to 81 % *ee*).^[32]^ This approach based on chiral silyl Lewis acids competes with the asymmetric counter‐anion‐directed silylium catalysis popularized by the List group.[^34^, ^35^] The question whether the stereochemical information at the silicon atom is retained by the neighboring group effect of an electron‐donating group during the typical ionization/nucleophilic addition sequence of silylium ion catalysis was recently addressed by Landais, Robert and co‐workers.[^36^, ^37^] They showed in their chiral memory experiments for the intramolecularly stabilized silyl cations **V**–**VII** that during the complete silyl cation formation/hydride addition reaction sequence the stereochemistry at the silicon atom is controlled by the anchimeric assistance of the oxygen or nitrogen donors.

**Figure 2:**
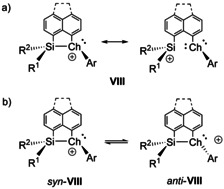
a) *peri*‐Naphthyl and *peri*‐acenaphthyl‐based silylchalconium ions **VIII**. (Ch=O, S, Se, Te; R^1^=Me, Ph, *t*Bu, R^2^=Me, Ar=Ph, Mes) b) Inversion of the configuration at the chalcogen atom in cations **VIII** (for Ch=S, Se, Te).

We recently investigated silyl chalconium ions **VIII** (R^1^=R^2^=Me) based on the rigid *peri*‐naphthyl and *peri*‐acenaphthyl skeleton (Figure 2).^[38]^ These silyl cations are stabilized by the electron donating neighboring effect of the chalcogen atom generating a trigonal pyramidal chiral center at the chalcogen atom. Based on the result by the Oestreich group,^[27]^ we expected control of the stereochemistry at the chalcogen atom by installing a chiral silicon center (R^1^=*t*Bu, Ph; R^2^=Me). We report here the synthesis of cations **VIII** with non‐symmetric substitution pattern at the silicon atom (R^1^≠R^2^). Depending on the substituents at silicon, we found indeed a clear control of the stereochemistry at the chalcogen donor and we determined its stability by NMR methods supported by the results of density functional calculations. Finally, the conservation of the stereochemistry at the chiral silicon atom through anchimeric assistance by the chalcogenyl group was tested applying the silicon cation formation/hydride addition sequence suggested by Landais and Robert.[^36^, ^37^]

## Results and Discussion

**Scheme 1:**
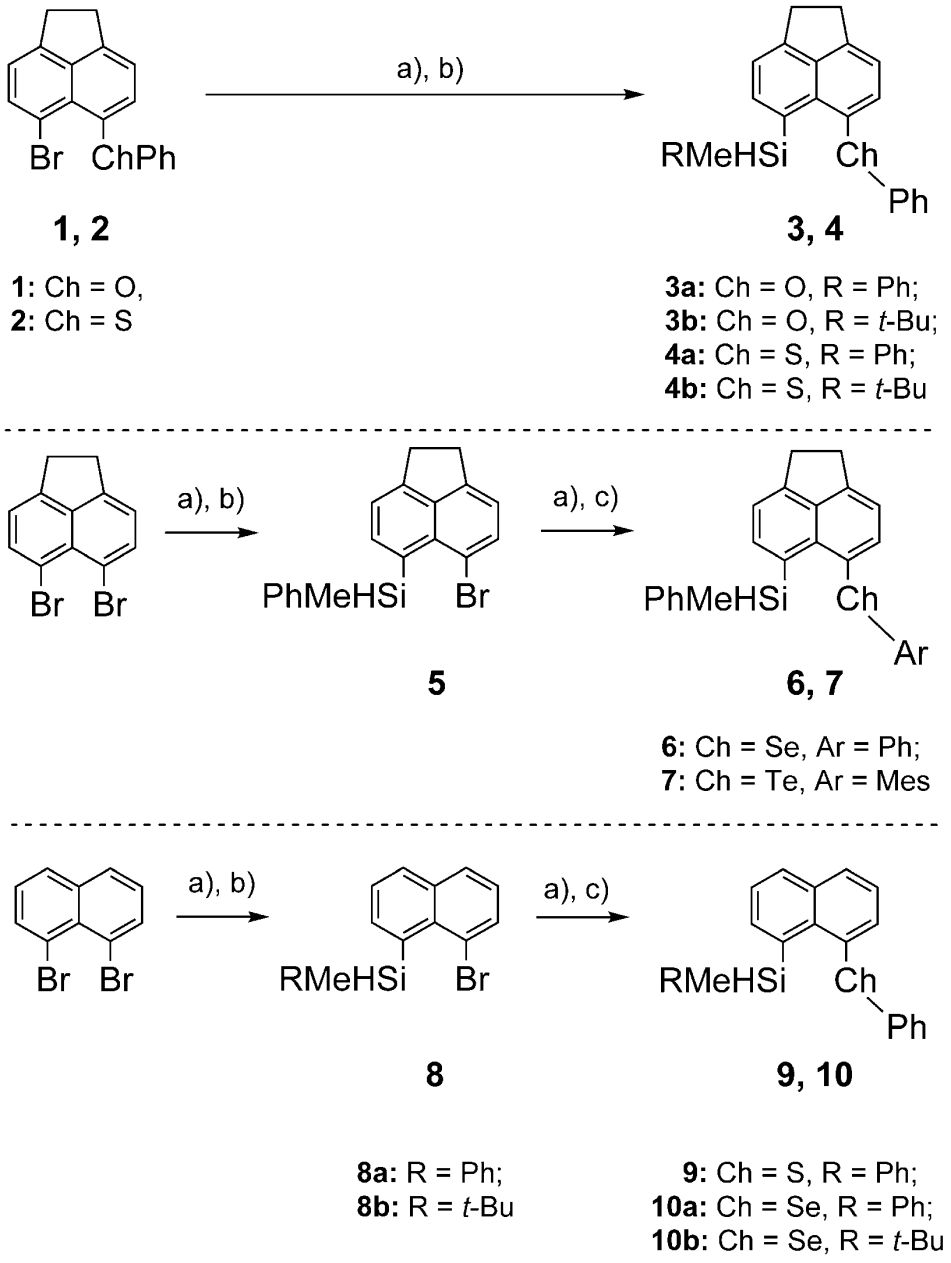
Syntheses of silanes **3**, **4**, **6**, **7**, **9**, **10**. a) 1 equiv *n*BuLi, THF, −80 °C; b) 1 equiv RMeSiHCl, THF, −80 °C, R=Me, Ph, *t*Bu; c) 1 equiv PhSSPh or PhSeSePh or MesTeTeMes, THF, −80 °C.

The synthesis of phenoxy‐ and phenylsulfanyl‐substituted acenaphthyl silanes **3** and **4** was realized by a metalation/salt metathesis sequence starting from 5‐bromo‐6‐phenoxyacenaphthene **1** or 5‐bromo‐6‐(phenylsulfanyl)acenaphthene **2** (Scheme 1, top). Selanyl‐ and tellanyl‐substituted derivatives **6** and **7** as well as naphthyl‐substituted derivatives **9** and **10** were synthesized by two subsequent metalation/salt metathesis steps starting from 5,6‐dibromoacenaphthene or 1,8‐dibromonaphthalene (Scheme 1, middle, bottom). In the first step, the silyl group is introduced to obtain *peri*‐bromo silyl acenaphthene **5** or naphthalenes **8** and in the second step the chalcogenyl group is installed by lithiation and subsequent treatment with the corresponding dichalcogenide.[^38^, ^39^]

**Scheme 2:**
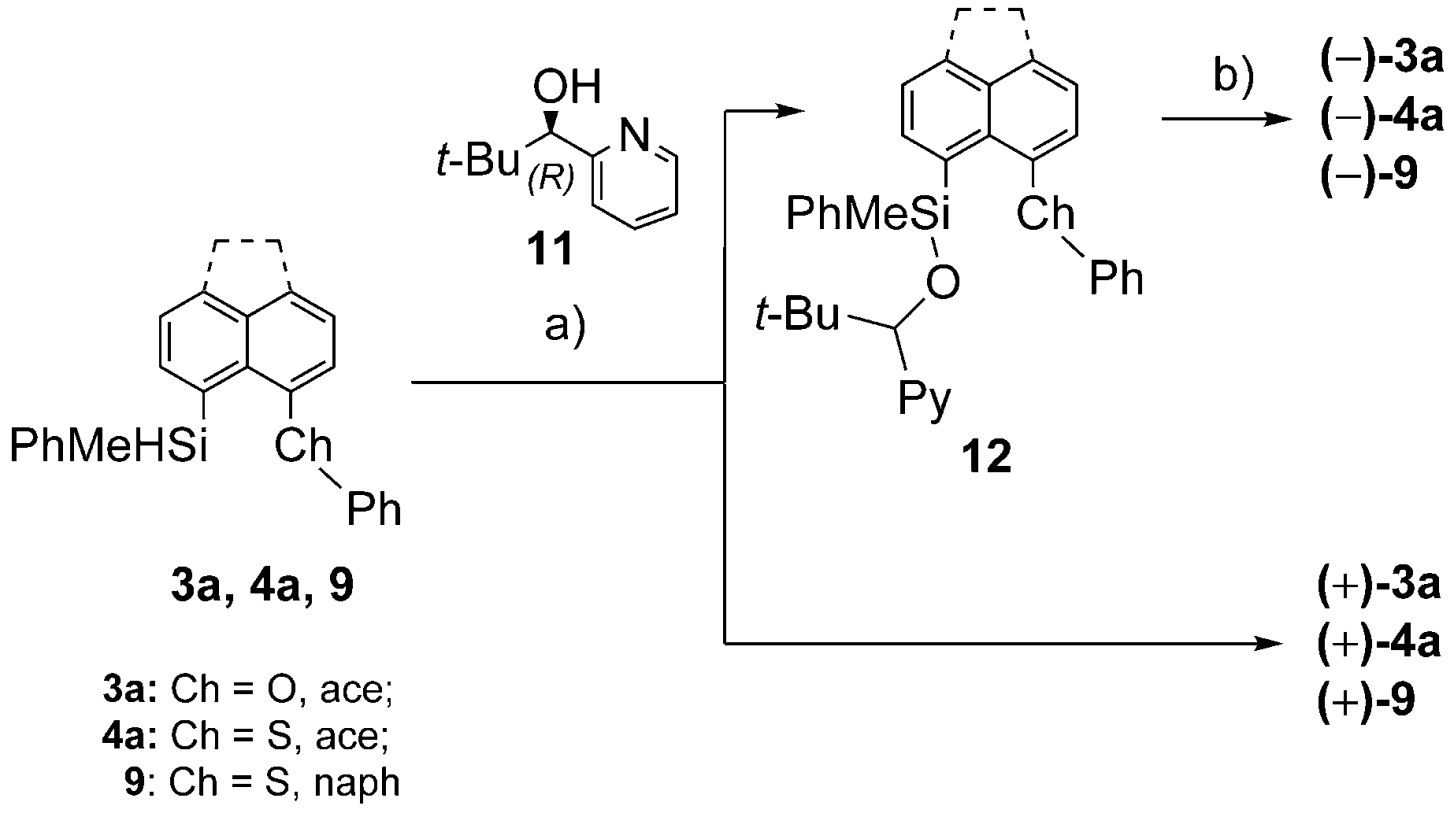
Chiral resolution of silanes **3 a**, **4 a** and **9**. a) 0.2 equiv CuCl, 0.4 equiv Ph_3_P, 0.2 equiv NaO*t*Bu, 0.5 equiv (*R*)‐**11**, toluene, r.t.; b) 2 equiv DIBAL‐H, Et_2_O, r.t.).

Kinetic resolution using a protocol developed by Oestreich et al. was successful for the separation of the racemic phenyl substituted silanes **3 a**, **4 a** and **9** into their enantiomers.^[40]^ It relies on the dehydrogenative Si–O coupling of silanes and uses 0.5 equivalent *tert*‐butylpyridyl alcohol (*R*)‐**11** as an auxiliary and a copper(I) catalyst system (Scheme 2). The enantiomerically selective dehydrogenative Si–O coupling led to the formation of siloxanes **12**, while the (+)‐enantiomer of silanes **3 a**, **4 a** and **9** did not react. After separation from the (+)‐silanes, the siloxanes **12** were reduced to the (−)‐silanes **3 a**, **4 a** and **9** using diisobutylaluminium hydride. Enantiomerically enriched silanes **(+)‐3 a**, **4 a**, **9** and **(−)‐3 a**, **4 a**, **9** were obtained in moderate to good purities (*ee*=54–84 %, further details see Supporting Information). The *tert*‐butyl‐substituted silane **4 b** was found to be completely inert under the tested reaction conditions, probably due to the steric demand of the *tert*‐butyl substituent.

The obtained silanes were fully characterized by multinuclear NMR and IR spectroscopy as well as using high‐resolution mass spectrometry and elemental analysis. Selected NMR and IR data are summarized in Table 1. All investigated silanes **3**, **4**, **6**, **7**, **9** and **10** show the expected ^1^H NMR chemical shift for the hydrogen atom which is attached to the silicon atom in the range from δ^1^H=4.95 to 6.29.[^38^, ^39^] The corresponding ^29^Si NMR chemical shifts are between δ^29^Si=1.3 and −21.3. The Si–H coupling constants are in the expected range of ^1^ *J*(HSi)=193 to 203 Hz.[^38^, ^39^] A trend of the ^29^Si NMR resonance moving towards higher field with increasing atomic weight of the remote chalcogen atom is noticeable (Table 1). The ^77^Se and ^125^Te NMR chemical shifts of silanes **6**, **7** and **10 a**,**b** are in the expected range for diarylchalcogenides.[^38^, ^39^, ^41^, ^42^, ^43^] Remarkably, the selanyl‐ and tellanyl‐substituted derivatives show in addition to the Si‐H ^1^ *J* coupling, a characteristic chalcogen–hydrogen through‐space coupling (^TS^ *J*(ChH)) between the chalcogen atom and the hydrogen atom of the silyl group (^TS^ *J*(SeH)=14–28 Hz and ^TS^ *J*(TeH)=66 Hz).[^38^, ^44^, ^45^] Previously, Beckmann and co‐workers disclosed a related Si–H⋅⋅⋅P interaction in acenaphthene based compounds.^[46]^ The IR absorption bands for the Si–H stretching vibration of silanes **3**, **4**, **6**, **7**, **9** and **10** are in the expected range of 2083–2175 cm^−1^. Hereby it is noteworthy, that silanes **3 a,b** and **10 a** show two IR bands due to Fermi resonance.^[47]^

**Table 1:** Selected NMR^[a]^ and IR^[b]^ spectroscopic data of silanes.

Compound	δ^29^Si	δ^1^H *J*(HX)[Hz]	δ^77^Se/δ^125^Te *J*(ChX) [Hz]	*ṽ*(Si‐H)^exp^ [cm^−1^]
**3 a** OPh, Ph	−14.7	5.63 ^1^ *J*(HSi)=196	–	2096, 2136
**3 b** OPh, *t‐*Bu	1.3	4.95 ^1^ *J*(HSi)=193	–	2090, 2151
**4 a** SPh, Ph	−17.2	5.89 ^1^ *J*(HSi)=203	–	2091
**4 b** SPh, *t‐*Bu	−3.2	5.80 ^1^ *J*(HSi)=200	–	2175
**6** SePh, Ph	−18.1	5.55 ^1^ *J*(HSi)=202	δ^77^Se=374.1	2094
**7** TeMes, Ph	−21.3	6.29 ^1^ *J*(HSi)=193, ^TS^ *J*(HTe)=66	δ^125^Te=432.4	2108
**9** SPh, Ph	−16.9	5.83 ^1^ *J*(HSi)=203	–	2144
**10 a** SePh, Ph	−18.0	5.98 ^1^ *J*(HSi)=202	δ^77^Se=393.0 ^TS^ *J*(SeH)=14	2083, 2141
**10 b** SePh, *t‐*Bu	−4.9	5.45 ^1^ *J*(HSi)=196 ^TS^ *J*(HSe)=28	δ^77^Se=416.3 ^TS^ *J*(SeH)=27	2131

[a] In C_6_D_6_. [b] Neat.

**Figure 3:**
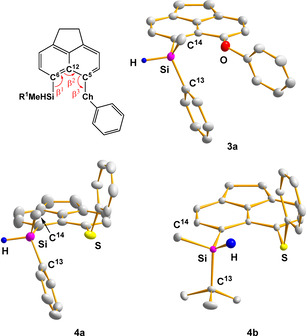
Molecular structures of phenylmethylsilanes **3 a**, **4 a** and *tert*‐butylmethyl silane **4 b** (thermal ellipsoids at 50 % probability, H atoms omitted for clarity except the Si‐H). Pertinent bond lengths [pm] and bond angles [°]. Phenylmethyl silane **3 a** (triclinic space group P‐1): ): Si/O 292.3(2), Si−C6 187.6(1), O−C5 138.8(2), Si−H 141(2), O‐C5‐C12 114.7(9), Si‐C6‐C12 126.8(8), C5‐C12‐C6 127.4(1), C6‐Si‐C13 109.4(6), C6‐Si‐C14 116.9(7), C13‐Si‐C14 (112.6(8). Phenylmethyl silane **4 a** (monoclinic space group Cc): Si/S 330.55(2), Si−C6 189.6(5), S−C5 178.4(4), Si−H 147.(5), S‐C5‐C12 121.8(4), Si‐C6‐C12 132.1(3), C5‐C12‐C6 128.2(4), C6‐Si‐C13 113.5(3), C6‐Si‐C14 115.7(3), C13‐Si‐C14 (111.2(3). *tert‐*Butylmethyl silane **4 b** (triclinic space group P‐1): Si/S 333.49(3), Si−C6 190.15(5), S−C5 177.16(5), Si−H 138, S/H 281, S−C5‐C12 123.20(4), Si‐C6‐C12 130.63(3), C5‐C12‐C6 128.99(4), C6‐Si‐C13 110.063(23), C6‐Si‐C14 108.420(25), C13‐Si‐C14 108.718(24).

Single crystals suitable for X‐ray diffraction (XRD) analysis have been obtained for silanes **3 a**, **4 a**, and **4 b** (Figure 3). Selected structural data are listed in Table 2. In general, the molecular structures show indications of steric strain due to the *peri*‐disubstitution as measured by the sum of the bond angles in the bay region Σβ (Σ*β*=β_1_+β_2_+β_3_) and the out‐of‐plane distances d of the *peri*‐substituents (Figure 3).^[48]^ In the case of the phenylsulfanyl‐substituted derivatives **4 a,b**, Σβ are 382° and 384° and are larger compared to the phenoxy‐substituted derivatives **3 a** (Σ*β*=369°), which equals almost that of unstrained acenaphthene (Σ*β*=368°). The out‐of‐plane distances of the *peri*‐substituents of silanes **3 a**, **4 a**, and **4 b** are rather small (*d*=3.2–21.3 pm). The heavy atom distances in the *peri*‐positions are larger than the distance in unstrained acenaphthene (d(Si/Ch)=292–334 pm vs. d(H/H)=270 pm) but distinct smaller than the corresponding sum of the van der Waals radii of the silicon and the chalcogen atom (ΣvdW (Si/O)=362 pm, ΣvdW (Si/S)=390 pm).^[49]^

**Table 2:** Selected structural parameters from XRD analysis of silanes **3 a** and **4**.

Cpd.	d(Si/Ch) [pm]	Σβ [°]	Σα(SiC_3_) [°]	d(Si) [pm]	d(Ch) [pm]
**3 a**	292.3	368.9	336.5	21.3	−11.7
**4 a**	330.6	382.1	340.5	6.9	−3.2
**4 b**	333.5	383.8	327.2	5.6	13.2

A remarkable feature of the molecular structures of the silanes **3 a**, and **4 a,b** is the configuration of the silyl hydrogen atom relative to the chalcogenyl substituent. The molecular structure of the *tert*‐butyl‐substituted silane **4 b**, reveals a *syn*‐orientation of the hydrogen atom relative to the phenylsulfanyl substituent (C12‐C6‐Si‐H=38.5°). The S/H distance is by 9 pm smaller than the sum of the van der Waals radii (d(S/H)=281 pm, ΣvdW (S/H)=290 pm).^[49]^ The sum of the bond angles around the silicon atom in derivative **4 b** is Σα(SiR_3_)=327.2°, confirming the expected tetrahedral coordination sphere. In contrast, the orientation of the silyl hydrogen atom in phenyl‐substituted silanes **3 a**, **4 a** is *anti* relative to the chalcogenphenyl group (i.e. in **4 a**: C12‐C6‐Si‐H=170.4°). The sums of the bond angles of the silicon atom to the carbon substituents of derivatives **3 a**, **4 a** show a trigonal flattening of the coordination sphere of the silicon atom (i.e. **4 a** Σα(SiR_3_)=340.5°). This distortion of the coordination environment of the silicon center in the *anti*‐structures of **3 a**, **4 a** indicates the onset of Lewis acid/base interaction between the silicon and the sulfur atoms.^[50]^ As a consequence of the donation of electron density of the chalcogen atom to the silicon atom in the *anti*‐structures, the silicon–hydrogen bond is weakened compared to the *syn*‐structures. This is visible by comparing the IR resonances of the Si–H vibration. The value for the experimentally determined IR resonance (ATR, solid state) of *tert*‐butylmethyl derivative **4 b** (*syn*‐structure) is by 84 cm^−1^ larger compared to the wave number of the derivative **4 a**. For comparison, the IR resonances for both conformers (*syn* and *anti*) of the species **3 a**, **4 a**,**b** were calculated using quantum mechanical methods at the DFT M06‐2X/def2‐TZVP level of theory.^[51]^ The calculated values show a similar hypsochromic shift of the wavenumber of the *syn*‐ relative to the *anti*‐conformation (Table 3), discarding the possibility of a pure substituent effect and supporting the assumption of an onset of an intramolecular Lewis‐acid/base interaction with a concomitant weakening of the Si−H bond.

**Table 3:** Experimental and calculated IR Si–H vibrations of silanes **3 a** and **4**.

Compound	*ṽ*(Si‐H)^exp^ [cm^−1^]	*ṽ*(Si‐H)^calc^ *anti‐*conformation [cm^−1^]	*ṽ*(Si‐H)^calc^ *syn‐*conformation [cm^−1^]
**3 a** OPh, Ph	2096, 2136	2138	2101
**4 a** SPh, Ph	2091	2103	2153
**4 b** SPh, *t‐*Bu	2175	2102	2169

**Scheme 3:**
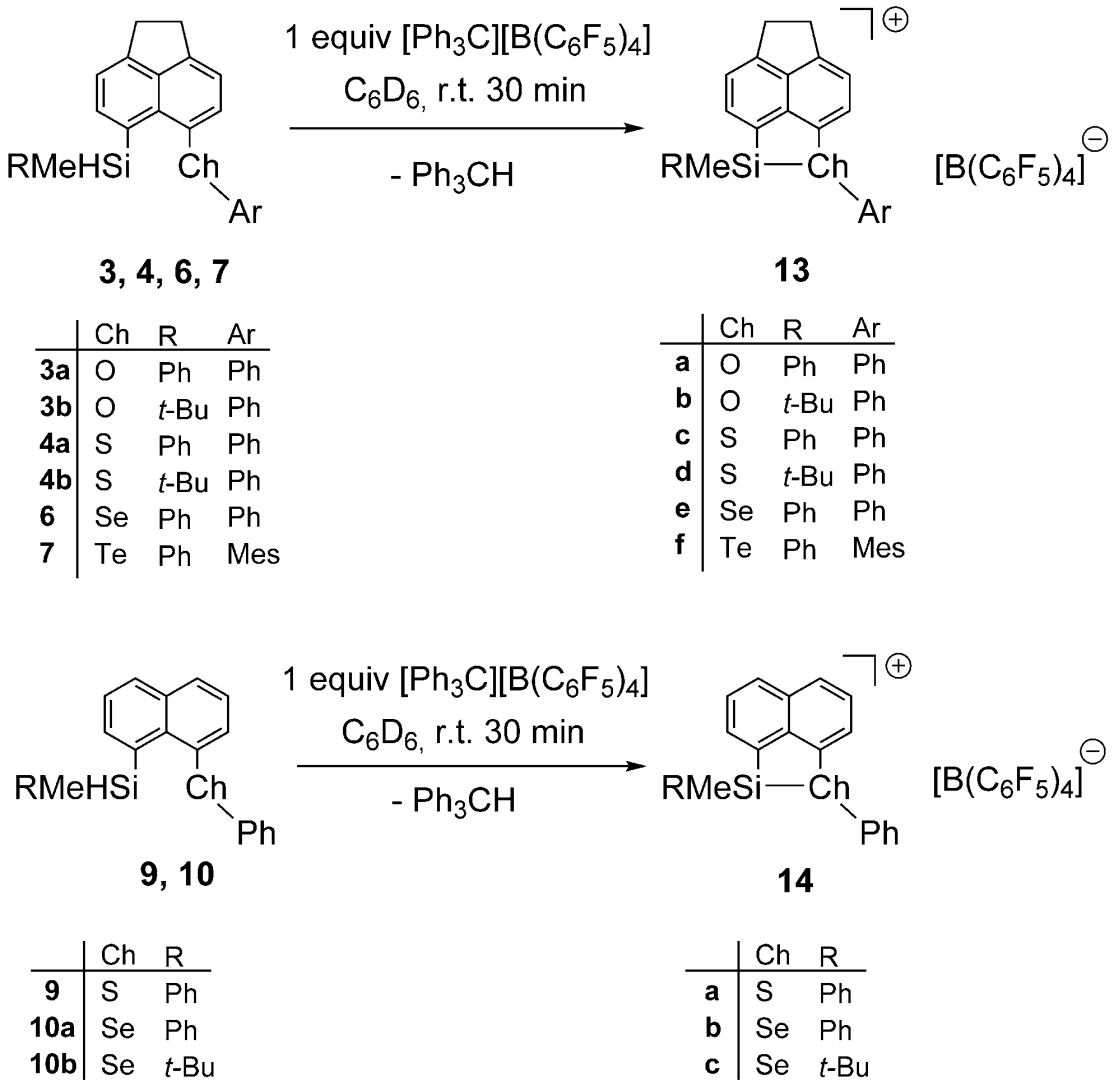
Synthesis of silyl borates **13**[B(C_6_F_5_)_4_] and **14**[B(C_6_F_5_)_4_].

For the synthesis of the silyl cations, we followed the standard Corey protocol.^[52]^ The reaction of silanes with trityl borate [Ph_3_C][B(C_6_F_5_)_4_] in benzene or toluene resulted in the formation of silyl borates **13**[B(C_6_F_5_)_4_] and **14**[B(C_6_F_5_)_4_] (Scheme 3). All new silyl borates were fully characterized by multinuclear NMR spectroscopy. Selected NMR data are summarized in Table 4.

**Table 4:** Selected NMR data of silyl borates **13**[B(C_6_F_5_)] and **14**[B(C_6_F_5_)_4_] (in C_6_D_6_).

Compound	δ^29^Si	δ^1^H of SiCH_3_	δ^77^Se/δ^125^Te	^1^ *J*(ChSi) [Hz]	*cis*/*trans* ratio^[b]^
**13 a** OPh, Ph	60.8	0.63	–	–	–
**13 b** OPh, *t‐*Bu	72.2	0.37	–	–	–
**13 c** SPh, Ph	42.7 (*trans*) 45.2 (*cis*)	0.34 (*trans*) 0.80 (*cis*)	–	–	60:40
**13 d** SPh, *t‐*Bu	70.0 (*trans*)	0.12 (*trans*)	–	–	<1:99
**13 e** SePh, Ph^[a]^	49.7 (*trans*) 42.3 (*cis*)	0.40 (*trans*) 0.83 (*cis*)	260.3 (*cis*), 262.6 (*trans*)	60 (*trans*), 9 (*cis*)	60:40
**13 f** TeMes, Ph	36.4 (*trans*), 40.7 (*cis*)	0.62 (*trans*), 0.88 (*cis*)	234.4 (*trans*), 239.7 (*cis*)	171 (*trans*), 163 (*cis*)	50:50
**14 a** SPh, Ph	42.9 (*trans*) 45.5 (*cis*)	0.33 (*trans*) 0.73 (*cis*)	–	–	60:40
**14 b** SePh, Ph	41.4 (*trans*) 43.7 (*cis*)	0.37 (*trans*) 0.77 (*cis*)	254.7 (*cis*), 256.1 (*trans*)	61 (*cis*)	60:40
**14 c** SePh, *t‐*Bu	54.3 (*cis*) 62.9 (*trans)*	0.06 (*trans*) 0.37 (*cis*)	202.0 (*trans*), 251.7 (*cis*)	68 (*trans*)	<1:99

[a] In C_7_D_8_. [b] Obtained by integration of the ^1^H NMR signals of the methyl groups attached to the silicon center.

**Figure 4:**
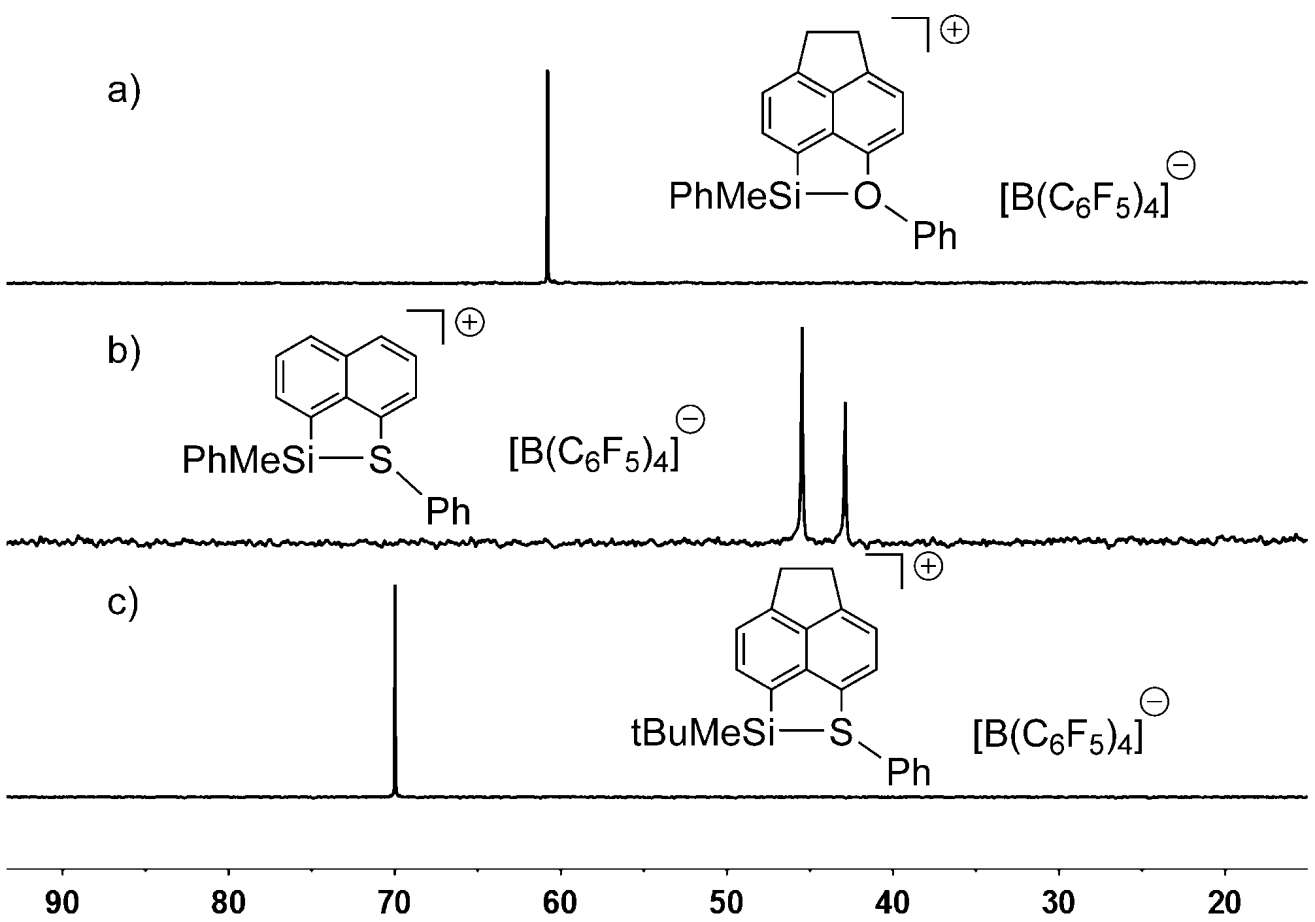
^29^Si{^1^H} NMR spectra (99 MHz, 305 K) of a) silyloxonium borate **13 a**[B(C_6_F_5_)_4_] in C_6_D_6_, b) silylsulfonium borate **14 a**[B(C_6_F_5_)_4_] in C_7_D_8_ and c) silyl sulfonium borate **13 d**[B(C_6_F_5_)_4_] in C_6_D_6_.

The phenylmethyl‐substituted silylsulfonium, silylselenonium and silyltelluronium borates **13 c**, **13 e**, **13 f**, **14 a** and **14 b**[B(C_6_F_5_)_4_] show in all recorded NMR spectra two sets of signals, indicating the formation of two isomers, whereas the silyloxonium ions **13 a**, **13 b** and the *tert*‐butylmethyl‐substituted silylsulfonium **13 d** and silylselenonium ion **14 c** show only one set of signal. This is demonstrated in Figure 4 for a set of ^29^Si NMR spectra. The ^29^Si NMR chemical shifts of silyl borates **13** and **14** are in the expected region for intramolecularly chalcogenyl‐stabilized silyl cations (δ^29^Si=36–72).[^27^, ^28^, ^30^, ^31^, ^32^, ^33^, ^38^, ^39^] As shown previously for related silyl cations, the ^29^Si NMR resonance experiences a high‐field shift with increasing size and polarizability of the remote donor atom.^[38]^ Furthermore, *tert*‐butylmethyl‐substituted derivatives **13 b**,**d** and **14 c** are low‐field shifted compared to the corresponding phenylmethyl‐substituted derivatives **13 a**,**c** and **14 b**. This effect is most pronounced for the phenylsulfanyl‐stabilized acenaphthyl‐substituted derivatives **13 c** and **13 d** (δ^29^Si=42.7 and 45.2 (**13 c**, PhMe) vs. δ^29^Si=70.0 (**13 d**, *t*Bu)).

The ^77^Se NMR chemical shifts of silylselenonium ions **13 e**, **14 b** and **14 c** are significantly high‐field shifted compared to the corresponding precursors (Δδ^77^Se=−114 (**13 e**), −138 (**14 b**), −165 (**14 c**)), indicating the increase of the coordination number of the selenium atom by intramolecular addition of the electropositive silyl group. This effect is also visible for the ^125^Te NMR chemical shift of the tellanyl‐stabilized silyl borate **13 f**[B(C_6_F_5_)_4_] (Δδ^125^Te=−193). Moreover, a *J*‐coupling between the silicon atom and the selenium and tellurium atom, respectively, is observable. The coupling constants are ^1^ *J*(SiSe)=59–68 Hz, ^1^ *J*(SiTe)=163 Hz and 171 Hz and are in accordance with those reported previously for the corresponding dimethyl‐substituted derivatives (cf. ^1^ *J*(SiSe)=55–66 Hz, ^1^ *J*(SiTe)=161–169 Hz).^[38]^ These observations demonstrate the direct linkage of the silicon center to the chalcogen atom.

**Figure 5:**
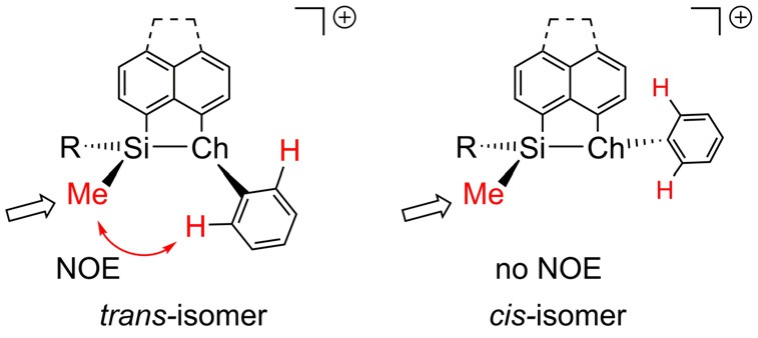
Assignment of the absolute stereochemistry at the Si−Ch linkage in cations **13** and **14** by NOE NMR spectroscopy (Ch=S, Se; R=Ph, *t*Bu).

The formation of two isomers upon ionization of the sulfanyl‐, selanyl‐ and tellanyl‐substituted silanes is a result of the trigonal pyramidal coordination environment of the heavy chalcogen atom in the formed cation. This causes a *syn*/*anti* relation of the substituents at the silicon atom relative to the aryl substituent at the chalcogen atom. This coordination environment is expected based on a fundamental VSEPR treatment and it was confirmed recently by the results of XRD analysis of related dimethyl‐substituted silylselenonium borates and trisilylsulfonium borates and carborates[^38^, ^39^, ^53^] The stereochemistry of cations **13 c**–**f** and **14** was determined by ^1^H NMR Nuclear Overhauser Effect (NOE) difference spectroscopy. Saturation of the ^1^H NMR resonance of the silylmethyl groups leads in the case of the *trans*‐isomers to an enhancement of the signals of the *ortho*‐H‐atoms of the phenyl substituent at the chalcogen atom (Figure 5). The *cis*/*trans*‐ratio was determined by ^1^H NMR spectroscopy to be 60:40 in the case of the PhMeSi substituted cations **13 c**,**e** and **14 a**,**b** while the two stereoisomers of tellanyl‐stabilized cation **13 f** were produced in equal amounts. In contrast, the *trans*‐isomers of *tert*‐butylsilyl substituted cations **13 d** and **14 c** were formed almost stereoselectively. Only in the case of cation **14 c**, small amounts of the *cis*‐stereoisomer were detected (app. 1 %, see Supporting Information).

The different *cis*/*trans*‐ratios are the result of the interaction between the substituents of the silyl group and the aryl substituent at the chalcogen atom. The preference of the *cis*‐configurated phenylmethyl‐substituted derivatives **13 c**,**e**, and **14 a**,**b** indicates slightly attractive interactions (π‐stacking) between the two phenyl groups at the two heteroatoms. In contrast, in *tert*‐butylmethyl‐substituted derivatives **13 d** and **14 c** the *t*Bu group at silicon is oriented *anti* relative to the phenyl substituent at sulfur, demonstrating the strong steric repulsion between the two substituents. Consequently, the configuration of the sulfanyl‐, selanyl‐ and tellanyl‐substituted derivatives is controlled by the substitution pattern at the silicon atom.

**Scheme 4:**
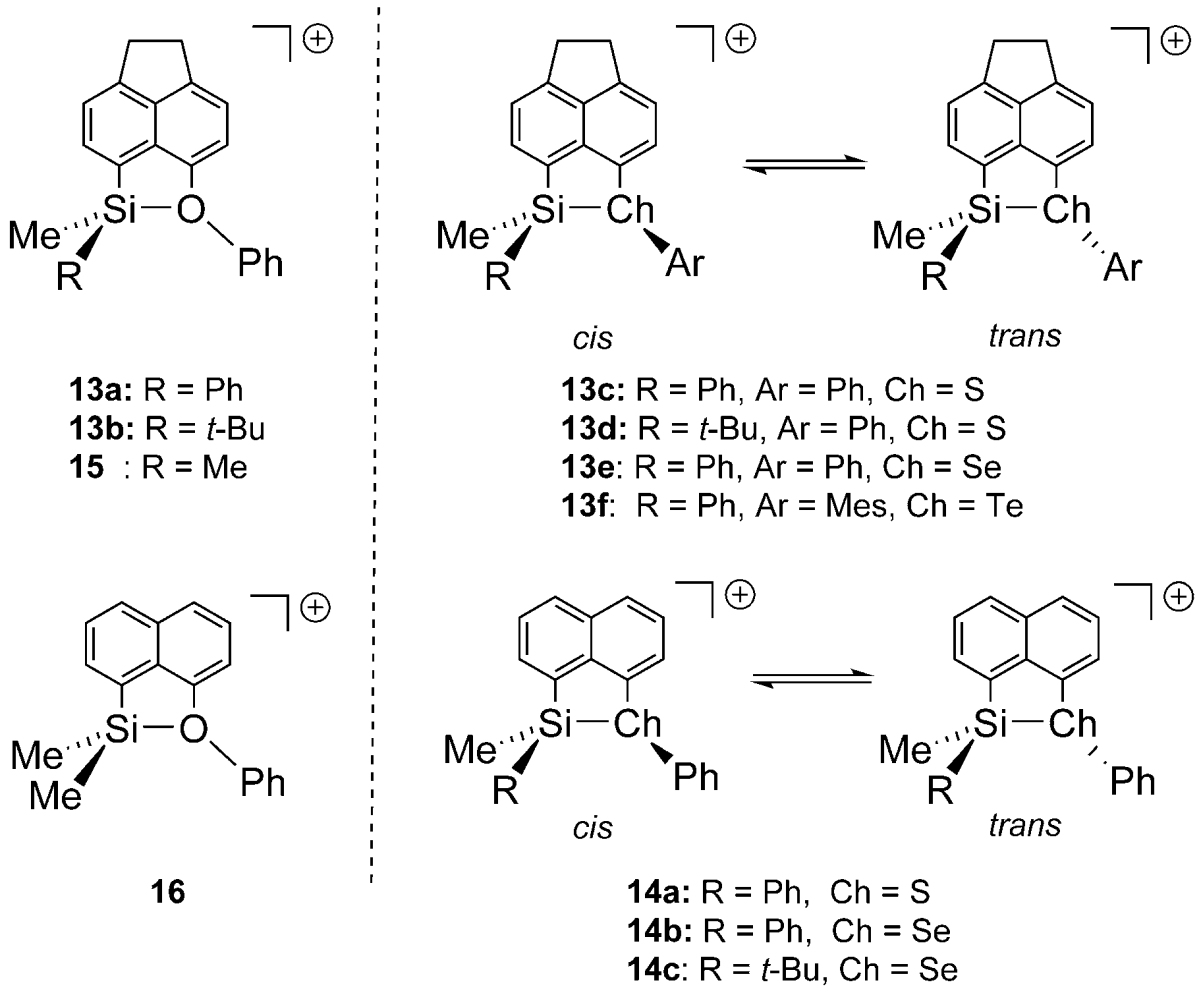
Silyloxonium ions **13 a**,**b**, **15**, **16** with the trigonal planar coordination sphere of the oxygen atom and silylchalconium ions **13 c**–**f**, **14** with the trigonal pyramidal coordination sphere of the heavy chalcogen atom.

The coordination environment of the oxygen atom in symmetrically substituted silyloxonium ions **15** and **16** (Scheme 4) is trigonal planar in solution and in the solid state.[^38^, ^39^] The formation of only one isomer of silyloxonium ions **13 a**,**b** suggests also in these unsymmetrical substituted cations a planar coordination at the tricoordinated oxygen atom.

**Figure 6:**
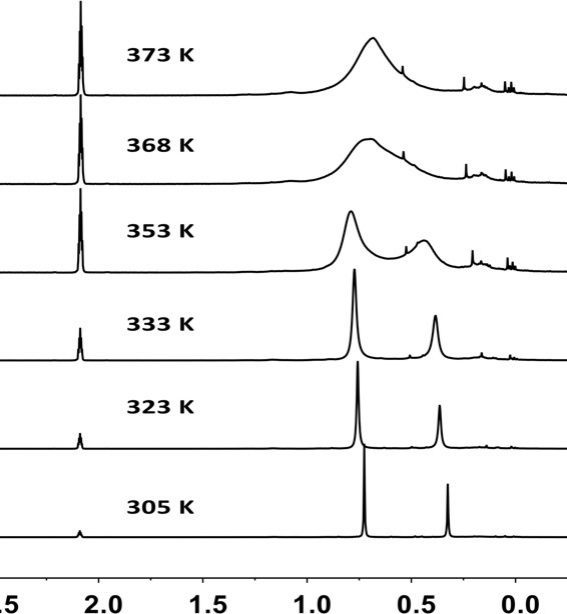
High‐field region of the VT ^1^H NMR spectra (500 MHz, C_7_D_8_) of phenylmethyl‐substituted phenylsulfanyl‐stabilized silyl borate **14 a** [B(C_6_F_5_)_4_].

Variable temperature experiments of the phenylmethyl‐substituted phenylsulfanyl‐stabilized silyl cations **13 c** and **14 a** revealed a dynamic process which leads to a line broadening of the ^1^H NMR signals and finally to coalescence of the signals of the methyl groups at the silicon atom of the *cis*‐ and *trans*‐isomers upon heating (Figure 6). With the coalescence temperatures obtained for the phenylsulfanyl‐stabilized derivatives **13 c** and **14 a**, the free Gibbs energy barriers for this process that interconverts *cis*‐ and *trans*‐isomers were determined to be Δ*G* ^‡^(378 K)=74 kJ mol^−1^ (**13 c**) and Δ*G* ^‡^(368 K)=72 kJ mol^−1^ (**14 a**). For the selanyl‐stabilized derivative **13 e**, line broadening was observed at *T*=373 K, but coalescence of the signals was not achieved. The telluronium ion **13 f** was not tested at higher temperatures, since related telluronium ions showed decomposition at higher temperatures.^[38]^

**Scheme 5:**
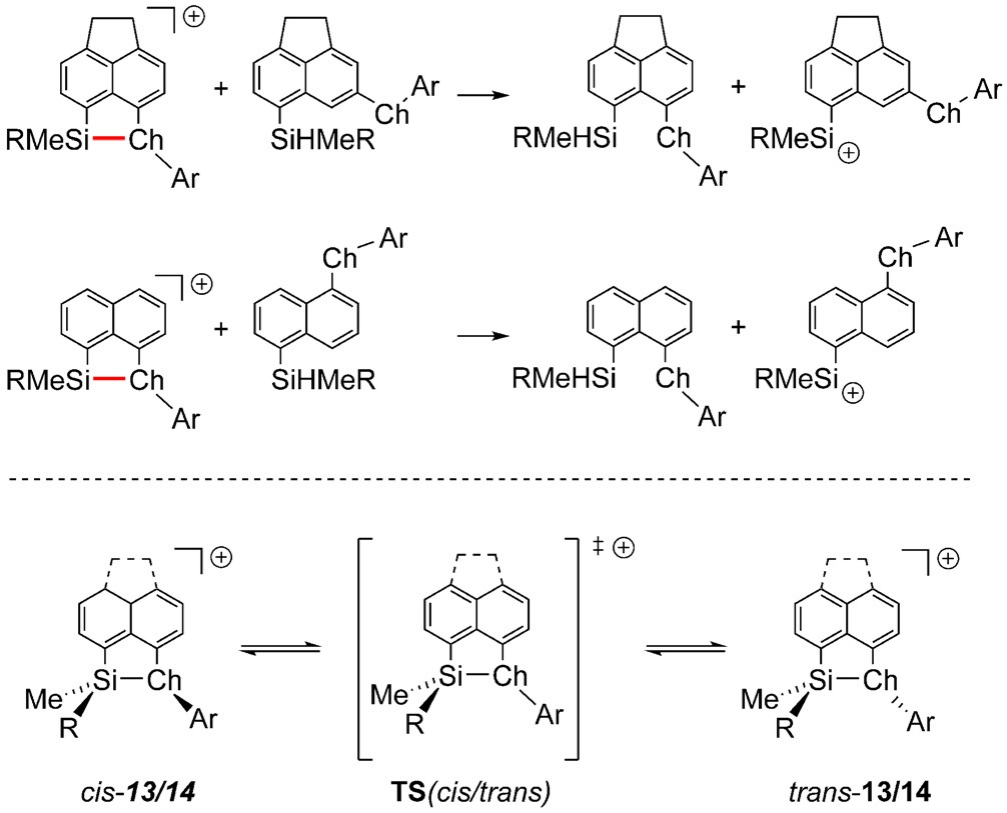
Isodesmic reactions for the assessment of the bond dissociation energy of the Si−Ch bond in silyl cations **13**,**14**; and inversion of the configuration of the chalcogen atom (for Ch=S, Se, Te).

In principle, two possible processes could be responsible for the observed configurational instability of the phenylsulfanyl substituted cations **13 c** and **14 a**. The cleavage of the S−Si bond would allow free rotation around both the C−Si and the S−C bond. An alternative process could be the inversion of the configuration at the tricoordinated sulfur atom without breaking the Si−S linkage, similar to the well‐known inversion of configuration in isoelectronic phosphanes and amines. Quantum mechanical calculations at the DFT M06‐2X/def2‐TZVP level of theory were used to distinguish between the two possibilities.^[51]^ In qualitative agreement with the results of the NMR investigation, this model chemistry predicts a relative small preference of the *cis*‐configuration for the SiMePh substituted cations and favors significantly the *trans*‐isomers of the SiMe*t*Bu substituted cations (Table 5). The heterolytic bond dissociation energies (BDE) of the Si−S bonds, as obtained from the isodesmic reaction shown in Scheme 5 are in the range of BDE=141–169 kJ mol^−1^ (Table 5) and are similar to those reported for the dimethylsilyl derivatives (BDE=144 and 188 kJ mol^−1^).^[38]^ In short, we note that the calculated BDEs are significantly larger than the measured free activation enthalpies Δ*G* ^‡^ (see Table 5). For the non‐dissociative inversion process, the cation passes through a transition state with a trigonal planar coordination environment of the sulfur atom (Scheme 5, TS‐*cis*/*trans*). The calculated free Gibbs energy barriers (Δ*G* ^calc^) for this process are for the series phenylsulfanyl‐substituted cations **13 c**,**d**, **14 a** in a range of Δ*G* ^‡calc^=71–74 kJ mol^−1^, in almost perfect agreement with the experimental values (Table 5). Therefore, the observed *cis*/*trans* isomerization of phenylsulfanyl‐substituted cations **13 c** and **14 a** proceed via inversion of the configuration of the sulfur atom. The calculated free Gibbs energy barriers of selanyl‐ and tellanyl‐stabilized silyl cations **13 e**, **f** and **14 b** are with Δ*G* ^calc^=90–101 kJ mol^−1^ significantly higher. In these cases, the VT NMR experiments did not lead to coalescence up to *T*=378 K. These results suggest, that selanyl‐ and tellanyl‐stabilized silyl cations **13 e**,**f** and **14 b** are configurationally more stable than their phenylsulfanyl‐stabilized counterparts **13 c** and **14 a**. Consequently, not only the choice of the substitution pattern (as described above) controls the configuration of chalcogenyl‐stabilized silyl cation but also the choice of the stabilizing donor group.

**Table 5:** Experimentally obtained free Gibbs activation enthalpies for the *cis*→*trans* isomerization in stabilized silyl cations, calculated energies for the inversion of the configuration of the chalcogen atom and the BDE of the Si‐Ch bond (M06‐2X/def2‐TZVP, all energies are given in kJ mol^−1^).

Compound	Δ*G* ^‡^	Δ*G* ^‡calc^	BDE	Δ*G* ^calc^ (*cis*→*trans*)
**13 a** OPh, Ph			94	
**13 c** SPh, Ph	74	74	141	4
**13 d** SPh, *t‐*Bu	–	71	164	−13
**13 e** SePh, Ph	–	93	138	2
**13 f** TeMes, Ph	–	101	152	1
**14 a** SPh, Ph	72	71	169	1
**14 b** SePh, Ph	–	90	166	1
**14 c** SePh, *t‐*Bu	–	95	196	−13

**Scheme 6:**
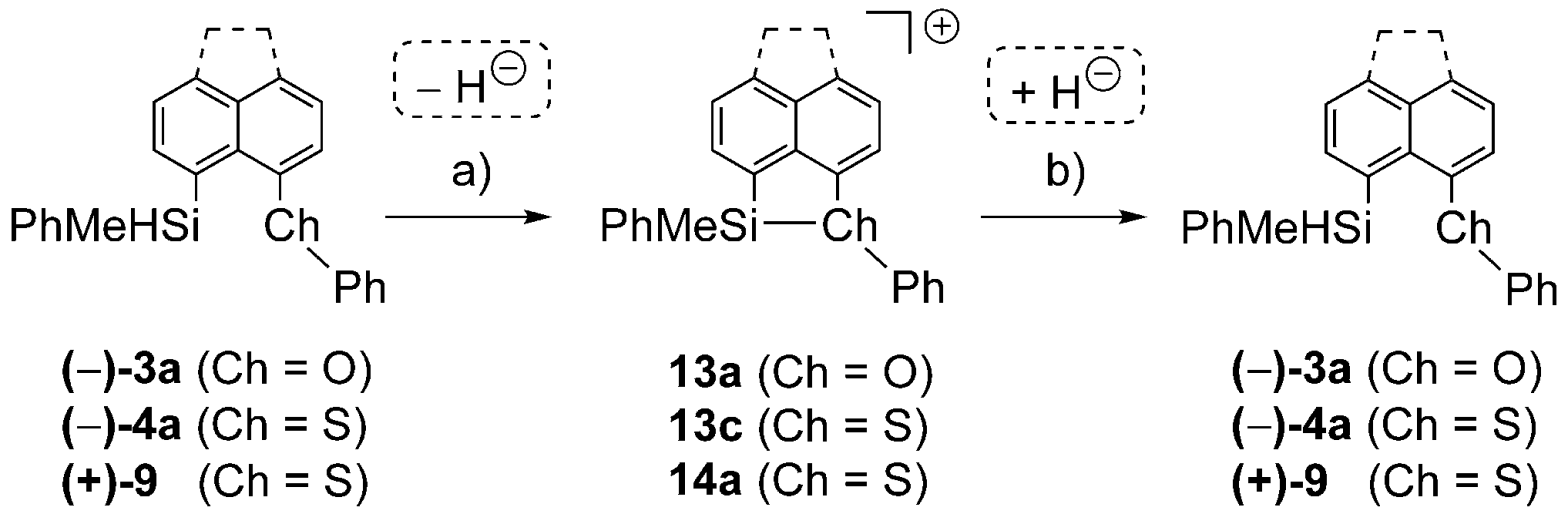
Test for the chiral memory of silyl borates **13** and **14**[B(C_6_F_5_)_4_] (The anion is not shown, conditions: a) 1 equiv [Ph_3_C][B(C_6_F_5_)_4_], C_6_H_5_Cl, r.t. or −40 °C, 30 min; b) 1 equiv Na[Et_3_BH], C_6_H_5_Cl, r.t.,16 h or −40 °C, 30 min then to r.t.).

In the next step, we tested if the neighboring group effect of the chalcogenyl substituent can be used to preserve stereochemical information at the silicon atom during the generation of silyl borates from chiral silanes. For this purpose we followed the chiral memory strategy developed by Landais and co‐workers.[^36^, ^37^] The enantiomerically enriched silanes **3 a**, **4 a** und **9** were treated with trityl borate. The resulting silyl borates **13 a**, **13 c** and **14 a**[B(C_6_F_5_)_4_] were subsequently reduced to regenerate the precursor silanes. The obtained silanes were then analyzed regarding their enantiopurity (Scheme 6).

The test reactions were carried out in chlorobenzene at different temperatures and their results are summarized in Table 6. The reaction of naphthyl‐substituted silane (+)‐**9** (*ee*=84 %) was carried out at +20 °C. After purification, the recovered silane **9** showed no enantiomeric excess indicating complete racemization (Table 6, Entry 1). The reaction of the phenoxy‐stabilized silane **(−)‐3 a** (*ee*=54 %) was carried out at −40 °C (Table 6, Entry 2). After work up and purification an enantiomeric excess of *ee*=32 % was obtained, consequently a racemization of about 41 % has occurred. The experiment was repeated at −80 °C in dichloromethane but only the decomposition of the silyl borate **13 a**[B(C_6_F_5_)_4_] was observed, due to reaction with the solvent. The best result was obtained with phenylsulfanyl‐substituted silane **(−)4 a**. At −40 °C, the complete conservation of the chiral information was observed (Table 6, Entry 4).

**Table 6:** Results of the chiral memory test for silanes **3 a**, **4 a**, **9**.

Entry	silane^[a]^	solvent	*T* [°C]	*ee* start [%]	*ee* end [%]
1	**9**	C_6_H_5_Cl	+20	84	0
2	**3 a**	C_6_H_5_Cl	−40	54	32
3	**3 a**	CH_2_Cl_2_	−80	decomposition	
4	**4 a**	C_6_H_5_Cl	−40	64	64

**Figure 7:**
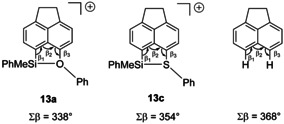
The sum of the bay angles Σβ as a qualitative measure for strain from calculated molecular structures of cations **13 a** and **13 c** (at M062X/def2‐TZVP) and. The value for acenaphthene is given for comparison.

These results are in agreement with our previous conclusions that in *peri*‐acenaphthyl substituted silyl cations similar to **13** the phenylsulfanyl substituent is a more efficient donor group than the phenoxy group.^[38]^ The small size of the oxygen atom creates significant strain that weakens the Si−O interaction and leads to loss of stereochemical information at the silicon atom. This idea is supported by comparison of the calculated structures of cations **13 a** and **13 c** (Figure 7). The sum of the bay angles Σβ is significantly smaller for cation **13 a** than for the phenylsulfanyl substituted cation **13 c** and non‐substituted acenaphthene,^[54]^ which indicates decreasing strain in this series. In agreement, the calculated BDE for the Si−O bond in cation **13 a** is 94 kJ mol^−1^, only 2/3 of that predicted for the phenylsulfanylstabilized cation **13 c** (Table 5). Landais and co‐workers observed a similar decisive influence of strain on the configurational stability of chiral silicon centers in their recent study on pyridyl‐ and quinoline‐stabilized chiral silyl cations **VI** and **VII**.^[37]^

**Scheme 7:**
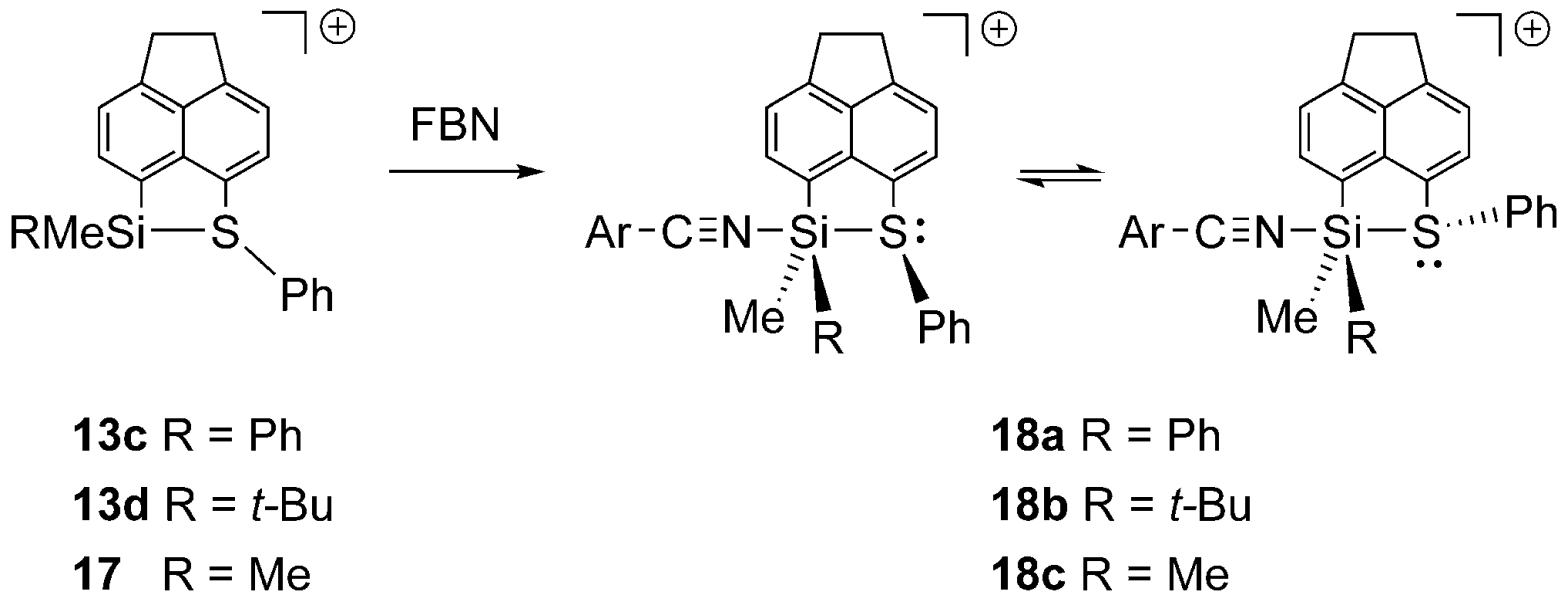
Formation of nitrilium borates **17**[B(C_6_F_5_)_4_] from phenylsulfanyl substituted silyl borates **13 c**, **d**[B(C_6_F_5_)_4_] (FBN: *p*‐fluorobenzonitrile, Ar=*p*‐F‐C_6_H_4_, the [B(C_6_F_5_)_4_]^−^ anion is not shown).

**Figure 8:**
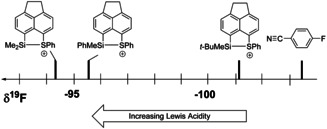
Substituent effect on the Lewis acidity for phenylsulfanyl‐stabilized silyl cations based on the ^19^F NMR chemical shift of the corresponding FBN complexes.

Recently, our group developed an experimental acidity scale for the assessment of the Lewis acidity of intramolecularly stabilized silyl cations using *para*‐fluorobenzonitrile (FBN) as a NMR chemical shift probe.^[39]^ This method relies on the fact, that coordination of the nitrile to the silicon atom does not cancel the intramolecular interaction of the donor group with the silicon atom. Thereby, it provides a measure of the actual Lewis acidity of the stabilized silyl cation. The *para*‐fluorine atom of the coordinated nitrile is used as the reporter nuclei. The stronger the Lewis acid, the more deshielded is the fluorine atom. We tested the effect of the substituent at silicon on the Lewis acidity in phenylsulfanyl stabilized silyl cations using the FBN probe (Scheme 7, Figure 8). The ^19^F NMR chemical shifts of the formed nitrile complexes **18** are δ^19^F=−95.5 (**18 a**) and δ^19^F=−101.3 (**18 b**). This indicates that the Lewis acidity of both here tested cations **13 c**,**d** is smaller than that of the dimethylsilyl substituted cation **17** (δ^19^F=−94.3 (**18 c**). The effect of the phenyl substituent in **13 c** on the Lewis acidity is small and expected due to its electron donating ability. On the first view surprising is the large decrease in Lewis acidity by the *tert‐*butyl substituent. The ^19^F NMR chemical shift of the complex **18 b** is close to that of free FBN (δ^19^F(FBN)=−103.4). We suggest that this is due to steric hindrance of complex formation by the *tert‐*butyl group, rather than to an electronic effect.

Some details of the reaction of the *cis*‐ and *trans*‐mixture of cations **13 c** with FBN are of interest. As expected, the ^29^Si NMR chemical shift of silylnitrilium ion **18 a** is high‐field shifted compared to the corresponding phenylmethyl‐substituted silyl cation **13 c** (δ^29^Si=6.4 vs. δ^29^Si=42.7 (*trans*‐**13 c**), 45.2 (*cis*‐**13 c**)), indicating the increase of the coordination number at the silicon atom. Notably, at r.t., the formation of *cis*‐ and *trans*‐ isomers was not observed (only one set of signals in the ^1^H, ^13^C as well as in the ^29^Si NMR spectra). VT ^1^H NMR spectroscopy of the nitrilium borate **18 a**[B(C_6_F_5_)_4_] showed however severe broadening of the ^1^H NMR signal of the methyl groups at 203 K and two signals below this temperature. The observation of *cis*‐ and *trans*‐isomers of silylnitrilium **18 a** points to an intact Si−S bond and penta‐coordination for the silicon atom. The simultaneous interaction of the donating sulfanyl group and the FBN probe molecule is the premise for an accurate determination of the Lewis acidity of the intramolecularly stabilized Lewis acid. The coalescence temperature corresponds to a free Gibbs energy barrier of the inversion of the configuration at the sulfur atom of Δ*G* ^‡^(203 K)=39 kJ mol^−1^ which is 54 % of that determined for cation **13 c** (Δ*G* ^‡^(378 K)=72 kJ mol^−1^).

## Conclusions

We synthesized a series of chalcogenyl‐substituted acenaphthyl and naphthyl‐silanes **3**, **4**, **6**, **7**, **9**, **10** with an unsymmetrical substituted silicon atom. The chalcogenyl substituent was varied from oxygen to tellurium. For the sub‐set of phenylsulfanyl substituted silanes **3 a**, **4 a** and **9** an established protocol relying on kinetic resolution^[40]^ allowed the separation of both enantiomers. The silanes were used as starting material for the preparation of potentially chiral intramolecularly chalcogenyl stabilized silyl borates **13**[B(C_6_F_5_)_4_] and **14**[B(C_6_F_5_)_4_] based on the acenaphthene and naphthalene scaffold according to the standard Corey protocol. These cations are characterized by a relatively strong chalcogen—silicon bond (BDE=94–169 kJ mol^−1^). In silyloxonium ions **13 a**,**b**, we found, according to our NMR investigations and in agreement with previous results for related cations,[^38^, ^53^, ^55^, ^56^, ^57^] a trigonal planar configuration at the oxygen atom. In contrast, the higher congeners show a trigonal pyramidal configuration of the chalcogenyl group. This is demonstrated by the formation of a mixture of *cis*‐ and *trans*‐isomers of phenylmethylsilyl cations **13 c**, **e**, **f** and **14 a**, **b**. In the case of the *tert*‐butyl‐substituted cations **13 d** and **14 c** only the *trans*‐isomer was detected by NMR spectroscopy, indicating that the steric bulk at the silicon atom controls the configuration at the chalcogenyl group. The experimentally determined barriers for the inversion of the configuration at the sulfur atoms in phenylsulfanyl‐stabilized silyl cations **13 c** and **14 a** are substantial (Δ*G* ^‡^=72–74 kJ mol^−1^) and close to that measured for isoelectronic neutral silyl‐ and arylphosphanes (Δ*G* ^‡^=69–75 kJ mol^−1^).[^58^, ^59^] Moreover, the experimental data is in almost perfect agreement with the computed barrier for the inversion process using DFT methods at the M062X/def2‐TZVP level (Table 5). The calculations predict an even higher inversion barrier for the selanyl and tellanyl stabilized silyl cations **13 e**, **f** and **14 b**. We used the cationization/reduction sequence proposed by Landais and co‐workers[^36^, ^37^] to demonstrate that the neighboring effect of the phenylsulfanyl substituent in cation **13 c** is sufficiently strong to preserve the chiral information at the silicon atom at −40 °C. In addition, these chiral memory experiments showed that the phenylsulfanyl group is a better donor to the silyl cation than the phenoxy substituent. Finally, the determination of the Lewis acidity using the FBN protocol^[39]^ indicated that Lewis acidity of these intramolecularly stabilized silyl cation can be fine‐tuned by electronic effects of the substituent at silicon.

## Experimental Section

Experimental Details, all relevant analytic information including NMR spectra and computational details can be found in the Supporting Information.

## Conflict of interest

The authors declare no conflict of interest.

## Supporting information

As a service to our authors and readers, this journal provides supporting information supplied by the authors. Such materials are peer reviewed and may be re‐organized for online delivery, but are not copy‐edited or typeset. Technical support issues arising from supporting information (other than missing files) should be addressed to the authors.

Supplementary

Supplementary

## References

[1] J. B. Lambert , L. Kania , S. Zhang , Chem. Rev. 1995, 95, 1191.

[2] T. Müller , Adv. Organomet. Chem. Eds.: West, Hill, Stone, Academic Press, 2005, Vol. 53, pp. 155.

[3] H. F. T. Klare , M. Oestreich , Dalton Trans. 2010, 39, 9176. 20405073 10.1039/c003097j

[4] T. Müller , Functional Molecular Silicon Compounds I (Ed.: Scheschkewitz ), Springer, Cham, 2014, Vol. 155, pp. 107.

[5] V. Y. Lee , A. Sekiguchi , Organosilicon Compounds (Ed.: Lee ), Academic Press, Oxford, 2017, Vol. 1, pp. 197.

[6] V. Y. Lee , Russ. Chem. Rev. 2019, 88, 351.

[7] J. C. L. Walker , H. F. T. Klare , M. Oestreich , Nat. Rev. Chem. 2020, 4, 54.

[8] J. S. Siegel , Nat. Rev. Chem. 2020, 4, 4.

[9] H. F. T. Klare , K. Bergander , M. Oestreich , Angew. Chem. Int. Ed. 2009, 48, 9077; 10.1002/anie.200904520 19862787

[10] A. R. Nödling , K. Müther , V. H. G. Rohde , G. Hilt , M. Oestreich , Organometallics 2014, 33, 302.

[11] R. K. Schmidt , K. Müther , C. Mück-Lichtenfeld , S. Grimme , M. Oestreich , J. Am. Chem. Soc. 2012, 134, 4421. 22309027 10.1021/ja211856m

[12] K. Hara , R. Akiyama , M. Sawamura , Org. Lett. 2005, 7, 5621. 16321006 10.1021/ol052206g

[13] V. J. Scott , R. Çelenligil-Çetin , O. V. Ozerov , J. Am. Chem. Soc. 2005, 127, 2852. 15740111 10.1021/ja0426138

[14] R. Panisch , M. Bolte , T. Müller , J. Am. Chem. Soc. 2006, 128, 9676. 16866520 10.1021/ja061800y

[15] C. Douvris , O. V. Ozerov , Science 2008, 321, 1188. 18755971 10.1126/science.1159979

[16] N. Lühmann , R. Panisch , T. Müller , Appl. Organomet. Chem. 2010, 24, 533.

[17] S. Duttwyler , C. Douvris , N. L. P. Fackler , F. S. Tham , C. A. Reed , K. K. Baldridge , J. S. Siegel , Angew. Chem. Int. Ed. 2010, 49, 7519; 10.1002/anie.201003762 20818636

[18] C. Douvris , C. M. Nagaraja , C.-H. Chen , B. M. Foxman , O. V. Ozerov , J. Am. Chem. Soc. 2010, 132, 4946. 20218686 10.1021/ja100605m

[19] T. Stahl , H. F. T. Klare , M. Oestreich , J. Am. Chem. Soc. 2013, 135, 1248. 23311960 10.1021/ja311398j

[20] G. Meier , T. Braun , Angew. Chem. Int. Ed. 2009, 48, 1546; 10.1002/anie.200805237 19148912

[21] T. Stahl , H. F. T. Klare , M. Oestreich , ACS Catal. 2013, 3, 1578.

[22] L. Omann , Z.-W. Qu , E. Irran , H. F. T. Klare , S. Grimme , M. Oestreich , Angew. Chem. Int. Ed. 2018, 57, 8301; 10.1002/anie.201803181 29741219

[23] M. Devillard , B. de Bruin , M. A. Siegler , J. I. van der Vlugt , Chem. Eur. J. 2017, 23, 13628. 28816371 10.1002/chem.201703798 PMC5656908

[24] M. Johannsen , K. A. Jørgensen , G. Helmchen , J. Am. Chem. Soc. 1998, 120, 7637.

[25] B. Mathieu , L. de Fays , L. Ghosez , Tetrahedron Lett. 2000, 41, 9561.

[26] Z. Tang , B. Mathieu , B. Tinant , G. Dive , L. Ghosez , Tetrahedron 2007, 63, 8449.

[27] V. H. G. Rohde , P. Pommerening , H. F. T. Klare , M. Oestreich , Organometallics 2014, 33, 3618.

[28] V. H. G. Rohde , M. F. Müller , M. Oestreich , Organometallics 2015, 34, 3358.

[29] R. K. Schmidt , H. F. T. Klare , R. Fröhlich , M. Oestreich , Chem. Eur. J. 2016, 22, 5376. 26929105 10.1002/chem.201504777

[30] P. Shaykhutdinova , M. Oestreich , Organometallics 2016, 35, 2768.

[31] P. Shaykhutdinova , S. Kemper , M. Oestreich , Eur. J. Org. Chem. 2018, 2896.

[32] P. Shaykhutdinova , M. Oestreich , Org. Lett. 2018, 20, 7029. 30362776 10.1021/acs.orglett.8b02945

[33] P. Shaykhutdinova , M. Oestreich , Synthesis 2019, 51, 2221.

[34] T. Gatzenmeier , M. Turberg , D. Yepes , Y. Xie , F. Neese , G. Bistoni , B. List , J. Am. Chem. Soc. 2018, 140, 12671. 30277760 10.1021/jacs.8b07092

[35] L. Schreyer , R. Properzi , B. List , Angew. Chem. Int. Ed. 2019, 58, 12761; 10.1002/anie.201900932 30840780

[36] P. Ducos , V. Liautard , F. Robert , Y. Landais , Chem. Eur. J. 2015, 21, 11573. 26139434 10.1002/chem.201501987

[37] A. Fernandes , C. Laye , S. Pramanik , D. Palmeira , Ö. Ö. Pekel , S. Massip , M. Schmidtmann , T. Müller , F. Robert , Y. Landais , J. Am. Chem. Soc. 2020, 142, 564. 31814388 10.1021/jacs.9b11704

[38] N. Kordts , S. Künzler , S. Rathjen , T. Sieling , H. Großekappenberg , M. Schmidtmann , T. Müller , Chem. Eur. J. 2017, 23, 10068. 28374465 10.1002/chem.201700995

[39] S. Künzler , S. Rathjen , A. Merk , M. Schmidtmann , T. Müller , Chem. Eur. J. 2019, 25, 15123. 31469201 10.1002/chem.201903241 PMC6899571

[40] S. Rendler , O. Plefka , B. Karatas , G. Auer , R. Fröhlich , C. Mück-Lichtenfeld , S. Grimme , M. Oestreich , Chem. Eur. J. 2008, 14, 11512. 19021177 10.1002/chem.200801377

[41] H. Duddeck , Progr. NMR Spectroscopy 1995, 27, 1.

[42] L. M. Diamond , F. R. Knight , K. S. Athukorala Arachchige , R. A. M. Randall , M. Bühl , A. M. Z. Slawin , J. D. Woollins , Eur. J. Inorg. Chem. 2014, 1512.

[43] T. Nakai , M. Nishino , S. Hayashi , M. Hashimoto , W. Nakanishi , Dalton Trans. 2012, 41, 7485. 22584428 10.1039/c2dt30516j

[44] J.-C. Hierso , Chem. Rev. 2014, 114, 4838. 24533483 10.1021/cr400330g

[45] F. B. Mallory , J. Am. Chem. Soc. 1973, 95, 7747.

[46] E. Hupf , E. Lork , S. Mebs , J. Beckmann , Organometallics 2015, 34, 3873.

[47] J. B. G. Lambert , S. Gronert , H. F. Shurvell , D. A. Lightner , Spektroskopie: Strukturaufklärung in der Organischen Chemie, Pearson Deutschland, 2012.

[48] F. R. Knight , A. L. Fuller , M. Bühl , A. M. Z. Slawin , J. D. Woollins , Chem. Eur. J. 2010, 16, 7503. 20468030 10.1002/chem.200903523

[49] M. Mantina , A. C. Chamberlin , R. Valero , C. J. Cramer , D. G. Truhlar , J. Phys. Chem. A 2009, 113, 5806. 19382751 10.1021/jp8111556 PMC3658832

[50] E. Hupf , M. Olaru , C. I. Raţ , M. Fugel , C. B. Hübschle , E. Lork , S. Grabowsky , S. Mebs , J. Beckmann , Chem. Eur. J. 2017, 23, 10568. 28395126 10.1002/chem.201700992

[51] All calculations were performed using the Gaussian 16 program. See the Supporting Information for details.

[52] J. Y. Corey , J. Am. Chem. Soc. 1975, 97, 3237.

[53] K. Bläsing , R. Labbow , D. Michalik , F. Reiß , A. Schulz , A. Villinger , S. Walker , Chem. Eur. J. 2020, 26, 1640. 31738450 10.1002/chem.201904403 PMC7028070

[54] A. C. Hazell , R. G. Hazell , L. Norskov-Lauritsen , C. E. Briant , D. W. Jones , Acta Crystallogr. Sect. C 1986, 42, 690.

[55] Z. Xie , R. Bau , C. A. Reed , J. Chem. Soc. Chem. Commun. 1994, 2519.

[56] M. Driess , R. Barmeyer , C. Monsé , K. Merz , Angew. Chem. Int. Ed. 2001, 40, 2308; 10.1002/1521-3773(20010618)40:12<2308::AID-ANIE2308>3.0.CO;2-Q 29711844

[57] A. Schäfer , M. Reißmann , A. Schäfer , M. Schmidtmann , T. Müller , Chem. Eur. J. 2014, 20, 9381.24942460

[58] R. T. Boeré , J. D. Masuda , Can. J. Chem. 2002, 80, 1607.

[59] M. A. Petrie , P. P. Power , J. Chem. Soc. Dalton Trans. 1993, 1737.

