# Supplementary material for: Chiral Chalcogenyl‐Substituted Naphthyl‐ and Acenaphthyl‐Silanes and Their Cations
Source: Chemistry. 2020 Oct 27;26(69):16441–9. doi: 10.1002/chem.202002977 (PMC7756486; doi:10.1002/chem.202002977)
Supplement: Supplementary file 1 — Supplementary [file CHEM-26-16441-s001.pdf]

Chemistry–A European Journal

Supporting Information

Chiral Chalcogenyl-Substituted Naphthyl- and Acenaphthyl-Silanes and Their Cations

Sandra Künzler,[a] Saskia Rathjen,[a] Katherina Rüger,[a] Marie S. Würdemann,[a] Marcel Wernke,[a] Patrik Tholen,[a] Corinna Girschik,[a] Marc Schmidtmann,[a] Yannick Landais,[b] and Thomas Müller*[a]

Supporting Information

for

Chiral Chalcogenyl-Substituted Naphthyl- and Acenaphthylsilanes and their Cations

Sandra Künzler, Saskia Rathjen, Katherina Rüger, Marie S. Würdemann, Marcel Wernke, Patrik Tholen, Corinna Girschik, Marc Schmidtmann, Yannick Landais and Thomas Müller*

Table of Contents

1.	Experimental Part	2
1.1	General remarks	2
1.2	Synthesis and characterization of silanes	4
1.2.1	Synthesis and characterization of bromo-substituted silanes	5
1.2.2	Synthesis and characterization of chalcogenyl-substituted silanes	12
1.3	Synthesis and characterization of chalcogenyl-stabilized silyl borates	35
1.4	Chiral Resolution and Chiral Memory Experiments	67
1.5	Data from X-ray diffraction analysis of compounds **3a, 4a and 4b**	79
2.	Computational Details	82
3.	References	85

1. Experimental part

1.1 General remarks

All experiments were carried out under argon or nitrogen atmosphere using Schlenk techniques. The glass equipment was stored in an oven at 120°C and evacuated prior to use. The solvents *n*-pentane, *n*-hexane, benzene, tetrahydrofuran and diethyl ether were dried over sodium-potassium alloy and distilled under nitrogen atmosphere. The deuterated solvents were first dried over NaK and then either condensed before use or stored over molecular sieve (4 Å). Commercially available solid materials were stored and weighted in a glove box or dried at high vacuum before use. The *n*-butyl lithium was used as a 1.6 M solution in *n*-hexane. 1,8-Dibromonaphthalene, 5,6-dibromoacenaphtene, 1-Bromo-8-dimethylsilylnaphthalene, trityl borate $[Ph_3C][B(C_6F_5)_4]$ were synthesized according to literature procedures.[S1-S4]

Thin-layer chromatography was performed using commercial available aluminium foil (Fluka) coated with silica gel 60 and fluorescent indicator F254. For the column chromatography silica gel of the mesh size 60 from Merck was used. For preparative TLC, the pre-coated TLC plates *SIL G-200* from MACHEREY-NAGEL were used.

GC/MS spectra were performed on a Thermo Focus DSQ (stationary phase: DB5 column, length 25 m, diameter 0.2 mm, film thickness 0.33 µm; temperature program: $T_{initial}$ = 60°C for 5 min, then heating with 10°C/min to T_{end} = 280°C, staying at this temp. for 10 min; detector: EI with 70 eV) or on a Shimadzu GCMS-QP2020 equipped with a Macherey-Nagel Optima 5 HT column (30 m, 0.25 mm ID, 0.25 µm film thickness) and a Shimadzu *GC/MS-QP2020 mass selective* detector. High resolution mass spectra were measured on a Finnigan-LCQ or a Finnigan-MAT95 spectrometer using ESI, CI or EI.

GC spectra were performed on a Shimadzu *GC-2010 Plus* equipped with a Macherey-Nagel Optima 5 MS column (15 m, 0.25 mm ID, 0.25 µm film thickness) and a flame ionization detector.

Chiral GC spectra were measured on a GC-2010 Plus from Shimadzu. The column used, is a FS-Lipodex E [Octakis-(2,3-di-O-pentyl-3-butyryl)-γ-cyclodextrin, 25 m, 0.25 mm, constant flow, carrier gas hydrogen: 1.5 cm^3min^{-1}] from Macherey-Nagel.

Chiral HPLC was performed on a Thermo Scientific Dionex Ultimate 3000 with a Lux 5µm Cellulose-3, 250 x 4.6 mm column and a flow rate of the eluent of 1mL/min at 25°C.

The determination of the optical rotation was performed using the Polartronic M from Schmidt+Haensch with Na-D-line (589 nm) at 20°C (cell lengths: 1 dm).

Infrared spectra were performed on a Bruker Tensor 27 spectrometer with a MKII Reflection Golden Gate Single Diamond ATR system.

NMR spectra were recorded on Bruker Avance 500, Bruker Avance III 500 spectrometer. ^1H NMR spectra were referenced to the residual solvent resonance as internal standard (benzene-d_6: $\delta^1H(C_6D_5H)$ = 7.20, toluene-d_8: $\delta^1H(C_6D_5CD_2H)$ = 2.08, chloroform-d_1:

$δ^1H(CHCl_3)$ = 7.24, chlorobenzene-d_5: $δ^1H(C_6D_4HCl)$ = 7.14) and ^{13}C NMR spectra by using the central line of the solvent signal (benzene-d_6: δ13C(C_6D_6) = 128.0, toluene-d_8: $δ^{13}C(C_6D_5CD_3)$ = 20.4, chloroform-d_1: $δ^{13}C(CDCl_3)$ = 77.0, chlorobenzene-d5: $δ^{13}C(C_6D_5Cl)$ = 134.2). $^{29}Si\{^1H\}$ NMR spectra were referenced to an external standard ($^{29}Si(Me_2SiHCl)$ = 11.1 versus tetramethylsilane (TMS)). ^{77}Se NMR spectra against external Me2Se ($δ^{77}Se(Me_2Se)$ = 0.0), ^{125}Te NMR spectra against external Ph_2Te_2 ($δ^{125}Te(Ph_2Te_2)$ = 422.0), ^{19}F NMR spectra against external $CFCl_3$ ($δ^{19}F(CFCl_3)$ = 0.0), and ^{11}B NMR spectra against $BF_3·OEt_2$ ($δ^{11}B(BF_3·OEt_2)$ = 0.0). The $^{29}Si\{^1H\}$ NMR inverse gated spectra were recorded with a relaxation delay D1 = 10 s. The $^{29}Si\{^1H\}$ INEPT spectra were recorded with delays D3 = D4 = 0.001 (for silanes) D3 = 0.0122 s and D4 = 0.0313 s (for silyl borates), if not given otherwise. For silanes, combustion analysis values for carbon show often too low values, which we attribute to the formation and incomplete combustion of silicon carbide, although vanadium pentoxide as combustion aid was used. Satisfactory combustion analyses could not be obtained from all silyl borates due to their high reactivity.

1.2 Synthesis and characterization of silanes

General procedure A: The starting material was dissolved in THF and cooled to -80 °C. Then *n*-butyl lithium was added dropwise. The reaction mixture was stirred for 60 min at that temperature. After the dropwise addition of the corresponding chlorosilane, the mixture was stirred for another 60 min at -80 °C and was then allowed to warm to room temperature over night. Thereafter, an aqueous NH_4Cl solution (ca. 20 mL) was added to the reaction mixture and the product was extracted with Et_2O (3 x 20 mL). The combined organic layer was dried over Na_2SO_4 and the solvent was removed under reduced pressure. The product was purified by column chromatography or crystallization.

General procedure B: The starting material was dissolved in THF and cooled to -80°C. Then *n*-butyl lithium was added dropwise and the reaction mixture was stirred for 70 minutes. During this time, the reaction mixture was allowed to warm to -30°C. Subsequently, the reaction mixture was cooled to -70°C and chlorodimethylsilane was added. The mixture was stirred for additional 30 minutes at -70°C and then warmed to r.t. over night. After completion of the reaction, NH_4Cl solution (10 mL) was added to the reaction mixture and the product was extracted with Et_2O (3 x 10 mL). The organic layer was dried over Na_2SO_4 and the solvent was removed under low pressure. The product was purified by column chromatography or crystallization.

General Procedure C: The starting material was dissolved in THF and cooled to -80 °C. Then *n*-butyl lithium was added dropwise. The reaction mixture was stirred for 60 min at that temperature. A solution of diphenyl disulfide or diphenyl diselenide in THF was added dropwise and the mixture was stirred for another 60 min at -80 °C. Then the mixture was allowed to warm to room temperature over night. An aqueous NH_4Cl solution (ca. 20 mL) was added to the reaction mixture and the product was extracted with Et_2O (3 x 20 mL). The combined organic layer was dried over Na_2SO_4 and the solvent was removed under reduced pressure. The product was purified by column chromatography or crystallization.

1.2.1 Synthesis and characterization of bromo-substituted silanes

8-Phenylmethylsilyl-1-bromonaphthalene 8a

The title silane was synthesized according to General Procedure **A** using 1.0 equiv. of 1,8-dibromonaphthalene (10.00 mmol, 2.86 g), 1.0 equiv. of *n*-butyllithium (10.00 mmol, 6.25 mL) and 1.0 equiv. of phenylmethylchlorosilane (10.00 mmol, 1.57 g). The product was purified by column chromatography using petroleum ether as eluent (R_F = 0.18). 8-Phenylmethylsilyl-1-bromonaphthalene was obtained as a yellow oil. Yield 3.00 g (9.10 mmol; 91 %).

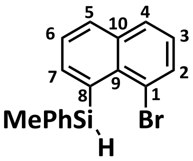

^{1}H NMR (500.13 MHz, 297.4 K, C_6D_6) δ = 0.87 (d, $^3J_{H,H}$ = 3.6 Hz, 3 H, $SiCH_3$), 6.01 (q, $^3J_{H,H}$ = 3.6 Hz, $^1J_{Si,H}$ = 203.5 Hz, 1 H, SiH), 6.82 (t, $^3J_{H,H}$ = 7.8 Hz, 1 H, 3-H), 7.08 (t, $^3J_{H,H}$ = 7.6 Hz, 1 H, 6-H), 7.19-7.22 (m, 3 H, *o*-Ph-H, 2-H), 7.42 (dd, $^3J_{H,H}$ = 8.1 Hz, $^4J_{H,H}$ = 1.0 Hz, 1 H, 4-H), 7.51 (dd, $^3J_{H,H}$ = 8.2 Hz, $^4J_{H,H}$ = 1.0 Hz, 1 H, 5-H), 7.58-7.61 (m, 3 H, *m*-Ph-H, *p*-Ph-H), 8.01 (dd, $^3J_{H,H}$ = 7.0 Hz, $^4J_{H,H}$ = 1.2 Hz, 1 H, 7-H). **^{13}C{^{1}H} NMR** (125.77 MHz, 297.6 K, C_6D_6) δ = -0.1 ($SiCH_3$), 123.8 (C-1), 125.7 (C-6), 126.1 (C-3), 128.2 (*m*-PhCH), 129.2 (*p*-PhCH), 129.6 (C-4), 131.9 (C-5), 132.6 (C-2), 134.6 (C-8), 134.9 (*o*-PhCH), 136.5 (C-9), 137.3 (C-10), 139.3 ($SiC(CH_5)$), 141.0 (C-7). **^{29}Si{^{1}H} INEPT NMR** (99.31 MHz, 673.2 K, C_6D_6, D_3 = 0.0013, D_4 = 0.0013) δ = -13.9. **GC/MS** t_R: 23.7 min, m/z (%) 329 [M^++2] (1), 327 [M^+] (2), 325 (2), 313 (7), 311 (8), 250 (65), 248 (67), 231 (46), 215 (17), 202 (38), 179 (10), 168 (100), 154 (28), 141 (52), 126 (15), 123 (18), 115 (23), 109 (22), 107 (19), 105 (35), 103 (13), 91 (10), 77 (26), 63 (6), 53 (29). **IR** (ATR, 298 K, neat): ν = 2103, 2152 cm^{-1}. **HR/MS** (LIFDI) $C_{17}H_{15}{}^{79}Br^{28}Si$, calc.: 326.0121, found: 326.0122. **EA** $C_{17}H_{15}BrSi$, calculated: C 62.39, H 4.62, found: C 62.69, H 4.95.

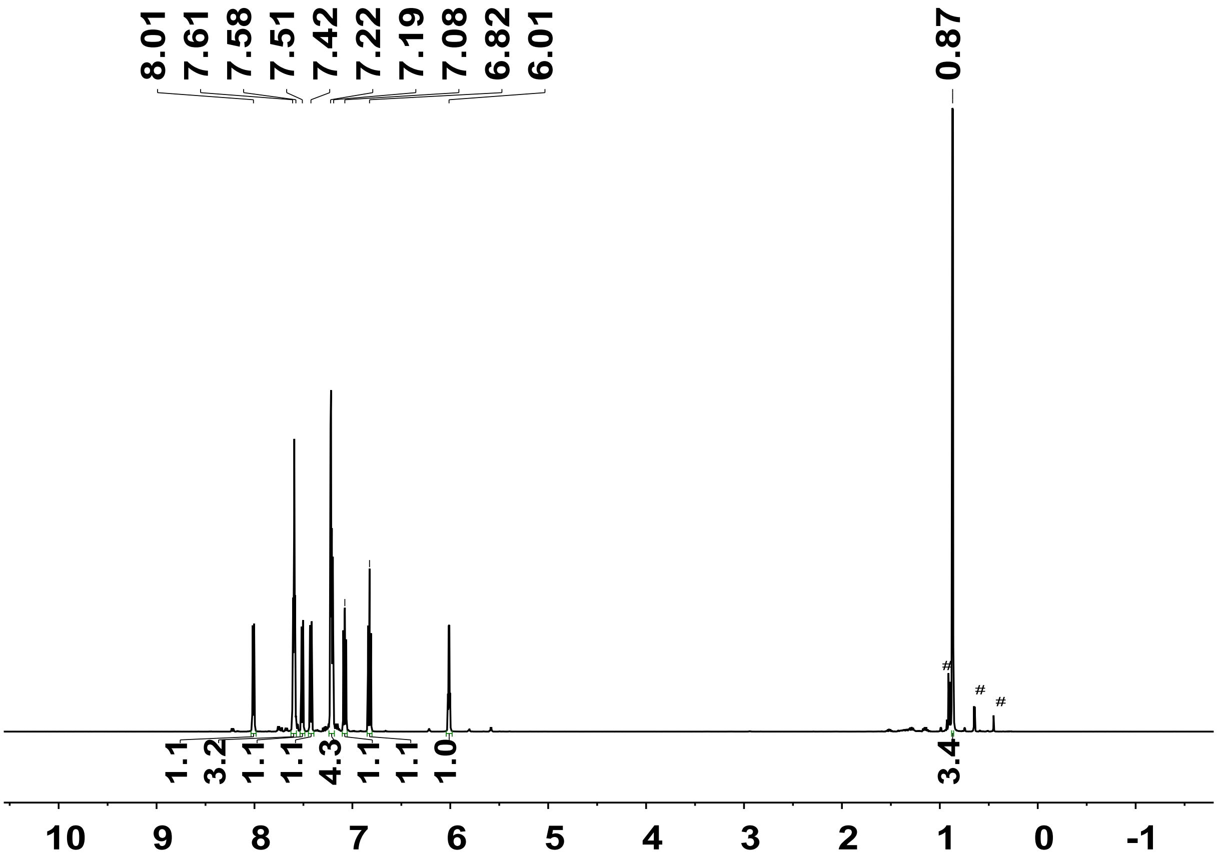

Figure S 1 – ^{1}H NMR spectrum (500.13 MHz, 297.4 K, C_6D_6) of 8-phenylmethylsilyl-1-bromonaphthalene **8a** (# impurities).

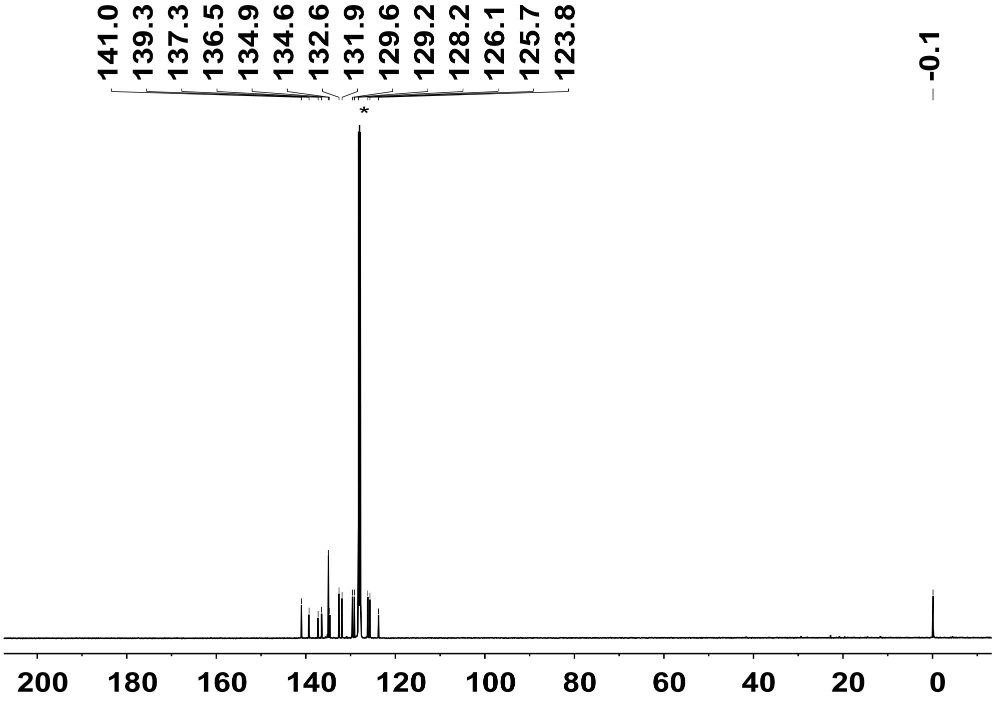

Figure S 2 – $^{13}C\{^{1}H\}$ NMR spectrum (125.77 MHz, 297.6 K, C_6D_6) of 8-phenylmethylsilyl-1-bromonaphthalene **8a** (*C_6D_6).

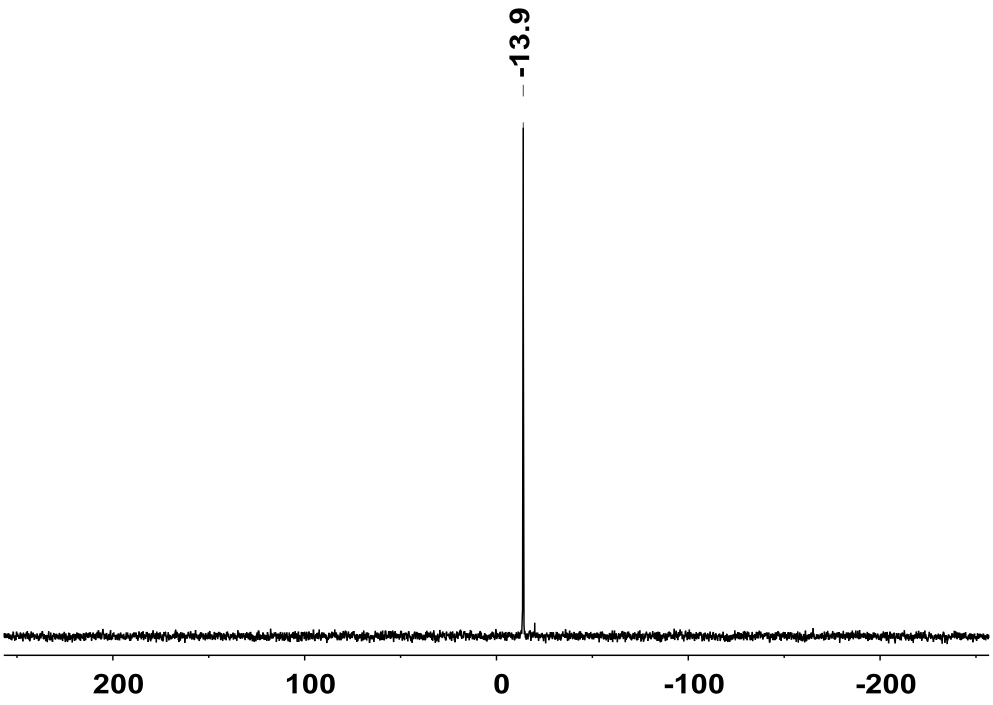

Figure S 3 – $^{29}Si\{^1H\}$ INEPT NMR spectrum (99.31 MHz, 673.2 K, C_6D_6) of 8-phenylmethylsilyl-1-bromonaphthalene **8a**.

8-*tert*-Butylmethylsilyl-1-bromonaphthalene, 8b

The title compound was synthesized according to general procedure **A** using 0.40 g (1.40 mmol) 1,8-Dibromonaphthalene, 0.74 mL (1.40 mmol) *tert*-butyl lithium and 0.19 g (1.40 mmol) chloro(*tert*-butyl)methylsilane. The by-product 1-bromonaphthalene was removed by bulb to bulb distillation (78°C, 0.13 mbar). The 8-*tert*-butylmethylsilyl-1-bromonaphthalene **8b** was obtained as a yellow oil. Yield 0.20 g (0.64 mmol; 46 %).

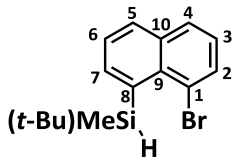

^{1}H NMR (500.13 MHz, 298.4 K, C_6D_6) δ = 0.60 (d, $^3J_{H,H}$ = 3.7 Hz, 3 H, $SiCH_3$), 1.08 (s, 9 H, $C(CH_3)_3$), 5.56 (q, $^3J_{H,H}$ = 3.7 Hz, $^1J_{Si,H}$ = 201.2 Hz, 1 H, SiH), 6.86 (t, $^3J_{H,H}$ = 7.7 Hz, 1 H, 3-H), 7.17 (t, $^3J_{H,H}$ = 7.5 Hz, 1 H, 6-H), 7.44 (dd, , $^3J_{H,H}$ = 8.1 Hz, $^4J_{H,H}$ = 1.1 Hz, 1 H, 4-H), 7.52 (dd, $^3J_{H,H}$ = 8.1 Hz, $^4J_{H,H}$ = 1.1 Hz, 1 H, 5-H), 7.70 (dd, $^3J_{H,H}$ = 8.0 Hz, $^4J_{H,H}$ = 1.4 Hz, 1 H, 2-H), 7.97 (dd, $^3J_{H,H}$ = 7.0 Hz, $^4J_{H,H}$ = 1.2 Hz, 1 H, 7-H). **$^{13}C\{^1H\}$ NMR** (125.77 MHz, 298.6 K, C_6D_6) δ = -2.5 ($SiCH_3$), 18.2 ($SiC(CH_3)_3$), 28.9 ($SiC(CH_3)_3$), 123.6 (C-1), 125.1 (C-6), 125.9 (C-3), 129.7 (C-4), 131.5 (C-5), 133.0 (C-2), 135.7 (C-8), 136.5 (C^{10}), 137.2 (C^9), 139.0 (C-7). **$^{29}Si\{^1H\}$ NMR** (99.31 MHz, 305.0 K, C_6D_6): δ = -0.7. **^{29}Si INEPT NMR** (99.31 MHz, 305.0 K, C_6D_6, D3 = D4 = 0.0013 s) δ = -0.7 (d, $1J_{Si,H}$ = 201.9 Hz). **GC/MS** t_R: 20.1 min, m/z (%) 307 [M^+-H, 37Cl] (0.3),

305 [M$^+$-H, 35Cl] (0.3), 291 (0.8), 249 (56), 235 (3), 183 (3), 169 (100), 167 (49), 155 (16), 141 (15), 129.1 (7), 115 (14), 107 (7), 77 (4), 57 (6). **IR** (ATR, 298 K, neat): v = 2100, 2170 cm^{-1} **HR/MS** (LIFDI) $C_{15}H_{19}{}^{79}Br^{28}Si$, calculated: 306.0434, found: 306.0442. **EA** $C_{15}H_{19}BrSi$, calc.: C 58.63, H 6.23, found: C 58.72, H 6.49.

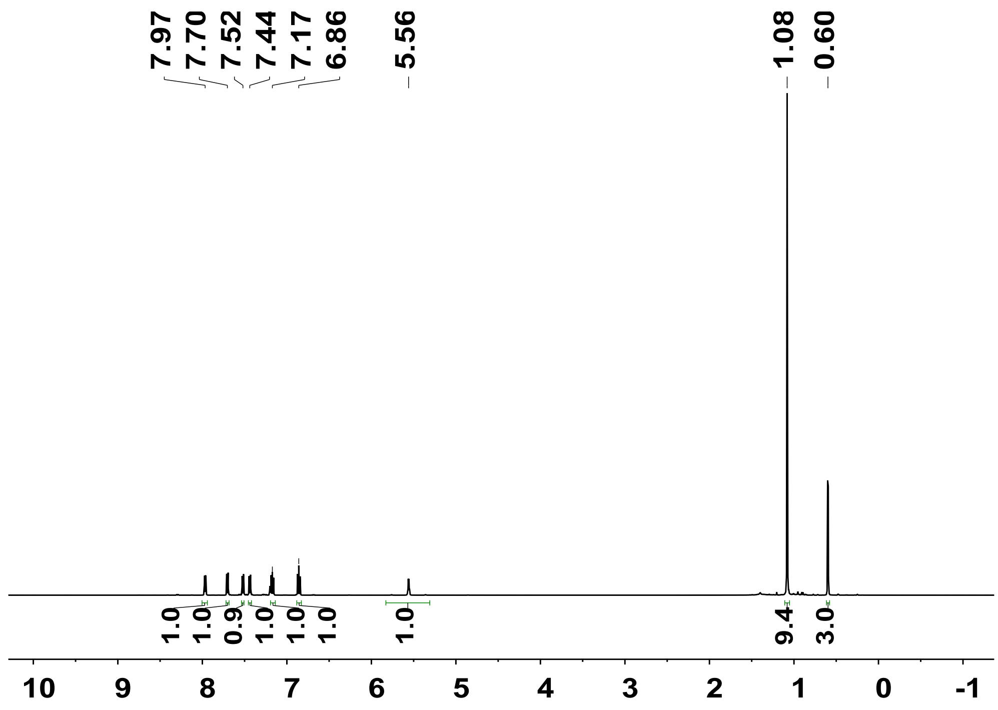

Figure S 4 – ^{1}H NMR spectrum (500.13 MHz, 298.4 K, C_6D_6) of 8-*tert*-butylmethylsilyl-1-bromonaphthalene **8b**.

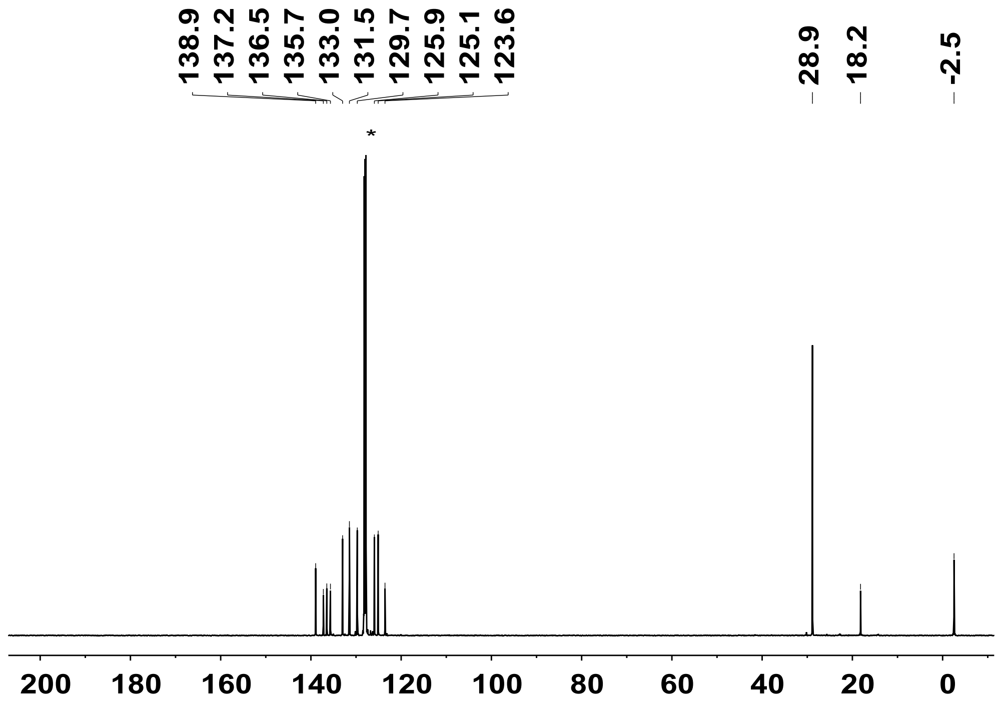

Figure S 5 – $^{13}C\{^1H\}$ NMR spectrum (125.77 MHz, 298.6 K, C_6D_6) of 8-*tert*-butylmethylsilyl-1-bromonaphthalene **8b** (*C_6D_6).

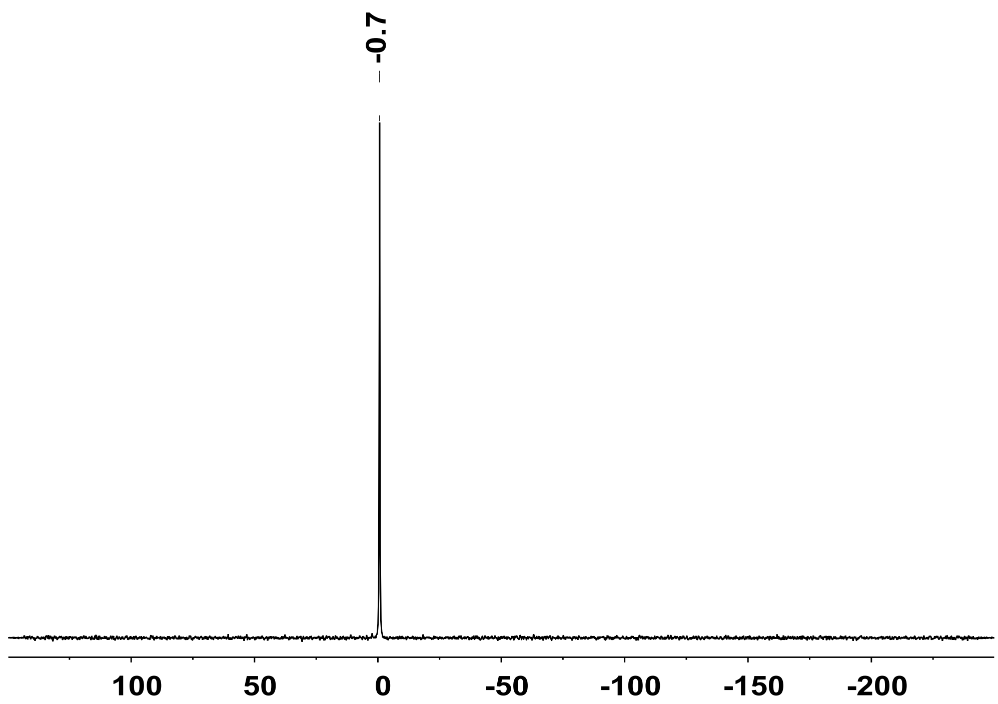

Figure S 6 – $^{29}Si\{^1H\}$ INEPT NMR spectrum (99.31 MHz, 305.0 K, C_6D_6) of 8-*tert*-butylmethylsilyl-1-bromonaphthalene **8b**.

6-Phenylmethylsilyl-5-bromoacenaphthene 5

The title compound was synthesized according to general procedure **A** using 1.87 g (6.00 mmol) 5,6-dibromoacenaphthene, 3.8 mL (6.00 mmol) *n*-butyl lithium and 0.89 mL (6.00 mmol) chloro(methyl)phenylsilane. The product was purified by crystallization from hexanes at r.t. and was obtained as a yellow solid. Yield 1.88 g (5.32 mmol, 89 %).

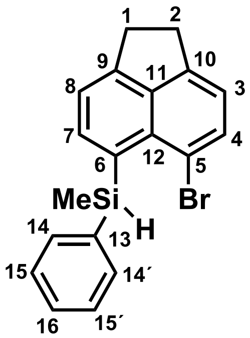

^{1}H NMR (499.87 MHz, 305.1 K, C_6D_6): δ = 0.93 (d, 3 H, $^3J_{H,H}$ = 3.7 Hz, $SiCH_3$), 2.77-2.82 (m, 2 H, 2-H), 2.85-2.90 (m, 2 H, 1-H), 6.07 (q, 1 H, $^3J_{H,H}$ = 3.7 Hz, $^1J_{H,Si}$ = 202.2 Hz, SiH), 6.71 (d, 1 H, $^3J_{H,H}$ = 7.4 Hz, 3-H), 6.97 (d, 1 H, $^3J_{H,H}$ = 7.0 Hz, 8-H), 7.22-7.26 (m, 3 H, 15-H, 15´-H, 16-H), 7.61 (d, 1 H, $^3J_{H,H}$ = 7.4 Hz, 4-H), 7.64-7.70 (m, 2 H, 14-H, 14´-H), 7.99 (d, 1 H, $^3J_{H,H}$ = 7.0 Hz, 7-H). **$^{13}C\{^1H\}$ NMR** (125.71 MHz, 305.0 K, $CDCl_3$): δ = -0.6 (CH_3), 29.8 (CH_2), 30.4 (CH_2), 118.1 (C), 119.9 (CH), 120.6 (CH), 128.0 (CH), 128.7 (C), 129.0 (CH), 133.4 (CH), 134.9 (CH), 135.7 (C), 138.9 (C), 141.2 (C), 141.7 (CH), 146.9 (C), 149.7 (C). **$^{29}Si\{^1H\}$ NMR** (99.31 MHz,

305.0 K, $CDCl_3$): δ = -15.2. **^{29}Si{^{1}H} NMR** (99.36 MHz, 299.6 K, C_6D_6): *δ* = -15.0. **IR** (ATR, 298 K, neat): $\tilde{\nu}$ = 2146 cm^{-1}. **HR/MS** (EI) $C_{19}H_{17}BrSi$, calc.: 352.0277, found: 352.0287. **EA** $C_{19}H_{17}BrSi$ calc.: C 64.59, H 4.85, found: C 64.80, H 5.17.

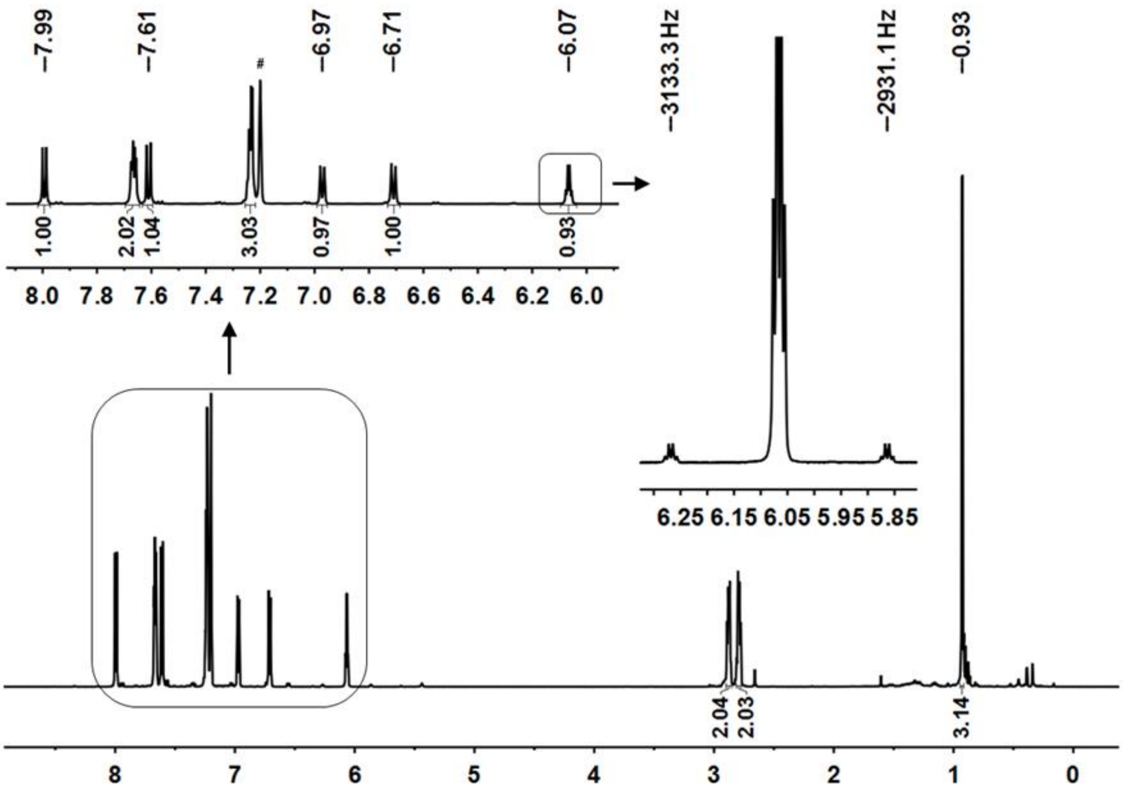

Figure S 7 – ^{1}H NMR spectrum (499.87 MHz, 305.1 K, C_6D_6) of 5-bromo-6-(methylphenylsilyl)acenaphthene **5** (#C_6D_6).

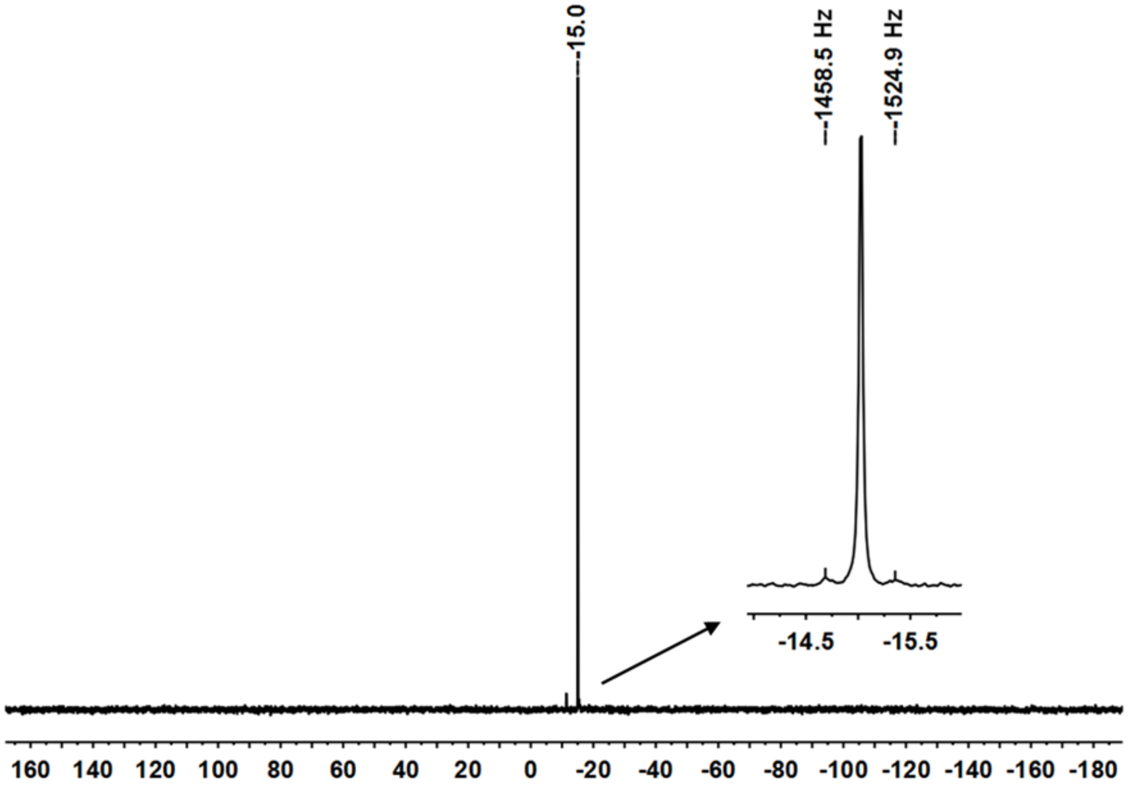

Figure S 8 – $^{29}Si\{^{1}H\}$ INEPT NMR spectrum (99.31 MHz, 305.0 K, C_6D_6) of 5-bromo-6-(methylphenylsilyl)acenaphthene **5**.

1.2.2 Synthesis and characterization of chalcogenyl-substituted silanes

5-Methylphenylsilyl-6-phenoxylacenaphthene 3a

The title compound **3a** was synthesized according to general procedure **B** using 1.0 equiv. (2.00 mmol, 0.65 g) 5-Bromo-6-phenoxylacenaphene **1**, 1.0 equiv. (2.00 mmol, 1.26 mL) *n*-butyl lithium and 1.2 equiv. (2.40 mmol, 376 mg) chloro(methyl)phenylsilane. In addition, 1.1 equiv. (2.20 mmol, 256 mg) TMEDA was added to the starting material before adding *n*-butyl lithium. The raw product was purified by column chromatography using *n*-pentane as eluent (R_F = 0.20). The product **3a** was obtained as a colorless oil. Yield 0.40 mg (1.11 mmol; 55 %).

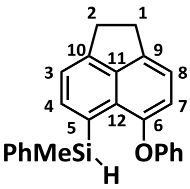

^{1}H NMR (499.9 MHz, 298.1 K, C_6D_6) δ = 0.84 (d, $^3J_{H,H}$ = 3.7 Hz, 3 H, $SiCH_3$), 2.98-3.02 (m, 2 H, 1-H), 3.05-3.08 (m, 2 H, 2-H), 5.63 (q, $^3J_{H,H}$ = 3.7 Hz, $^1J_{H,Si}$ = 196.2 Hz, 1 H, Si-H), 6.75 (d, $^3J_{H,H}$ = 6.6 Hz, 1 H, 7-H), 6.81-6.83 (m, 2 H, O-*o*-Ph), 6.87-6.92 (m, 2 H, 8-H, O-*p*-Ph), 7.04-7.09 (m, 2 H, O-*m*-Ph), 7.13-7.19 (m, 4 H, Si-*m/p*-Ph, 3-H), 7.58-7.61 (m, 2 H, Si-*o*-Ph), 7.99 (d, $^3J_{H,H}$ = 6.9 Hz, 1 H, 4-H) ppm. **^{13}C{^{1}H} NMR** (125.7 MHz, 297.8 K, C_6D_6) δ = -2.9 ($SiCH_3$), 29.7 (CH_2, C-2), 31.0 (CH_2, C-1), 113.8 (C-7), 119.4 (C-8), 120.2 (C-3), 120.7 (CH, OPh), 123.8 (CH, OPh), 125.7 (C, C-5), 127.9 (CH), 128.9 (CH), 129.2 (C), 129.9 (CH), 135.0 (CH, SiPh), 138.2 (C, Si-*ipso*-Ph), 139.3 (CH, C-4), 140.5 (C), 141.4 (C), 148.6 (C), 153.3 (C, C-6), 156.8 (C, O- *ipso*-Ph). **^{29}Si{^{1}H} INEPT NMR** (99.3 MHz, 297.9 K, C_6D_6) δ = -14.7. **IR** (ATR, fest): $\tilde{\nu}$(Si-H) [cm^{-1}] = 2096, 2136. **GC-MS** t_R = 32.8 min, m/z (M^+) = 366. **HR/MS** calculated: m/z = 366.1440; found (EI): m/z = 366.1442.

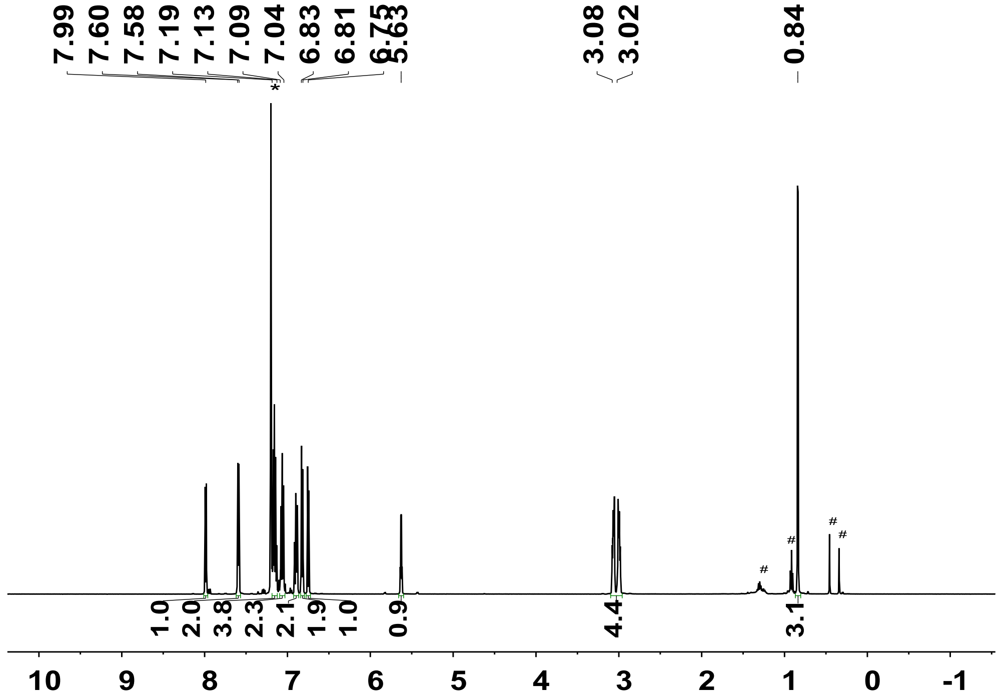

Figure S 9 – ^{1}H NMR spectrum (499.9 MHz, 298.1 K, C_6D_6) of 5-(methylphenylsilyl)-6-phenoxyacenaphthene **3a** (*C_6D_5H, # impurities).

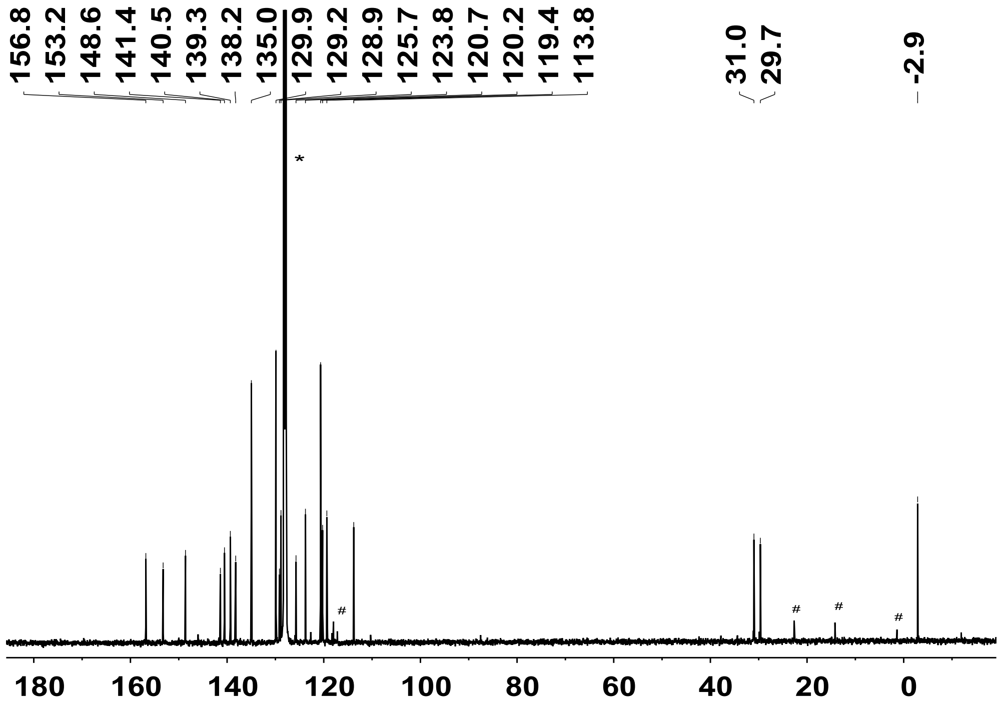

Figure S 10 – $^{13}C\{^{1}H\}$ NMR spectrum (125.7 MHz, 297.8 K, C_6D_6) of 5-(methylphenylsilyl)-6-phenoxyacenaphthene **3a** (*C_6D_6, # impurities).

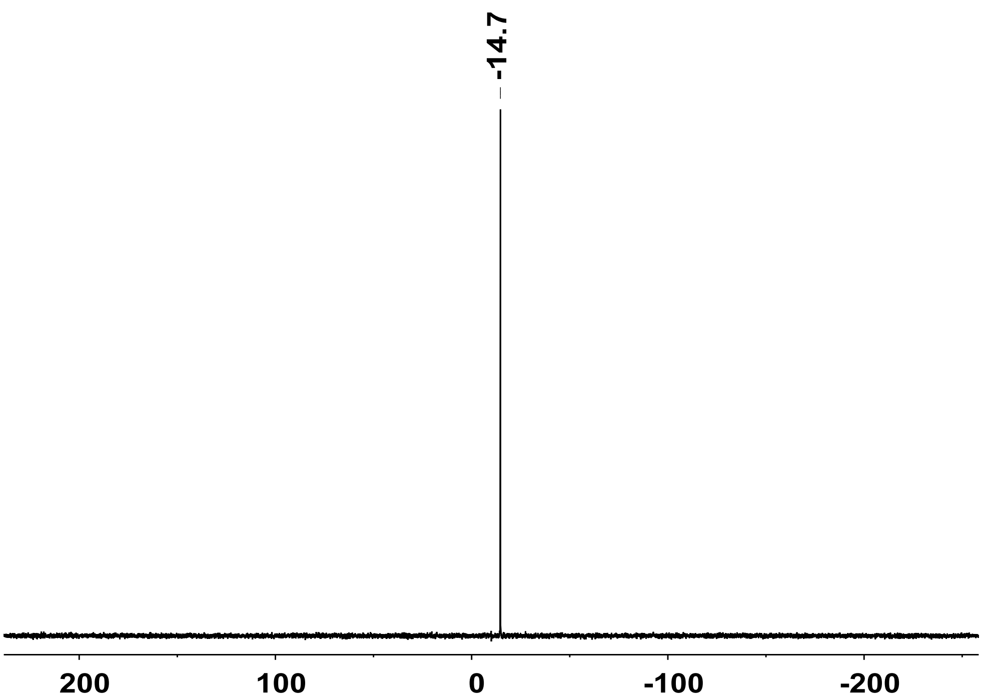

Figure S 11 – $^{29}Si\{^{1}H\}$ INEPT NMR spectrum (99.3 MHz, 297.9 K, C_6D_6) of 5-(methylphenylsilyl)-6-phenoxyacenaphthene **3a**.

5-Methyl-*tert*-butylsilyl-6-phenoxylacenaphthene 3b

The title compound **3b** was synthesized according to general procedure **B** using 1.0 equiv. (1.00 mmol, 325 mg) 5-bromo-6-phenoxylacenaphene **1**, 1.1 equiv. (1.10 mmol, 0.70 mL) *n*-butyl lithium and 1.5 equiv. (1.50 mmol, 205 mg) chloro(*tert*-butyl)methylsilane. The chlorosilane was added at -50 °C and the reaction mixture was stirred for further 60 min at this temperature. The raw product was purified by column chromatography using *n*-pentane as eluent (R_F = 0.26). The product **3b** was obtained as a colorless oil. Yield 134 mg (0.39 mmol; 39 %).

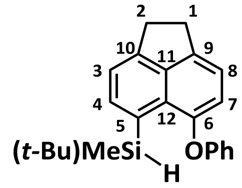

^{1}H NMR (499.9 MHz, 298.1 K, C_6D_6) δ = 0.56 (d, $^{3}J_{H,H}$ = 3.8 Hz, 3 H, SiMe), 1.19 (s, 9 H, Si(*t*-Bu)), 2.98-3.02 (m, 2 H, 1-H), 3.05-3.09 (m, 2 H, 2-H), 4.95 (q, $^{3}J_{H,H}$ = 3.8 Hz, $^{1}J_{H,Si}$ = 192.9 Hz, 1 H, Si-H), 6.85 (d, $^{3}J_{H,H}$ = 7.5 Hz, 1 H, 7-H), 6.91-6.96 (m, 2 H, 3-H, p-Ph), 7.07-7.10 (m, 2 H, o-Ph), 7.12-7.16 (m, 2 H, m-Ph), 7.21-7.22 (m, 1 H, 3-H), 7.98 (d, $^{3}J_{H,H}$ = 6.9 Hz, 1 H, 4-H). **$^{13}C\{^{1}H\}$ NMR** (125.7 MHz, 297.8 K, C_6D_6) δ = -4.8 (SiCH3), 18.1 (C, t-Bu), 28.5 (CH3, t-Bu), 29.6 (CH2, C-2), 30.9 (CH2, C-1), 114.9 (CH, C-7), 119.3 (CH, C-8), 119.9 (CH, C-3), 120.8 (CH, Ph), 123.7 (CH, Ph), 125.9 (C, C-5), 129.5 (C), 130.1 (CH, Ph), 139.0 (CH, C-4), 141.0 (C), 141.4 (C), 148.2 (C), 153.2 (C, C-6), 157.6 (C, i-Ph). **$^{29}Si\{^{1}H\}$ NMR** (99.3 MHz, 297.9 K,

C_6D_6) δ = 3.1. **GC-MS** t_R = 26.5 min, m/z (M^+) = 346. **HR/MS** calculated: m/z = 346.1747; found (EI): m/z = 346.1757. **IR** (ATR, solid): $\tilde{\nu}$(Si-H) [cm^{-1}] = 2090, 2151. **EA** $C_{23}H_{26}SiO$, calculated: C 79.72, H 7.56, solid: C 80.00, H 7.66.

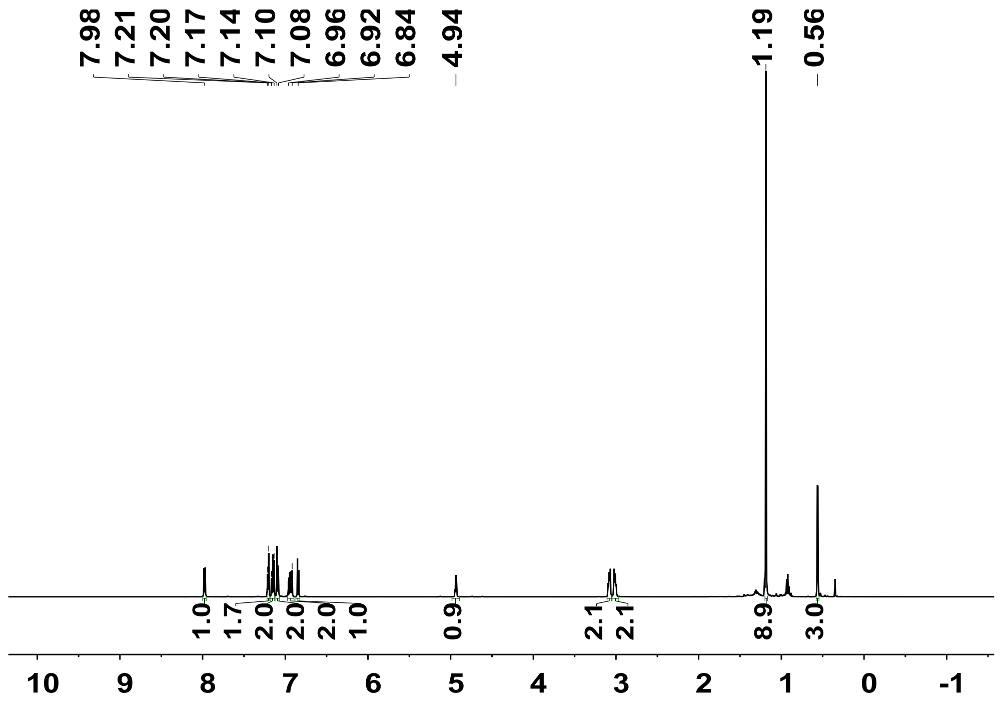

Figure S 12 – ^{1}H NMR spectrum (499.9 MHz, 298.1 K, C_6D_6) of 5-(*tert*-butylmethylsilyl)-6-phenoxyacenaphthene **3b**.

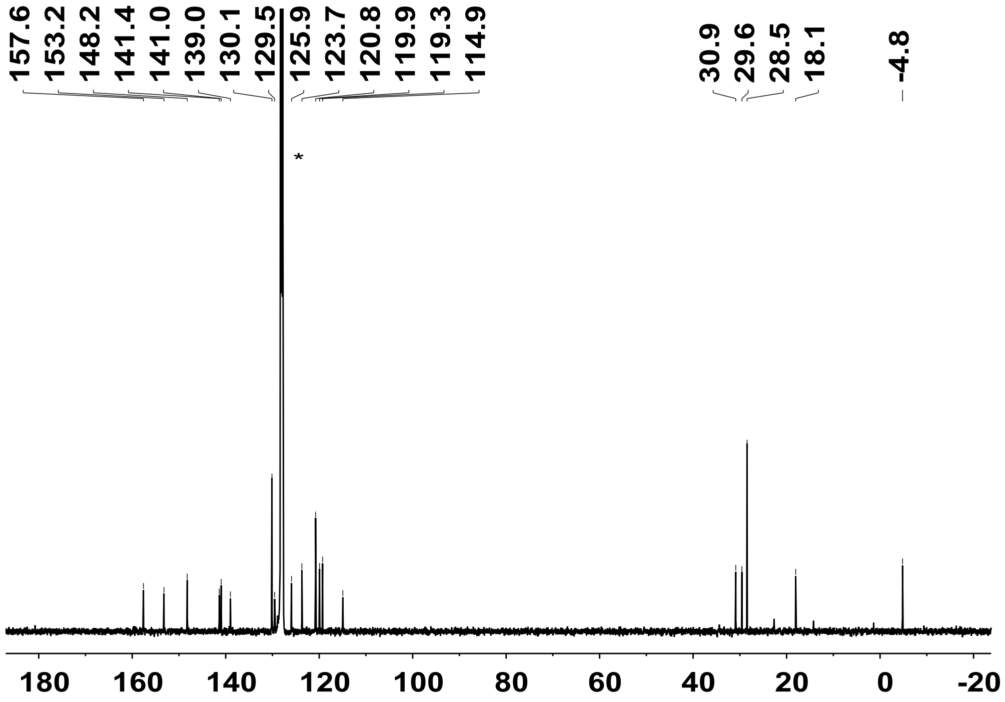

Figure S 13 – ^{13}C{^{1}H} NMR spectrum (125.7 MHz, 297.8 K, C_6D_6) of 5-(*tert*-butylmethylsilyl)-6-phenoxyacenaphthene **3b** (*C_6D_6).

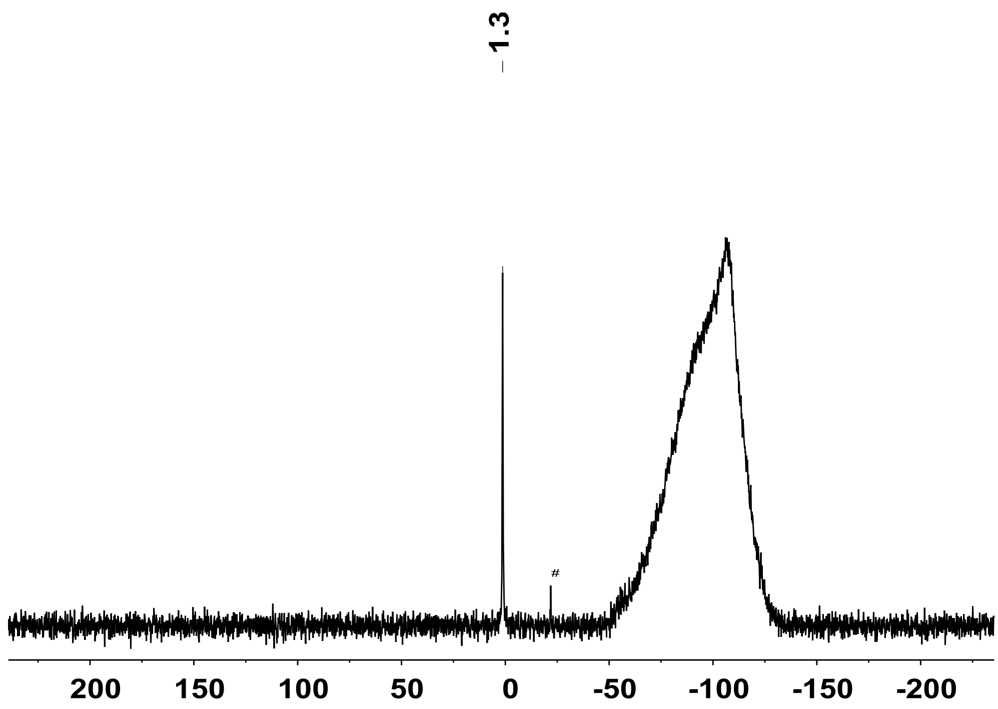

Figure S 14 – $^{29}Si\{^1H\}$ NMR spectrum (99.3 MHz, 297.9 K, C_6D_6) of 5-(*tert*-butylmethylsilyl)-6-phenoxyacenaphthene **3b** (# silicon grease).

1-Phenylsulfanyl-8-(phenylmethylsilyl)naphthalene 9

The title compound was synthesized according to general procedure **C** using 1.0 equiv. (3.06 mmol, 1.00 g) 8-phenylmethylsilyl-1-bromonaphthalene, 1.0 equiv. (3.06 mmol, 1.91 mL) *n*-butyl lithium and 1.0 equiv. (3.06 mmol, 0.67 g) diphenyl disulfide. The raw-product was purified by column chromatography using petroleum ether/ethyl acetate (99:1) as eluent (R_F = 0.26). Silane **9** was obtained as a colorless solid after crystallization from *n*-pentane at -18 °C. Yield 0.96 g (2.70 mmol; 88 %).

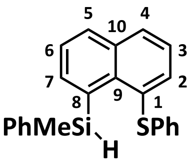

^{1}H NMR (500.13 MHz, 298.4 K, C_6D_6) δ = 0.85 (d, $^3J_{H,H}$ = 3.5 Hz, 3 H, $SiCH_3$), 5.87 (q, $^3J_{H,H}$ = 3.5 Hz, $^1J_{H,Si}$ = 203.0 Hz, 1 H, SiH), 6.77-6.84 (m, 5 H, SPh), 7.07 (t, $^3J_{H,H}$ = 7.6 Hz, 1 H, 3-H), 7.11-7.15 (m, 3 H, Si-*m*-Ph, Si-*p*-Ph), 7.21-7.24 (m, 1 H, 6-H), 7.53-7.55 (m, 2 H, Si-*o*-Ph), 7.60 (dd, $^3J_{H,H}$ = 8.1 Hz, $^4J_{H,H}$ = 1.2 Hz, 1 H, 4-H), 7.66 (dd, $^3J_{H,H}$ = 8.1 Hz, $^4J_{H,H}$ = 1.1 Hz, 1 H, 5-H), 7.75 (dd, $^3J_{H,H}$ = 7.2 Hz, $^4J_{H,H}$ = 1.3 Hz, 1 H, 2-H), 8.08 (dd, $^3J_{H,H}$ = 6.9 Hz, $^4J_{H,H}$ = 1.0 Hz, 1 H, 7-H). **$^{13}C\{^1H\}$ NMR** (125.77 MHz, 298.3 K, C_6D_6) δ = -0.5 ($SiCH_3$), 125.4 (CH), 126.0 (CH), 126.2 (CH), 127.2 (C), 128.5 (CH), 128.7 (CH), 129.0 (CH), 131.5 (CH), 131.9 (C), 132.1 (CH), 134.2 (CH), 134.7 (CH), 135.6 (C), 137.6 (C), 139.6 (C), 140.6 (CH), 140.6 (C), 140,9 (CH). **$^{29}Si\{^1H\}$ NMR** (99.31 MHz, 673.2 K, C_6D_6) δ = -16.9. **IR** (ATR, solid):

$\tilde{\nu}$(Si-H) [cm^{-1}] = 2144. **HR/MS** calculated: m/z = 356.1055; found (ESI): m/z = 355.0595. **EA** $C_{23}H_{20}SSi$, calculated.: C 77.48, H 5.65; S 8.99, found: C 76.41, H 5.98, S 8.60.

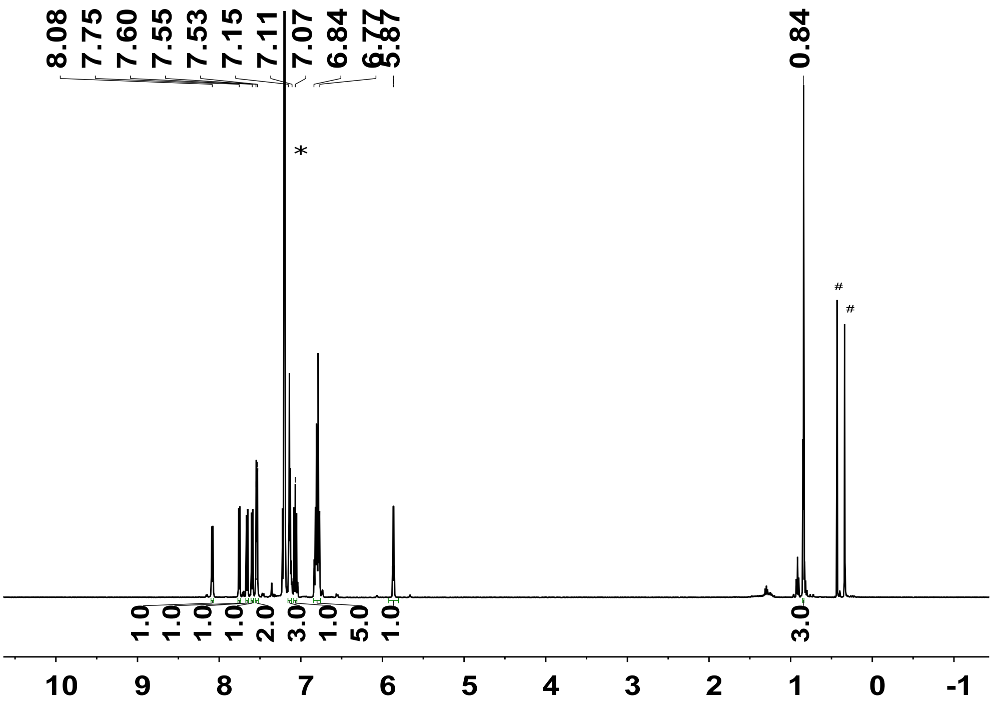

Figure S 15 – ^{1}H NMR spectrum (499.87 MHz, 305.1 K, C_6D_6, *C_6D_5H) of 1-phenylsulfanyl-8-phenylmethylsilyl-naphthalene **9** (*C_6D_5H, # silicon grease and water).

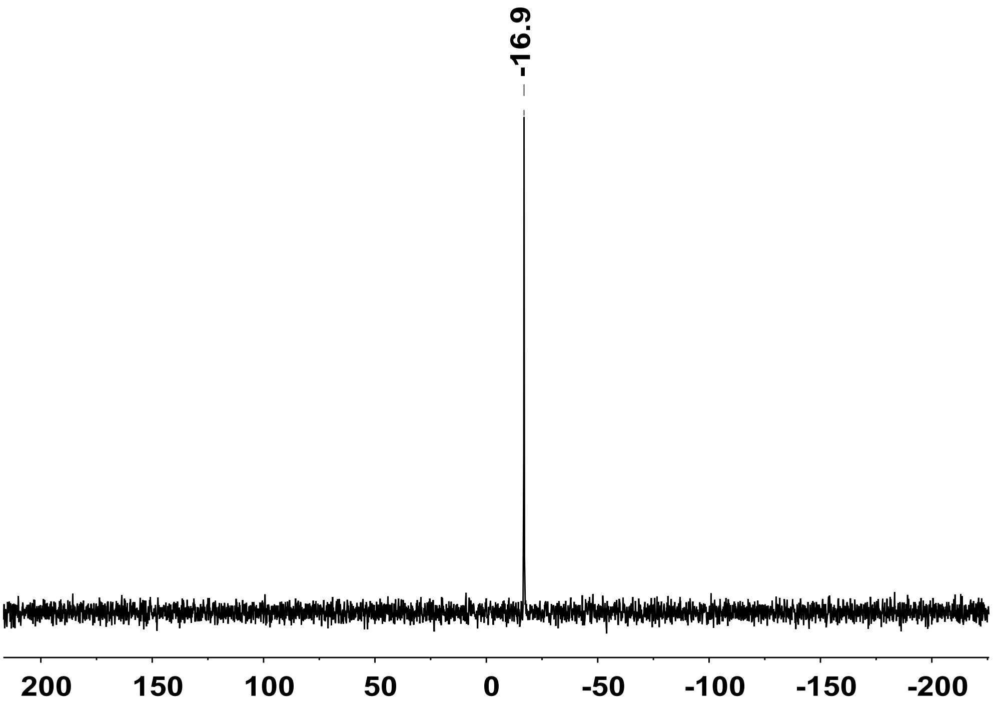

Figure S 16 – $^{29}Si\{^{1}H\}$ INEPT NMR spectrum (99.3 MHz, 297.9 K, C_6D_6, D_3 = D_4 = 0.0013) of 1-phenylsulfanyl-8-phenylmethylsilylnaphthene **9**.

5-Phenylsulfanyl-6-phenylmethylsilylacenaphthene 4a

The title compound was synthesized according to general procedure **A** using 1.0 equiv. (4.31 mmol, 1.47 g) 5-bromo-6-phenylsulfanylacenaphene **2**, 1.0 equiv. (4.31 mmol, 2.69 mL) *n*-butyl lithium and 1.0 equiv. (4.31 mmol, 0.65 mL) chloro(methyl)phenylsilane. The product was purified by crystallization from *n*-pentane and obtained as a colorless solid. Yield 1.07 g (2.80 mmol; 65 %).

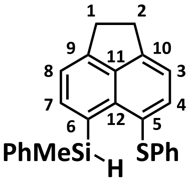

^{1}H NMR (499.87 MHz, 305.1 K, C_6D_6) δ = 0.88 (d, ^{3}J = 3.6 Hz, 3 H, $SiCH_3$), 2.91-3.95 (m, 2 H, CH_2), 2.96-3.00 (m, 2H, CH_2), 5.89 (q, ^{3}J = 3.4 Hz, $^{1}J_{H,Si}$ = 202.8 Hz, 1 H, SiH), 6.80-6.83 (m, 1 H, CH, S-*p*-Ph), 6.87-6.92 (m, 4 H, CH, S-*m/o*-Ph), 6.96 (d, ^{3}J = 7.3 Hz, 1 H, CH, 3-H), 7.10 (d, ^{3}J = 7.0 Hz, 1 H, CH, 8-H), 7.14-7.17 (m, 3 H, CH, Si-*m/p*-Ph), 7.60-7.64 (m, 2 H, CH, Si-*o*-Ph), 7.79 (d, ^{3}J = 7.1 Hz, 1 H, CH, 4-H), 8.05 (d, ^{3}J = 7.0 Hz, 1 H, CH, 7-H). **^{13}C{^{1}H} NMR** (125.71 MHz, 305.0 K, C_6D_6) δ = -1.0 ($SiCH_3$), 30.1 (CH_2, C-2), 30.3 (CH_2, C-1), 120.2 (CH, C-8), 120.7 (CH, C-3), 125.1 (CH, S-*p*-Ph), 126.7 (C, C-5), 126.9 (CH, S-*m*-Ph), 128.1 (CH, Si-*m*-Ph), 128.8 (CH, S-*p*-Ph), 128.9 (C, C-6), 129.0 (CH, S-*o*-Ph), 134.9 (CH, Si-*o*-Ph), 139.3 (C, Si-*ipso*-Ph), 139.5 (C, C-12), 139.7 (CH, C-4), 140.9 (C, C-11), 141.5 (C, S-*ipso*-Ph), 141.9 (CH, C-7), 149.5 (C, C-10), 149.7 (C, C-9). **^{29}Si{^{1}H} NMR** (99.31 MHz, 305.0 K, C_6D_6) δ = -17.2. **GC/MS** t_R = 30.8 min, m/z (%) = 51 (16), 77 (38), 152 (71), 227 (64), 289 (100), 305 (24), 367 (41), 382 (23) [M^+]. **HR/MS** calculated: m/z = 382.1206; found (EI): m/z = 382.1196. **IR** (ATR, solid): $\tilde{\nu}$(Si-H) [cm^{-1}] = 2091. **EA** $C_{25}H_{22}SSi$, calculated: C 78.48, H 5.80, S 8.38; found: C 78.57, H 6.63, S 8.08.

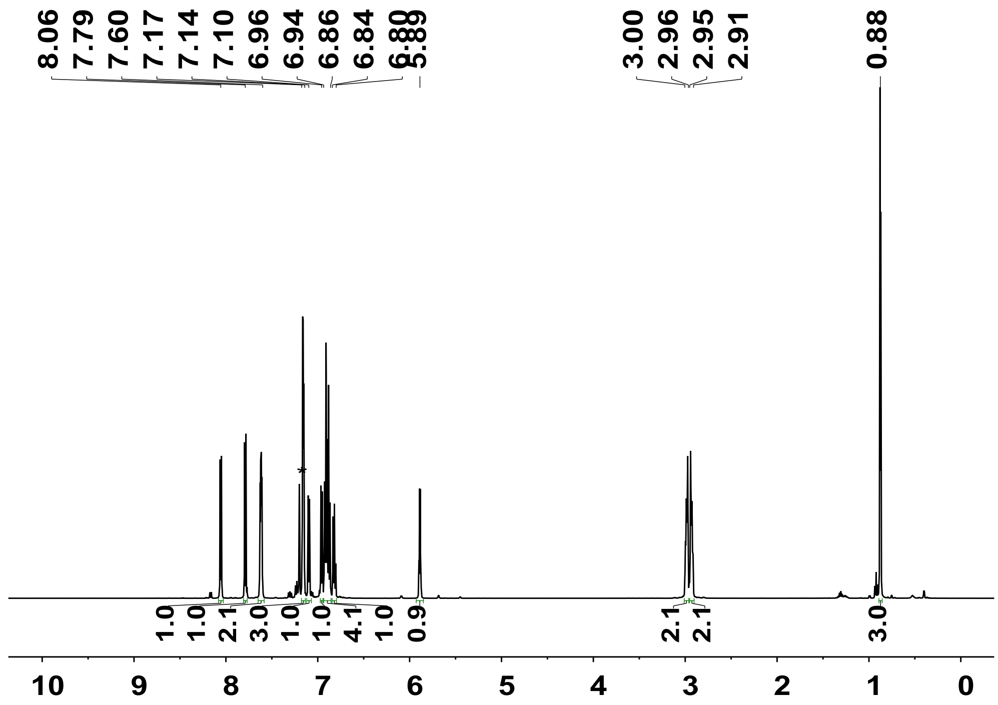

Figure S 17 – ^{1}H NMR spectrum (499.87 MHz, 305.1 K, C_6D_6) of 5-phenylsulfanyl-6-phenylmethylsilylacenaphthene **4a** (*C_6D_5H).

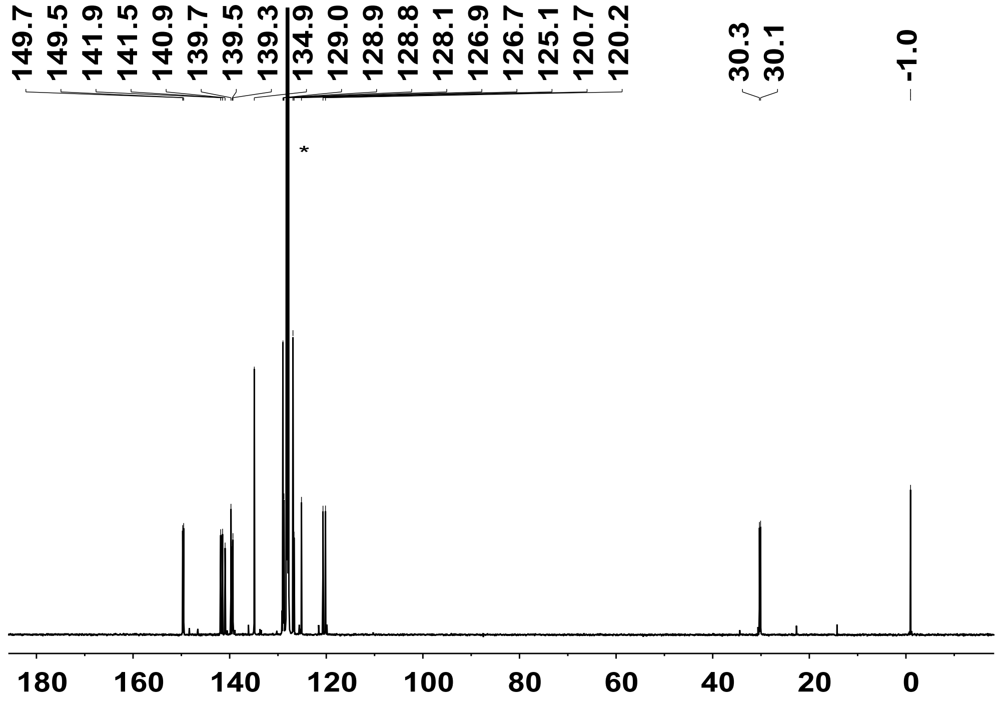

Figure S 18 – ^{13}C{^{1}H} NMR spectrum (125.71 MHz, 305.0 K, C_6D_6) of 5-phenylsulfanyl-6-phenylmethylsilylacenaphthene **4a** (*C_6D_6).

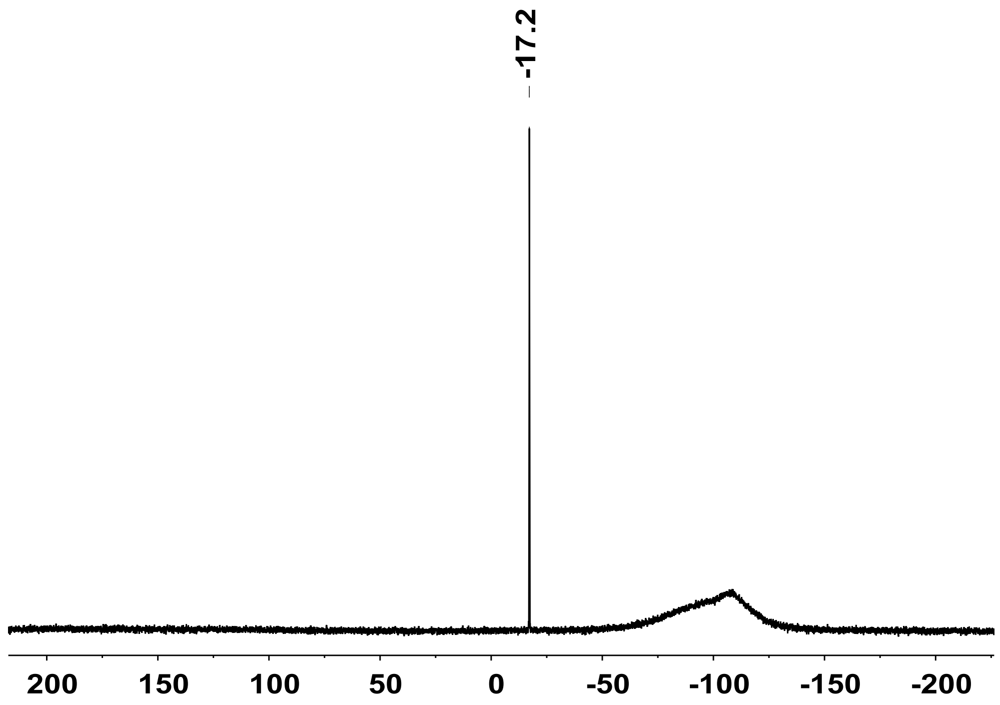

Figure S 19 – $^{29}Si\{^1H\}$ NMR spectrum (99.31 MHz, 305.0 K, C_6D_6) of 5-phenylsulfanyl-6-phenylmethylsilylacenaphthene **4a**.

5-Phenylsulfanyl-6-methyl-*tert*-butylsilylacenaphthene 4b

The title compound was synthesized according to general procedure **B** using 1.0 equiv. (990.43 µmol, 338 mg) 5-bromo-6-phenylsulfanylacenaphene **2**, 1.0 equiv. (990.43 µmol, 0.63 mL) *n*-butyl lithium and 1.0 equiv. (990.43 µmol, 94 mg) chloro(*tert*-butyl)methyl silane. The product **4b** was purified by crystallization from *n*-pentane and obtained as an orange solid. Yield 107 mg (297.13 µmol, 30 %).

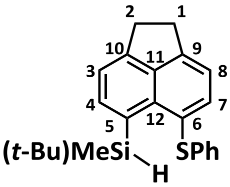

^{1}H NMR (500.13 MHz, 298.9 K, C_6D_6) δ = 0.54 (d, $^3J_{H,H}$ = 3.4 Hz, 3 H, $SiCH_3$), 1.19 (s, 9 H, Si(*t*-Bu)), 2.91-2.95 (m, 2 H, CH_2), 2.98-3.02 (m, 2 H, CH_2), 5.41 (q, $^3J_{H,H}$ = 3.3 Hz, $^1J_{H,Si}$ = 199.8 Hz, 1 H, SiH), 6.82-6.85 (m, 1 H, *p*-Ph), 6.92-6.95 (m, 2 H, *m*-Ph), 6.98 (d, $^3J_{H,H}$ = 7.1 Hz, 1 H, 3-H), 7.05-7.06 (m, 2 H, *o*-Ph), 7.16 (d, $^3J_{H,H}$ = 7.0 Hz, 1 H, 8-H), 7.88 (d, $^3J_{H,H}$ = 7.1 Hz, 1 H, 4-H), 8.01 (d, $^3J_{H,H}$ = 7.0 Hz, 1 H, 7-H). **$^{13}C\{^1H\}$ NMR** (125.71 MHz, 305.0 K, C_6D_6) δ = -3.2 ($SiCH_3$), 18.5 (C, *t*Bu), 30.0 (CH_2, C-2), 30.3 (CH_2, C-1), 119.8 (CH, C-8), 120.5 (CH, C-3), 125.2 (CH, *p*-Ph), 126.9 (C, *ipso*-Ph), 127.2 (CH, *o*-Ph), 129.1 (CH, *m*-Ph), 129.6 (C, C-6), 139.4 (C, C-12), 139.8 (CH, C-4), 140.2 (CH, C-7), 141.0 (C, C-5), 141.3 (C, C-11), 149.2 (C, C-9), 149.5 (C, C-10). **$^{29}Si\{^1H\}$ NMR** (99.36 MHz, 299.1 K, C_6D_6) δ = -3.3. **HR/MS** calculated: m/z = 362.1519; found (EI): m/z = 362.1508. **IR** (ATR, solid): $\tilde{\nu}$(Si-H) [cm^{-1}] = 2175.

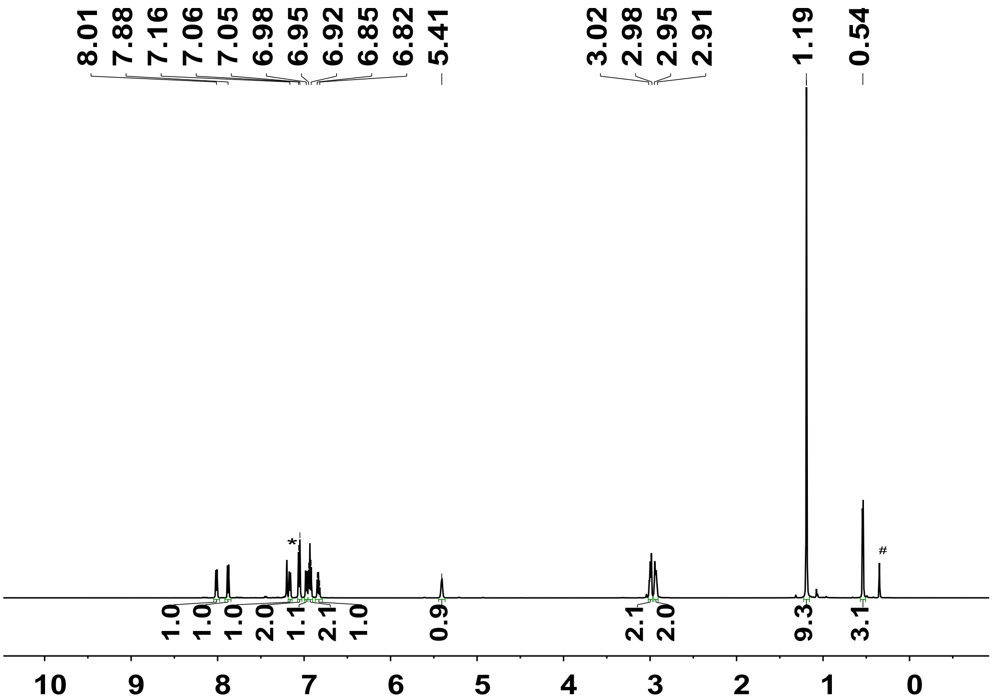

Figure S 20 – ^{1}H NMR spectrum (500.13 MHz, 298.9 K, C_6D_6) of 5-phenylsulfanyl-6-(*tert*-butyl)methylsilylacenaphthene **4b** (*C_6D_5H, # silicon grease).

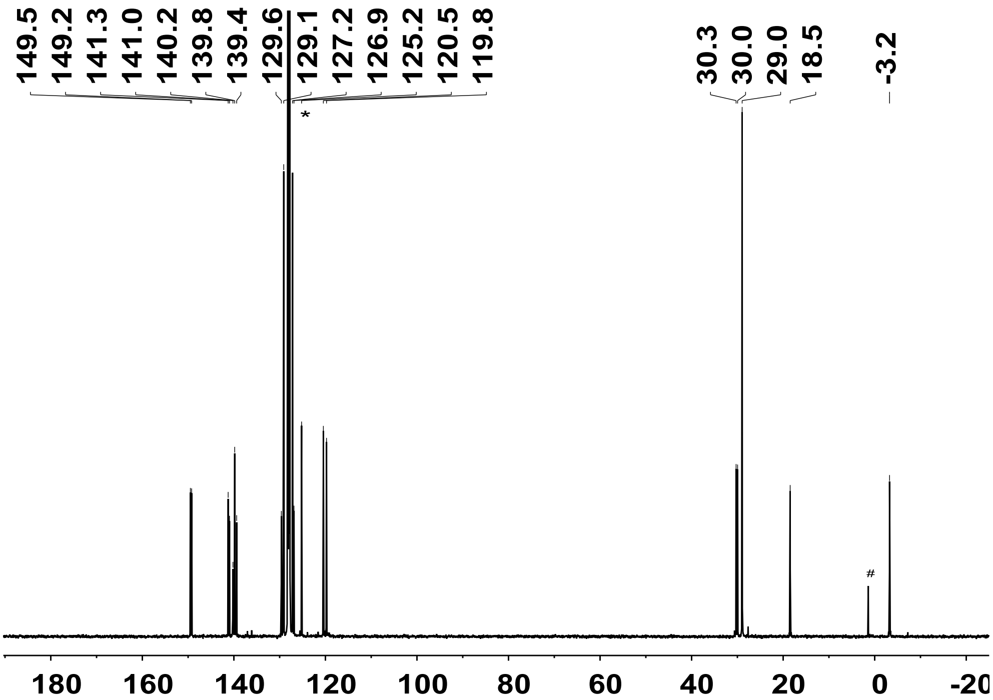

Figure S 21 – ^{13}C{^{1}H} NMR spectrum (125.71 MHz, 305.0 K, C_6D_6) of 5-phenylsulfanyl-6-(*tert*-butyl)methylsilylacenaphthene **4b** (*C_6D_6, # silicon grease).

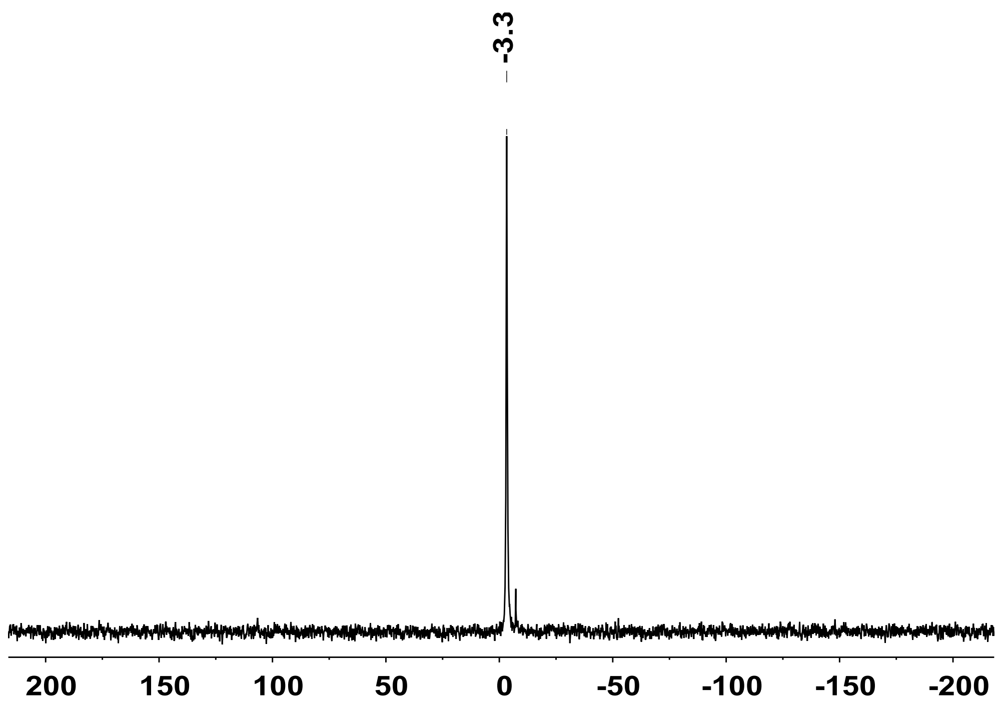

Figure S 22 – $^{29}Si\{^{1}H\}$ INEPT NMR spectrum (99.36 MHz, 299.1 K, C_6D_6) of 5-phenylsulfanyl-6-(*tert*-butyl)methylsilylacenaphthene **4b**.

1-Phenylselanyl-8-phenylmethylsilylnaphthalene 10a

The title compound was synthesized according to general procedure **C** in diethylether using 1.00 g (3.06 mmol) 8-phenylmethylsilyl-1-bromonaphthalene **8a**, 1.91 mL (3.06 mmol) *n*-butyl lithium and 0.95 g (3.06 mmol) diphenyl diselenide. The raw-product was purified by column chromatography using *n*-hexane as eluent (R_F = 0.11). Silane **10a** was obtained as a colorless oil. Yield 1.10 g (2.76 mmol; 91 %).

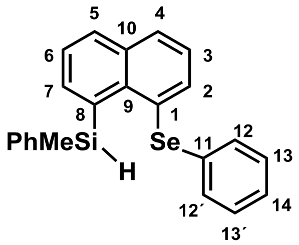

^{1}H NMR (499.87 MHz, 305.0 K, C_6D_6): δ = 0.87 (d, 3H, $^{3}J_{H,H}$ = 3.6 Hz), 5.98 (q, 1H, $^{3}J_{H,H}$ = 3.6 Hz, $^{1}J_{H,Si}$ = 202.4 Hz), 6.77 - 6.83 (m, 3H), 6.91 - 6.93 (m, 2H), 7.02-7.05 (m, 1H), 7.12 - 7.15 (m, 3H), 7.19 - 7.22 (m, 1H), 7.54 - 7.56 (m, 2H), 7.60 (dd, 1H, $^{3}J_{H,H}$ = 8.1 Hz, $^{4}J_{H,H}$ = 1.5 Hz), 7.65 (dd, 1H, $^{3}J_{H,H}$ = 8.1 Hz, $^{4}J_{H,H}$ = 1.4 Hz), 7.95 (dd, 1H, $^{3}J_{H,H}$ = 7.1 Hz, $^{4}J_{H,H}$ = 1.5 Hz), 8.09 (dd, 1H, $^{3}J_{H,H}$ = 6.9 Hz, $^{4}J_{H,H}$ = 1.4 Hz). **$^{13}C\{^{1}H\}$ NMR** (125.71 MHz, 305.0 K, C_6D_6): δ = -0.0 (CH_3), 125.7 (CH), 126.21 (CH), 126.24 (CH), 128.8 (CH), 129.3 (CH), 129.8 (CH), 130.4 (C), 131.5 (CH), 132.0 (CH), 134.7 (CH), 135.6 (C), 135.7 (C), 136.4 (C), 139.4 (CH), 139.6 (C), 140.9 (CH). Two C missing, due to overlap with the solvent signal. **$^{29}Si\{^{1}H\}$ NMR** (99.31 MHz, 305.0 K, C_6D_6): δ = -18.0. **^{29}Si INEPT NMR** (99.31 MHz, 305.0 K, C_6D_6, D3 = D4 = 0.0013): δ

= -18.0 (dm, $^{1}J_{Si,H}$ = 202.1 Hz). **^{77}Se{^{1}H} NMR** (95.36 MHz, 305.0 K, C_6D_6): δ = 393.0. **^{77}Se NMR** (95.36 MHz, 305.0 K, C_6D_6): δ = 393.0 (d, $^{TS}J_{Se,H}$ = 13.9 Hz). **GC/MS** t_R: 31.3 min, m/z (%) 403 [M+] (16), 389 (31), 387 (18), 326 (23), 311 (63), 309 (34), 249 (36), 231 (32), 215 (14), 202 (72), 179 (8), 169 (19), 167 (30), 141 (46), 127 (44), 115 (24), 105 (27), 77 (100), 51 (60). **IR** (ATR, 298 K, neat): ν = 2083, 2141 cm^{-1}. **HR/MS** (LIFDI) $C_{23}H_{20}SeSi$, calc.: 404.0494, found: 404.0482. **EA** $C_{23}H_{20}SeSi$, calc.: C 68.47, H 5.00, found: C 68.39, H 5.65.

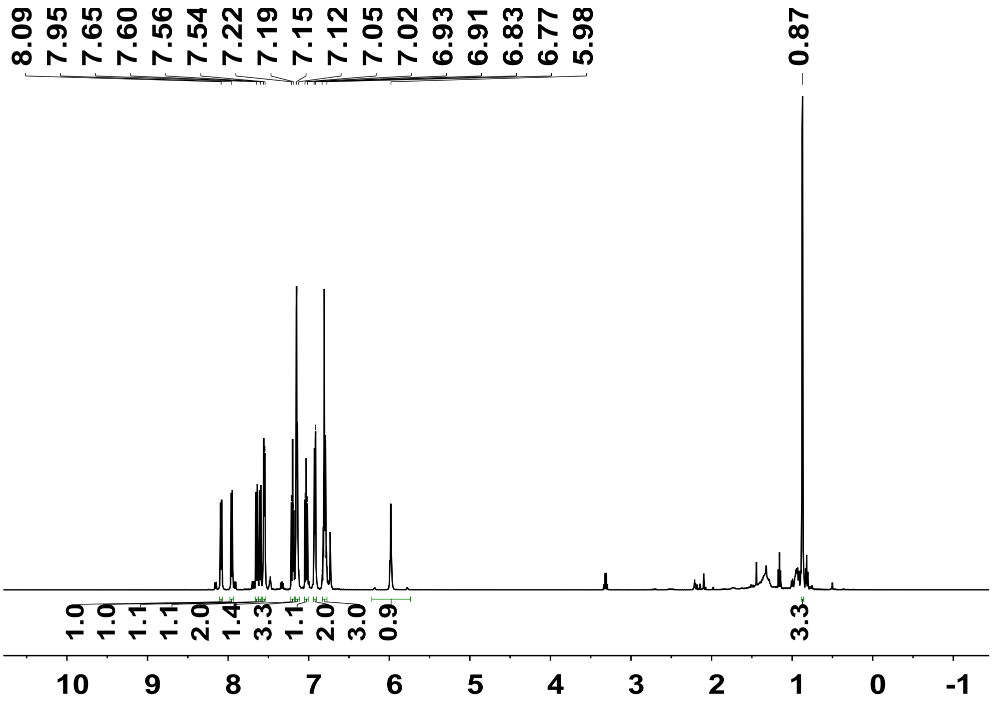

Figure S 23 – ^{1}H NMR spectrum (499.87 MHz, 305.0 K, C_6D_6) of 1-phenylselanyl-8-phenylmethylsilylnaphthalene **10a**.

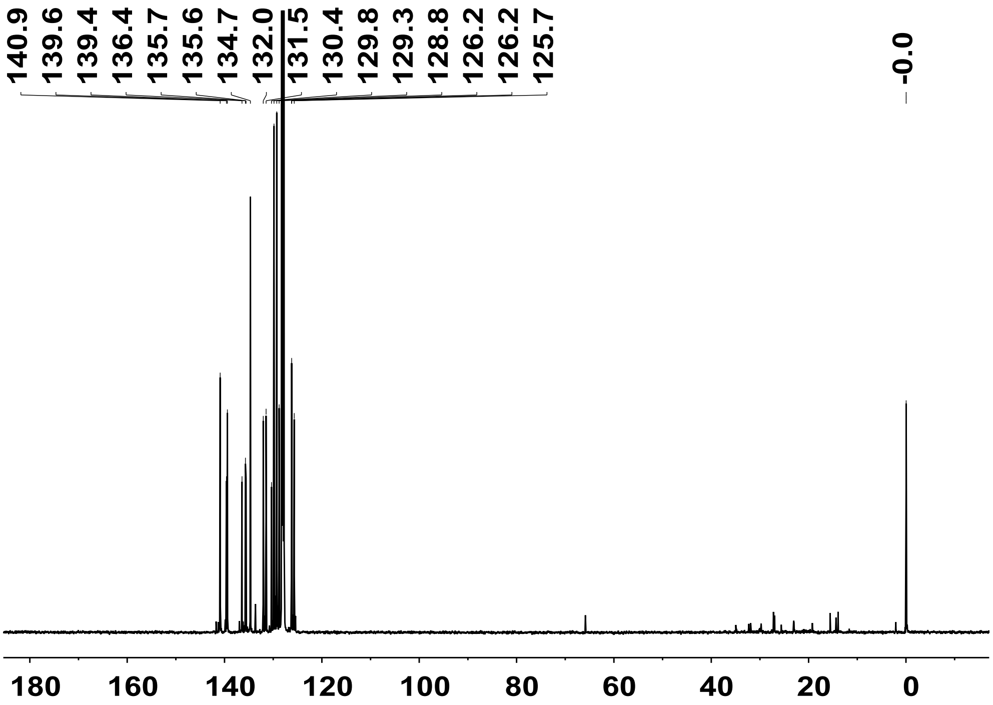

Figure S 24 – $^{13}C\{^1H\}$ NMR spectrum (125.71 MHz, 305.0 K, C_6D_6) of 1-phenylselanyl-8-phenylmethylsilylnaphthalene **10a**.

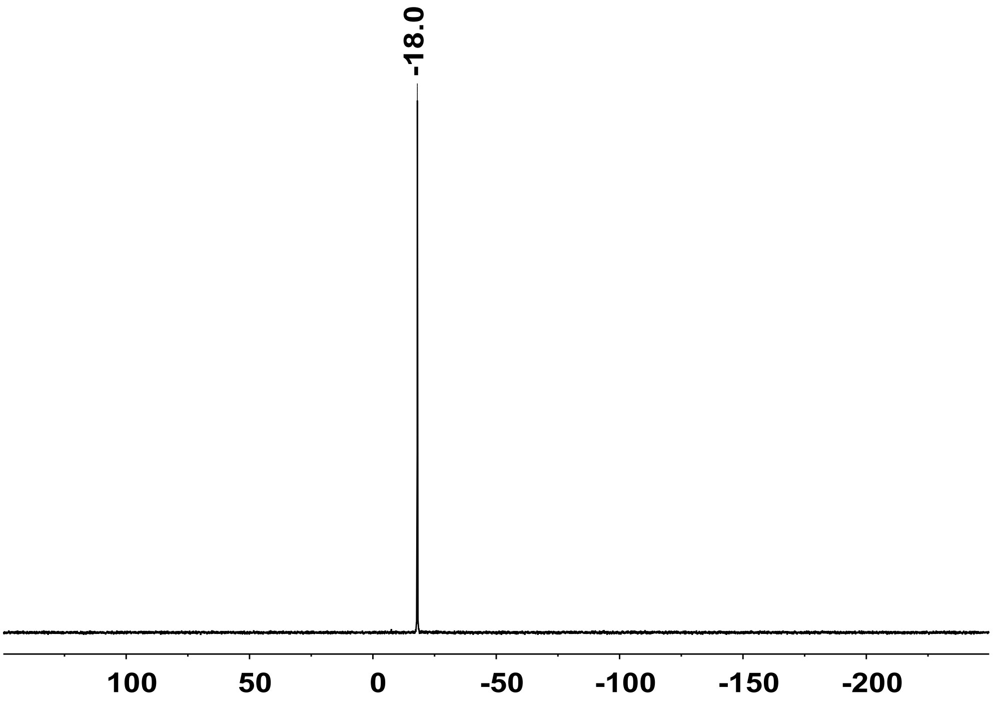

Figure S 25 – $^{29}Si\{^1H\}$ INEPT NMR spectrum (99.31 MHz, 305.0 K, C_6D_6) of 1-phenylselanyl-8-phenylmethylsilylnaphthalene **10a**.

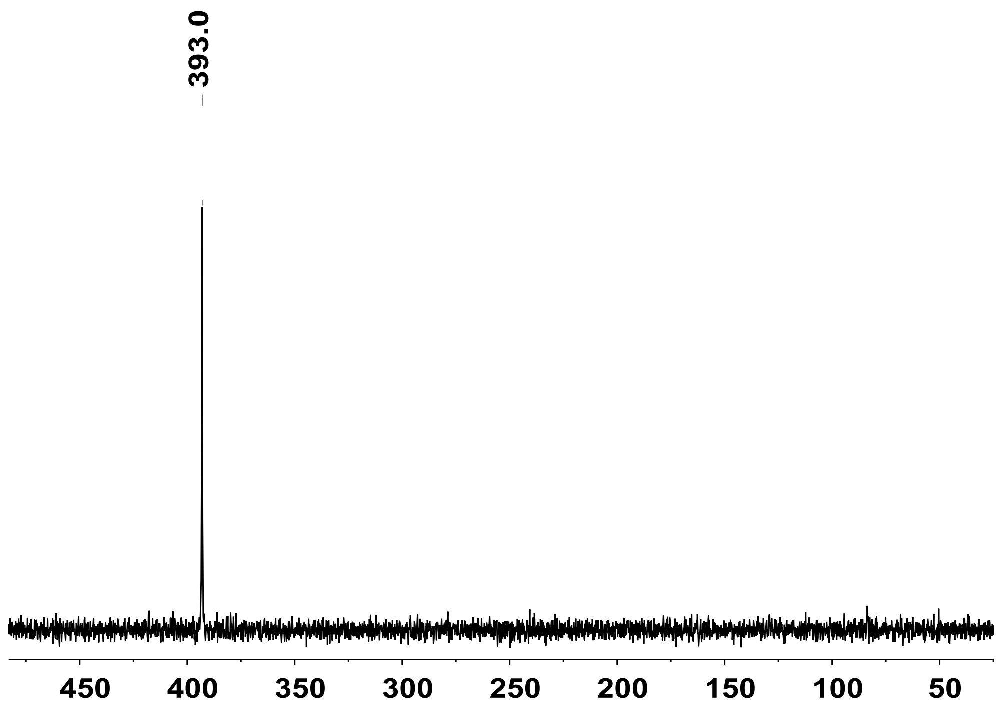

Figure S 26 – $^{77}Se\{^1H\}$ NMR spectrum (95.36 MHz, 305.0 K, C_6D_6) of 1-phenylselanyl-8-phenylmethylsilylnaphthalene **10a**.

1-Phenylselanyl-8-*tert*-buthylmethylsilylnaphthalene 10b

The title compound was synthesized according to general procedure **C** in diethyl ether using 500 mg (1.63 mmol) 8-*tert*-butylmethylsilyl-1-bromonaphthalene **8b**, 1.02 mL (1.63 mmol) *n*-butyl lithium and 508 mg (1.63 mmol) diphenyl diselenid. The raw-product was purified by column chromatography using *n*-hexane as eluent (R_F = 0.28). Silane **10b** was obtained as a colorless oil. Yield 0.40 g (0.75 mmol; 46 %).

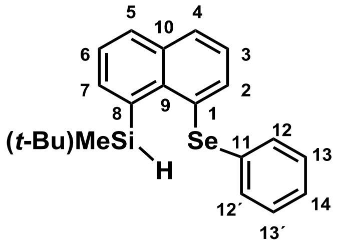

^{1}H NMR (499.87 MHz, 305.0 K, C_6D_6): δ = 0.61 (d, 3H, $^3J_{H,H}$ = 3.6 Hz, Si-CH_3), 1.09 (s, 9H, Si-$C(CH_3)_3$), 5.45 (q, 1H, $^1J_{H,Si}$ = 196.3 Hz, 3J = 3.6 Hz, $^{TS}J_{H,Se}$ = 28.1 Hz, Si-H), 6.85 - 6.88 (m, 3H, 12-H, 14-H), 7.00 (dd, 1H, $^3J_{H,H}$ = 7.3 Hz, $^3J_{H,H}$ = 8.0 Hz, 3-H), 7.15 - 7.17 (m, 2H, 13-H), 7.25 - 7.28 (m, 1H, 6-H), 7.53 (dd, 1H, $^3J_{H,H}$ = 8.1 Hz, $^4J_{H,H}$ = 1.4 Hz, 4-H), 7.63 (dd, 1H, $^3J_{H,H}$ = 8.1 Hz, $^4J_{H,H}$ = 1.3 Hz, 5-H), 7.91 (dd, 1H, $^3J_{H,H}$ = 7.2 Hz, $^4J_{H,H}$ = 1.3 Hz, 2-H), 7.95 (dd, 1H, $^3J_{H,H}$ = 6.9 Hz, $^4J_{H,H}$ = 1.2 Hz, 7-H). **$^{13}C\{^1H\}$ NMR** (125.71 MHz, 305.0 K, C_6D_6): δ = -3.6 (CH_3, Si-CH_3), 18.7 (C, Si-$C(CH_3)_3$), 28.8 (CH_3, Si-$C(CH_3)_3$), 124.9 (CH, C-6), 126.1 (CH, C-3), 126.8 (CH, C-14), 129.4 (CH, C-12), 130.5 (CH, C-4), 131.3 (CH, C-5), 131.7 (CH, C-13), 132.1 (C,

C-1), 135.3 (C, C-10), 136.4 (C, C-8), 136.6 (C, C-11), 137.4 (CH, C-7), 137.6 (CH, C-2), 140.7 (C, C-9). **^{29}Si{^{1}H} NMR** (99.31 MHz, 305.0 K, C_6D_6): δ = -4.9. **^{29}Si INEPT NMR** (99.31 MHz, 305.0 K, C_6D_6, D3 = D4 = 0.0013 s): δ = -4.8 (dm, $^{1}J_{Si,H}$ = 196.3 Hz). **^{77}Se{^{1}H} NMR** (95.36 MHz, 305.0 K, C_6D_6): δ = 416.2. **^{77}Se NMR** (95.36 MHz, 305.0 K, C_6D_6): δ = 416.3 (d, $^{TS}J_{Se,H}$ = 26.6 Hz), 448.7 (s), 461.6 (s). **GC/MS** t_R: 25.5 min, m/z (%) 383 [M+-H, 37Cl] (0.6), 381 [M+-H, 35Cl] (0.3), 369 (1), 327 (100), 249 (50), 235 (24), 202 (12), 167 (18), 154 (8), 141 (37), 127 (62), 101 (4), 77 (25), 57 (21). **IR** (ATR, 298 K, neat): v = 2131 cm-1. **HR/MS** (LIFDI) $C_{21}H_{24}{}^{80}Se^{28}Si$, calc.: 384.0807, found: 384.0808.

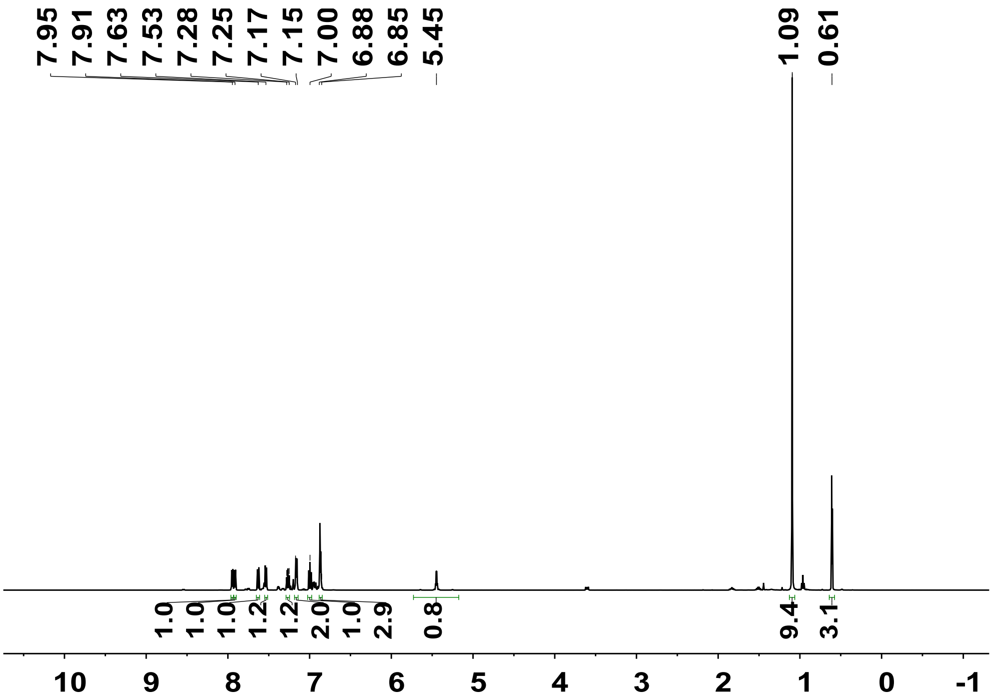

Figure S 27 – ^{1}H NMR spectrum (499.87 MHz, 305.0 K, C_6D_6) of 1-phenylselanyl-8-*tert*-butylmethylsilylnaphthalene **10b**.

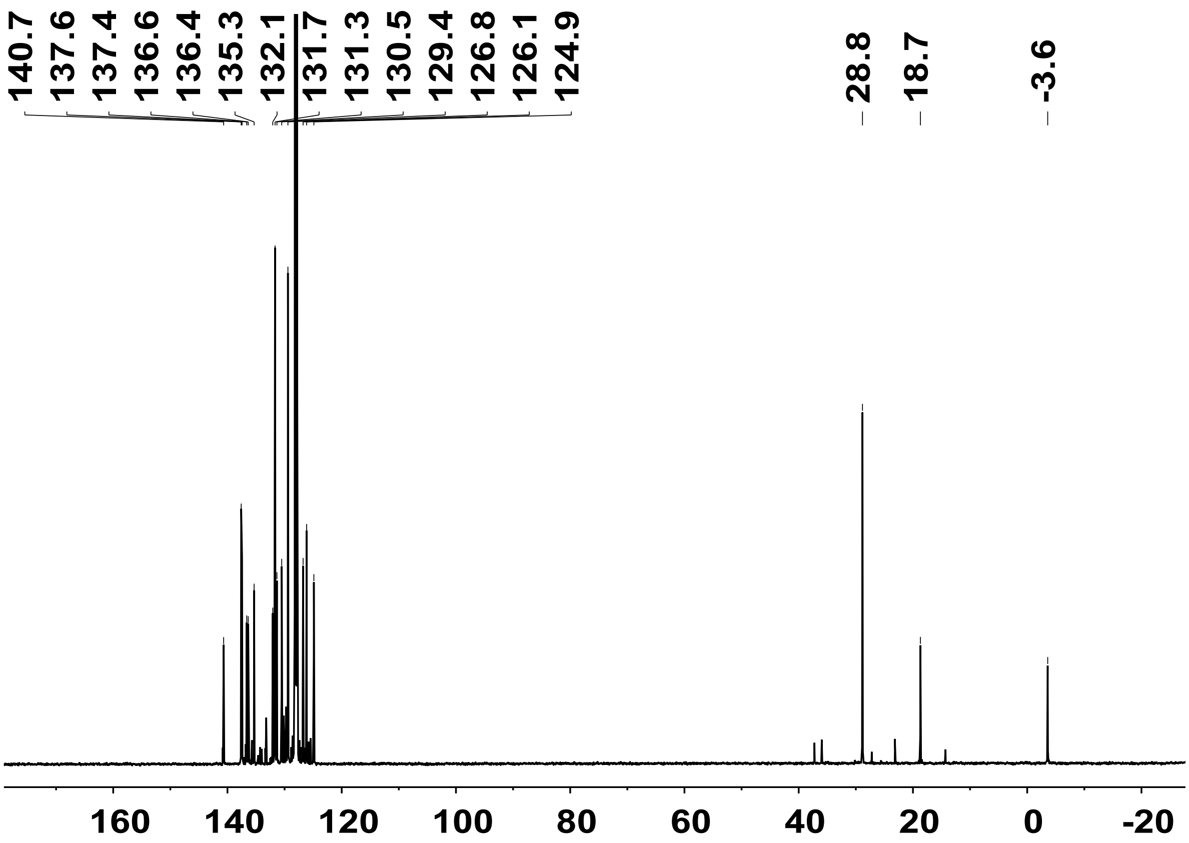

Figure S 28 – ^{13}C{^{1}H} NMR spectrum (125.71 MHz, 305.0 K, C_6D_6) of 1-phenylselanyl-8-*tert*-butylmethylsilylnaphthalene **10b**.

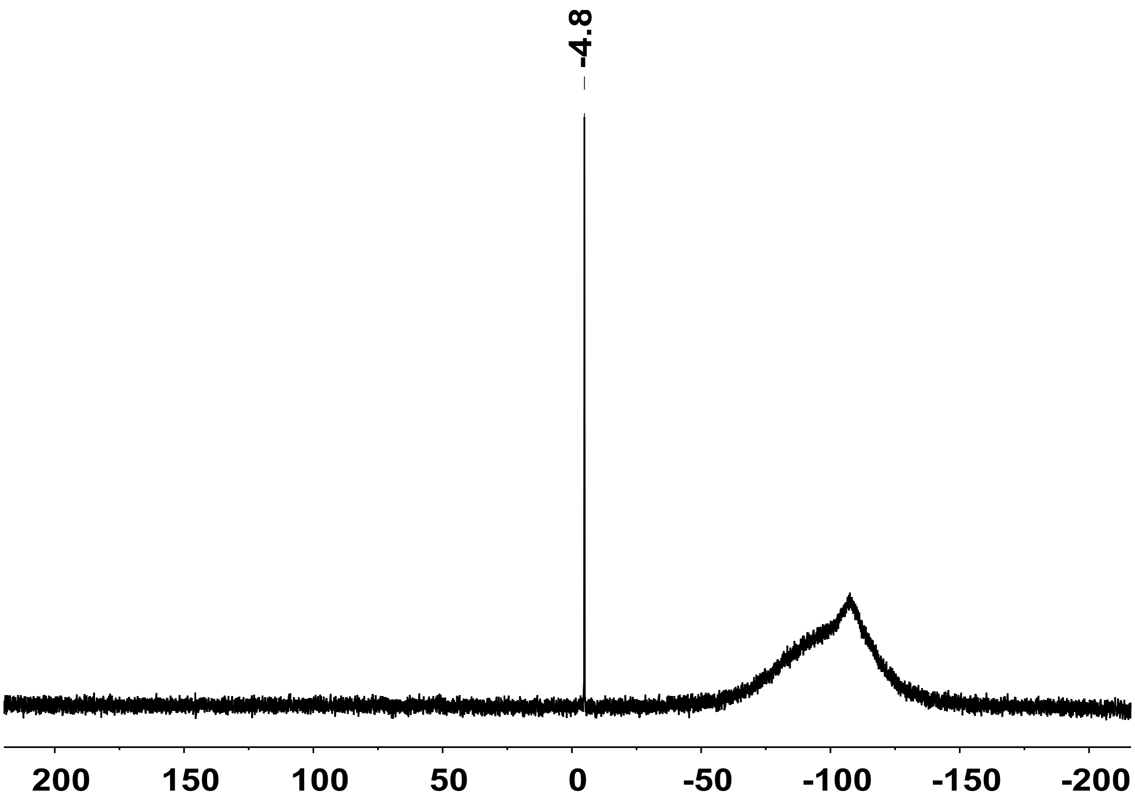

Figure S 29 – ^{29}Si{^{1}H} NMR spectrum (99.31 MHz, 305.0 K, C_6D_6) of 1-phenylselanyl-8-*tert*-butylmethylsilylnaphthalene **10b**.

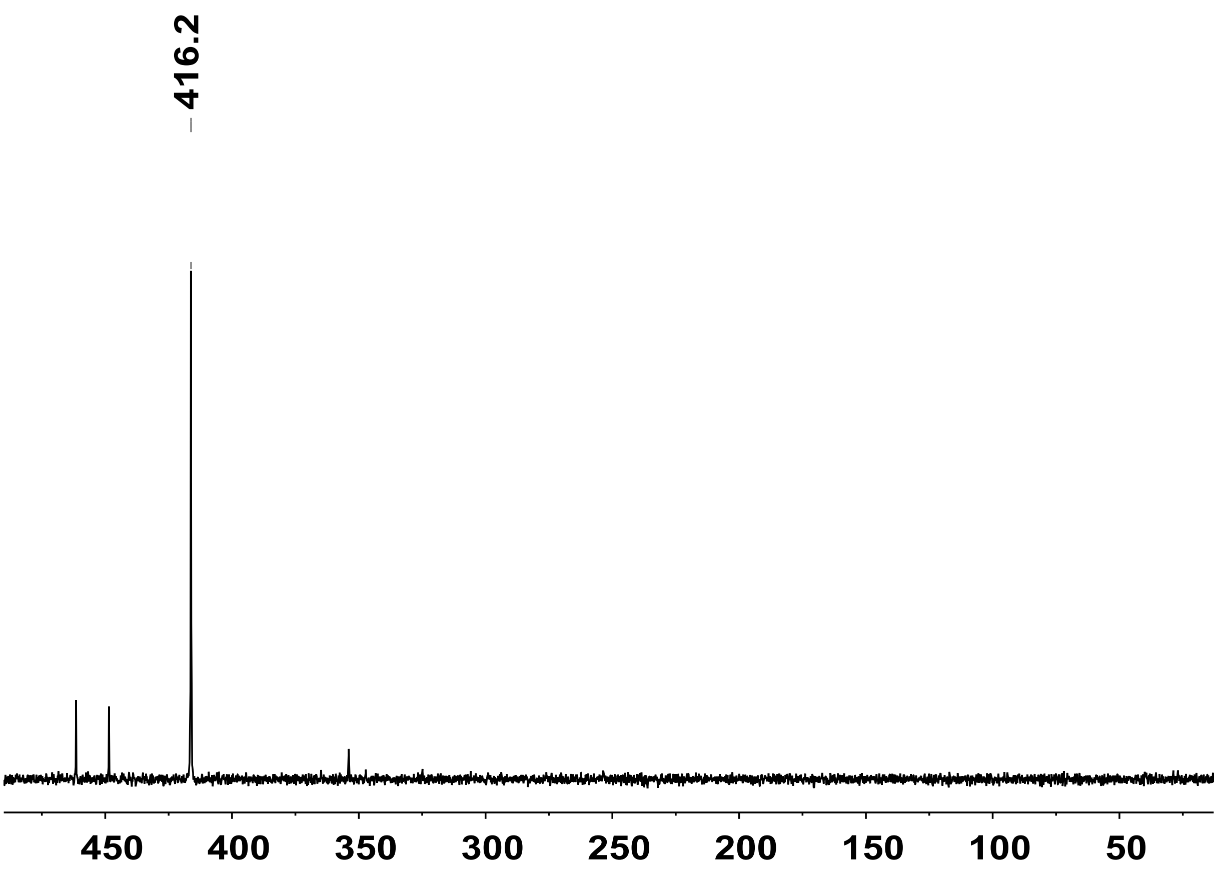

Figure S 30 – $^{77}Se\{^1H\}$ NMR spectrum (95.36 MHz, 305.0 K, C_6D_6) of 1-phenylseleno-8-*tert*-butylmethylsilylnaphthalene **10b**.

5-Phenylselanyl-6-phenylmethylsilylacenaphthene 6

The title compound was synthesized according to general procedure B using 1.06 g (3.00 mmol) 5-bromo-6-(methylphenylsilyl)acenaphthene **5**, 1.9 mL (3.00 mmol) *n*-butyl lithium and 0.94 g (3.00 mmol) diphenyl diselenid. The reaction was carried out at -30 °C. The product was purified by crystallization from *n*-pentane at -28 °C and was obtained as a white solid. Yield 0.8 g (1.86 mmol, 62 %).

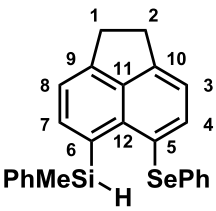

^{1}H NMR (499.87 MHz, 305.0 K, $CDCl_3$): δ = 0.73 (d, 3 H, $^3J_{H,H}$ = 3.6 Hz), 3.34-3.38 (m, 4 H), 5.55 (q, 1 H, $^3J_{H,H}$ = 3.6 Hz, $^1J_{H,Si}$ = 202.2 Hz), 6.91 - 6.93 (m, 2 H), 7.01 – 7.02 (m, 3 H), 7.19 - 7.27 (m, 5 H), 7.43 - 7.44 (m, 2 H), 7.85 - 7.87 (m, 2 H). **$^{13}C\{^1H\}$ NMR** (125.55 MHz, 305.0 K, $CDCl_3$): δ = -0.70 (CH_3), 30.1 (CH_2), 30.3 (CH_2), 119.8 (CH), 120.7 (CH), 124.2 (C), 125.8 (CH), 127.8 (CH), 128.6 (CH), 129.0 (CH), 129.5 (CH), 129.6 (C), 134.6 (CH), 136.4 (C), 139.3 (C), 139.3 (C), 140.6 (C), 140.9 (CH), 141.6 (CH), 149.2 (C), 149.8 (C). **$^{29}Si\{^1H\}$ NMR** (99.31

MHz, 305.0 K, $CDCl_3$): δ = -18.1. **^{77}Se{^{1}H} NMR** (95.36 MHz, 305.0 K, $CDCl_3$): δ = 374.1. **IR** (ATR, 298 K, neat): $\tilde{\nu}$ = 2094 cm-1. **HR/MS** (EI) $C_{25}H_{22}SiSe$, calc.: 430.0651, found: 430.0639. **EA** $C_{25}H_{22}SiSe$, calc.: C 69.91, H 5.16, found: C 70.05, H 5.55.

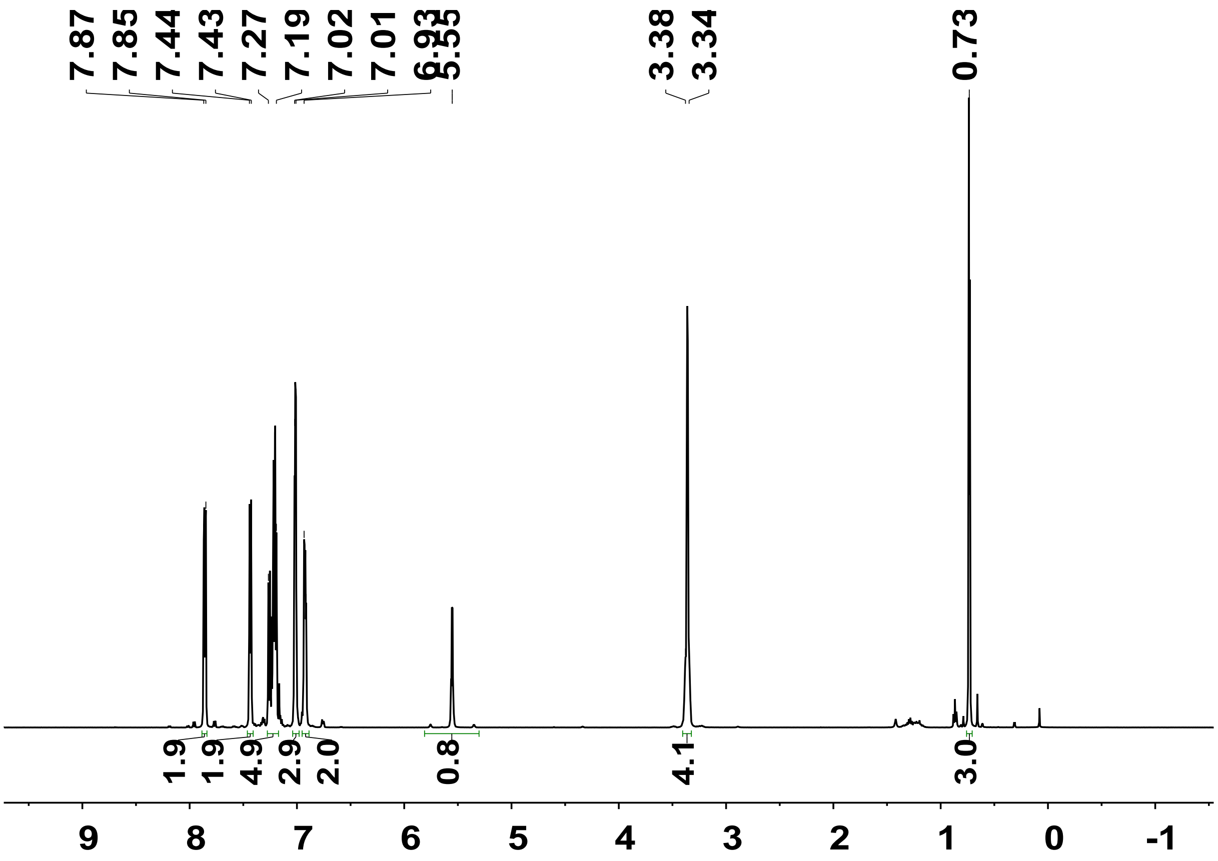

Figure S 31 – ^{1}H NMR spectrum (499.87 MHz, 305.0 K, $CDCl_3$) of 5-phenylselanyl-6-phenylmethylsilylacenaphthene **6**.

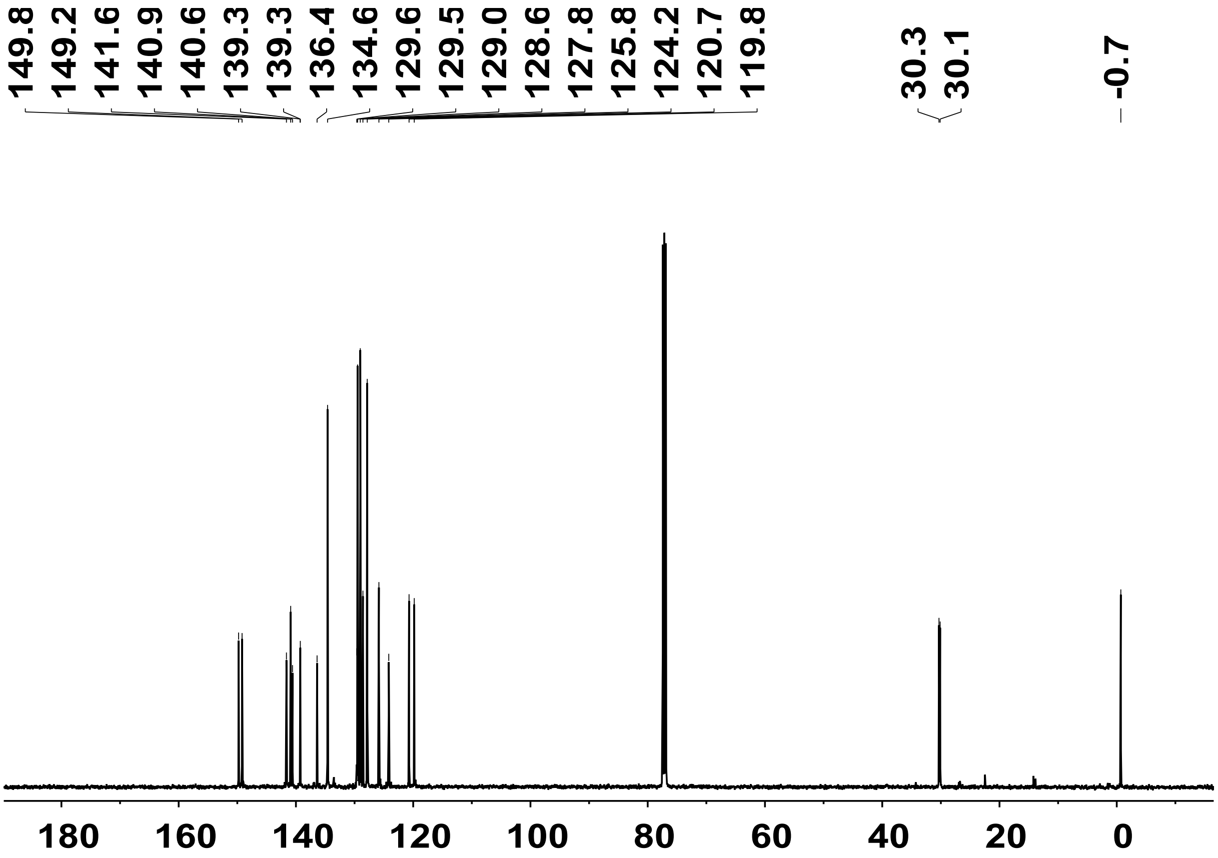

Figure S 32 – $^{13}C\{^{1}H\}$ NMR spectrum (125.55 MHz, 305.0 K, $CDCl_3$) of 5-phenylselanyl-6-phenylmethylsilylacenaphthene **6**.

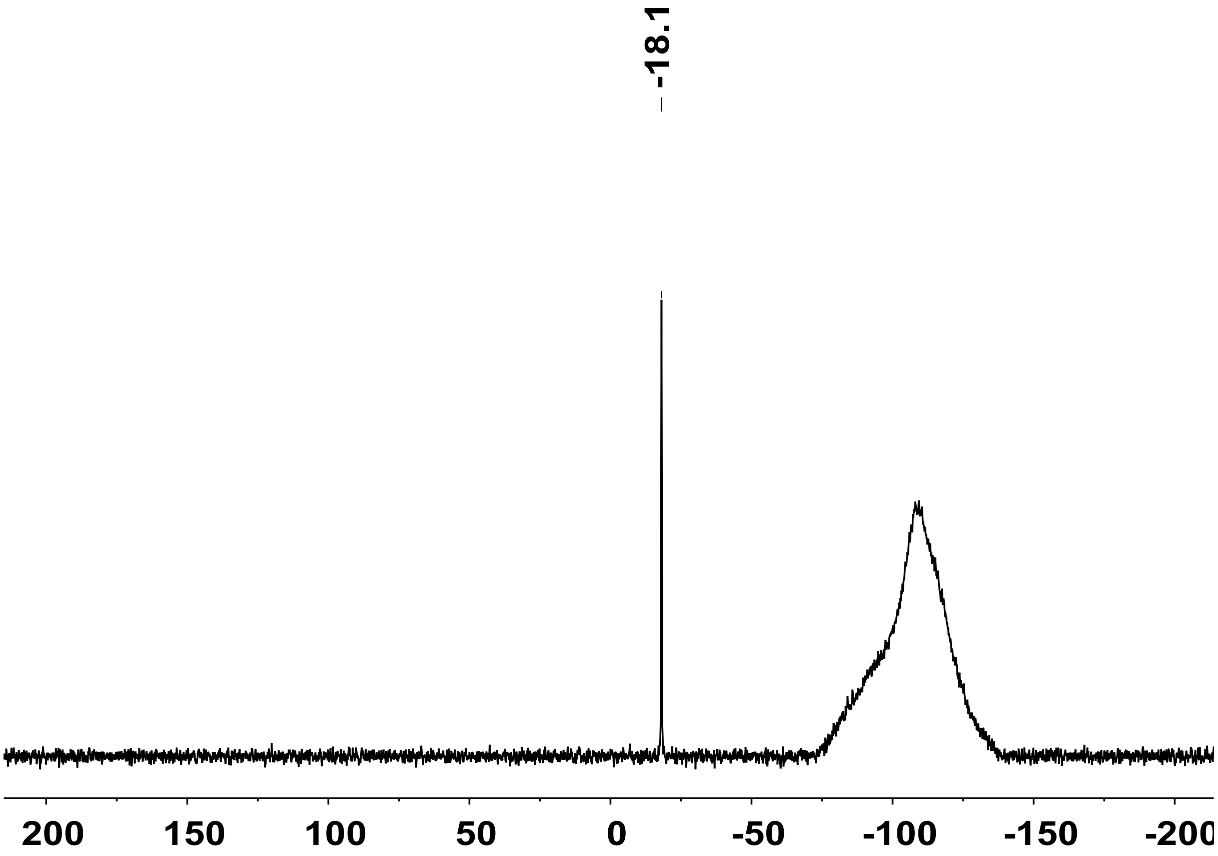

Figure S 33 – $^{29}Si\{^{1}H\}$ NMR spectrum (99.31 MHz, 305.0 K, $CDCl_3$) of 5-phenylselanyl-6-phenylmethylsilylacenaphthene **6**.

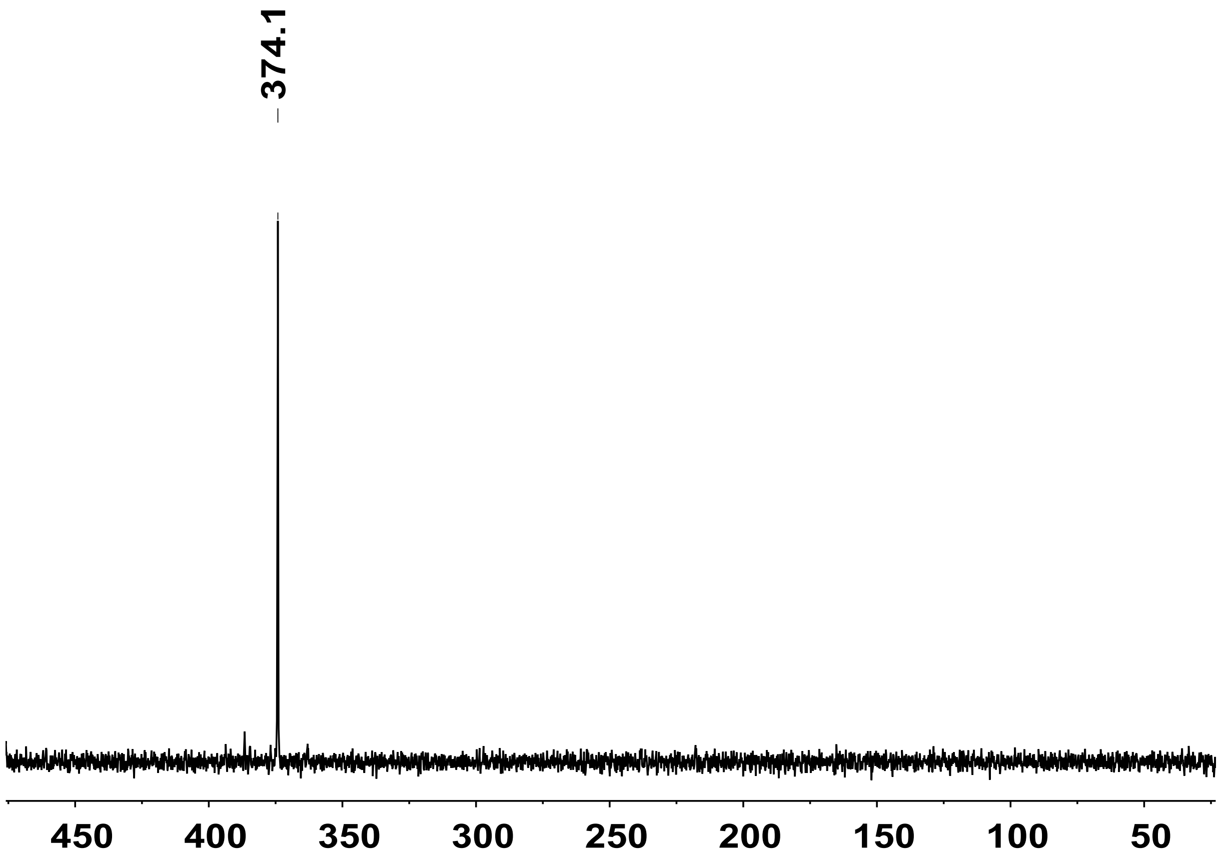

Figure S 34 – $^{77}Se\{^{1}H\}$ NMR spectrum (95.36 MHz, 305.0 K, $CDCl_3$) of 5-phenylselanyl-6-phenylmethylsilylacenaphthene **6**.

5-Mesityltellanyl-6-phenylmethylsilylacenaphthene 7

A solution of 1.77 g (5.00 mmol) 5-bromo-6-(methylphenylsilyl)acenaphthene **5** in diethyl ether (45 mL) was cooled to -10 °C. 3.1 mL (5.00 mmol) *n*-butyl lithium were added dropwise to the solution via syringe and the reaction mixture was stirred for four hours. In a round-bottom flask 2.47 mg (5.00 mmol) dimesityl ditelluride were suspended in diethyl ether (150 mL) and added to the reaction mixture at -80 °C via Teflon tube. The reaction mixture was allowed to warm to room temperature overnight. Then saturated ammonium chloride solution (200 mL) was added, the phases were separated and the aqueous phase was extracted with diethyl ether (3 × 200 mL). The combined organic layers were dried over magnesium sulphate, and the solvent was removed under reduced pressure. After column chromatography (PE/EA, 60:1, Rf = 0.25) only a crude product was obtained. Recrystallization from ethyl acetate yielded the pure product as a slightly beige solid. Yield 441 mg (0.85 mmol, 17 %).

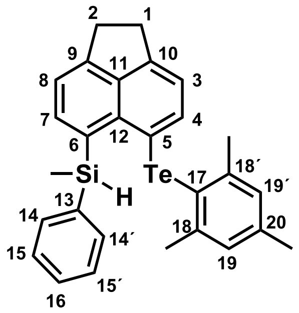

^{1}H NMR (499.87 MHz, 305.1 K, C_6D_6): δ = 0.99 (d, 3 H, $^{3}J_{H,H}$ = 3.5 Hz, $^{TS}J_{H,Te}$ = 9.0 Hz, $SiCH_3$), 2.10 (s, 3 H, *p*-CH_3), 2.45 (s, 6 H, *o*-CH_3), 2.86-2.88 (m, 2 H, H-2), 2.92-2.95 (m, 2 H, H-1), 6.29 (q, 1 H, $^{3}J_{H,H}$ = 3.5 Hz, $^{1}J_{H,Si}$= 193 Hz, $^{TS}J_{H,Te}$ = 66 Hz, SiH), 6.70 (d, 1 H, $^{3}J_{H,H}$ = 7.2 Hz, H-3), 6.81 (s, 2 H, H-19, H-19´), 7.06 (d, 1 H, $^{3}J_{H,H}$ = 7.0 Hz, H-8), 7.23-7.24 (m, 3 H, H-15, H-15´, H-16), 7.69-7.71 (m, 2 H, H-14, H-14´), 7.78 (d, 1 H, $^{3}J_{H,H}$ = 7.2 Hz, H-4), 7.92 (d, 1 H, $^{3}J_{H,H}$ = 7.0 Hz, H-7). **$^{13}C\{^{1}H\}$ NMR** (125.71 MHz, 305.0 K, C_6D_6): δ = 0.0 (CH_3, $SiCH_3$), 21.0 (CH_3, *p*-CH_3), 29.1 (2 × CH_3, *o*-CH_3), 29.6 (CH_2, C-2), 30.2 (CH_2, C-1), 114.2 (C, C−5), 119.5 (CH, C-8), 121.4 (CH, C-3), 125.8 (C, C-17), (128.1 (2 x CH, C-19, C-19´), 128.0, 128.2; overlap with solvent signal), 129.4 (3 × CH, C15, C15´, C16), 130.6 (C, C-6), 135.5 (CH, C-14, C-14´), 138.5 (C, C−13), C138.8 (C, C-20), 138.8 (CH, C-4), 140.4 (CH, C-7), 140.5, 141.3 (2 × C, C-11, C-12), 144.9 (2 × C, C-18, C-18´), 146.8 (C, C-10), 150.0 (C, C-9). **$^{29}Si\{^{1}H\}$ NMR** (99.31 MHz, 305.0 K, C_6D_6): δ = -21.3. **$^{29}Si\{^{1}H\}$ INEPT NMR** (99.31 MHz, 305.0 K, D3 = 0.0122 s, D4 = 0.0313 s, C_6D_6): δ = -21.3. **$^{125}Te\{^{1}H\}$ NMR** (157.74 MHz, 305.0 K, C_6D_6): δ = 432.4 ($^{TS}J_{Te,H}$= 91 Hz). **IR** (ATR, 305 K, neat): $\tilde{\nu}$SiH = 2108 cm-1. **HR/MS** (EI): [C28H28SiTe], calculated: m/z = 522.1017, found: m/z = 522.1000. **m.p.**: 146-148 °C

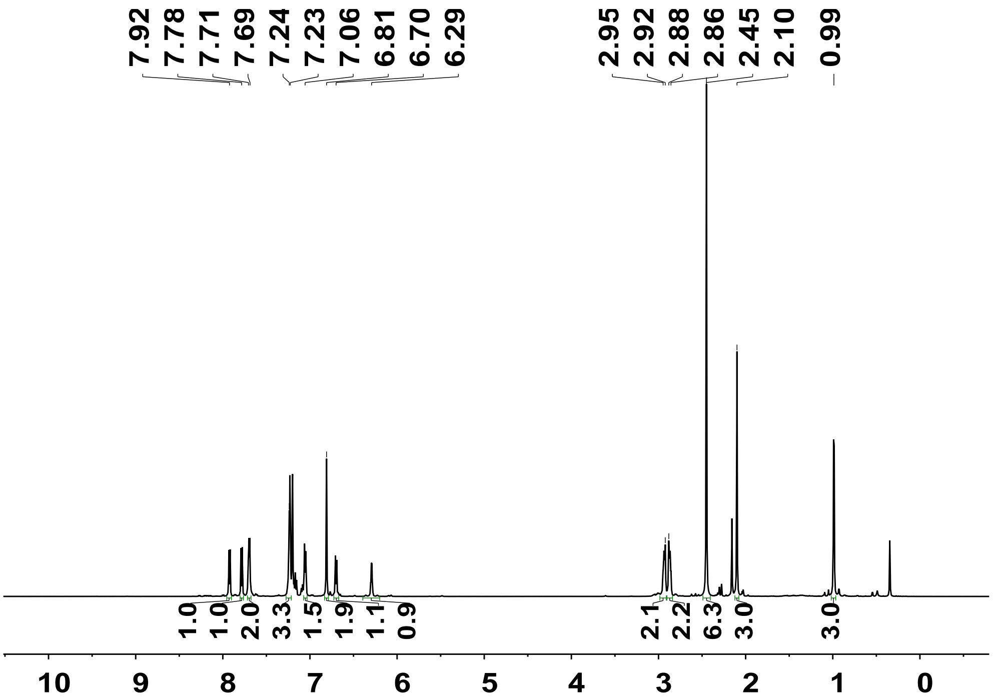

Figure S 35 – ^{1}H NMR spectrum (499.87 MHz, 305.1 K, C_6D_6) of 5-mesityltellanyl-6-phenylmethylsilylacenaphthene **7**.

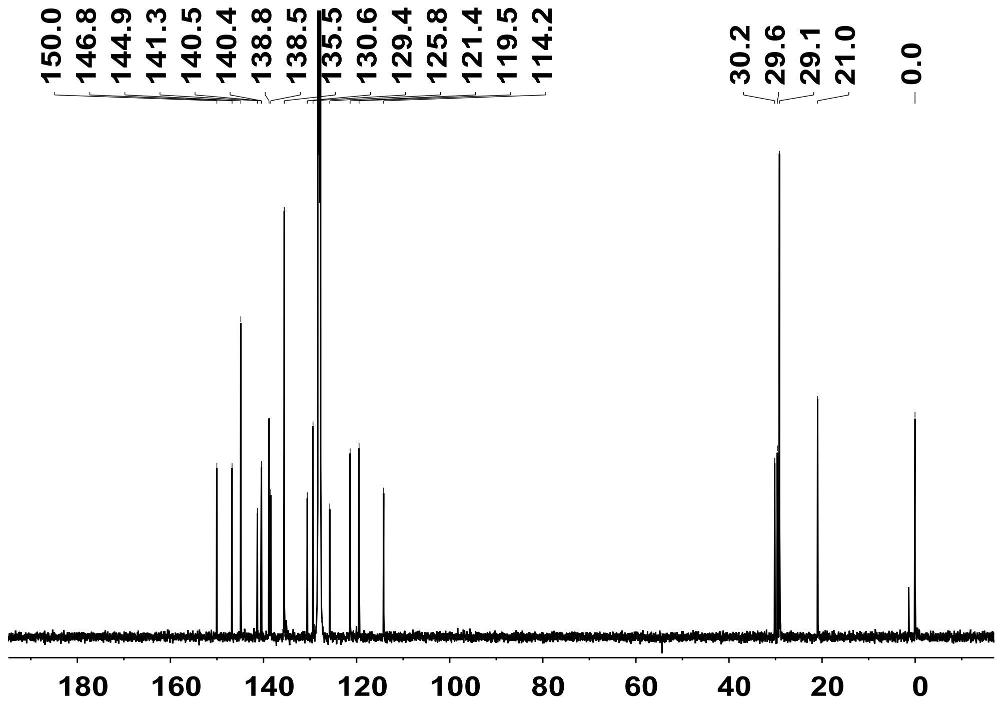

Figure S 36 – $^{13}C\{^{1}H\}$ NMR spectrum (125.71 MHz, 305.0 K, C_6D_6) of 5-mesityltellanyl-6-phenylmethylsilylacenaphthene **7**.

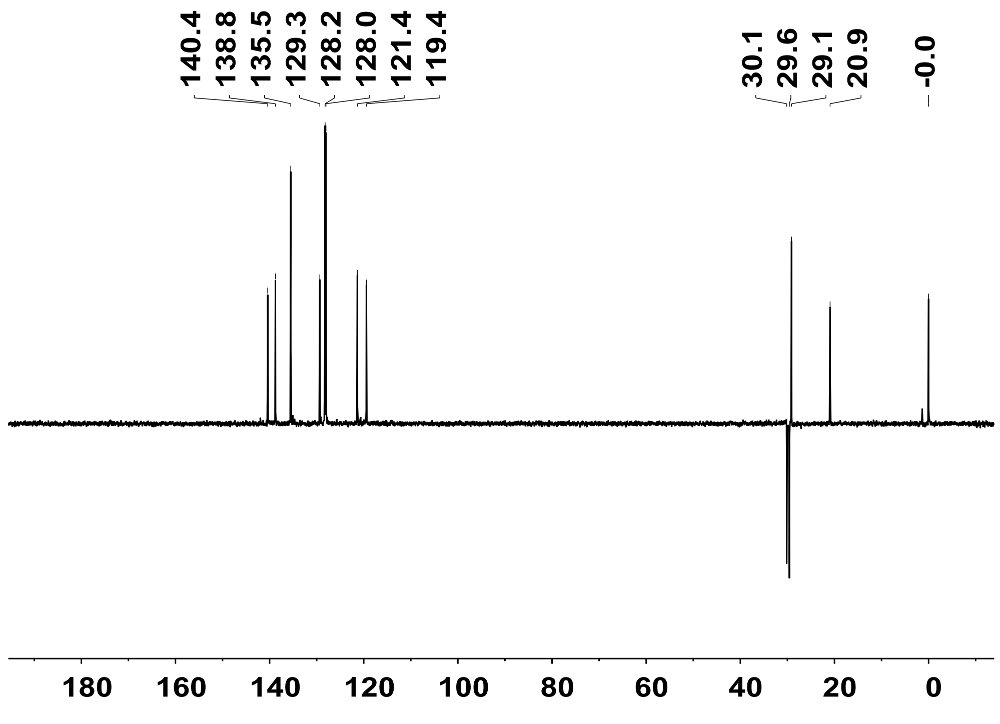

Figure S 37 – $^{13}C\{^{1}H\}$ DEPT NMR spectrum (125.71 MHz, 305.0 K, C_6D_6) of 5-mesityltellanyl-6-phenylmethylsilylacenaphthene **7**.

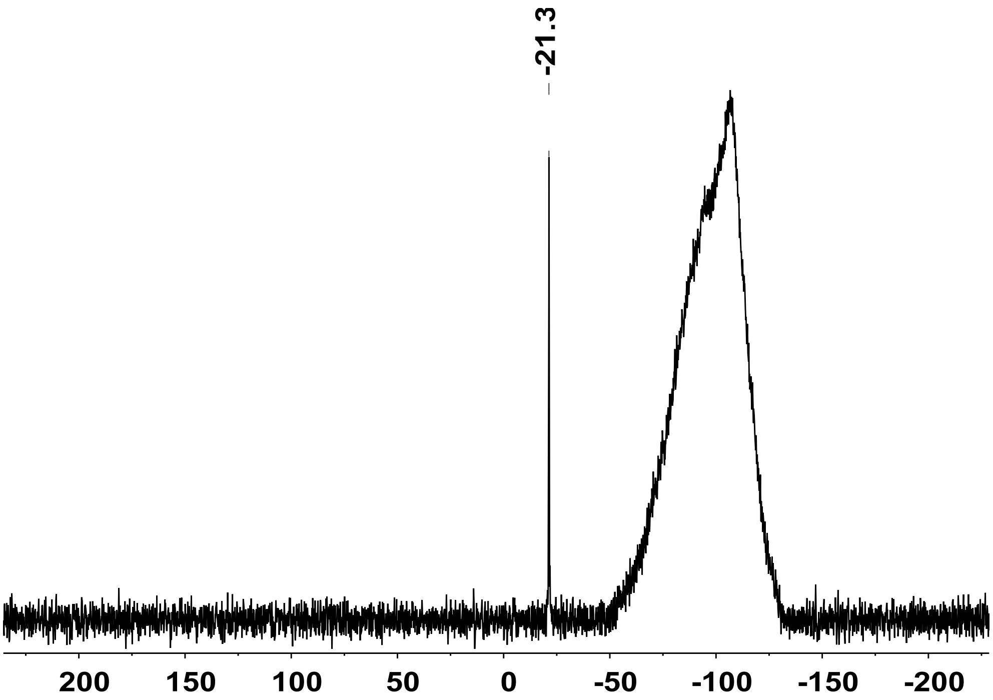

Figure S 38 – $^{29}Si\{^{1}H\}$ NMR spectrum (99.31 MHz, 305.0 K, C_6D_6) of 5-mesityltellanyl-6-phenylmethylsilylacenaphthene **7**.

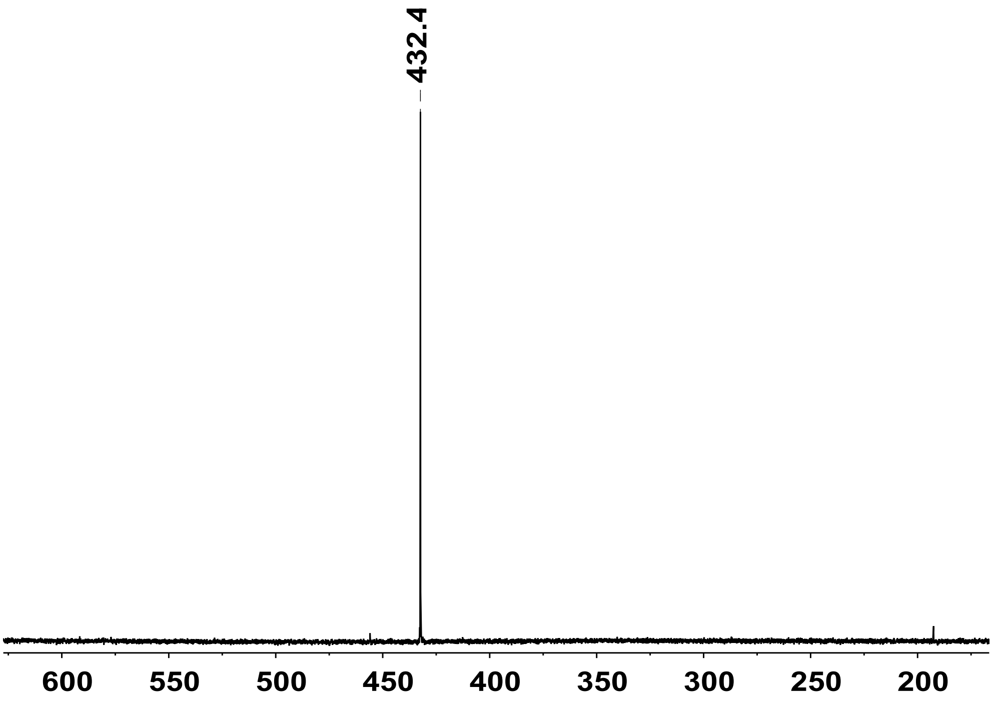

Figure S 39 – $^{125}Te\{^{1}H\}$ NMR spectrum (157.74 MHz, 305.0 K, C_6D_6) of 5-mesityltellanyl-6-phenylmethylsilylacenaphthene **7**.

1.3 Synthesis and characterization of chalcogenyl-stabilized silyl borates

General Procedure D: The silane and trityl borate $[Ph_3C][B(C_6F_5)_4]$ were dissolved in benzene. Then the solution of the trityl borate was added to the silane at r.t. and the biphasic reaction mixture was stirred for 30 min. Subsequently, the phases were separated, the upper, nonpolar phase was removed and the polar phase was washed with benzene three times. After removing the solvent under low pressure, the residue was dissolved in a deuterated solvent and analyzed by NMR spectroscopy.

Phenoxy-Stabilized Methylphenylsilyl borate 13a$[B(C_6F_5)_4]$

The title compound **13a**$[B(C_6F_5)_4]$ was synthesized according to general procedure **D** using 1.1 equiv. (410 µmol, 151 mg) of 5-methylphenylsilyl-6-phenoxyacenaphthene **3a** and 1.0 equiv. (380 µmol, 346 mg) of trityl borate.

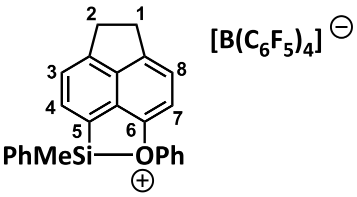

^{1}H NMR (499.9 MHz, 305.0 K, C_6D_6) δ = 0.63 (s, 3 H, $SiCH_3$), 3.07-3.12 (m, 2 H, CH_2), 3.19-3.24 (m, 2 H, CH_2), 6.15 (d, $^3J_{H,H}$ = 7.8 Hz, 1 H, 7-H), 6.82-6.84 (m, 1 H, 8-H), 6.98-7.02 (m, 2 H, SiPh), 7.08-7.13 (m, 3 H, SiPh), 7.28-7.33 (m, 1 H, OPh), 7.35-7.38 (m, 1 H, 3-H), 7.61 (d, $^3J_{H,H}$ = 7.0 Hz, 1 H, 4-H), the signals of *m*- and *o*-OPh were not found. **^{13}C{^{1}H} NMR** (125.7 MHz, 305.0 K, C_6D_6) δ = -3.9 ($SiCH_3$), 30.6 (CH_2), 32.4 (CH_2), 108.7 (CH, C-7), 112.1 (C, C-5), 119.7 (CH, C-8), 121.6 (CH, SiPh), 124.2 (CH, C-3), 125.0 (C, *ipso*-SiPh), 125.5 (C, C-12), 129.4 (CH, SiPh), 131.3 (CH, SiPh), 135.2 (CH), 135.5 (CH, SiPh), 135.9 (CH, C-4), 138.9 (C, C-11), 149.8 (C), 147.9 (C), 151.5 (C), 151.9 (C, C-6). **^{29}Si{^{1}H} NMR** (99.3 MHz, 305.0 K, C_6D_6) δ = 60.8 ppm. **^{11}B{^{1}H} NMR** (160.46 MHz, 305.0 K, C_6D_6) δ = -16.0. **^{19}F{^{1}H} NMR** (470.30 MHz, 305.0 K, C_6D_6) δ = -166.7-(-166.4) (m, 8 F, $[B(C_6F_5)_4]$), -162.6 (t, $^3J_{F,F}$ = 20.6 Hz, 4 F, $[B(C_6F_5)_4]$), -132.1-(-131.8) (m, 8 F, $[B(C_6F_5)_4]$).

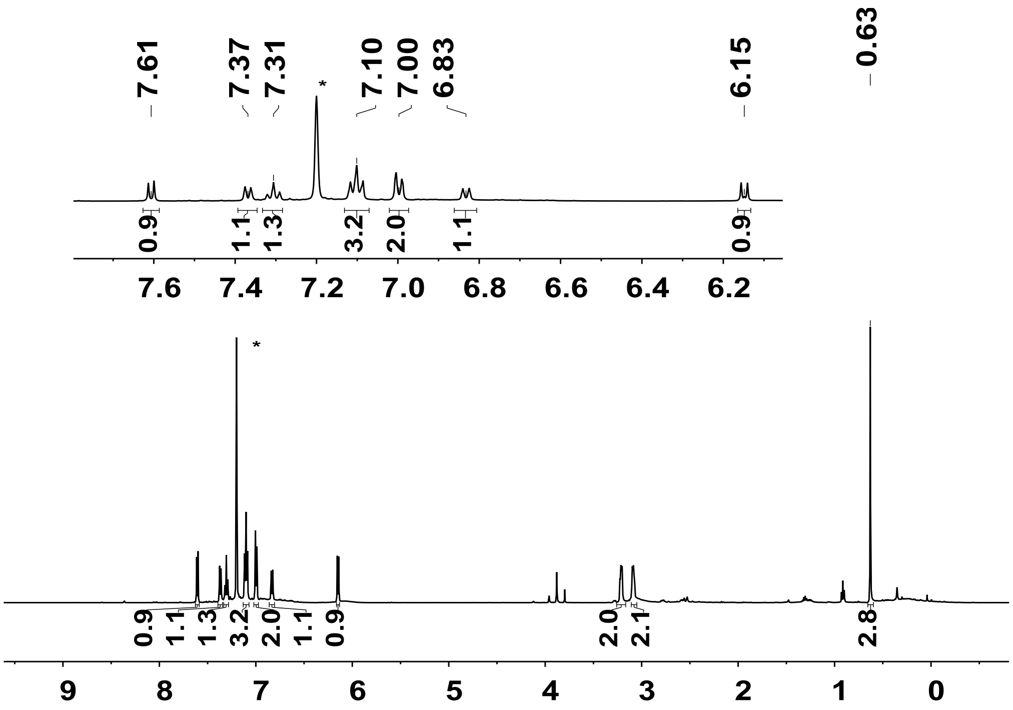

Figure S 40 – ^{1}H NMR spectrum (500 MHz, 305 K, C_6D_6) of phenylmethylsilyl borate **13a**[B(C_6F_5)$_4$] (*C_6D_5H).

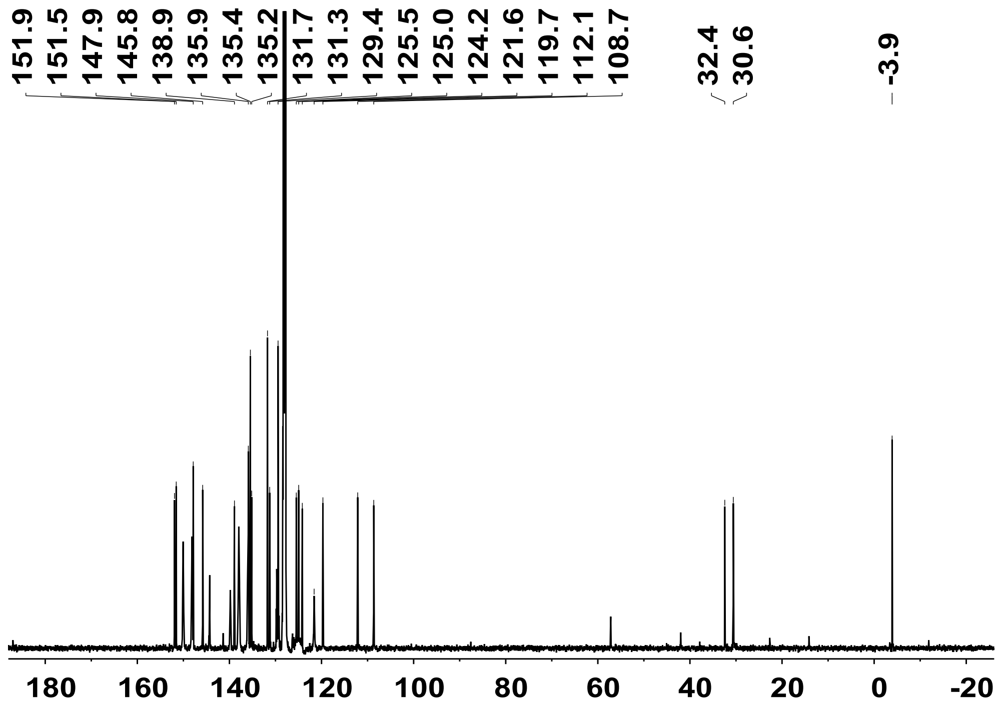

Figure S 41 – $^{13}C\{^{1}H\}$ NMR spectrum (125.7 MHz, 305.0 K, C_6D_6) of phenylmethylsilyl borate **13a**[B(C_6F_5)$_4$].

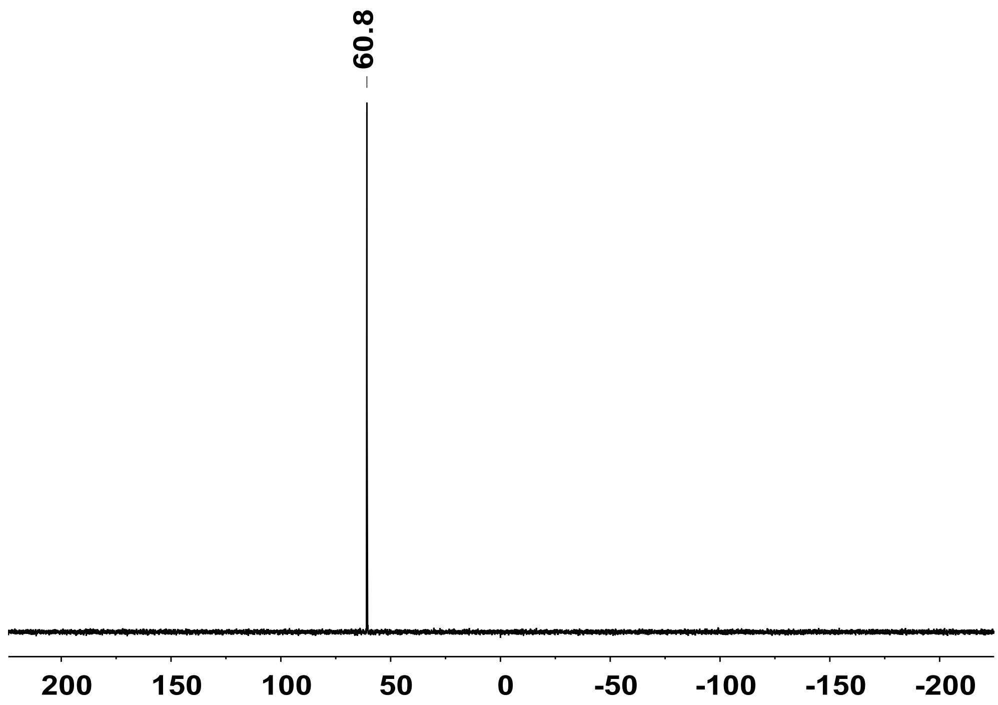

Figure S 42 – $^{29}Si\{^{1}H\}$ NMR spectrum (99.31 MHz, 305.0 K, C_6D_6) of phenylmethylsilyl borate **13a**[$B(C_6F_5)_4$].

Phenoxy-Stabilized *tert*-Butylmethylsilyl Borate 13b[$B(C_6F_5)_4$]

The title compound **13b[$B(C_6F_5)_4$]** was synthesized according to general procedure **D** using 1.0 equiv. (380 µmol, 130 mg) of 5-methylphenylsilyl-6-phenoxyacenaphthene **3b** and 1.0 equiv. (380 µmol, 346 mg) of trityl borate.

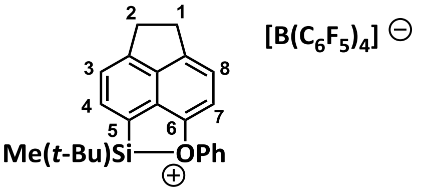

^{1}H NMR (499.9 MHz, 305.1 K, C_6D_6) δ = 0.37 (s, 3 H, CH_3), 0.69 (s, 9 H, *t*-Bu), 2.99-3.03 (m, 2 H, CH_2), 3.11-3.15 (m, 2 H, CH_2), 6.18 (d, $J_{H,H}$ = 7.8 Hz, 1 H, 7-H), 6.78-6.81 (m, 1 H, 8-H), 7.13-7.24 (m, 4 H, overlap with C_6D_5H), 7.28-7.31 (m, 1 H, 3-H), 7.50 (d, $J_{H,H}$ = 7.1 Hz, 1 H, 4-H), OPh signals broad and not listed. **$^{13}C\{^{1}H\}$ NMR** (125.7 MHz, 305.1 K, C_6D_6) δ = -4.9 ($SiCH_3$), 20.8 (C, *t*-Bu), 23.8 (CH_3, *t*-Bu), 30.5 (CH_2), 32.3 (CH_2), 108.7 (CH, C-7), 113.3 (C, C-5), 119.5 (CH, C-8), 121.4 (CH, OPh), 124.0 (CH, C-3), 125.0 (C, C-12), 131.6 (CH, OPh), 132.1 (CH, OPh), 135.1 (CH, C-4), 138.8 (C, C-11), 146.0 (C, C-9), 149.5 (C, *ipso*-OPh), 151.3 (C, C-10), 153.0 (C, C-6). **$^{29}Si\{^{1}H\}$ NMR** (99.3 MHz, 305.1 K, C_6D_6) δ = 72.2. **$^{19}F\{^{1}H\}$ NMR** (470.30 MHz, 305.0 K, C_6D_6) δ = -166.7-(-166.4) (m, 8 F, [$B(C_6F_5)_4$]), -162.6 (t, $^{3}J_{F,F}$ = 20.6 Hz, 4 F, [$B(C_6F_5)_4$]), -132.1-(-131.8) (m, 8 F, [$B(C_6F_5)_4$]).

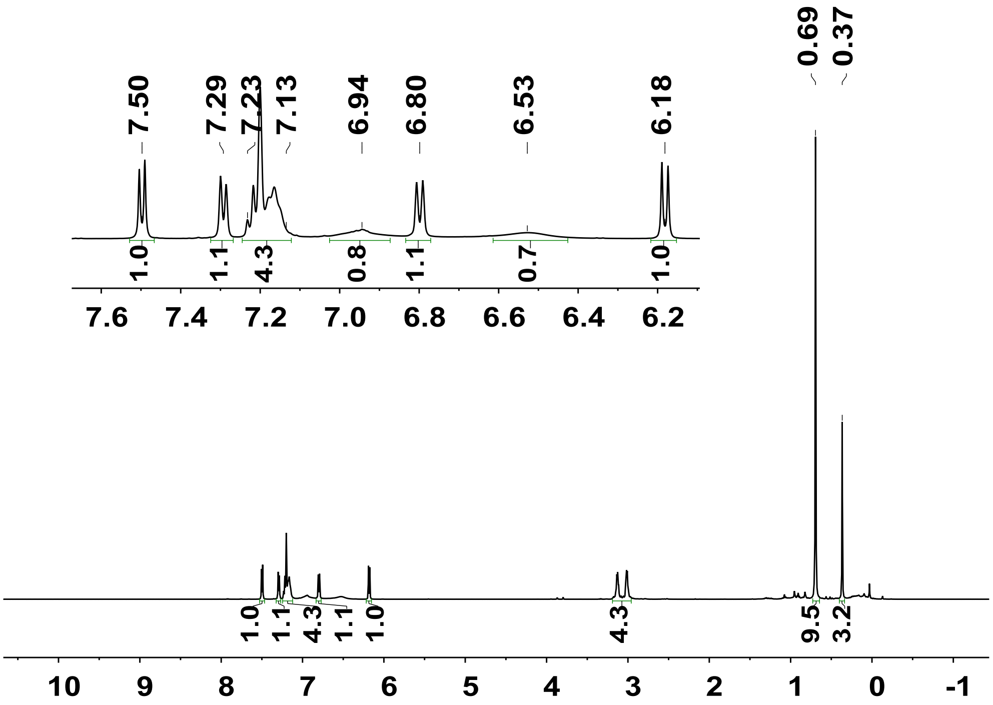

Figure S 43 – ^{1}H NMR spectrum (499.9 MHz, 305.1 K, C_6D_6) of phenoxy-stabilized *tert*-butylmethylsilyl borate **13b**[B(C_6F_5)$_4$].

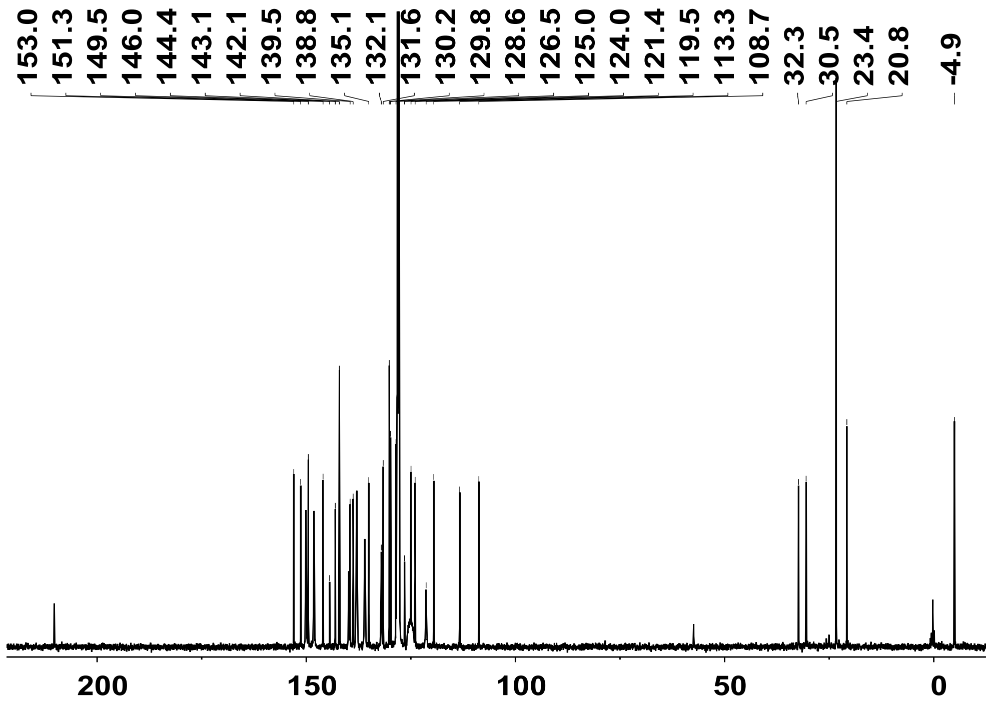

Figure S 44 – ^{13}C{^{1}H} NMR spectrum (125.7 MHz, 305.1 K, C_6D_6) of phenoxy-stabilized *tert*-butylmethylsilyl borate **13b**[B(C_6F_5)$_4$].

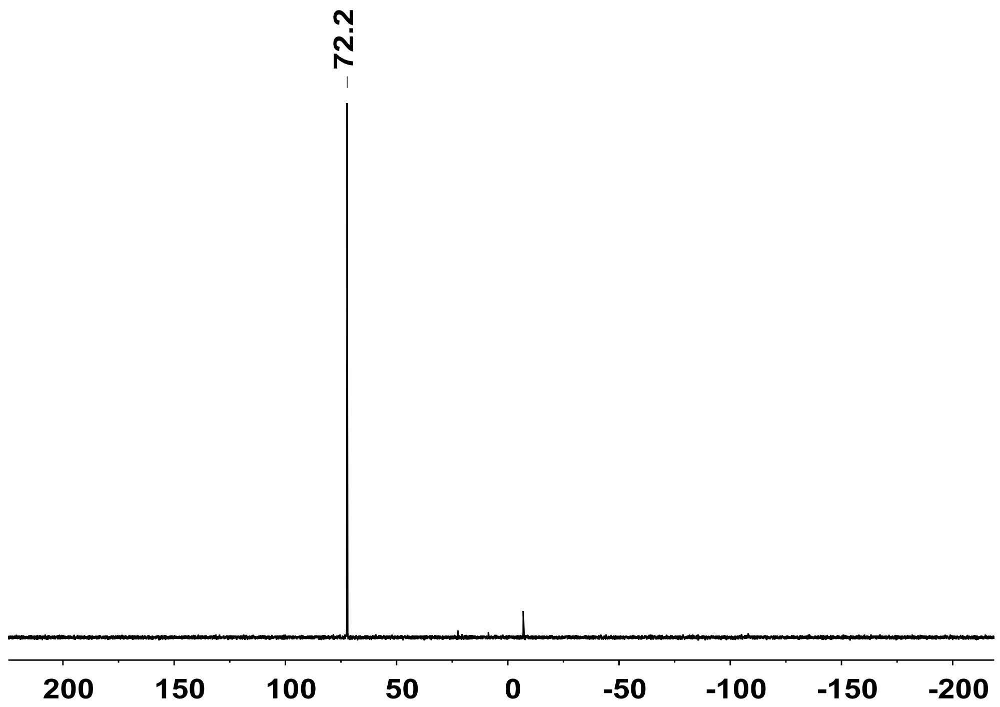

Figure S 45 – $^{29}Si\{^1H\}$ NMR spectrum (99.31 MHz, 305.0 K, C_6D_6) of phenoxy-stabilized *tert*-butylmethylsilyl borate **13b**[B(C_6F_5)$_4$].

Phenylsulfanyl-Stabilized Methylphenylsilyl Borate 13c[B(C_6F_5)$_4$]

The title compound **13c**[B(C_6F_5)$_4$] was synthesized according to general procedure **D** using 1.0 equiv. (347 µmol, 133 mg) of 6-methylphenylsilyl-5-phenylsulfanylacenaphthene **4a** and 1.0 equiv. (347 µmol, 312 mg) of trityl borate.

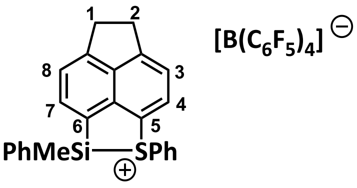

^{1}H NMR (499.9 MHz, 305.1 K, C_7D_8) δ = 0.33 (s, 2 H, $SiCH_3$, *trans*-**13c**), 0.79 (s, 3 H), $SiCH_3$, *cis*-**13c**), 3.03-3.17 (m, 7 H, CH_2), 6.15 (dm, $^3J_{H,H}$ = 7.9 Hz, 2 H, S-*o*-Ph, *cis*-**13c**), 6.54-6.59 (m, 3 H, SPh), 6.70-6.75 (m, 1 H, S-*p*-Ph, *cis*-**13c**), 6.79-6.87 (m, 4 H), 6.92-6.99 (m, 3 H, SPh), 7.02-7.20 (m, 9 H), 7.22-7.27 (m, 2 H), 7.40 (d, $^3J_{H,H}$ = 7.0 Hz, 1 H, 7-H, *cis*-**13c**), 7.43 (d, $^3J_{H,H}$ = 7.0 Hz, 0.6 H, 7-H, *trans*-**13c**). **$^{13}C\{^1H\}$ NMR** (125.7 MHz, 305.1 K, C_7D_8) δ = -4.4 ($SiCH_3$, *trans*-**13c**), -2.8 ($SiCH_3$, *cis*-**13c**), 31.0 (CH_2), 31.4 (CH_2), 116.1 (C), 117.3 (C), 119.0 (C-6), 122.7 (CH), 122.8 (CH), 123.4 (CH), 123.5 (CH), 123.6 (C), 125.5 (CH), 126.5 (C), 127.8 (CH, SPh), 128.3 (CH), 128.6 (CH), 129.0 (CH), 129.3 (CH), 129.7 (CH), 130.5 (CH), 131.5 (CH, SPh), 131.6 (CH), 132.8 (CH, SPh), 133.9 (CH), 134.2 (CH), 134.4 (CH), 134.9 (CH), 135.8 (CH), 137.0 (dm, $^1J_{C,F}$ = 246.3 Hz, [B(C_6F_5)$_4$]$^-$), 138.3 (CH, C-7), 138.4 (CH, C-7), 138.9 (dm, $^1J_{C,F}$ = 244.1 Hz, [B(C_6F_5)$_4$]$^-$), 139.4 (C), 139.6 (C), 139.7 (C), 139.9 (C), 149.2 (dm, $^1J_{C,F}$

= 240.0 Hz, $[B(C_6F_5)_4]^-$), 153.0 (C), 154.4 (C), 154.5 (C). **^{29}Si{^{1}H} NMR** (99.3 MHz, 305.1 K, C_7D_8) δ = 53.9 (*cis*-**13c**), 51.2 (*trans*-**13c**). **^{11}B{^{1}H} NMR** (160.38 MHz, 305.1 K, C_7D_8) δ = -16.0. **^{19}F{^{1}H} NMR** (470.30 MHz, 305.1 K, C_7D_8) δ = -166.6-(-166.4) (m, 8 F, $[B(C_6F_5)_4]$,), -162.7 (t, $^3J_{F,F}$ = 20.6 Hz, 4 F, $[B(C_6F_5)_4]$), -131.8-(-131.6) (m, 8 F, $[B(C_6F_5)_4]$).

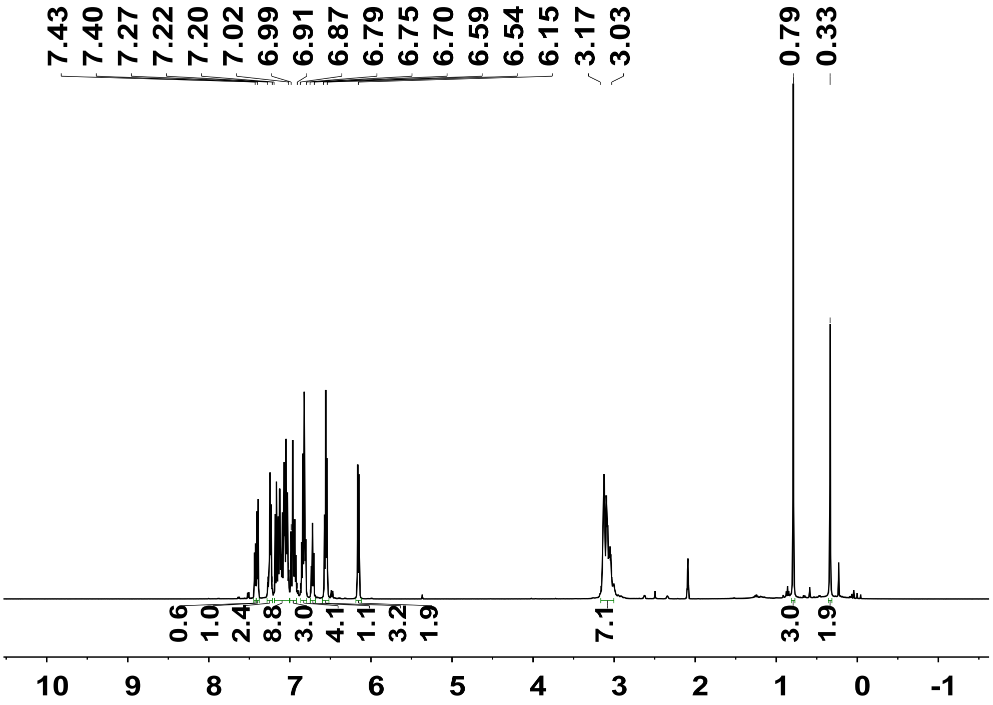

Figure S 46 – ^{1}H NMR spectrum (499.9 MHz, 305.1 K, C_7D_8) of phenylsulfanyl-stabilized methylphenylsilyl borate **13c**$[B(C_6F_5)_4]$.

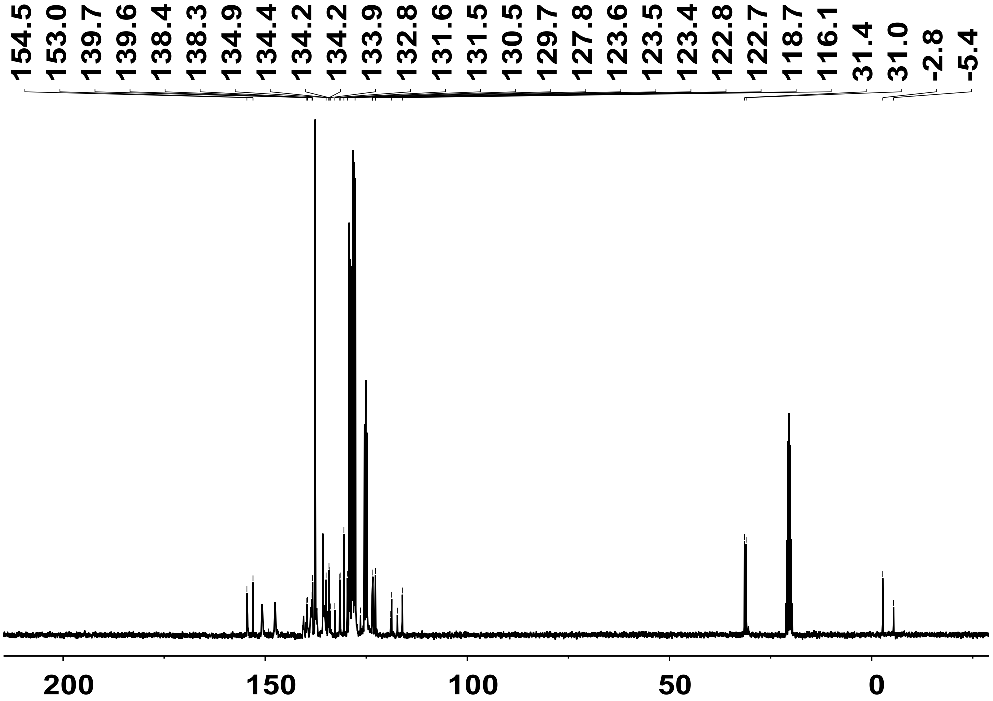

Figure S 47 – ^{13}C{^{1}H} NMR spectrum (125.7 MHz, 305.1 K, C_7D_8) of phenylsulfanyl-stabilized methylphenylsilyl borate **13c**$[B(C_6F_5)_4]$.

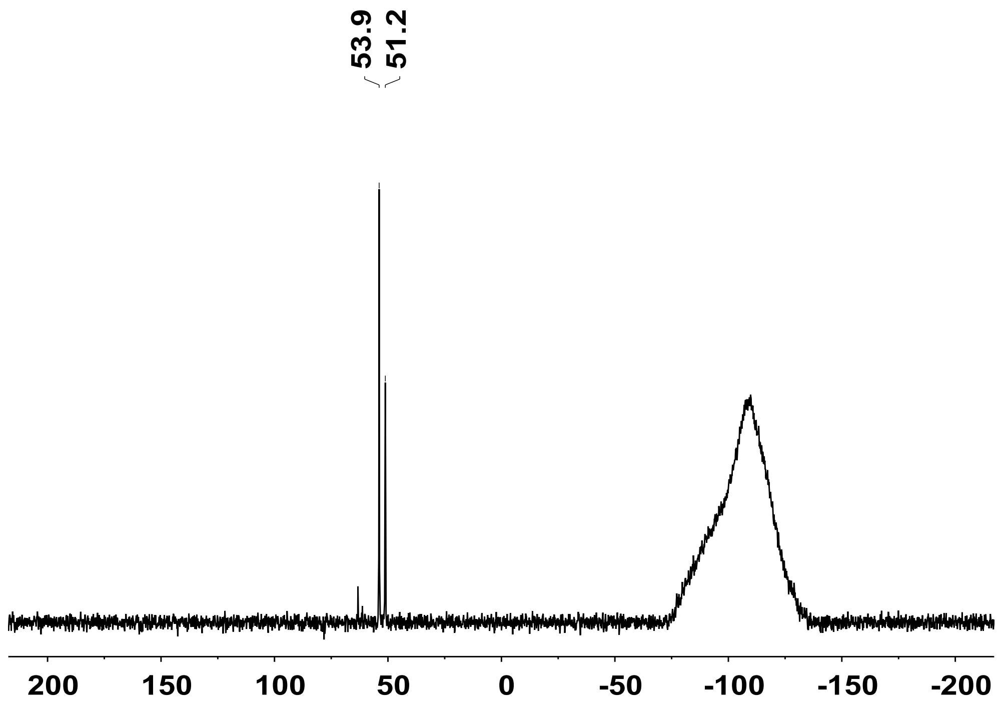

Figure S 48 – $^{29}Si\{^1H\}$ NMR spectrum (99.3 MHz, 305.1 K, C_7D_8) of phenylsulfanyl-stabilized methylphenylsilyl borate **13c**[B(C_6F_5)$_4$].

Naphthyl-Substituted Phenylsulfanyl-Stabilized Methylphenylsilyl Borate 14a[B(C_6F_5)$_4$]

The title compound **37b**[B(C_6F_5)$_4$] was synthesized according to general procedure **D** using 1.0 equiv. (433 µmol, 154 mg) of 8-methylphenylsilyl-1-phenylsulfanylnaphthalene **9** and 1.0 equiv. (433 µmol, 400 mg) of trityl borate.

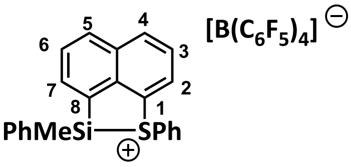

^{1}H NMR (499.87 MHz, 304.9 K, C_7D_8) δ = 0.33 (s, 2 H, SiC**H**$_3$, *trans*-**14a**), 0.73 (s, 3 H, SiC**H**$_3$, *cis*-**14a**), 6.10 (d, $^3J_{H\text{-}H}$ = 7.9 Hz, 2 H, S-*o*-Ph, *cis*-**14a**), 6.48 (d, $^3J_{H\text{-}H}$ = 7.9 Hz, 1 H, *S*-*o*-Ph, *trans*-**14a**), 6.56 (t, $^3J_{H\text{-}H}$ = 7.7 Hz, 2 H), 6.72-6.77 (m, 3 H), 6.82-6.85 (m, 2 H), 6.87-6.88 (m, 1 H), 6.91-6.94 (m, 2 H), 6.97-6.98 (m, 1 H), 7.03-7.08 (m, 4 H), 7.11-7.22 (m, 4 H), 7.24-7.28 (m, 2 H), 7.34-7.36 (m, 2 H), 7.41-7.44 (m, 2 H), 7.50-7.53 (m, 0.5 H), 7.75-7.81 (m, 3.5 H). **^{13}C{^{1}H} NMR** (75.48 MHz, 294.3 K, C_7D_8) δ = -6.1 (SiCH_3, trans-**14a**), -3.4 (CH_3, cis-**14a**), 122.4 (C), 122.7 (C), 124.5 (C), 124.6 (C), 125.1 (C), 127.9 (CH), 128.7 (CH), 128.7 (CH), 129.1 (CH), 129.3 (CH), 129.3 (CH), 129.7 (C), 127.8 (CH), 130.3 (CH), 130.7 (CH), 131.6 (CH), 131.8 (CH), 132.9 (C), 133.0 (CH), 133.3 (CH), 133.9 (C), 134.0 (C), 134.2 (CH), 134.3 (CH), 134.3 (C), 134.5 (C), 137.0 (dm, *J* = 246.6 Hz, [B(C_6F_5)$_4$]$^-$), 135.0 (CH), 135.8 (CH), 136.7

(C), 136.8 (CH), 138.9 (dm, J = 245.4 Hz, $[B(C_6F_5)_4]^-$), 139.7 (C), 139.9 (C), 142.3 (CH), 143.2 (CH), 149.2 (dm, J = 241.6 Hz, $[B(C_6F_5)_4]^-$), signal of *i*-C of $[B(C_6F_5)_4]^-$ overlaps with solvent signal. **^{29}Si{^{1}H} NMR** (99.31 MHz, 305.0 K, C_7D_8) δ = 42.9 (*trans*-**14a**), 45.5 (*cis*-**14a**).

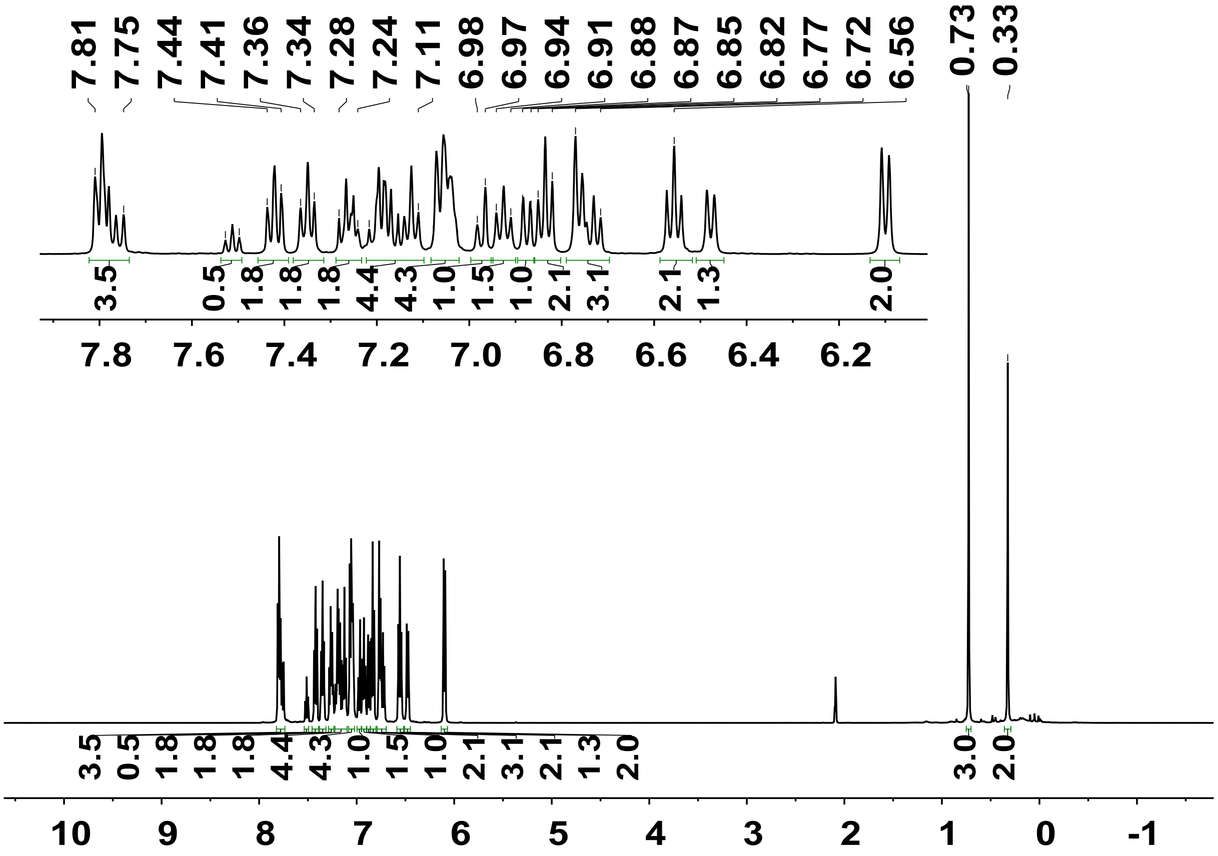

Figure S 49 – ^{1}H NMR spectrum (499.87 MHz, 304.9 K, C_7D_8) of phenylsulfanyl-stabilized methylphenylsilyl borate **14a**$[B(C_6F_5)_4]$.

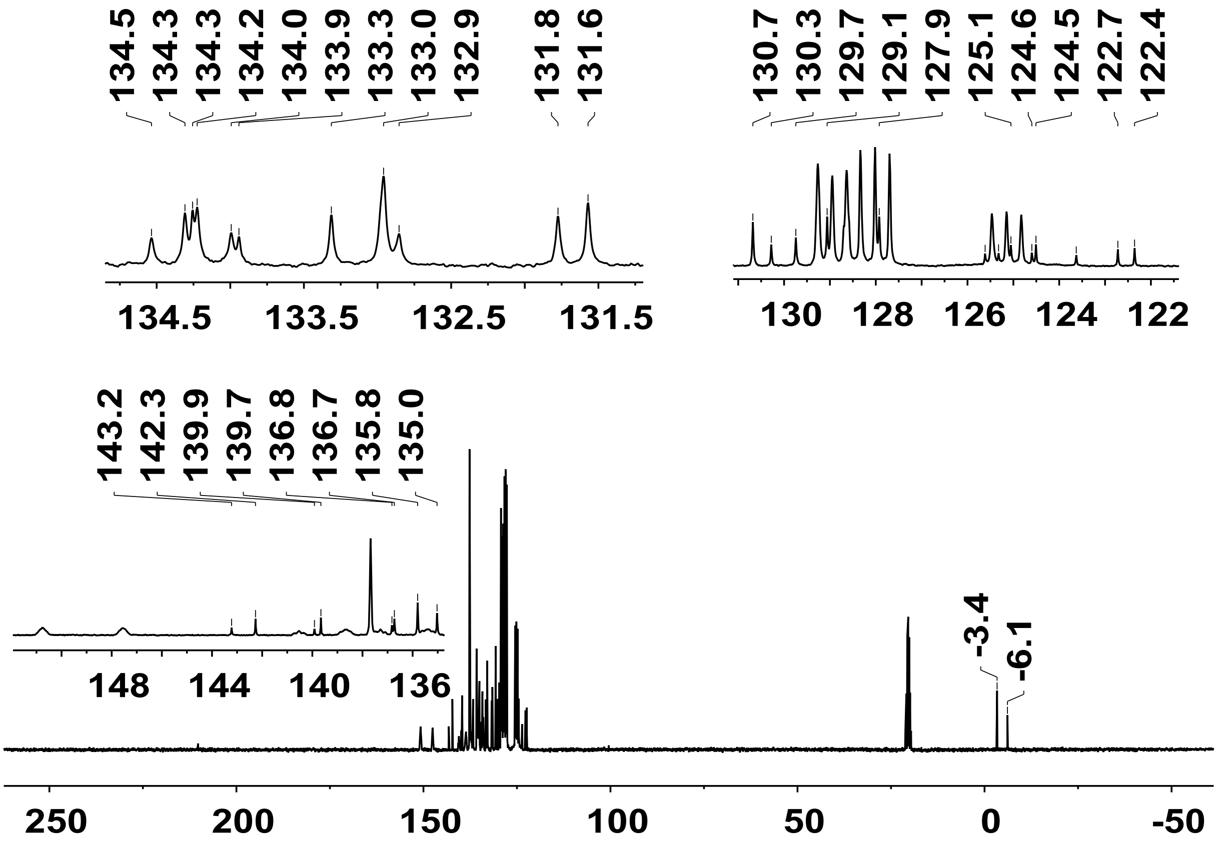

Figure S 50 – $^{13}C\{^1H\}$ NMR spectrum (75.48 MHz, 294.3 K, C_7D_8) of phenylsulfanyl-stabilized methylphenylsilyl borate **14a**[$B(C_6F_5)_4$].

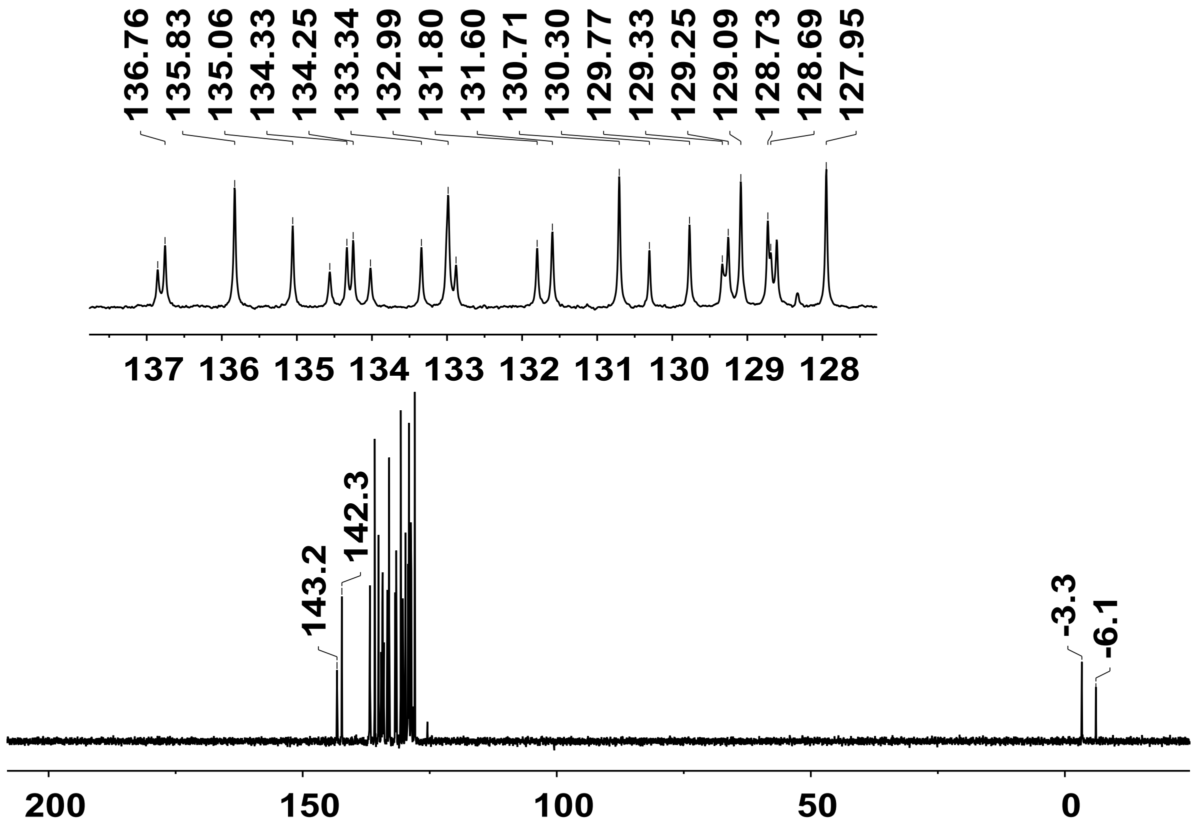

Figure S 51 – $^{13}C\{^{1}H\}$ DEPT NMR spectrum (75.48 MHz, 294.3 K, C_7D_8) of phenylsulfanyl-stabilized methylphenylsilyl borate **14a**[$B(C_6F_5)_4$].

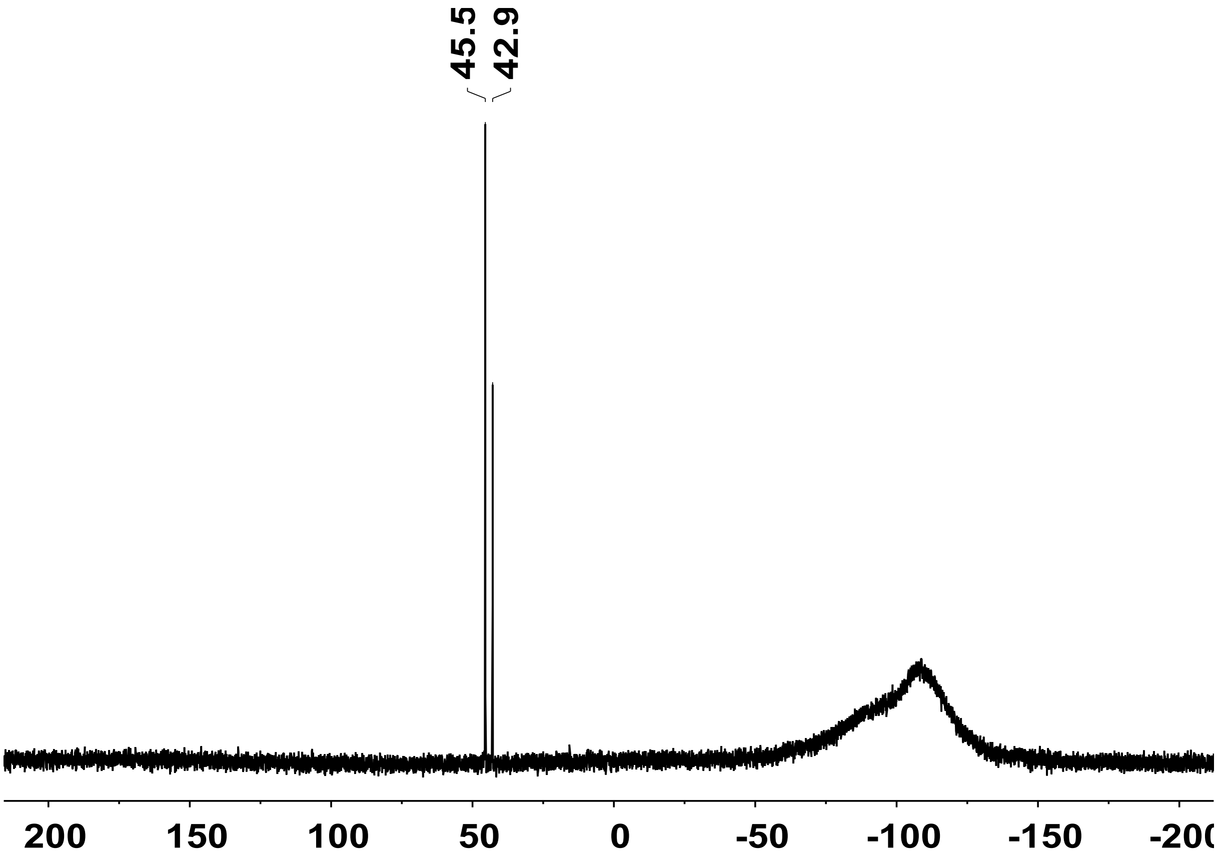

Figure S 52 – $^{29}Si\{^{1}H\}$ NMR spectrum (99.3 MHz, 305.1 K, C_7D_8) of phenylsulfanyl-stabilized methylphenylsilyl borate **14a**[$B(C_6F_5)_4$].

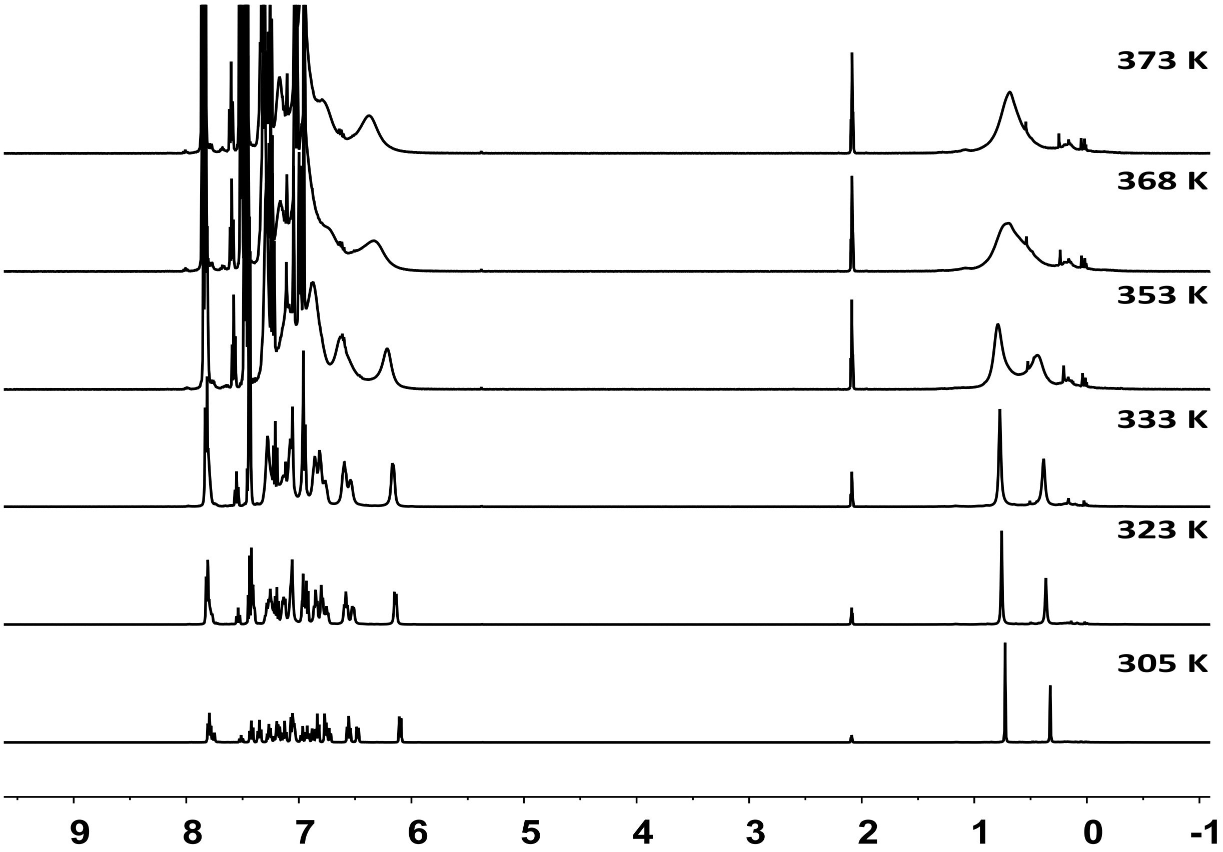

Figure S 53 – ^{1}H VT NMR spectra (500 MHz, C_7D_8) of phenylsulfanyl-stabilized methylphenylsilyl borate **14a**[B(C_6F_5)$_4$].

Phenylsulfanyl-Stabilized *tert*-Butylmethylsilyl Borate 13d[B(C_6F_5)$_4$]

The title compound **35c**[B(C_6F_5)$_4$] was synthesized according to general procedure **D** using 1.0 equiv. (389 µmol, 141 mg) of 6-*tert*-butylmethylsilyl-5-phenylsulfanylacenaphthene **4b** and 1.0 equiv. (389 µmol, 359 mg) of trityl borate.

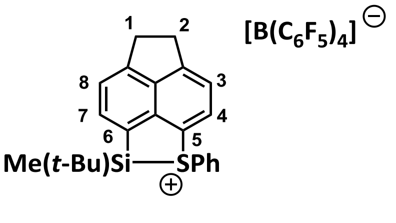

^{1}H NMR (499.9 MHz, 305.1 K, C_6D_6) δ = 0.12 (s, 3H, CH_3), 0.89 (s, 9 H, *t*-Bu), 3.05-3.25 (m, 4 H, CH_2), 6.67 (dm, $J_{H,H}$ = 7.9 Hz, 2 H, o-Ph), 7.03-7.07 (m, 2 H, *m*-Ph), 7.11-7.12 (m, 1 H, 3-H), 7.13-7.16 (m, 1 H, *p*-Ph), 7.27-7.29 (m, 1 H, 8-H), 7.34 (dm, $J_{H,H}$ = 7.0 Hz, 1 H, 8-H), 7.56 (d, $J_{H,H}$ = 7.0 Hz, 1 H, 7-H). **^{13}C{^{1}H} NMR** (125.7 MHz, 305.0 K, C_6D_6) δ = -7.3 ($SiCH_3$), 20.7 (C, *t*-Bu), 24.3 (CH_3, *t*-Bu), 30.9 (CH_2), 31.3 (CH_2), 116.7 (C), 119.6 (C-6), 122.6 (CH, C-3), 123.1 (CH, C-8), 124.4 (C), 124.4-126.1 (brm, [B(C_6F_5)$_4$]$^-$), 128.4 (CH, *o*-Ph), 131.4 (CH, *m*-Ph), 132.6 (CH, *p*-Ph), 133.5 (CH, C-4), 137.0 (dm, $^1J_{C,F}$ = 240.1 Hz, [B(C_6F_5)$_4$]$^-$), 137.4 (CH,

C-7), 138.9 (dm, $^{1}J_{C,F}$ = 238.5 Hz, $[B(C_6F_5)_4]^-$), 139.3 (C), 139.3 (C), 149.1 (dm, $^{1}J_{C,F}$ = 244.1 Hz, $[B(C_6F_5)_4]^-$), 152.5 (C), 154.4 (C). **^{29}Si{^{1}H} NMR** (99.3 MHz, 305.0 K, C_6D_6) δ = 70.0. **^{29}Si{^{1}H} NMR** (99.3 MHz, 297.9 K, CD_2Cl_2) δ = 70.5. **^{11}B{^{1}H} NMR** (160.38 MHz, 305.0 K, C_6D_6) δ = -16.0. **^{19}F{^{1}H} NMR** (470.30 MHz, 305.1 K, C_6D_6) δ = -166.6-(-166.4) (m, 8 F, $[B(C_6F_5)_4]$,), -162.7 (t, $^{3}J_{F,F}$ = 20.7 Hz, 4 F, $[B(C_6F_5)_4]$), -132.0-(-131.7) (m, 8 F, $[B(C_6F_5)_4]$).

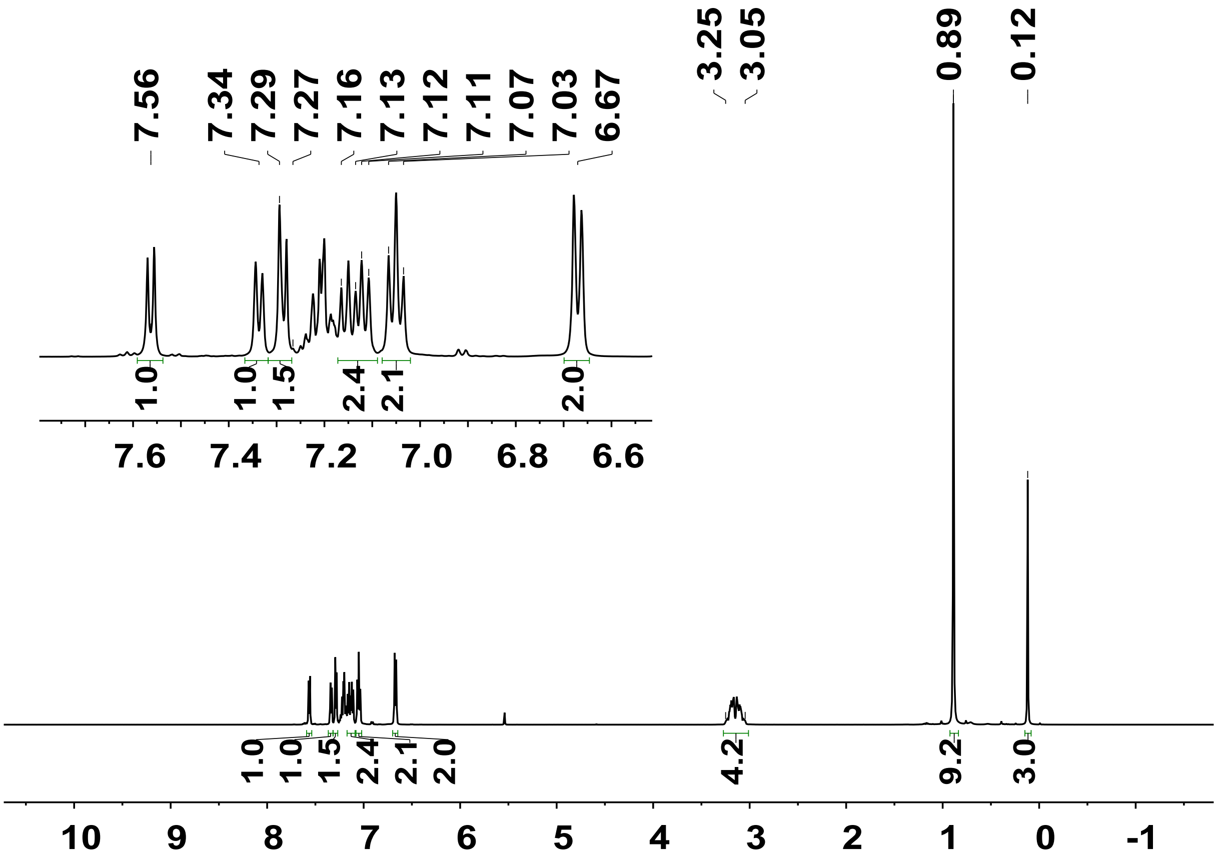

Figure S 54 – ^{1}H NMR spectrum (499.9 MHz, 305.1 K, C_6D_6) of phenylsulfanyl-stabilized methyl(*tert*-butyl)lsilyl borate **13d**$[B(C_6F_5)_4]$.

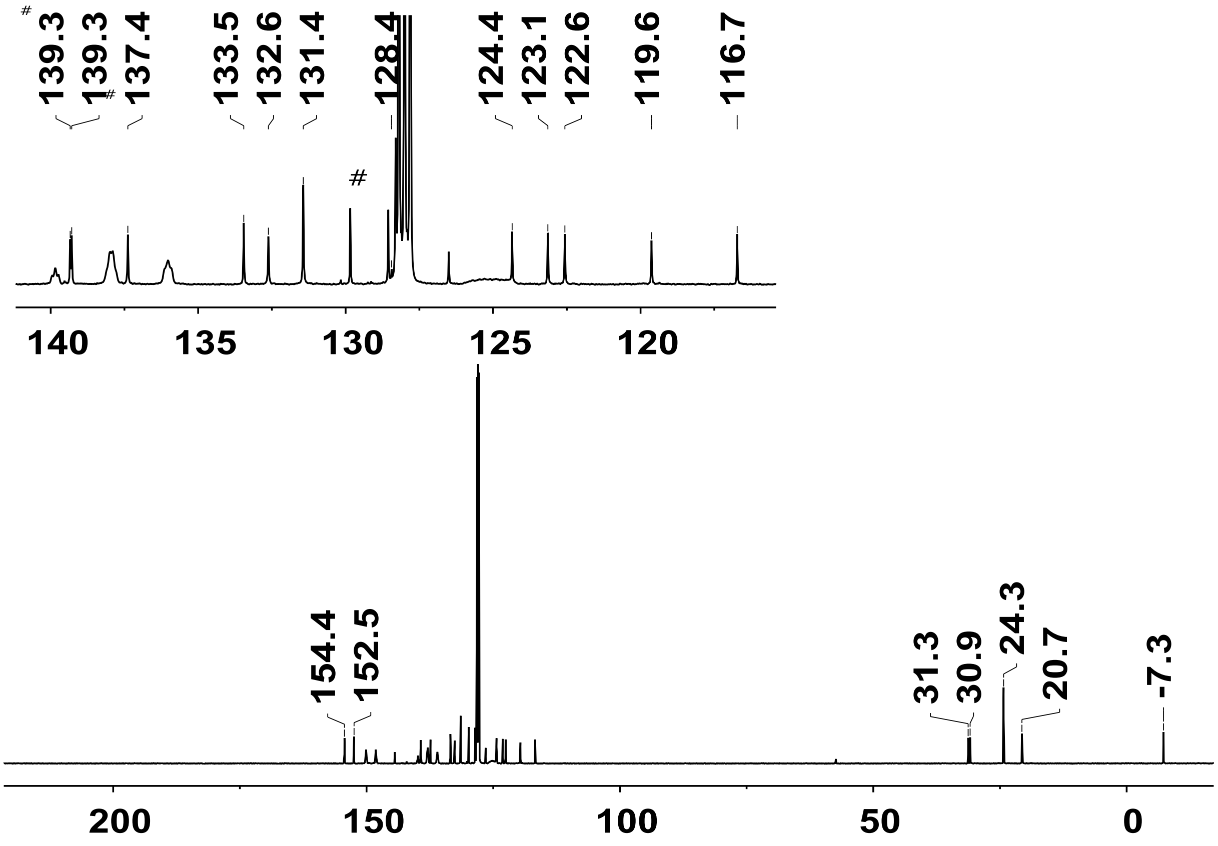

Figure S 55 – $^{13}C\{^{1}H\}$ NMR spectrum (125.7 MHz, 305.0 K, C_6D_6) of phenylsulfanyl-stabilized methyl(*tert*-butyl)silyl borate **13d**[$B(C_6F_5)_4$].

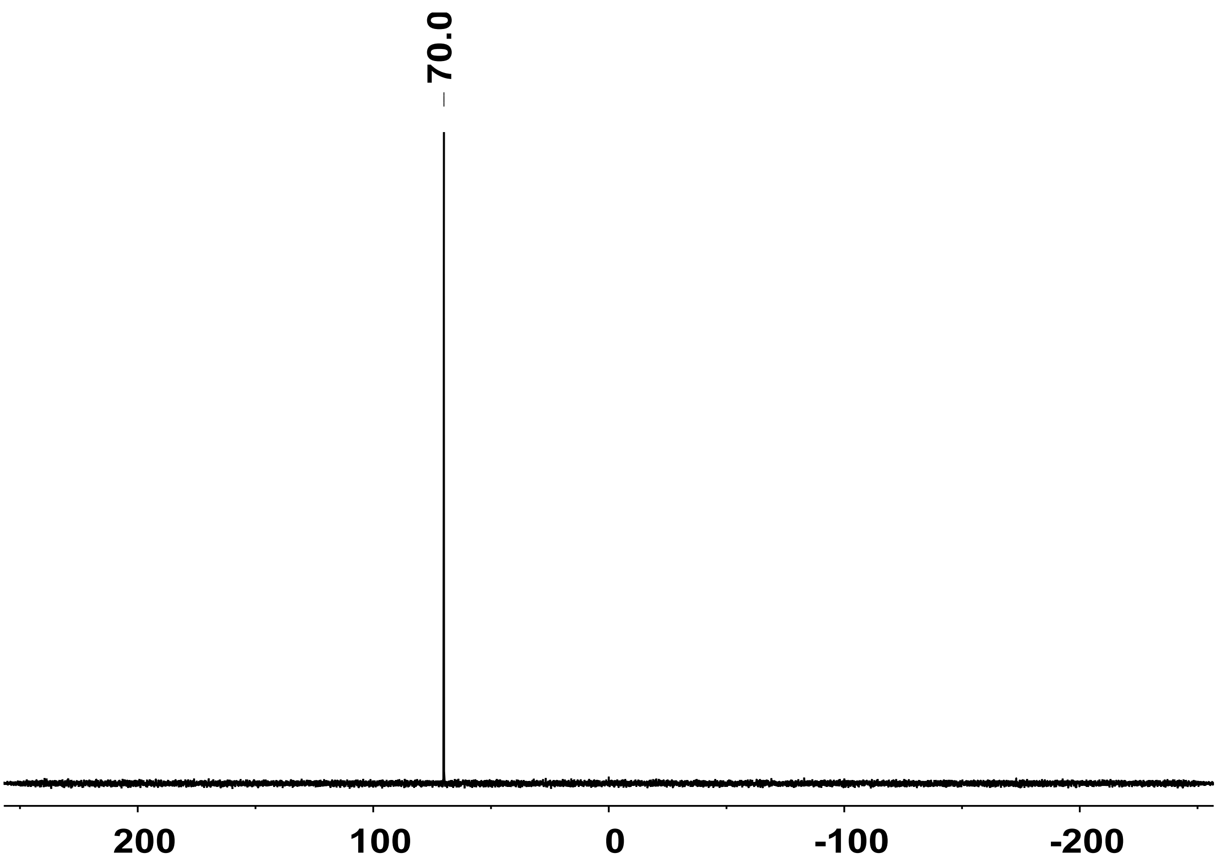

Figure S 56 – $^{29}Si\{^{1}H\}$ NMR spectrum (99.3 MHz, 305.0 K, C_6D_6) of phenylsulfanyl-stabilized methyl(*tert*-butyl)silyl borate **13d** [B(C_6F_5)$_4$].

Selanyl-Stabilized Methylphenylsilyl Borate 13e[B(C_6F_5)$_4$]

The title compound **13e**[B(C_6F_5)$_4$] was synthesized according to general procedure **D** using 1.0 equiv. (406 µmol, 174 mg) of 5-phenylselanyl-6-phenylmethylsilylacenaphthene **6** and 0.8 equiv. (325 µmol, 300 mg) of trityl borate.

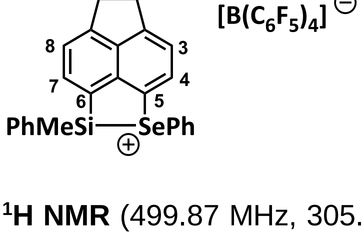

^{1}H NMR (499.87 MHz, 305.0 K, C_7D_8): δ = 0.40 (s, 1.8 H, *trans*-Me-H), 0.83 (s, 3 H, *cis*-Me-H), 3.05-3.14 (m, 7 H), 6.07 (d, 2 H, *cis*-*o*-Ph-H, ^{3}J = 7.3 Hz), 6.52-6.55 (m, 3 H), 6.71-6.74 (m, 1 H), 6.79-6.80 (m, 2 H), 6.82-6.85 (m, 2 H), 6.90-6.93 (m, 1 H), 6.96-7.08 (m, 4 H), 7.12-7.15 (m, 4 H), 7.18-7.20 (m, 2 H), 7.24-7.28 (m, 2 H), 7.31-7.36 (m, 2 H). **$^{13}C\{^{1}H\}$ NMR** (125.71 MHz, 305.0 K, C_7D_8): δ = -4.8 (CH_3, *trans*), -2.5 (CH_3, *cis*), 30.9 (CH_2), 31.3 (CH_2), 118.4 (C), 119.6 (C), 121.8 (C), 121.9 (C), 122.7 (CH), 122.8 (CH), 123.2 (CH), 123.2 (CH), 124.4 (C), 126.6 (CH), 126.9 (C), 128.6 (CH), 129.2 (CH), 129.7 (CH), 129.9 (CH), 130.8 (CH),130.9 (CH), 131.7 (CH), 132.1 (CH), 134.0 (CH), 134.3 (CH), 134.9 (CH), 135.0 (CH), 135.2 (CH),

135.6 (CH), 137.0 (dm, ^{1}J(C,F) = 236.8 Hz, [B(C_6F_5)$_4$]), 138.5 (CH), 138.7 (CH), 139.1 (dm, ^{1}J(C,F) = 235.4 Hz, [B(C_6F_5)$_4$]), 140.2 (C), 140.5 (C), 140.6 (C), 144.5 (C), 149.2 (dm, ^{1}J(C,F) = 242.7 Hz, [B(C_6F_5)$_4$]) 153.0 (C), 154.0 (C), 154.2 (C). (ipsp-C of [B(C_6F_5)$_4$] overlaps with the solvent signal). **^{29}Si{^{1}H} NMR** (99.31 MHz, 305.0 K, C_7D_8): δ = 49.7 ($J_{Si,Se}$ = 59.5 Hz, *trans*), 52.3 ($J_{Si,Se}$ = 59.1 Hz, *cis*). **^{77}Se{^{1}H} NMR** (95.36 MHz, 305.0 K, C_7D_8): δ = 260.3 (*cis*), 262.6 (*trans*). **^{11}B{^{1}H} NMR** (160.38 MHz, 305.0 K, C_7D_8): δ = -16.0. **^{19}F{^{1}H} NMR** (470.30 MHz, 305.0 K, C_7D_8): δ = -166.6-(-166.4) (m, 8F), -162.7 (t, J = 20.1, 4F), 131.8-(-131.5) (m, 8F).

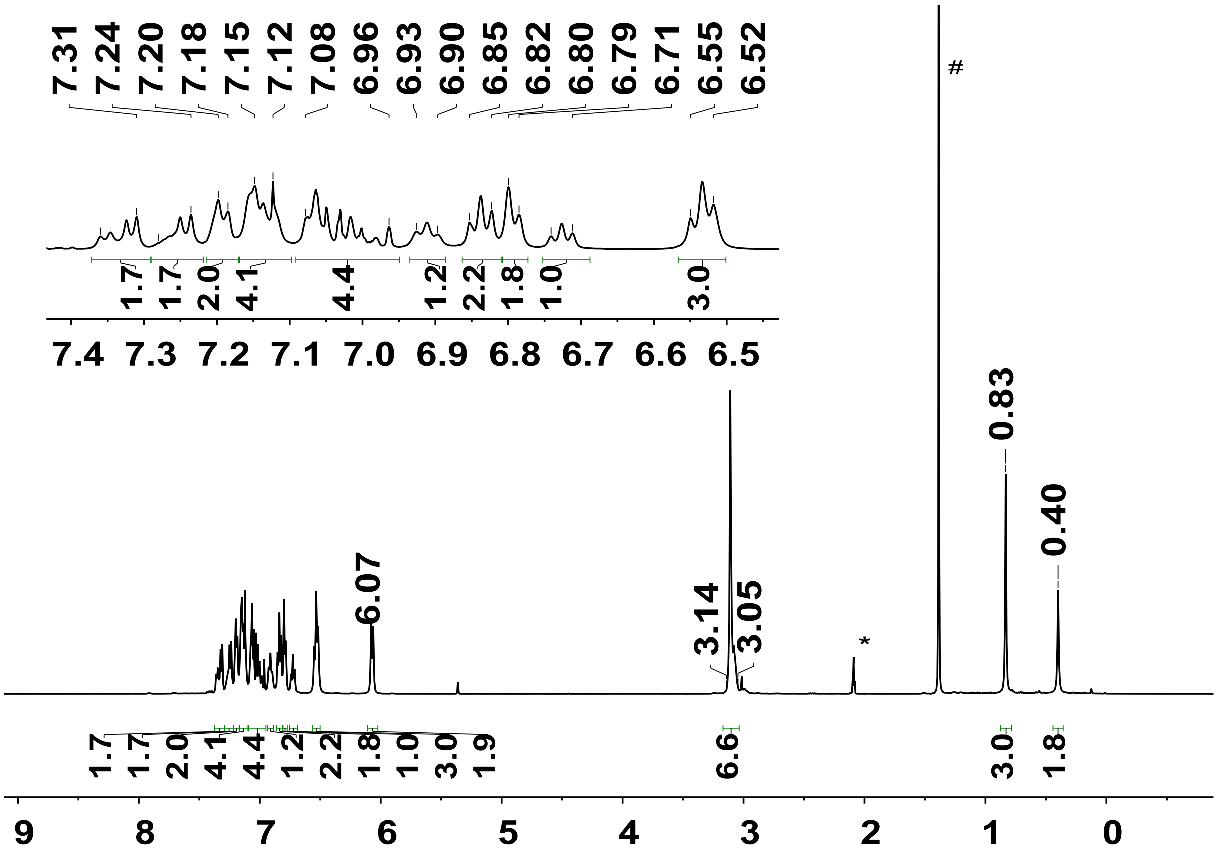

Figure S 57 – ^{1}H NMR spectrum (499.9 MHz, 305.1 K, C_7D_8) of phenylselanyl-stabilized methylphenylsilyl borate **13e**[B(C_6F_5)$_4$] (*$C_6D_5CHD_2$, #C_6H_{12}).

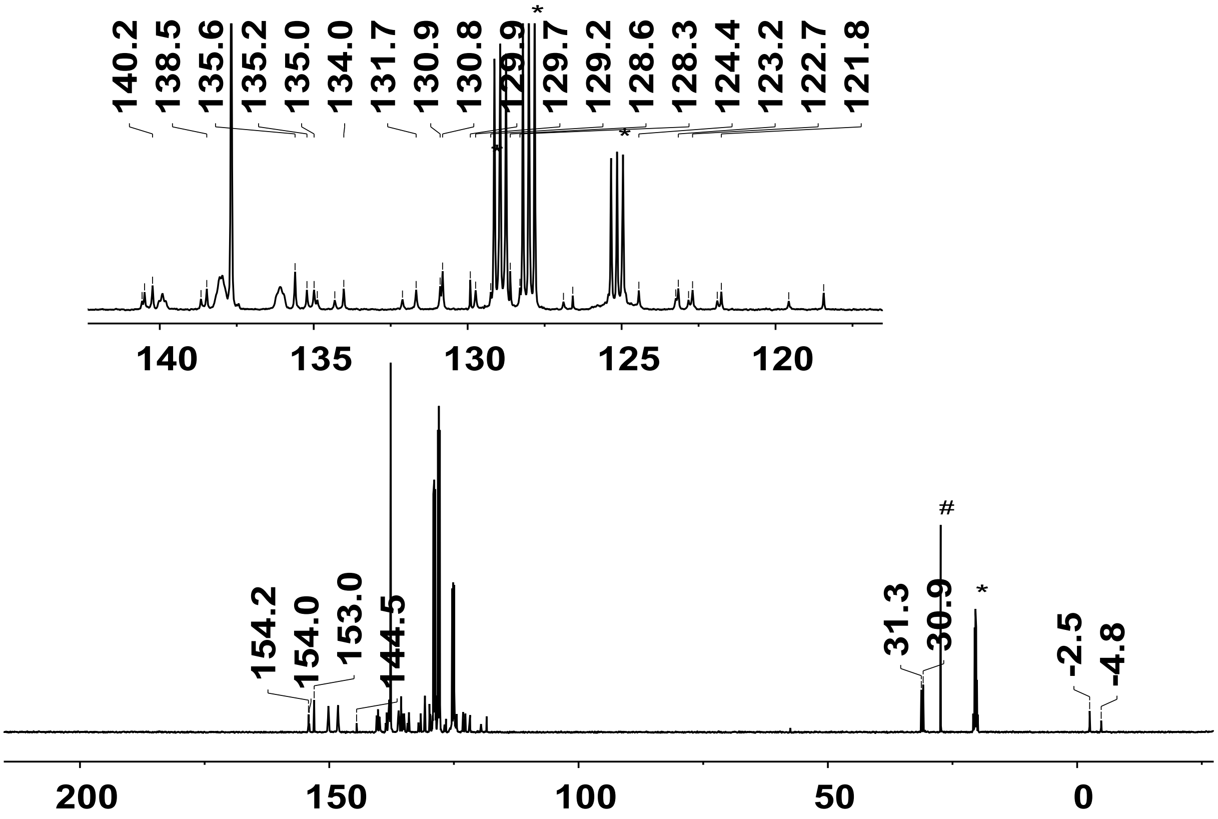

Figure S 58 – $^{13}C\{^1H\}$ NMR spectrum (125.7 MHz, 305.0 K, C_7D_8) of phenylselanyl-stabilized methylphenylsilyl borate **13e**[$B(C_6F_5)_4$] (*C_7D_8, #C_6H_{12}).

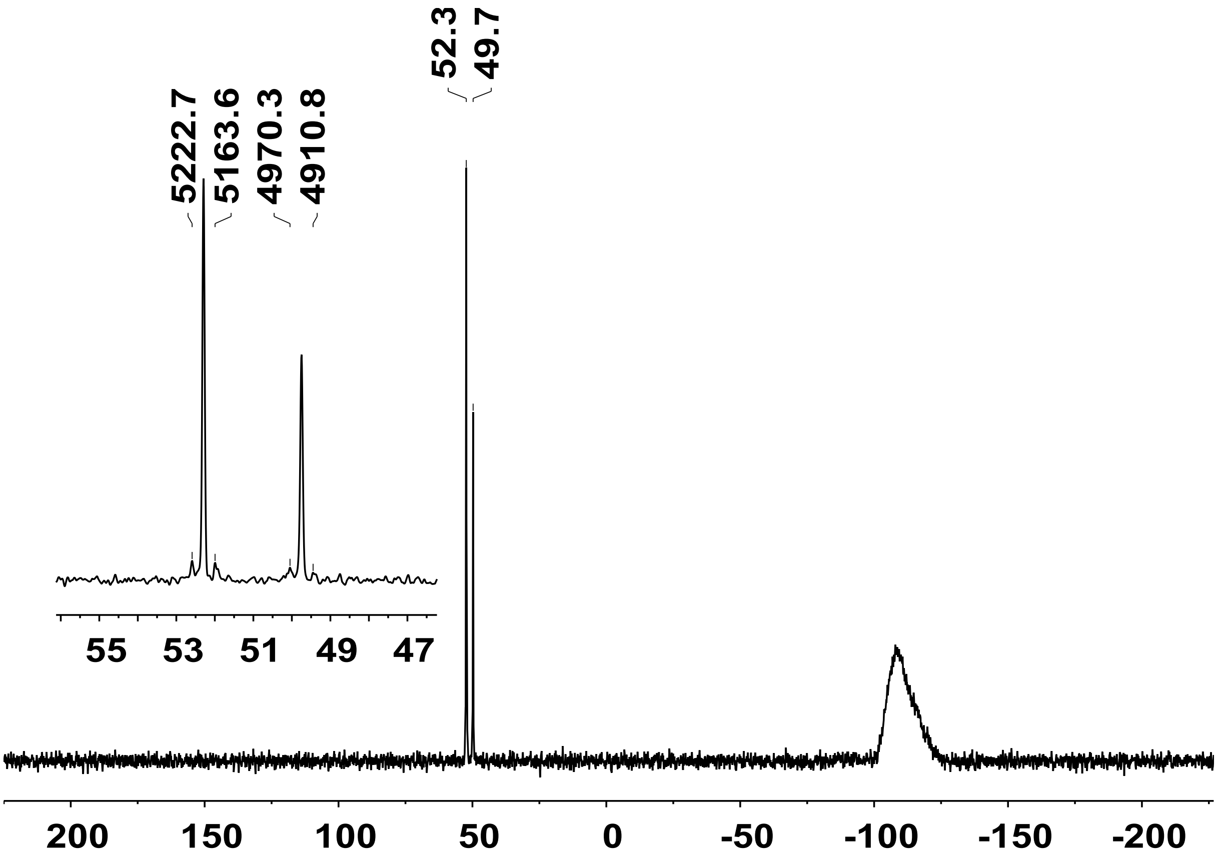

Figure S 59 – $^{29}Si\{^1H\}$ NMR spectrum (99.31 MHz, 305.0 K, C_7D_8) of phenylselanyl-stabilized methylphenylsilyl borate **13e**[$B(C_6F_5)_4$].

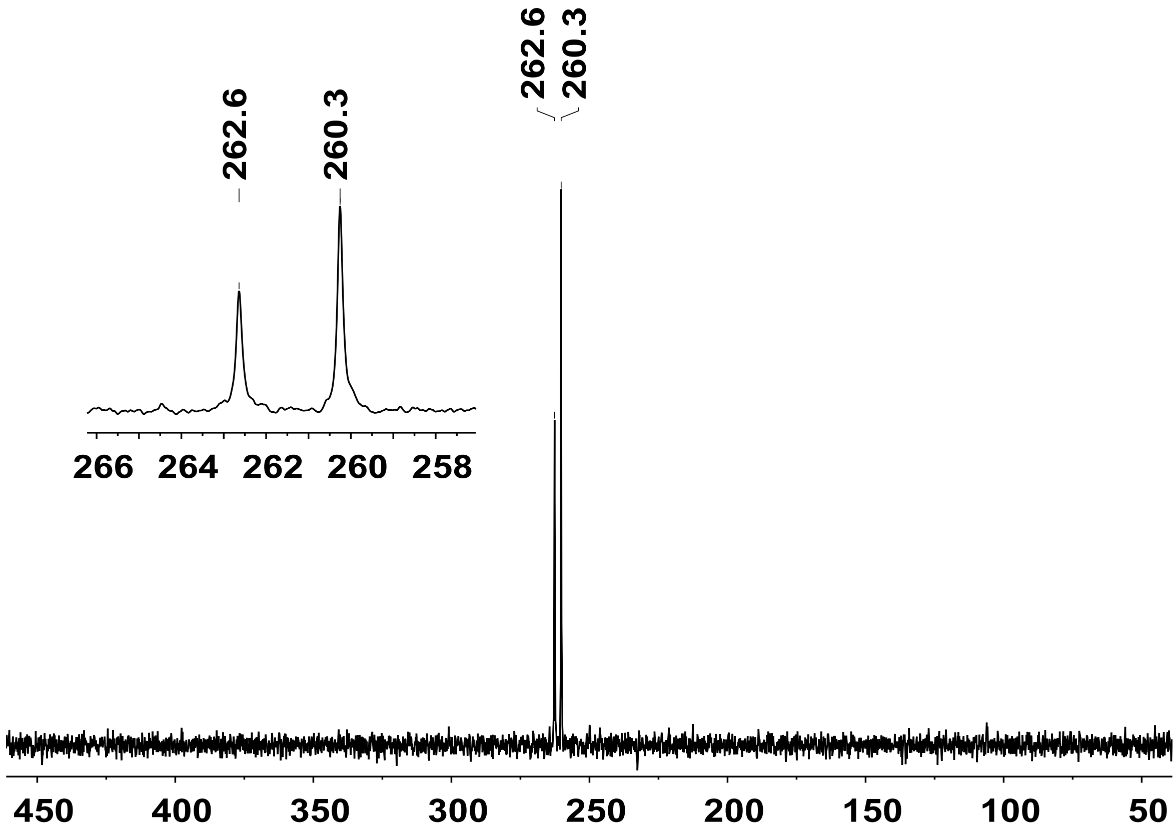

Figure S 60 – $^{77}Se\{^1H\}$ NMR spectrum (95.36 MHz, 305.0 K, C_7D_8) of phenylselanyl-stabilized methylphenylsilyl borate **13e**[B(C_6F_5)$_4$].

Selanyl-Stabilized Methylphenylsilyl Borate 14b[B(C_6F_5)$_4$]

The title compound **14b**[B(C_6F_5)$_4$] was synthesized according to general procedure **D** using 1.0 equiv. (446 µmol, 180 mg) of 1-phenylselanyl-8-phenylmethylsilylnaphthalene **10a** and 0.8 equiv. (357 µmol, 329 mg) of trityl borate.

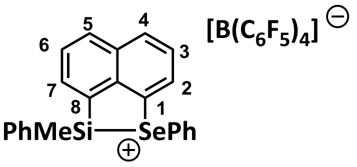

^{1}H NMR (499.87 MHz, 305.0 K, C_6D_6): δ = 0.37 (s, 2H, Si-CH_3, *trans*), 0.77 (s, 3H, Si-CH_3, *cis*), 6.03 (d, 2H, ^3J = 7.9 Hz, Se-Ph-*o*-H, *cis*), 6.44 (d, 1.4H, ^3J = 7.9 Hz, Se-Ph-*o*-H, *trans*), 6.56 (t, 2H, ^3J = 7.4 Hz, Se-Ph-*m*-H, *cis*), 6.72-6.80 (m, 3H, *cis*), 6.85-6.93 (m, 4H), 7.03 - 7.14 (m, 5H), 7.25 - 7.33 (m, 5H), 7.40 (t, ^3J = 7.5 Hz, 2H), 7.80-7.85 (m, 4H). **$^{13}C\{^1H\}$ NMR** (125.71 MHz, 305.0 K, C_6D_6): δ = -5.6 (CH_3, *trans*), -3.1 (CH_3, *cis*), 123.4 (C), 124.1-126.1 (brm, [B(C_6F_5)$_4$]), 124.3 (C), 124.7 (C), 125.9 (C), 126.5 (CH), 127.9 (CH), 128.3 (CH), 128.4 (CH), 128.7 (CH), 128.9 (CH), 129.6 (CH), 129.8 (CH), 130.8 (CH), 130.9 (CH), 131.6 (CH), 132.0 (C), 132.1 (CH), 132.8 (CH), 132.9 (CH), 133.8 (CH), 134.0 (CH), 134.3 (CH), 134.7 (C), 134.9 (CH), 135.5 (CH), 136.9 (CH), 136.9 (dm, $^1J_{C,F}$ = 234.6 Hz, [B(C_6F_5)$_4$]), 137.0 (CH),

138.9 (dm, $^{1}J_{C,F}$ = 136.4 Hz, [B(C_6F_5)$_4$]), 139.9 (C), 140.2 (C), 149.1 (d, $^{1}J_{C,F}$ = 239.8 Hz, [B(C_6F_5)$_4$]). **^{29}Si{^{1}H} NMR** (99.31 MHz, 305.0 K, C_6D_6): δ = 41.4 (*trans*), 43.7 (*cis*). **^{29}Si{^{1}H} INEPT NMR** (99.31 MHz, 305.0 K, C_6D_6, D_3 = 0.0112 s, D_4 = 0.0313 s): δ = 41.4, 43.7 ($J_{Si,Se}$ = 61 Hz). **^{77}Se{^{1}H} NMR** (95.36 MHz, 305.0 K C_6D_6): δ = 254.7 (*cis*), 256.1 (*trans*).

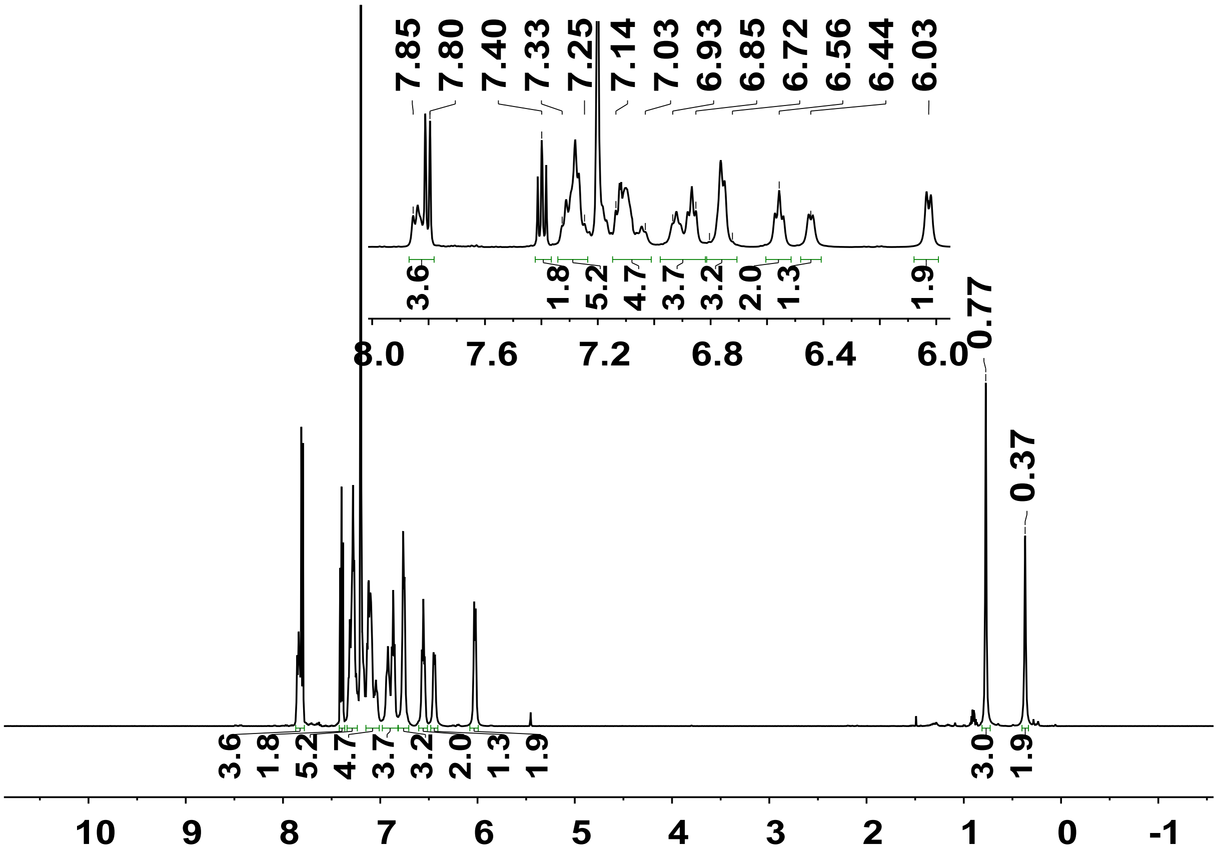

Figure S 61 – ^{1}H NMR spectrum (499.87 MHz, 305.0 K, C_6D_6) of phenylselanyl-stabilized methylphenylsilyl borate **14b**[B(C_6F_5)$_4$].

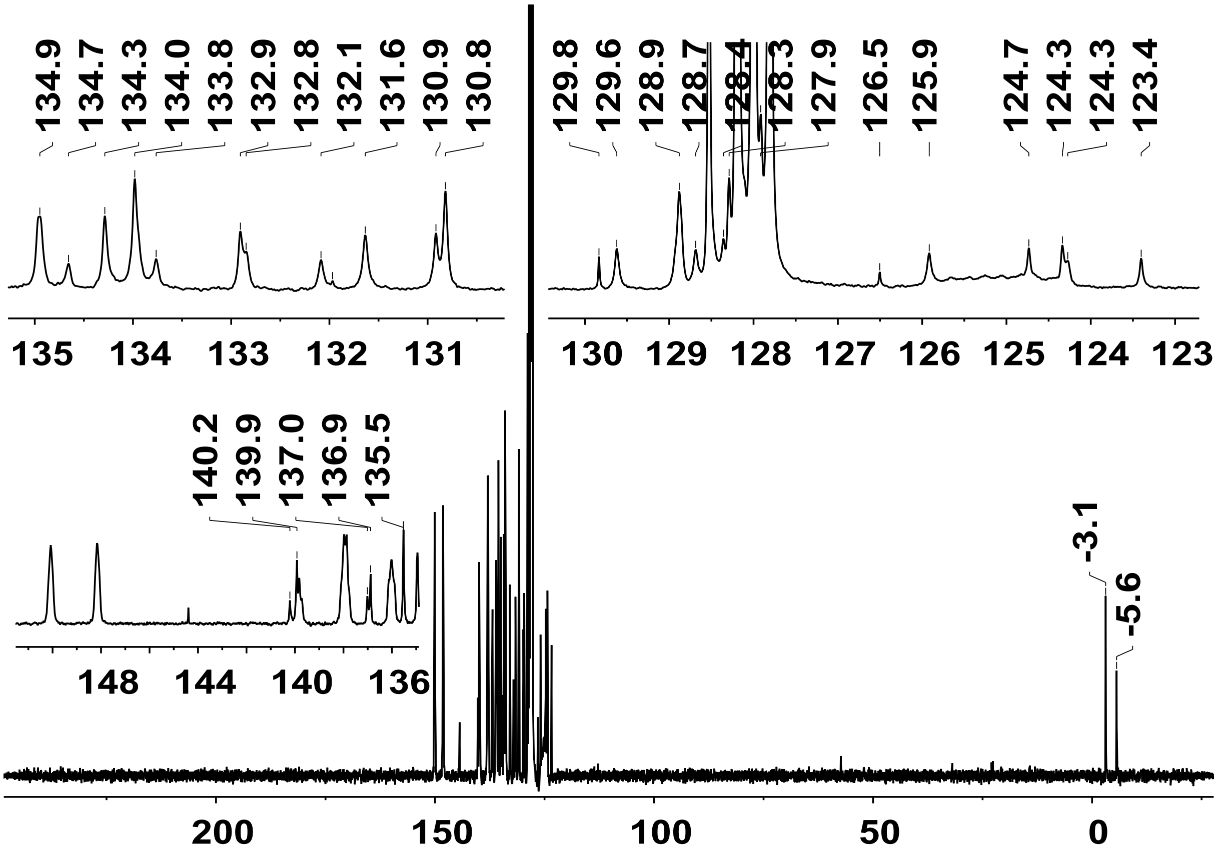

Figure S 62 – $^{13}C\{^{1}H\}$ NMR spectrum (125.71 MHz, 305.0 K, C_6D_6) of phenylselanyl-stabilized methylphenylsilyl borate **14b**[$B(C_6F_5)_4$].

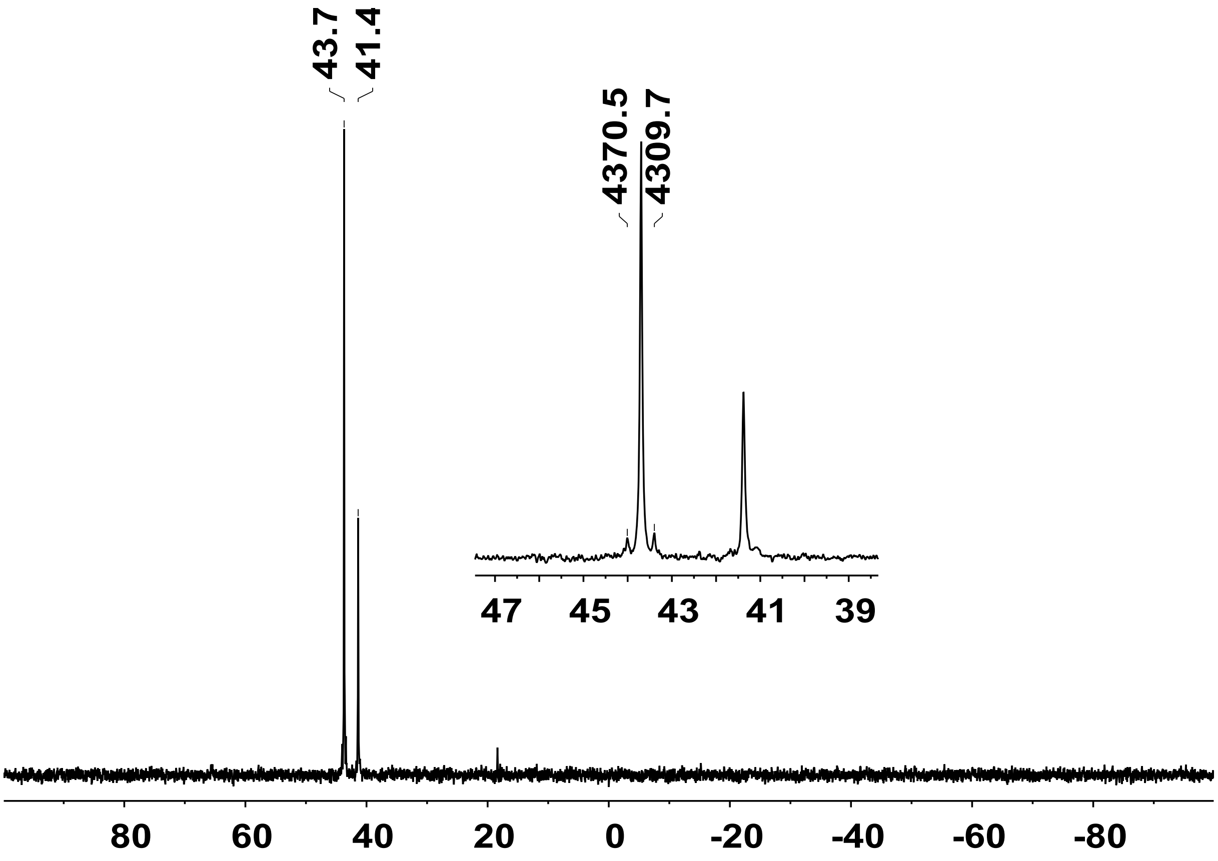

Figure S 63 – ^{29}Si{^{1}H} INEPT NMR spectrum (99.31 MHz, 305.0 K, C_6D_6, D_3 = 0.0112 s, D_4 = 0.0313 s) of phenylselanyl-stabilized methylphenylsilyl borate **14b**[B(C_6F_5)$_4$].

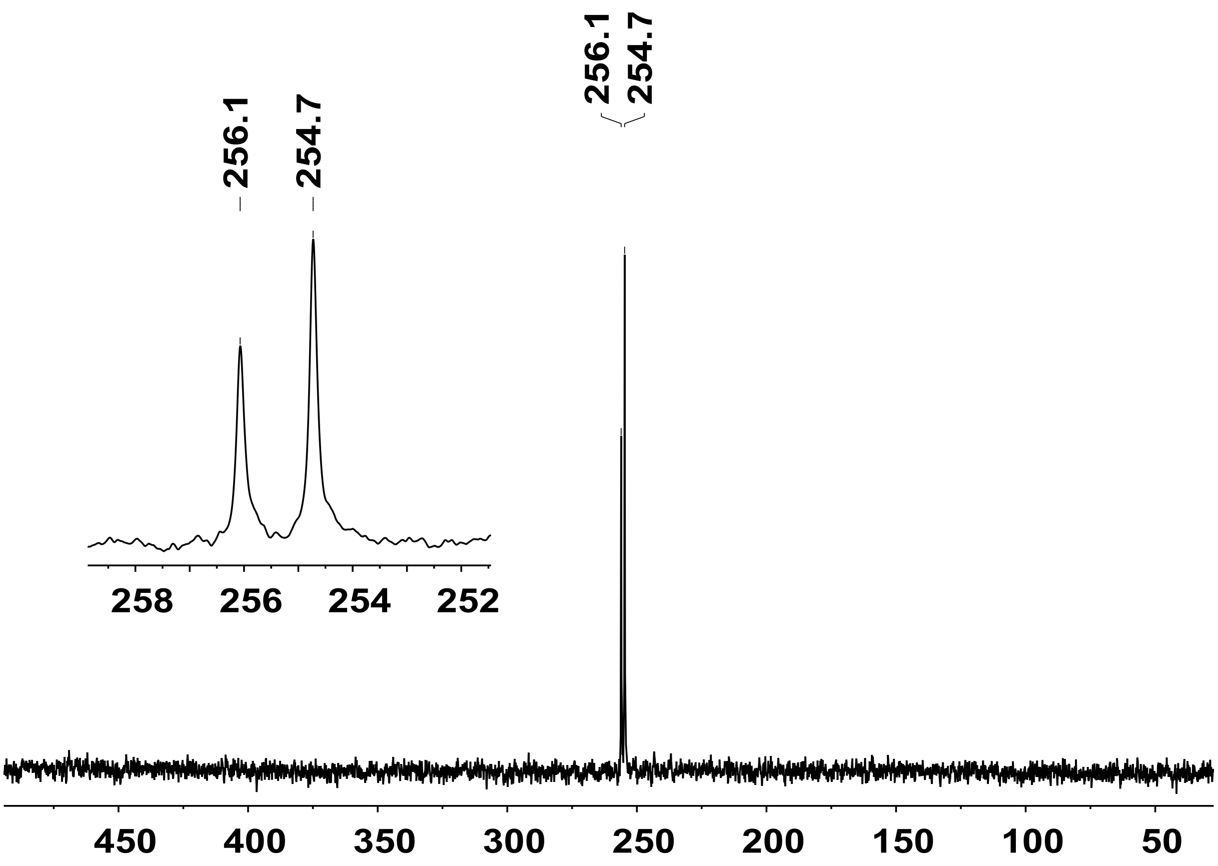

Figure S 64 – ^{77}Se{^{1}H} NMR spectrum (95.36 MHz, 305.0 K C_6D_6) of phenylselanyl-stabilized methylphenylsilyl borate **14b**[B(C_6F_5)$_4$].

Selanyl-Stabilized *tert*-butylphenylsilyl Borate 14c[B(C_6F_5)$_4$]

The title compound **14c**[B(C_6F_5)$_4$] was synthesized according to general procedure **D** using 1.0 equiv. (443 µmol, 170 mg) of 1-phenylselanyl-8-*tert*-butylmethylsilylnaphthalene **10b** and 0.8 equiv. (355 µmol, 327 mg) of trityl borate.

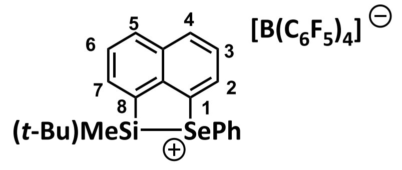

^{1}H NMR (499.87 MHz, 304.9 K, C_6D_6): δ = 0.06 (s, 3H, Si-CH$_3$, *trans*), 0.37 (s, Si-CH$_3$, *cis*), 0.59 (s, Si-C(C$\underline{H}_3$)$_3$, *cis*), 0.73 (s, 9H, Si-C(C$\underline{H}_3$)$_3$, *trans*), 6.40 - 6.42 (m, 2H, 2'-H), 6.87 (t, 2H, ^{3}J = 7.9 Hz, 3'-H), 6.98 - 7.01 (m, 1H, 4'-H), 7.19 - 7.22 (m, 1H, 3-H), 7.24-7.26 (m, 1H), 7.33 - 7.39 (m, 2H), 7.71-7.74 (m, 2H, 4-H, 6-H). **^{13}C{^{1}H} NMR** (125.71 MHz, 305.0 K C_6D_6): δ = -7.6 (CH$_3$, Si-$\underline{C}$H$_3$), 20.8 (C, Si-$\underline{C}$(CH$_3$)$_3$), 24.7 (CH$_3$, Si-C($\underline{C}$H$_3$)$_3$), 123.1 (C, C-1'), 124.3-125.9 (brm, [B(C_6F_5)$_4$]), 125.1 (C), 128.3 (CH, C-3), 128.5 (CH, C-2'), 128.7 (CH), 131.7 (CH, C-3'), 132.1 (CH, C-4'), 132.6 (CH, C-6), 133.7 (CH), 133.9 (CH, C-4), 134.7 (C, C-10), 136.2 (CH, C-7), 137.0 (dm, $^{1}J_{C,F}$ = 240.2 Hz, [B(C_6F_5)$_4$]), 138.9 (dm, $^{1}J_{C,F}$ = 240.2 Hz, [B(C_6F_5)$_4$]), 139.9 (CH, C-9), 149.1 (d, $^{1}J_{C,F}$ = 241.0 Hz, [B(C_6F_5)$_4$]). One C missing due to overlap. **^{29}Si{^{1}H}**

NMR (99.31 MHz, 305.0 K, C_6D_6): δ = 54.3 (*cis*), 62.9 (*trans*). **^{29}Si{^{1}H} INEPT NMR** (99.31 MHz, 305.0 K, C_6D_6, D_3 = 0.0112 s, D_4 = 0.0313 s) δ = 62.9 ($J_{Si,Se}$ = 67.6 Hz). **^{77}Se{^{1}H} NMR** (95.36 MHz, 305.0 K, C_6D_6): δ = 232.0 ($J_{Si,Se}$ = 67.6 Hz, *trans*), 251.7 (*cis*).

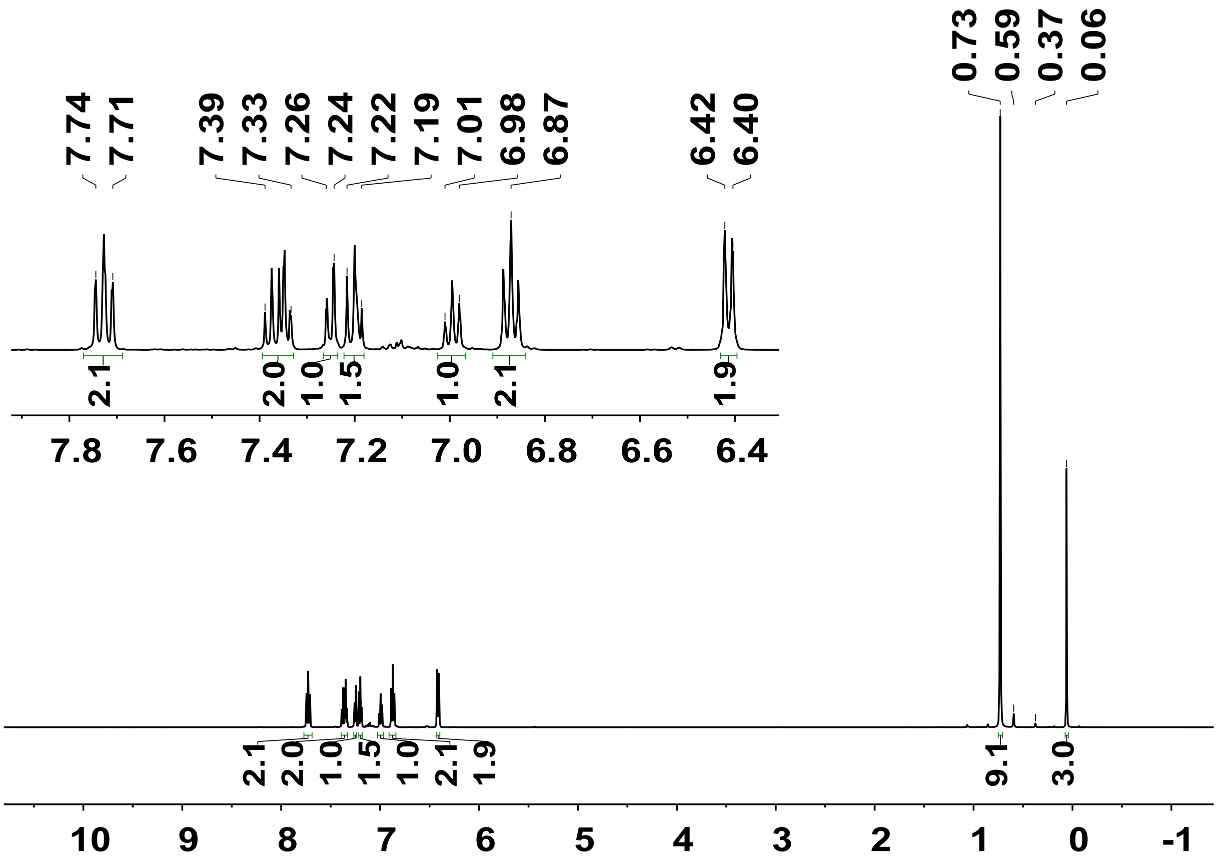

Figure S 65 – ^1H NMR spectrum (499.87 MHz, 304.9 K, C_6D_6) of phenylselanyl-stabilized *tert*-butylmethylsilyl borate **14c**[$B(C_6F_5)_4$].

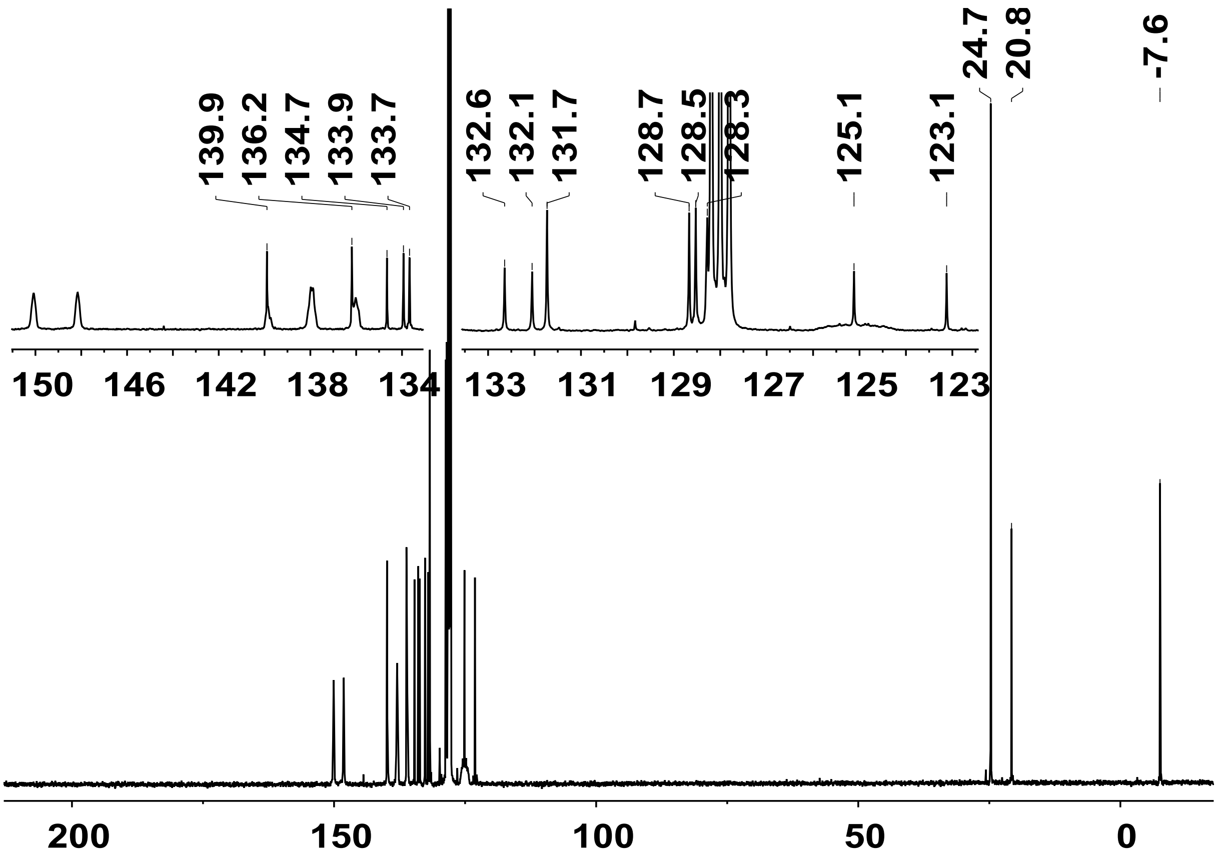

Figure S 66 – $^{13}C\{^{1}H\}$ NMR spectrum (125.71 MHz, 305.0 K C_6D_6) of phenylselanyl-stabilized *tert*-butylmethylsilyl borate **14c**[B(C_6F_5)$_4$].

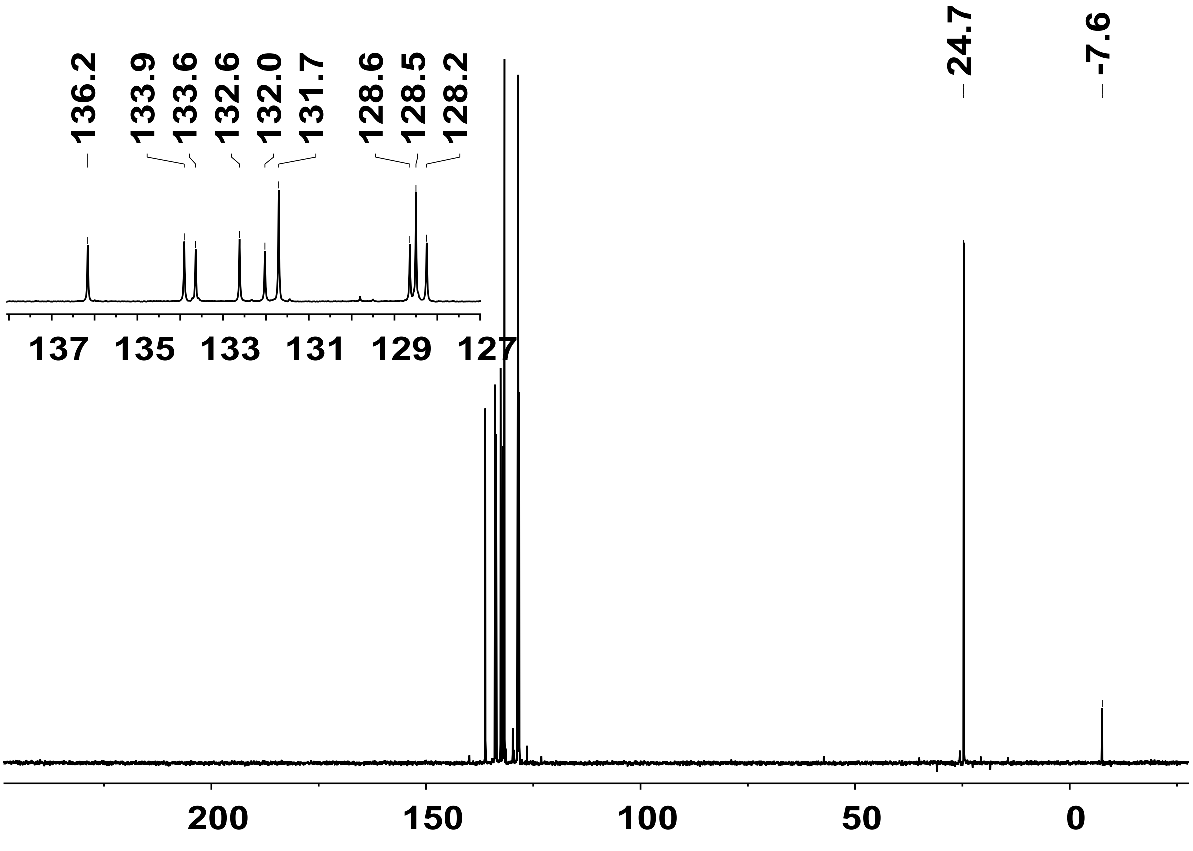

Figure S 67 – $^{13}C\{^{1}H\}$ DEPT NMR spectrum (125.71 MHz, 305.0 K C_6D_6) of phenylselanyl-stabilized *tert*-butylmethylsilyl borate **14c**[B(C_6F_5)$_4$].

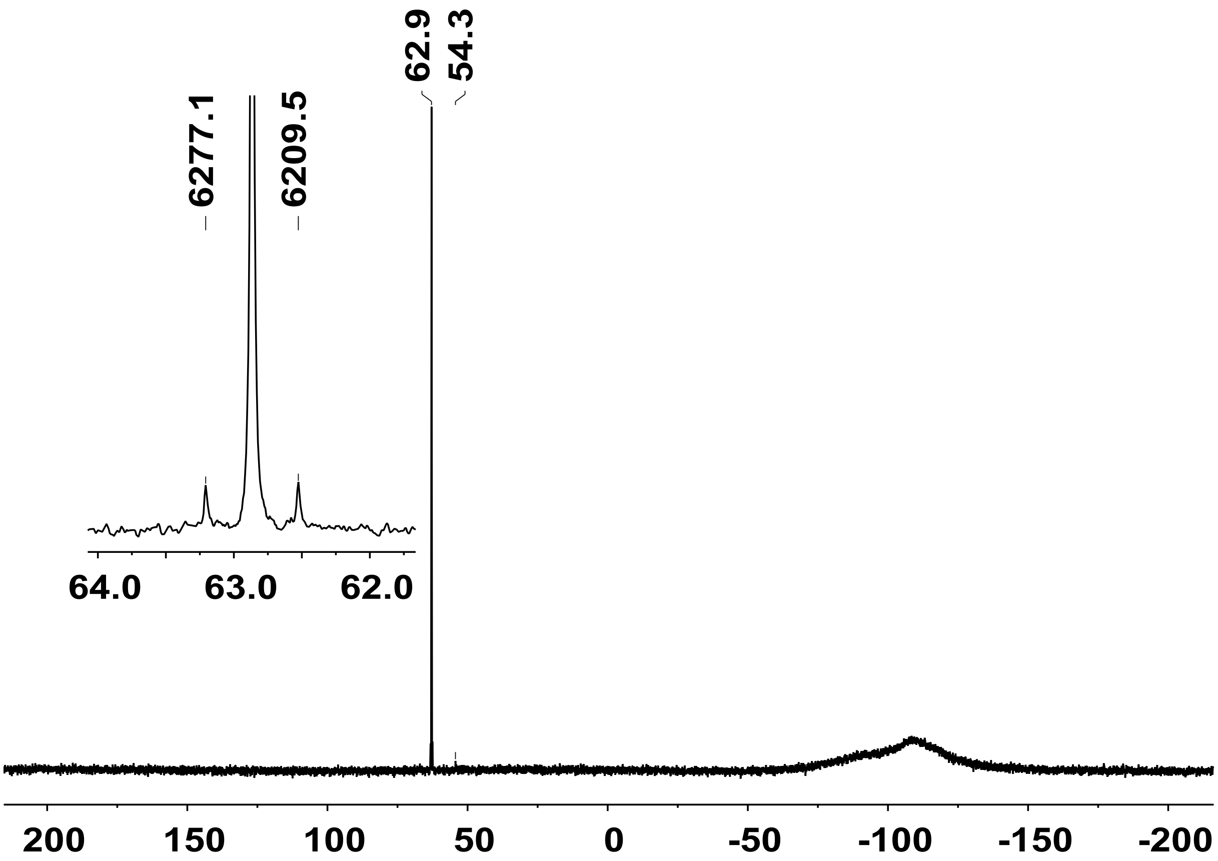

Figure S 68 – $^{29}Si\{^{1}H\}$ NMR spectrum (99.31 MHz, 305.0 K, C_6D_6) of phenylselanyl-stabilized *tert*-butylmethylsilyl borate **14c**[B(C_6F_5)$_4$].

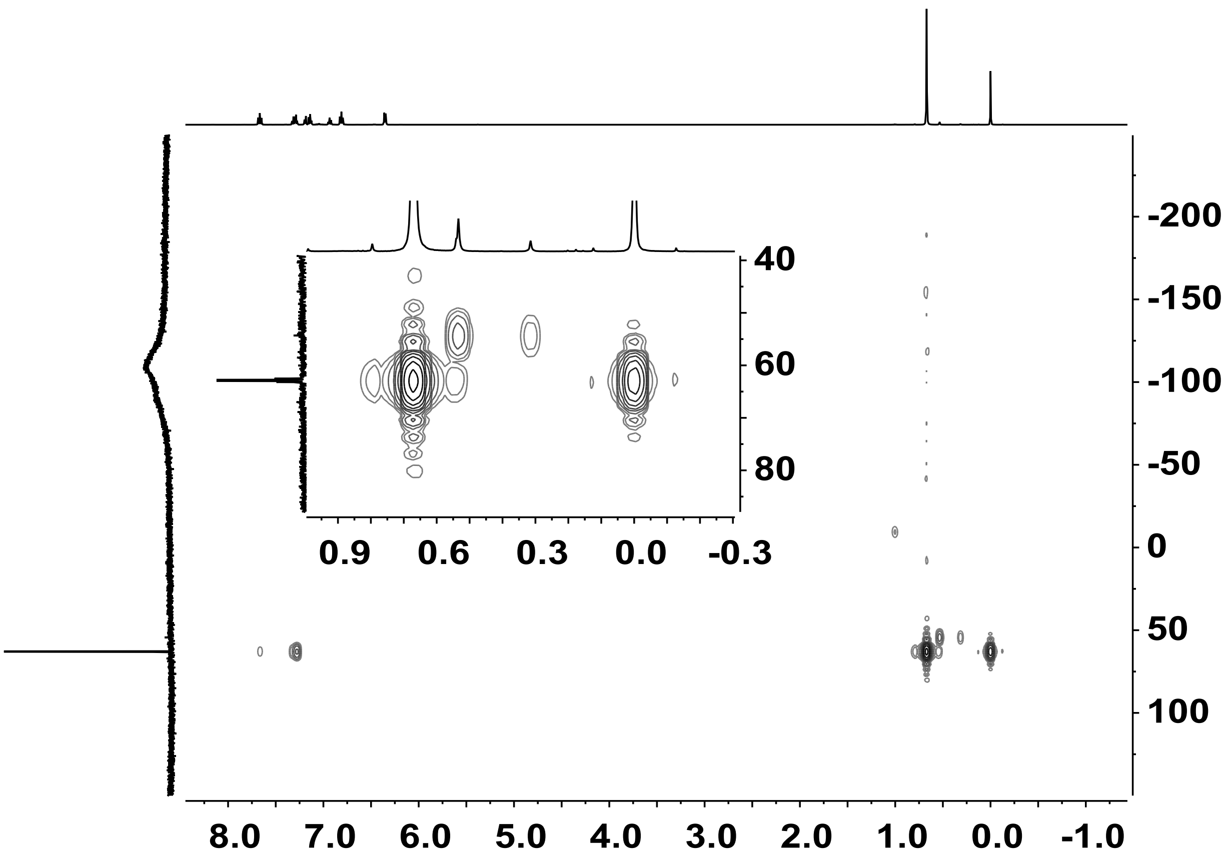

Figure S 69 – $^{1}H/^{29}Si$ HMBC NMR spectrum (499.87 MHz, 304.9 K, C_6D_6) of phenylselanyl-stabilized *tert*-butylmethylsilyl borate **14c**[B(C_6F_5)$_4$].

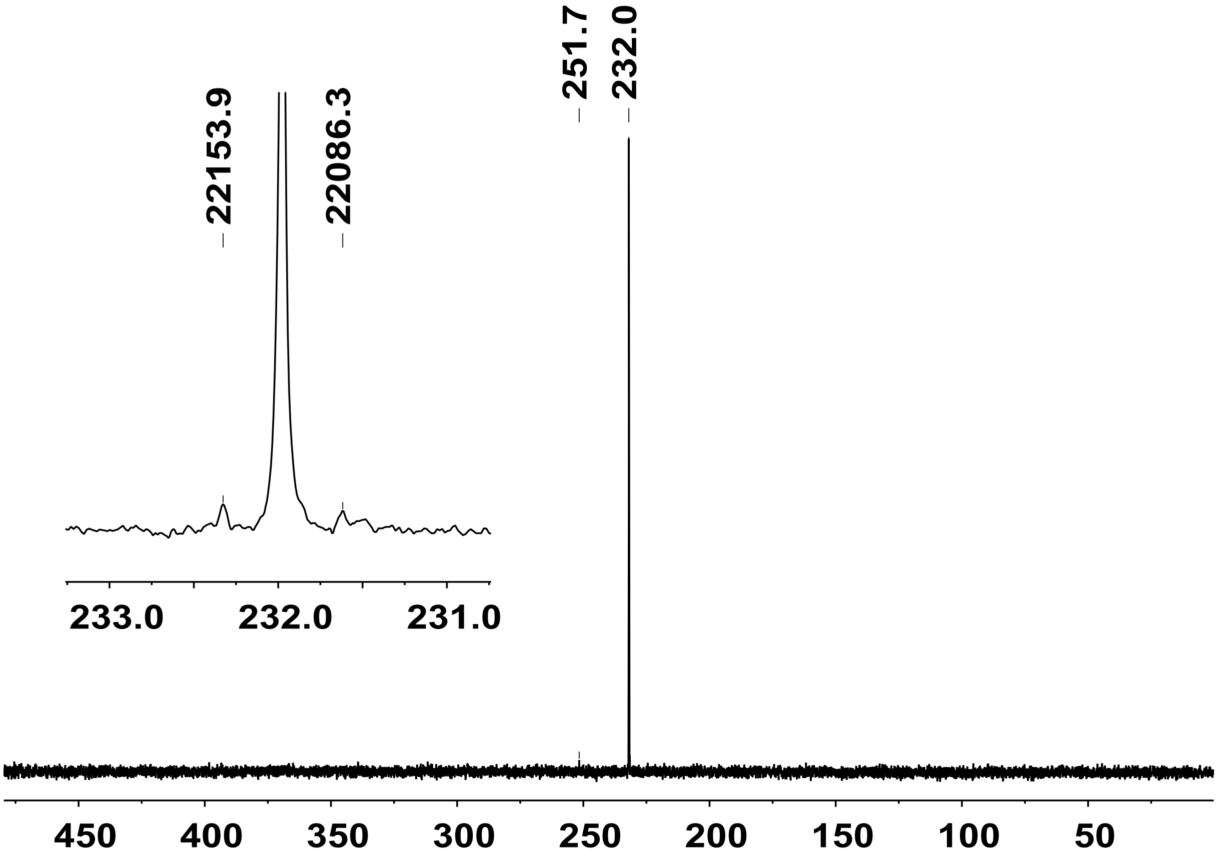

Figure S 70 – $^{77}Se\{^1H\}$ NMR spectrum (95.36 MHz, 305.0 K C_6D_6) of phenylselanyl-stabilized *tert*-butylmethylsilyl borate **14c**[$B(C_6F_5)_4$].

Tellanyl-Stabilized Methylphenylsilyl Borate 13f[$B(C_6F_5)_4$]

The title compound **13f**[$B(C_6F_5)_4$] was synthesized according to general procedure **D** using 1.2 equiv. (442 µmol, 230 mg) of 5-mesityltellanyl-6-phenylmethylsilylacenaphthene **7** and 1.0 equiv. (363 µmol, 335 mg) of trityl borate.

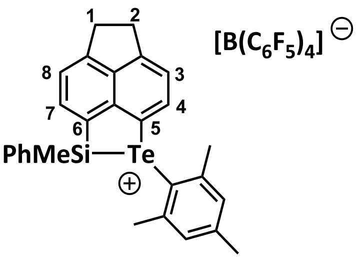

^{1}H NMR (499.87 MHz, 305.0 K, C_6D_6): δ = 0.62 (s, 3 H, $SiCH_3$, *trans*), 0.88 (s, 3 H, $SiCH_3$, $^3J_{H,Te}$ = 17.8 Hz, *cis*), 1.50 (s, 5 H, *o*-CH_3, *cis*), 1.70 (s, 6 H, *o*-CH_3, *trans*), 1.88 (s, 3 H, *p*-CH_3, *cis*), 2.06 (s, 3 H, *p*-CH_3, *trans*), 3.05–3.10 (m, 8 H, 4 × CH_2, H–1, H–2, *cis*/*trans*), 6.19 (s, 2 H, H–19, H–19´, *cis*), 6.60 (s, 2 H, H–19, H–19´, *trans*), 6.83–6.86 (m, 2 H, H–14, H–14´, *cis*), 7.02–7.05 (m, 1 H), 7.09–7.16 (m, 6 H, H–14, H–14´, *trans*), 7.21–7.25 (m, 3 H), 7.26–7.32 (m, 4 H), 7.36 (d, 1 H, $^3J_{H,H}$ = 7.0 Hz, H–7, *trans*), 7.45 (d, 1 H, $^3J_{H,H}$ = 7.3 Hz, H–4, *cis*). **$^{13}C\{^1H\}$ NMR** (125.71 MHz, 305.0 K, C_6D_6): δ = -2.7 (CH_3, $SiCH_3$, *trans*), -1.4 (CH_3, $SiCH_3$, *cis*), 19.9, 20.3 (2 × CH_3, *p*-CH_3), 23.8 (2 × CH_3, *o*-CH_3, *cis*), 24.4 (2 × CH_3, *o*-CH_3, *trans*), 30.5, 30.6,

30.7, 30.7 (4 × CH_2, C–1, C–2, *cis*/*trans*), 113.5, 113.9 (C), 114.6 (C, 2 × C, C–17, *cis*/*trans*), 122.1 (C), 122.3 (CH), 122.6 (CH), 122.6 (CH), 124.1–126.1 (m, C_{ipso}, $[B(C_6F_5)_4]^-$), 127.3 (C), 127.9 (C), 128.1 (C), 128.3 (CH), 128.4 (CH), 128.5 (CH), 128.6, 129.0 (CH), 129.6 (CH), 129.8, 131.4 (CH, C–19,C–19´, *trans*), 131.8 (CH, C–19,C–19´, *cis*), 133.0 (CH), 133.6 (CH), 134.9 (CH), 134.9 (CH), 135.4 (CH), 137.9 (tm, CF, $[B(C_6F_5)_4]^-$), 138.5 (CH), 138.6 (CH), 139.3 (CH, C–7, *trans*), 139.8 (CH, C–4, *cis*), 140.7 (C), 140.7 (C), 141.7, 141.9 (2 × C, C–20, *cis*/*trans*), 142.9, 143.2, 143.3, 143.4 (4 × C, C–18,C–18´, *cis*/*trans*), 149.2 (d, CF, $^1J_{C,F}$ = 241 Hz, $[B(C_6F_5)_4]^-$), 152.9, 153.1 (2 × C, C–9, *cis*/*trans*), 153.7, 153.9 (2 × C, C–10, *cis*/*trans*). **^{29}Si{^{1}H} NMR** (99.31 MHz, 305.0 K, C_6D_6): δ = 36.4 (*trans*), 40.7 (*cis*). **^{29}Si{^{1}H} INEPT NMR** (99.31 MHz, 305.0 K, C_6D_6): δ = 36.4 ($^1J_{Si,Te}$ = 171 Hz, *trans*), 40.7 ($^1J_{Si,Te}$ = 163 Hz, *cis*). **^{125}Te{^{1}H} NMR** (157.74 MHz, 305.0 K, C_6D_6): δ = 234.4 ($^1J_{Te,Si}$ = 171 Hz, *trans*), 239.7 ($^1J_{Te,Si}$ = 163 Hz, *cis*). **^{11}B{^{1}H} NMR** (160.38 MHz, 305.0 K, C_6D_6): δ = -15.9 ($[B(C_6F_5)_4]^-$). **^{19}F{^{1}H} NMR** (470.30 MHz, 305.0 K, C_6D_6): δ = -166.90–(-165.92) (m, 16 F, $[B(C_6F_5)_4]^-$), -162.87–(-162.23) (m, 8 F, $[B(C_6F_5)_4]^-$), -131.68 (brs, 16 F, $[B(C_6F_5)_4]^-$).

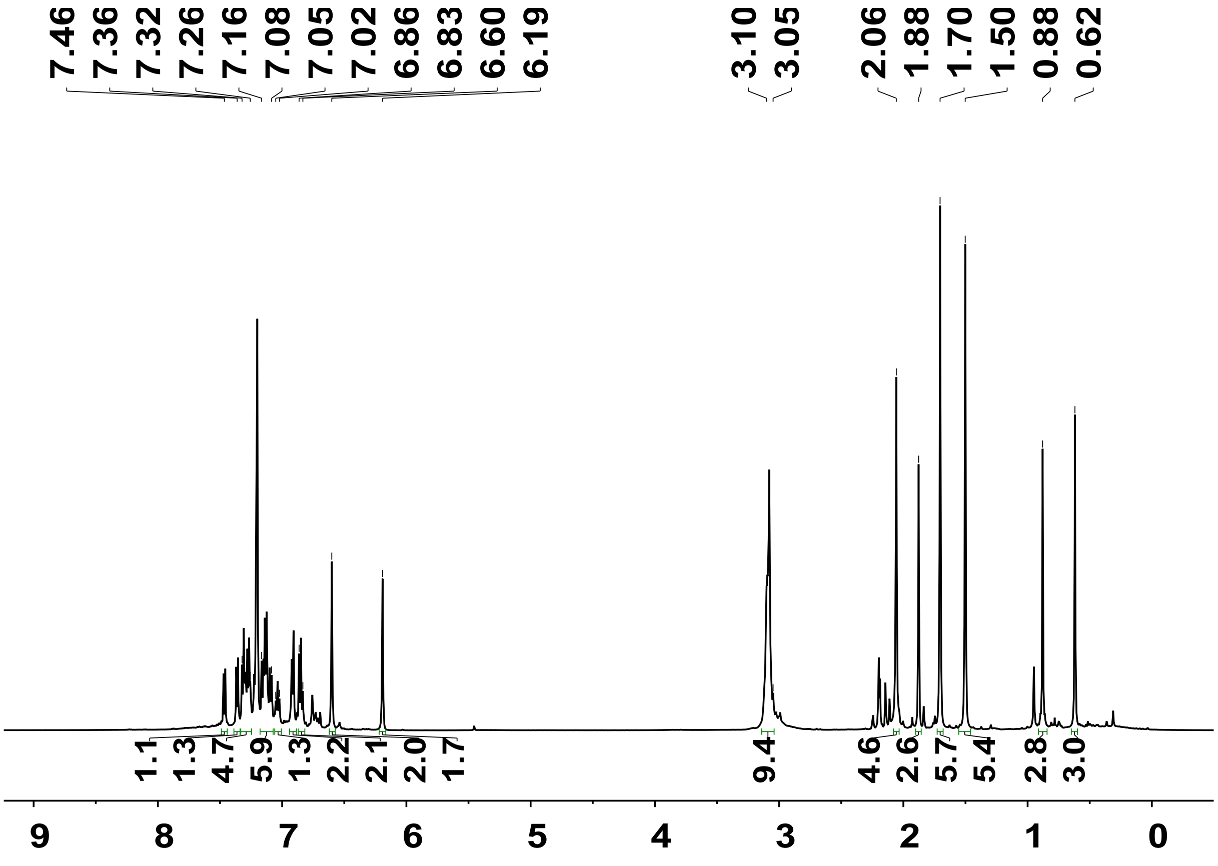

Figure S 71 – ^{1}H NMR spectrum (499.87 MHz, 305.0 K, C_6D_6) of mesityltellanyl-stabilized phenylmethylsilyl borate **13f**$[B(C_6F_5)_4]$.

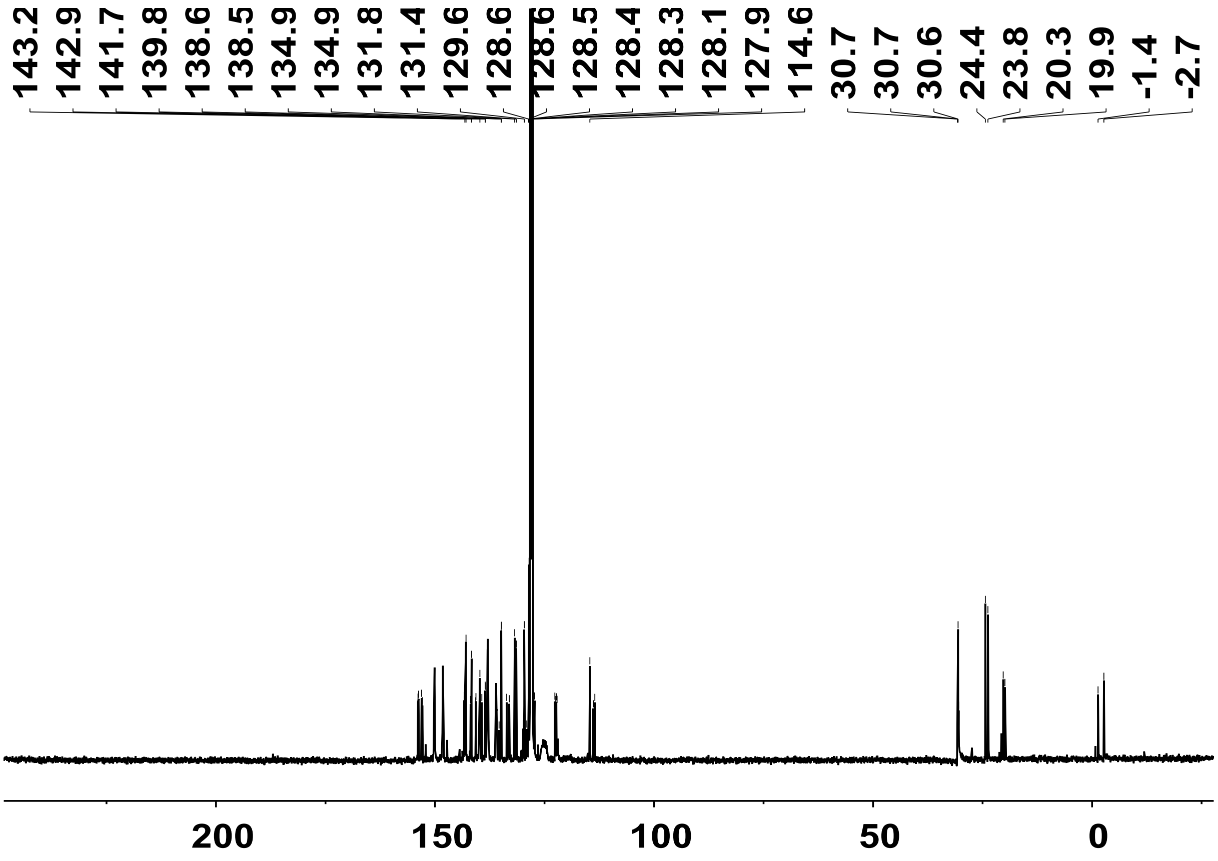

Figure S 72 – $^{13}C\{^{1}H\}$ NMR spectrum (125.71 MHz, 305.0 K, C_6D_6) of mesityltellanyl-stabilized phenylmethylsilyl borate **13f**[$B(C_6F_5)_4$].

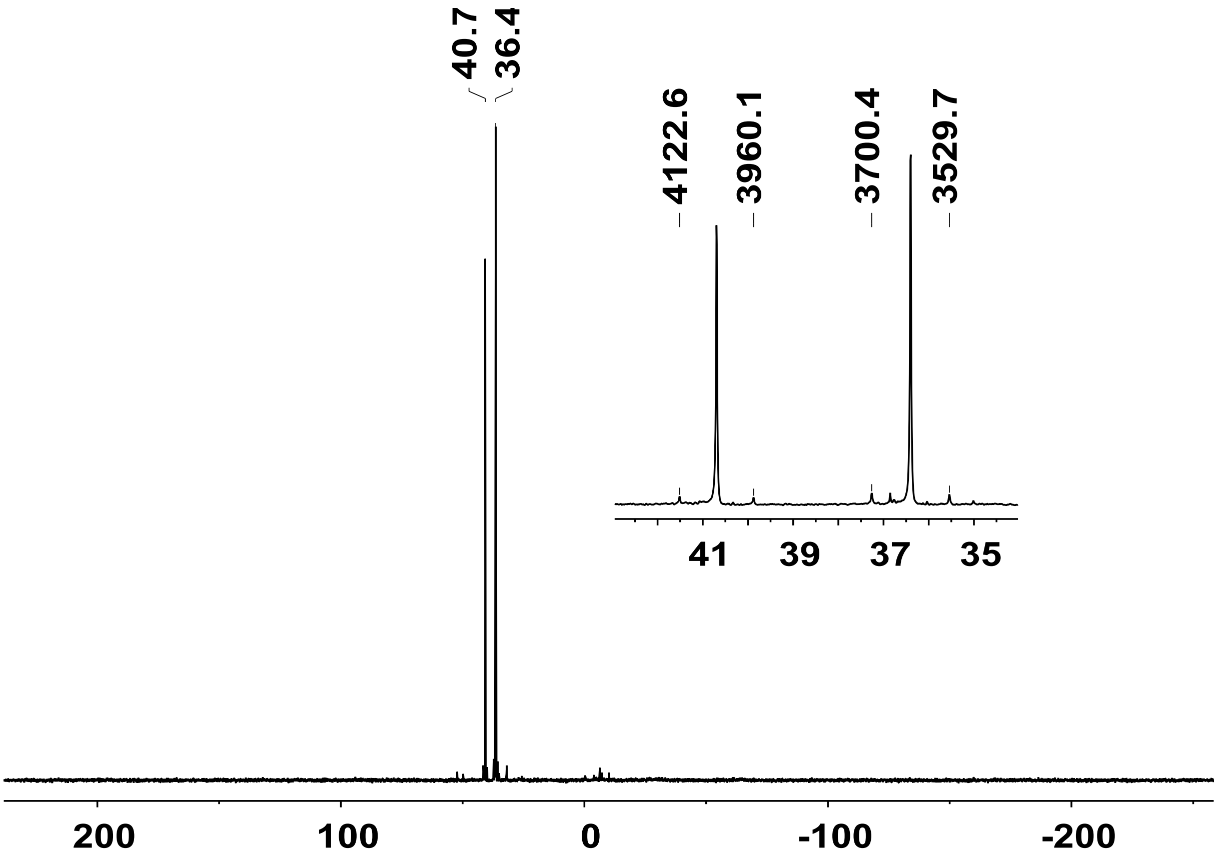

Figure S 73 – $^{29}Si\{^{1}H\}$ INEPT NMR spectrum (99.31 MHz, 305.0 K, C_6D_6) of mesityltellanyl-stabilized phenylmethylsilyl borate **13f**[$B(C_6F_5)_4$].

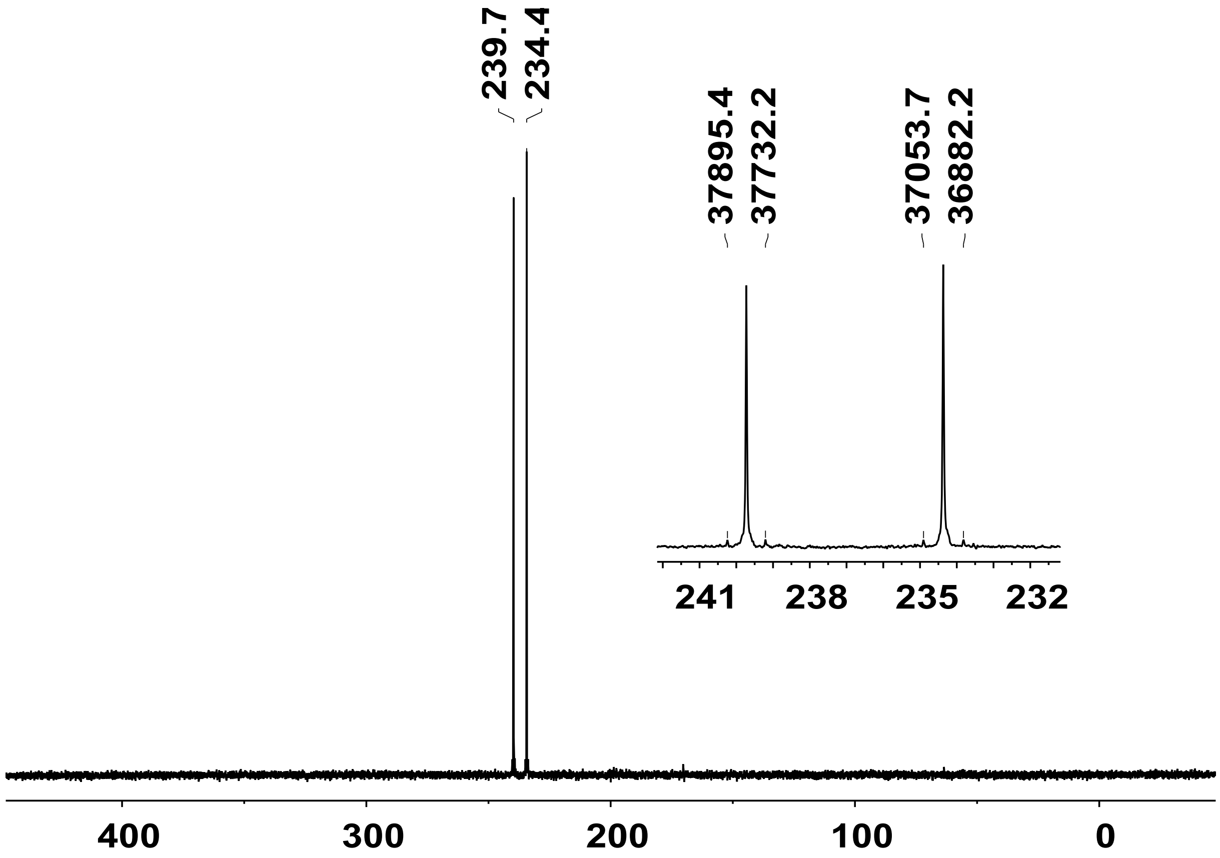

Figure S 74 – $^{125}Te\{^1H\}$ NMR spectrum (157.74 MHz, 305.0 K, C_6D_6) of mesityltellanyl-stabilized phenylmethylsilyl borate **13f**[B(C_6F_5)$_4$].

1.4 Chiral Resolution and Chiral Memory Experiments

General Procedure F: A Schlenk flask was charged with copper(I)chloride and triphenylphosphane in a ratio of 1:2. Toluene was added and the mixture was stirred until triphenylphosphane was dissolved. Subsequently sodium *tert*-butoxide (equimolar to CuCl) was added at r.t. and the mixture was stirred until the color turned yellow (5 – 10 min). The pyridyl alcohol (*R*)-E was dissolved in toluene and added at r.t. to the catalyst mixture. The mixture turned orange. Subsequently the silane was added either as a solid in one portion or dissolved in toluene whereupon the mixture turned brown-red. After stirring for approximately 16 h, the reaction mixture was filtrated through a thin layer of silica gel to remove the Cu(I) species, the solvent was removed and the crude product was purified by a two-step column chromatography if not mentioned otherwise in the details. The first column chromatography (eluent petroleum ether/ethyl acetate 100:0 → 50:50) with a short column resulted in two fraction: 1. (+)-Silane + Ph_3P, 2. Siloxanes + impurities. Both fractions needed further purification which is specified for each compound in detail below.

6-Phenoxy-5-methylphenylsilylacenaphthene 3a

The kinetic resolution of the title compound **3a** was performed according to General Procedure **F** using 305.6 µmol copper(I) chloride, 1.61 mmol of the pyridyl alcohol and 3.06 mmol of silane **3a.** The catalyst was prepared as usual at r.t., then the mixture was cooled with an ice bath, first the alcohol/toluene and subsequently the silane/toluene mixture was added dropwise, the mixture was warmed slowly to r.t. over night. The crude product was purified by a short column chromatography (eluent petroleum ether/ethyl acetate 100:0 → 0:100) resulting in two fractions. Fraction 1 ((+)-silane **3a** + Ph_3P) was further purified by oxidation of the phosphane with H_2O_2. Therefore, the solid were dissolved in petroleum ether and 0.6 mL H_2O_2 (30w% in H_2O) was added at r.t.. After stirring the mixture for 16 h the solid which precipitated was filtered off and the phases were separated. The solvent of the organic layer was removed and the residue was purified via recrystallization from hexanes. (+)-Silane **3a** was obtained with a yield of 52 % and an ee of 56 % ([α] = +12° (0.01 mol L^{-1})). Fraction 2 (siloxanes **12a**) was not further purified (yield 61 %). The crude fraction 2 was used for the reduction of siloxane to obtain (-)-silane **3a**.

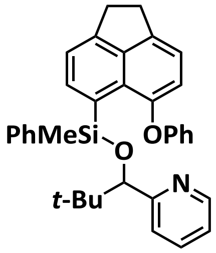

^{1}H NMR (499.87 MHz, 305.0 K, C_6D_6) δ = 0.99 (s, 3 H, $SiCH_3$), 1.21 (s, 9 H, *t*-Bu), 2.99-3.04 (m, 2 H, CH_2), 3.12-3.17 (m, 2 H, CH_2), 5.02 (s, 1 H, OCH), 6.47 (dm, $^3J_{H,H}$ = 7.7 Hz, 2 H, OPh), 6.50-6.53 (m, 1 H, Py), 6.56 (d, $^3J_{H,H}$ = 7.5 Hz, 1 H, 7-H), 6.78-6.81 (m, 2 H, Si-*m*-Ph), 6.82-6.85 (m, 2 H, 8-H, O-*ipso*-Ph), 6.89-6.97 (m, 4 H, Py, OPh, Si-*p*-Ph), 7.16-7.18 (m, 2 H, Si-*o*-Ph), 7.26 (dm, $^3J_{H,H}$ = 7.9 Hz, 1 H, Py), 7.46 (dm, $^3J_{H,H}$ = 6.9 Hz, 1 H, 3-H), 8.27-8.31 (m, 1 H, Py), 8.96 (d, $^3J_{H,H}$ = 6.9 Hz,1 H, 4-H). Additional signals for second diastereomer: 0.57 (s, SiCH3), 1.06 (s, *t*-Bu), 4.94 (s, OCH), 7.51 (d, $^3J_{H,H}$ = 7.0 Hz, 3-H), 8.47-8.49 (m, Py), 8.96 (d, $^3J_{H,H}$ = 7.0 Hz, 4-H). **^{13}C{^{1}H} NMR** (125.71 MHz, 305.0 K, C_6D_6) δ = -2.0 ($SiCH_3$), 26.8 (CH_3, *t*-Bu), 29.6 (CH_2), 31.0 (CH_2), 36.8 (C, *t*-Bu), 84.5 (CH, OC), 112.0 (CH, C-7), 119.1, 120.3 (CH, C-3), 121.3 (CH), 121.3 (CH, Py), 123.1 (CH, Py), 124.0 (CH), 126.1 (C), 127.1 (CH), 128.2 (CH, Py), 128.7 (C), 129.6 (CH), 133.6 (CH), 134.5 (CH, Py), 138.5 (CH, C-4), 139.0 (C), 140.0 (C, ace), 141.4 (C, ace), 147.5 (CH, Py), 148.5 (C, ace), 153.4 (C, C-6), 155.4 (C, O-*ipso*-Ph), 162.3 (C, Py). **^{29}Si{^{1}H} NMR** (99.31 MHz, 305.0 K, C_6D_6) δ = -5.0 (main), -4.6. **^{1}H/^{15}N HMBC NMR** (499.87 MHz, 305.0 K, C_6D_6) δ = 316.5 (main), 317.5.

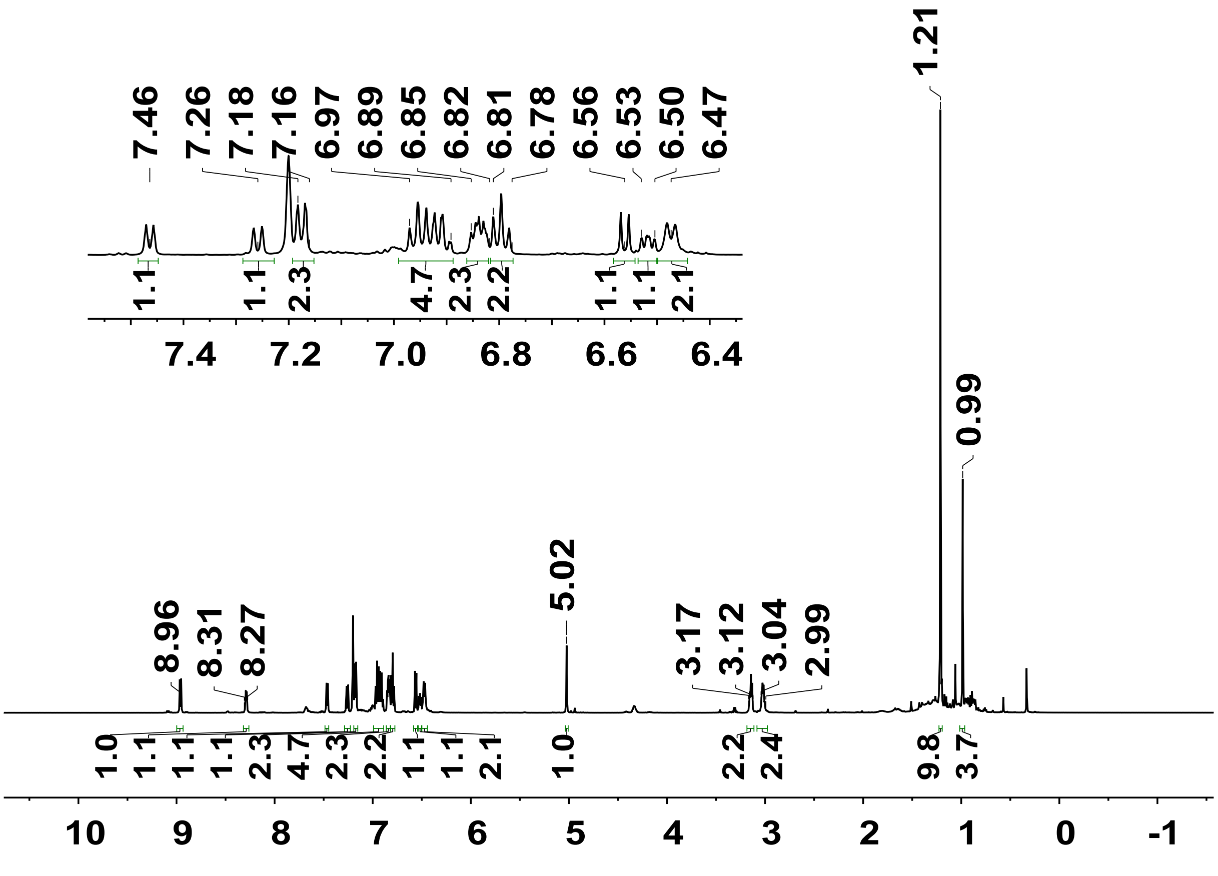

Figure S 75 – ^{1}H NMR spectrum (499.87 MHz, 305.0 K, C_6D_6) of siloxanes **12**(Ch=O).

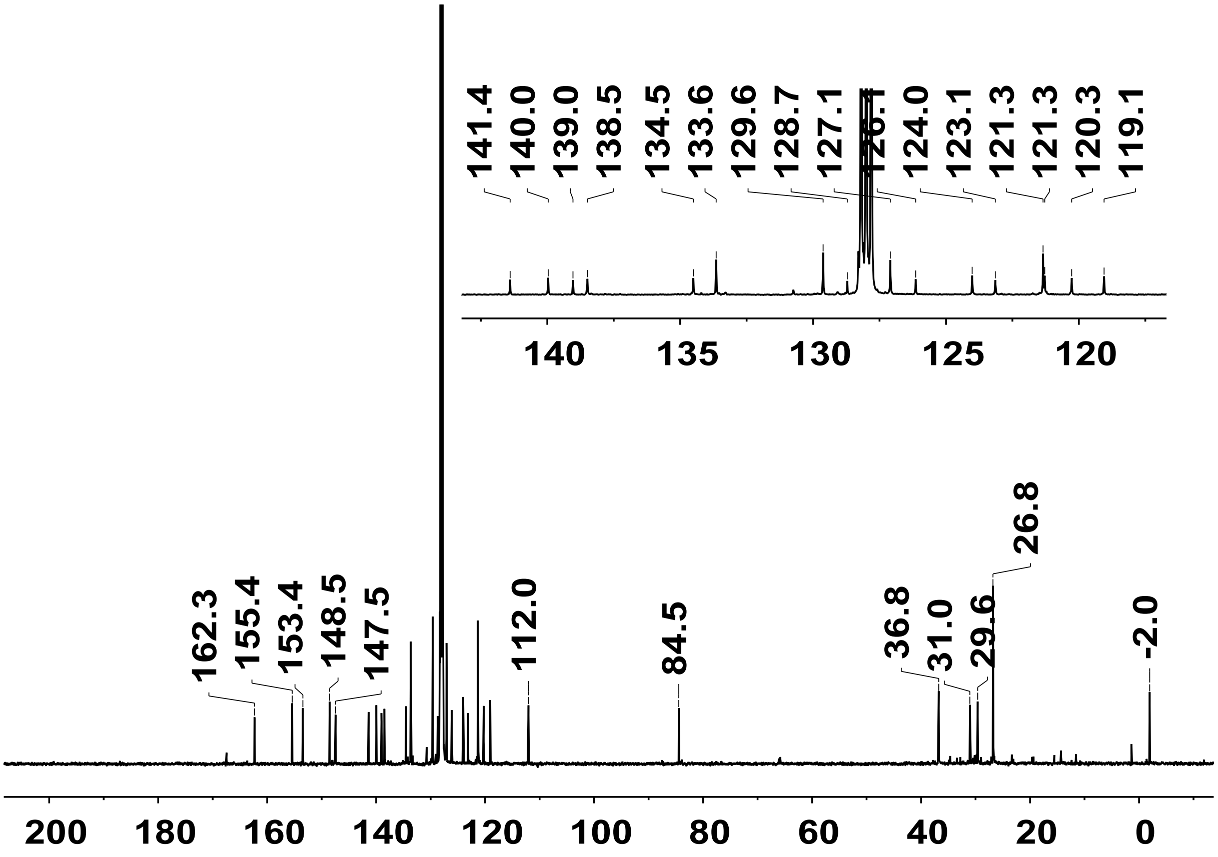

Figure S 76 – $^{13}C\{^{1}H\}$ NMR spectrum (125.71 MHz, 305.0 K, C_6D_6) of siloxanes **12**(Ch=O).

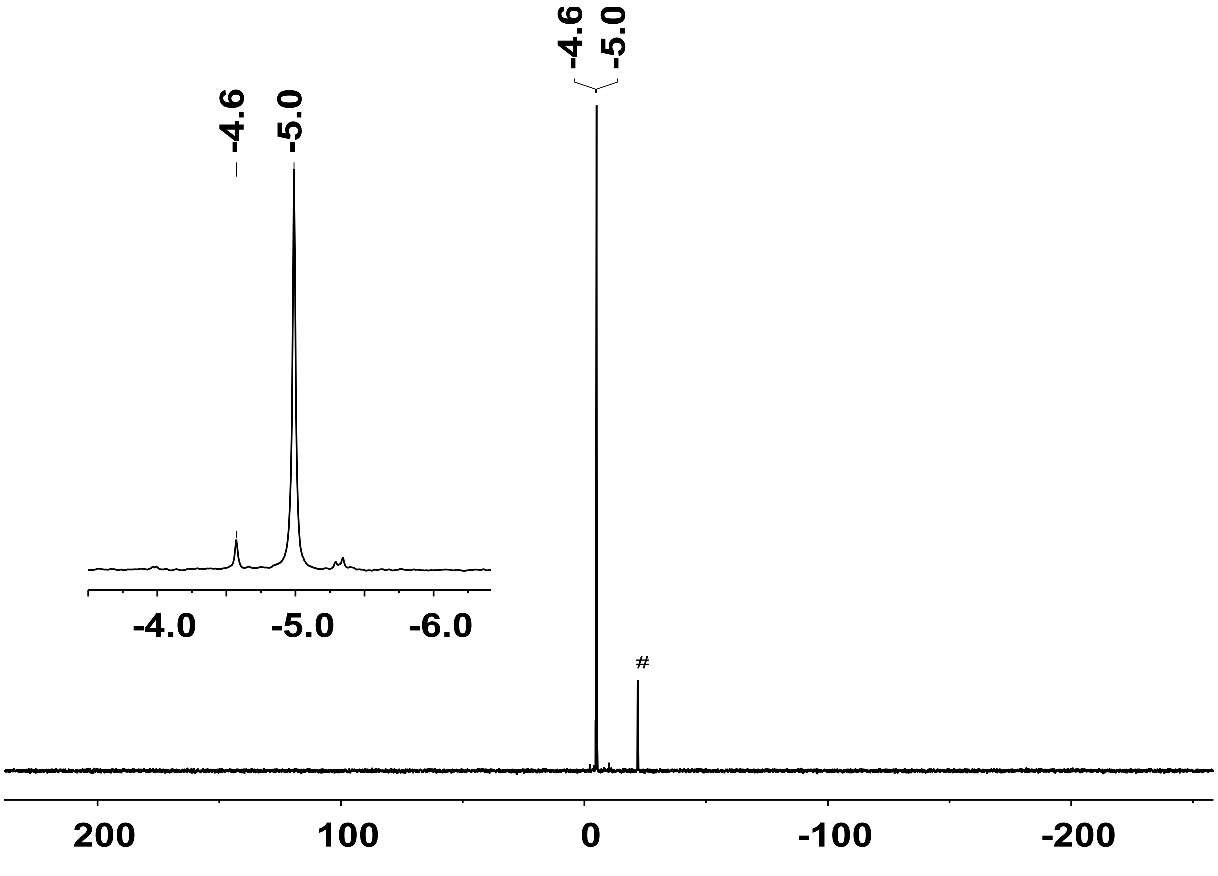

Figure S 77 – $^{29}Si\{^{1}H\}$ INEPT NMR spectrum ((99.31 MHz, 305.0 K, D_3 = 0.0122 s, D_4 = 0.0313 s, C_6D_6) of siloxanes **12**(Ch=O) (# silicon grease).

6-Methylphenylsilyl-5-phenylsulfanylacenaphthene **4a**

For the chiral resolution of 1.0 equiv. (1.05 mmol, 401.0 mg) of the title compound **4a** 0.2 equiv. (213.14 µmol, 21.1 mg) copper(I)chloride, 0.4 equiv. (242.34 µmol, 111.3 mg) triphenylphosphane, 0.2 equiv. (208.11 µmol, 20.0 mg) sodium *tert*-butoxide and 0.55 equiv. (576.75 µmol, 95.3 mg in 3.5 mL toluene) of the pyridyl alcohol (*R*)-**11** were used. The catalyst was prepared in 2.0 mL toluene and the silane **4a** was added as a solid in one portion. The crude product was an oil which was adsorbed on silica for a solid deposition at the column. Eluent for column chromatography petroleum ether/ethyl acetate 98:2 → 90:10. Two fractions collected: the first fraction was the (+)-silane **4a** and Ph_3P, which was further purified via preparative TLC (eluent petroleum ether/ethyl acetate 99:1, TLC was three times eluted). The yield of (+)-silane 43b ($[\alpha]_D$ = 11, c = 0.06 molL^{-1} in Et_2O; ee = 66 %) was 113.0 mg (295.35 µmol, 56 %). The second fraction contained the siloxanes **12b** and impurities. Siloxanes **12**(Ch=S) were obtained purely by adding a pentane/ethyl acetate 9:1 mixture to the oil. Siloxanes **12**(Ch=S) were dissolved and the impurities precipitated. After removal of the solvent, siloxanes **12**(Ch=S) were obtained as a colorless viscous oil. The yield of siloxanes **12**(Ch=S) was 171.0 mg (313.29 µmol, 60 %).

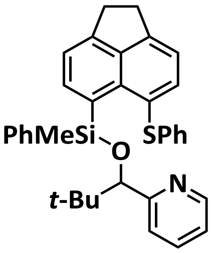

^{1}H NMR (499.87 MHz, 305.0 K, C_6D_6) δ = 1.09 (s, 3 H, $SiCH_3$), 1.19 (s, 9 H, *t*-Bu), 2.94-2.97 (m, 2 H, CH_2), 3.06-3.08 (m, 2 H, CH_2), 5.00 (s, 1 H, OCH), 6.49-6.52 (m, 1 H, Py), 6.56-6.58 (m, 2 H), 6.70-6.74 (m, 3 H), 6.77-6.80 (m, 2 H, SiPh), 6.84-6.90 (m, 2 H, Py), 6.93 (dm, $^3J_{H,H}$ = 7.2, 1 H, 3-H), 7.18-7.21 (m, 2 H, Py, overlap with C_6D_5H), 7.23-7.24 (m, 2 H, SiPh), 7.43 (dm, $^3J_{H,H}$ = 7.1, 1 H, 8-H), 7.66 (d, $^3J_{H,H}$ = 7.1 Hz, 1 H, 4-H), 8.29-8.30 (m, 1 H, Py), 9.19 (d, $^3J_{H,H}$ = 7.1 Hz, 7-H). Additional signals for second diasteromer: 0.66 (s, $SiCH_3$), 1.03 (s, *t*-Bu), 4.96 (s, OCH), 6.64-6.68 (m), 7.03-7.07 (m), 7.10-7.14 (m), 7.49 (dm, $^3J_{H,H}$ = 7.0 Hz), 7.53-7.56 (m), 7.59 (dm, $^3J_{H,H}$ = 7.9 Hz), 7.69 (d, $^3J_{H,H}$ = 7.2 Hz), 8.46-8.49 (m), 8.62 (d, $^3J_{H,H}$ = 7.0), 9.32 (d, $^3J_{H,H}$ = 7.1 Hz). **^{13}C{^{1}H} NMR** (125.71 MHz, 305.0 K, C_6D_6) δ = -0.6 ($SiCH_3$), 26.8 (CH_3, *t*-Bu), 30.1 (CH_2), 30.3 (CH_2), 36.8 (C, *t*-Bu), 84.5 (CH, OC), 120.2 (CH, C-8), 120.6 (CH, C-3), 121.2 (CH, Py), 123.2 (CH, Py), 124.8 (CH), 126.3 (C), 126.8 (CH), 127.3 (CH), 127.9 (CH), 128.6 (CH), 129.0 (C, C-6), 133.1 (CH, SiPh), 134.4 (CH), 139.4(CH, C-4), 139.4 (C), 140.0 (C), 140.9 (C, ace), 141.3 (CH, C-7), 141.4 (C), 147.5 (CH, Py), 149.3 (C, ace), 149.6 (C, ace),

162.2 (C, Py). Additional signals for the second diastereomer: -0.2 ($SiCH_3$), 26.7 (CH_3, *t*-Bu), 36.7 (C, *t*-Bu), 83.9 (CH, OC), 120.3 (CH), 120.6 (CH), 121.7 (CH), 122.9 (CH), 124.7 (CH), 126.2 (C), 126.5 (CH), 127.1, (CH), 127.9 (CH), 128.3 (CH), 129.0 (C), 135.1 (CH), 139.5 (CH), 139.6 (C), 140.1 (CH), 141.2 (C), 141.3 (CH), 141.7 (C), 148.0 (CH), 163.7 (C). **^{29}Si{^{1}H} NMR** (99.31 MHz, 305.0 K, C_6D_6) δ = -7.7 (main), -7.5. **^{1}H/^{15}N HMBC NMR** (499.87 MHz, 305.0 K, C_6D_6) δ = 310.6, 307.8 (main).

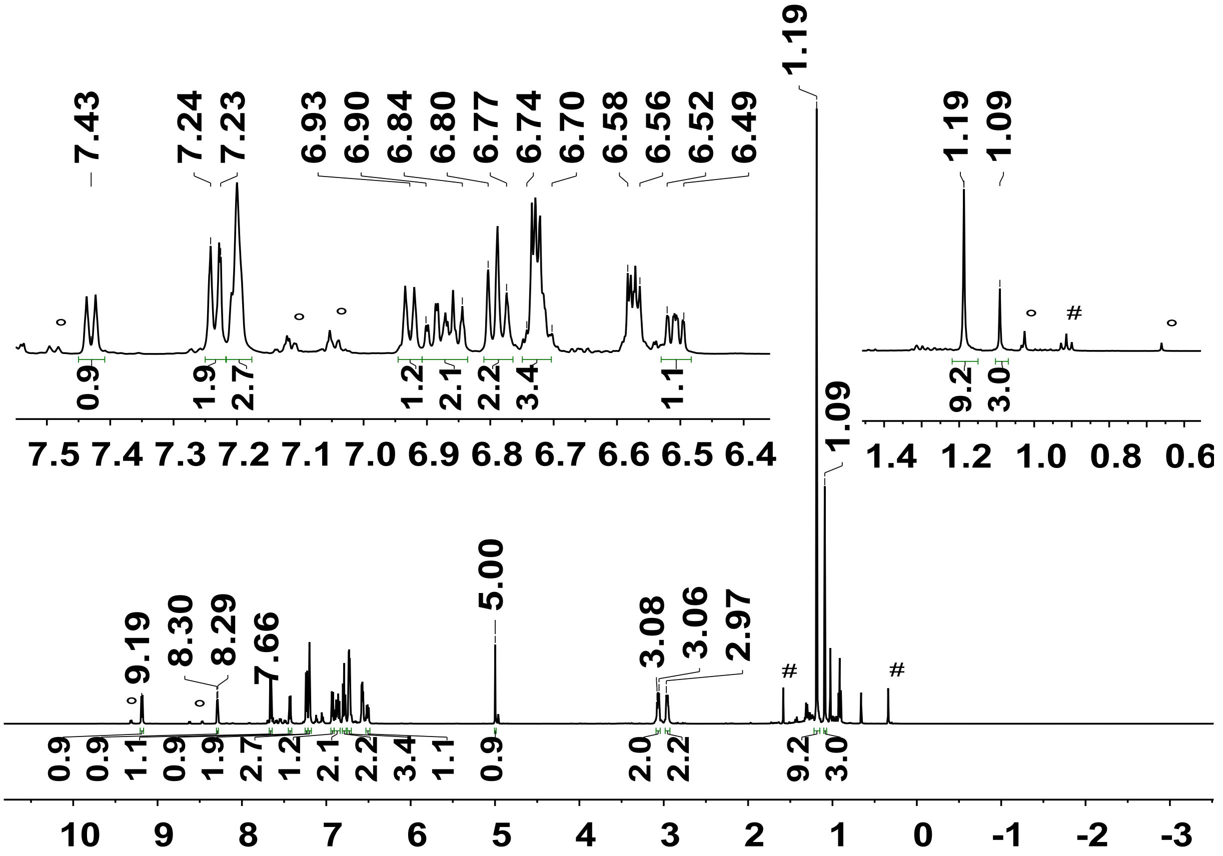

Figure S 78 – ^{1}H NMR spectrum (499.87 MHz, 305.0 K, C_6D_6) of siloxanes **12**(Ch=S) (° second diastereomer, # impurities or residual solvent).

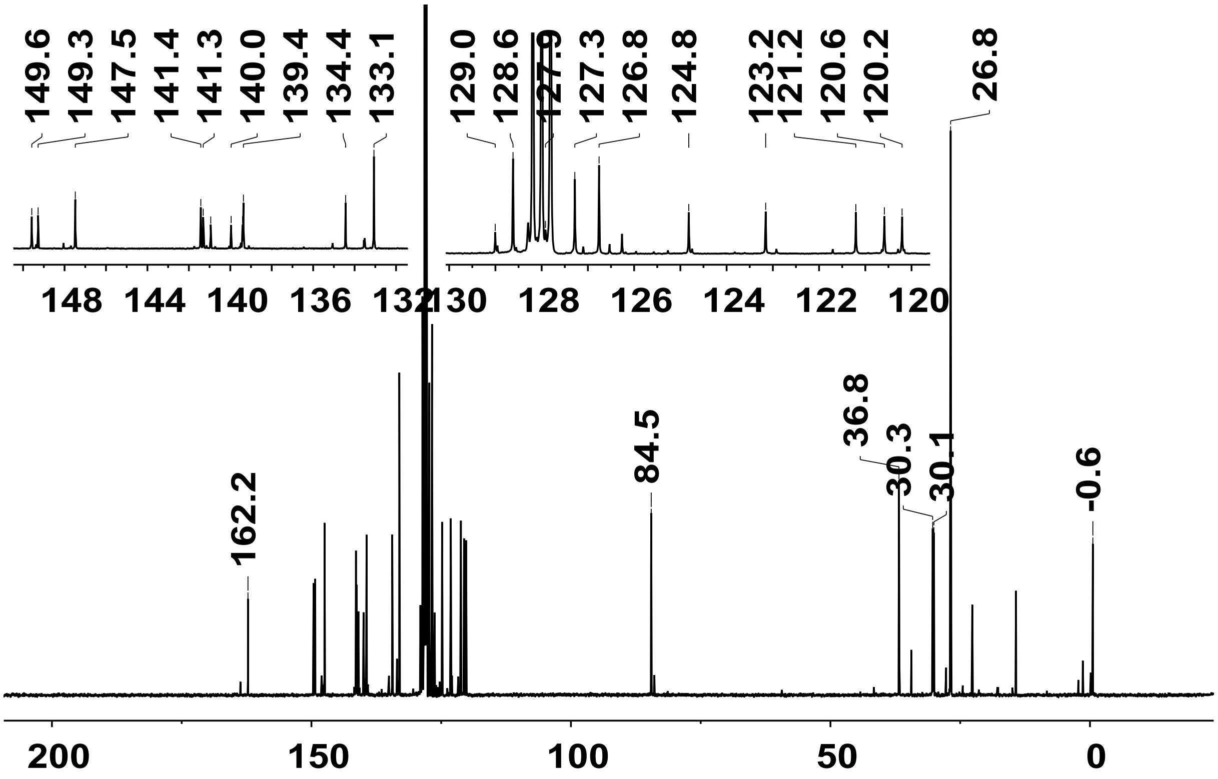

Figure S 79 – $^{13}C\{^{1}H\}$ NMR spectrum (125.71 MHz, 305.0 K, C_6D_6) of siloxanes **12**(Ch=S).

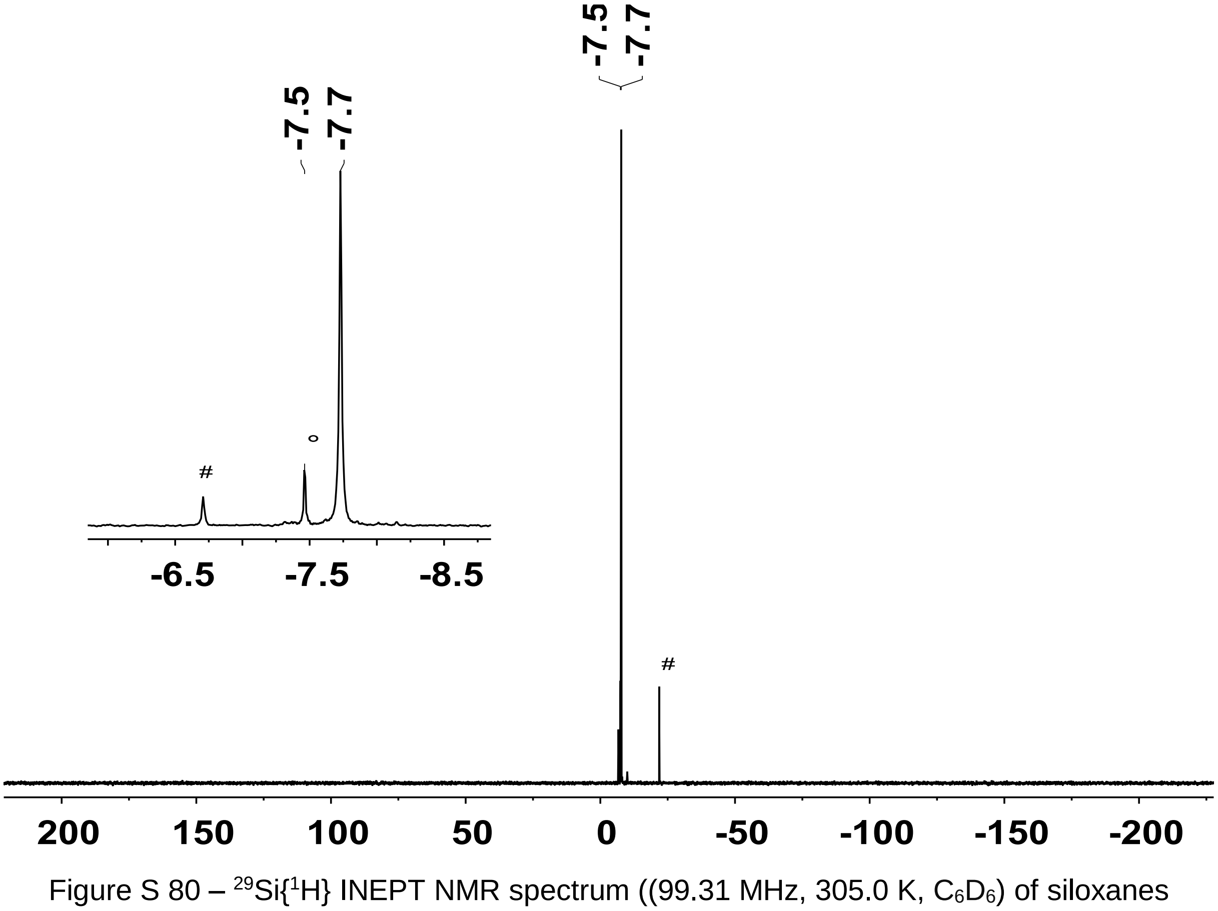

Figure S 80 – $^{29}Si\{^{1}H\}$ INEPT NMR spectrum ((99.31 MHz, 305.0 K, C_6D_6) of siloxanes **12**(Ch=S) (° second diastereomer, # silicon grease/impurities).

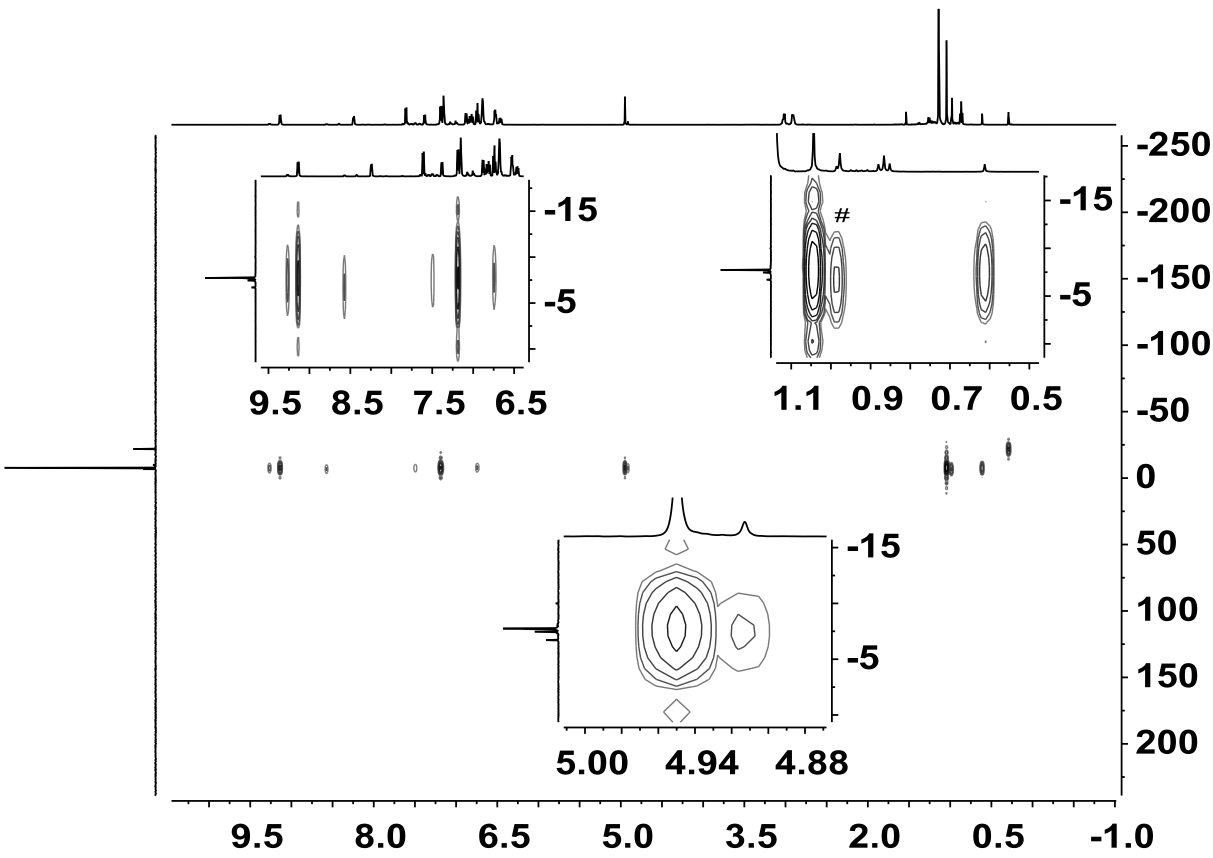

Figure S 81 – $^{1}H/^{29}Si$ HMBC NMR spectrum (499.87 MHz, 304.9 K, C_6D_6) of siloxanes **12**(Ch=S) (# impurities).

8-Methylphenylsilyl-1-phenylsulfanylnaphthalene **9**

For the chiral resolution of 1.0 equiv. (818.94 µmol, 292.0 mg) of the title compound **9** 0.1 equiv. (163.78 µmol, 16.0 mg) copper(I)chloride, 0.2 equiv. (327.58 µmol, 86.0 mg) triphenylphosphane, 0.1 equiv. (163.78 µmol, 16.0 mg) sodium *tert*-butoxide and 0.6 equiv. (491.36 µmol, 81.0 mg in 2 mL toluene) of pyridyl alcohol (*R*)-**11** were used. The catalyst was prepared in 2 mL toluene and the silane was added as a solid. The first column chromatography resulted in two fractions: 1. (+)-silane **9** + Ph_3P (209 mg), 2. Siloxanes **12**(naph,Ch=O) + impurities (217 mg). Preparative TLC of the first fraction (eluent petroleum ether, eluted three times) gave 86.0 mg (241.19 µmol, 59 %) of (+)-silane **9** ($[\alpha]_D$ = 17, c = 0.04 $molL^{-1}$ in Et_2O; ee = 84 %)). Preparative TLC of the second fraction (eluent petroleum ether/ethyl acetate 9:1) gave 159.0 mg (305.90 µmol, 75 %) of siloxanes **12**(naph,Ch=O).

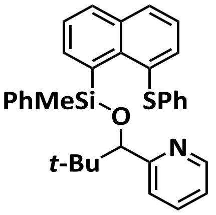

^{1}H NMR (499.87 MHz, 305.1 K, C_6D_6) δ = 1.07 (s, 3 H, $SiCH_3$), 1.15 (s, 9 H, *t*-Bu), 4.91 (s, 1 H, OCH), 6.40-6.43 (m, 2 H, SPh), 6.48-6.52 (m, 1 H, Py), 6.65-6.71 (m, 3 H, SPh), 6.74-6.78 (m, 2 H), 6.83-6.88 (m, 2H, Py), 7.04-7.10 (m, 3 H, naph, Py), 7.13-7.17 (m, 2 H, SiPh), 7.56-7.61 (m, 1 H, 6-H), 7.62-7.67 (m, 2 H, SiPh), 7.76-7.79 (m, 1 H), 8.26-8.29 (m, 1 H, Py), 9.24-9.28 (m, 7-H), the sum of the integrals in the aromatic region is by 1 H too high, most likely due to overlap with the second diastereomer. Additional signals for second diastereomer: 0.63 (s, $SiCH_3$), 0.99 (s, *t*-Bu), 4.90 (s, OCH), 6.36-6.39 (m), 6.57-6.59 (m), 7.43-7.48 (m), 7.49-7.52 (m), 8.44-8.47 (m, Py), 9.33-9.36 (m, 7-H). **^{13}C{^{1}H} NMR** (125.71 MHz, 305.0 K, C_6D_6) δ = -0.6 ($SiCH_3$), 26.7 (CH_3, *t*-Bu), 36.6 (C, *t*-Bu), 84.4 (CH, OC), 120.9 (CH, Py), 122.9 (CH, Py), 125.0 (CH), 125.8 (CH, naph), 125.8 (CH), 126.7 (CH, SPh), 127.1 (CH), 127.6 (CH), 128.5 (CH), 131.2 (C), 131.3 (CH), 131.5 (CH), 132.6 (CH), 134.1 (C, C-8/Si-*ipso*-Ph), 134.2 (CH), 135.4 (C), 137.0 (CH), 139.2 (C, C-8/Si-*ipso*-Ph), 140.1 (CH, C-7), 140.1 (C), 140.9 (C), 147.3 (CH, Py), 161.9 (C, Py). Additional signals for the second diastereomer: -0.1 ($SiCH_3$), 26.6 (CH_3, *t*-Bu), 36.5 (C, *t*-Bu), 83.3 (CH, OC), 121.5 (CH), 122.7 (CH), 124.9 (CH), 135.6 (C), 140.6 (C), 141.7 (C), 147.9 (CH), 163.4 (C). ^{29}Si{^{1}H} NMR (99.31 MHz, 305.0 K, C_6D_6) δ = -11.2 (main), -10.9. ^{1}H/^{15}N HMBC NMR (499.87 MHz, 305.0 K, C_6D_6) δ = 316.6. HRMS protonated at N atom $C_{33}H_{34}NOSSi$, found: 520.2128, calculated 520.2130.

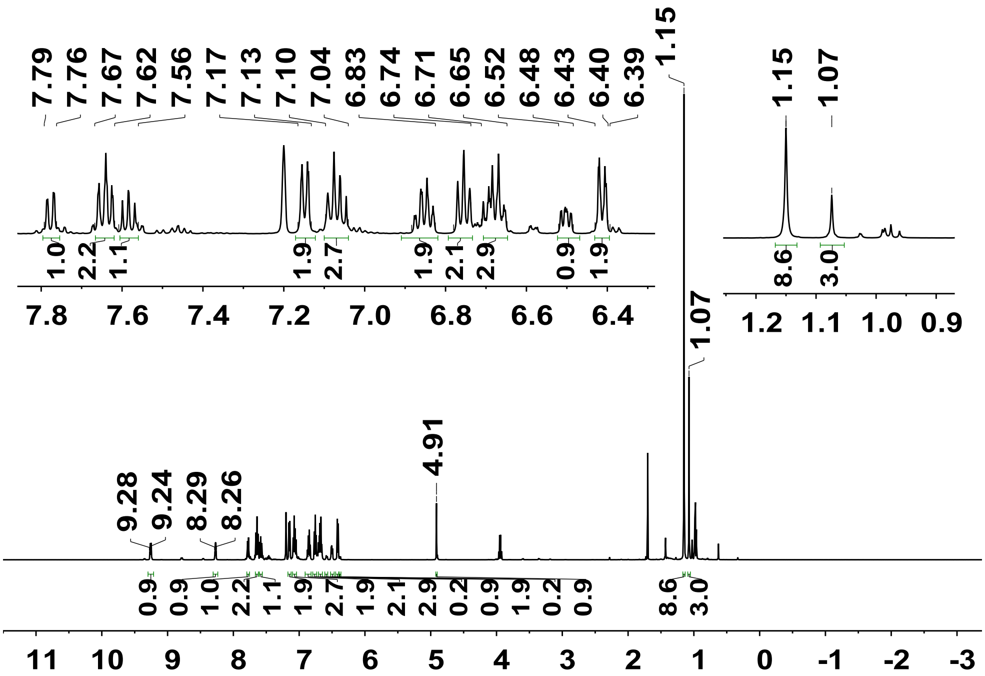

Figure S 82 – ^{1}H NMR spectrum (499.87 MHz, 305.0 K, C_6D_6) of siloxanes **12**(naph,Ch=O) (° second diastereomer, # impurities or residual solvent).

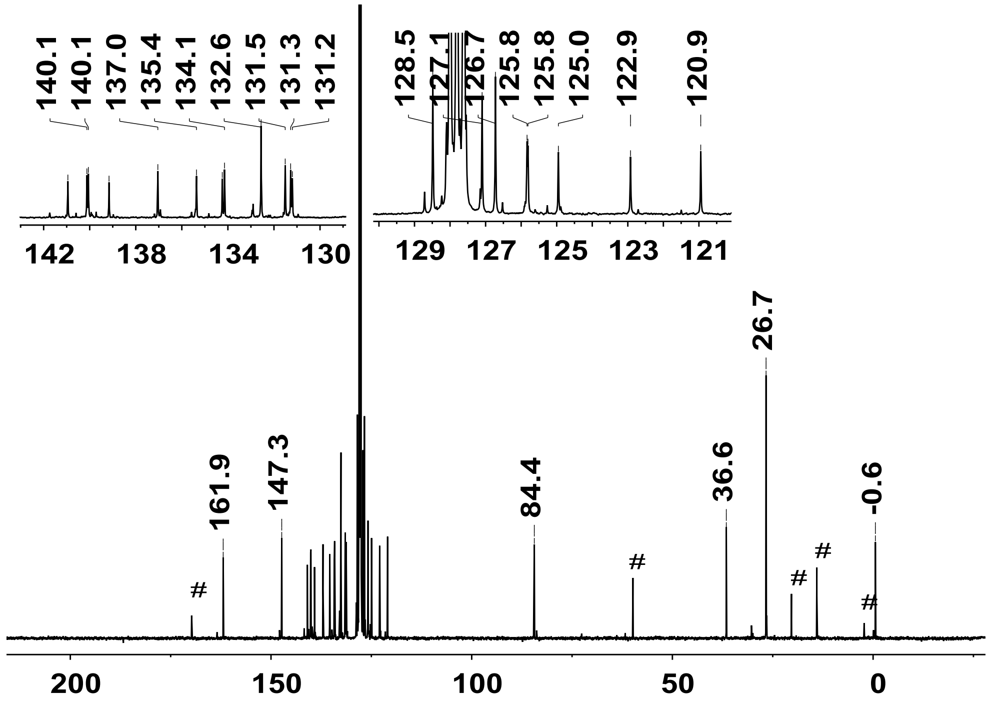

Figure S 83 – $^{13}C\{^{1}H\}$ NMR spectrum (125.71 MHz, 305.0 K, C_6D_6) of siloxanes **12**(naph,Ch=O).

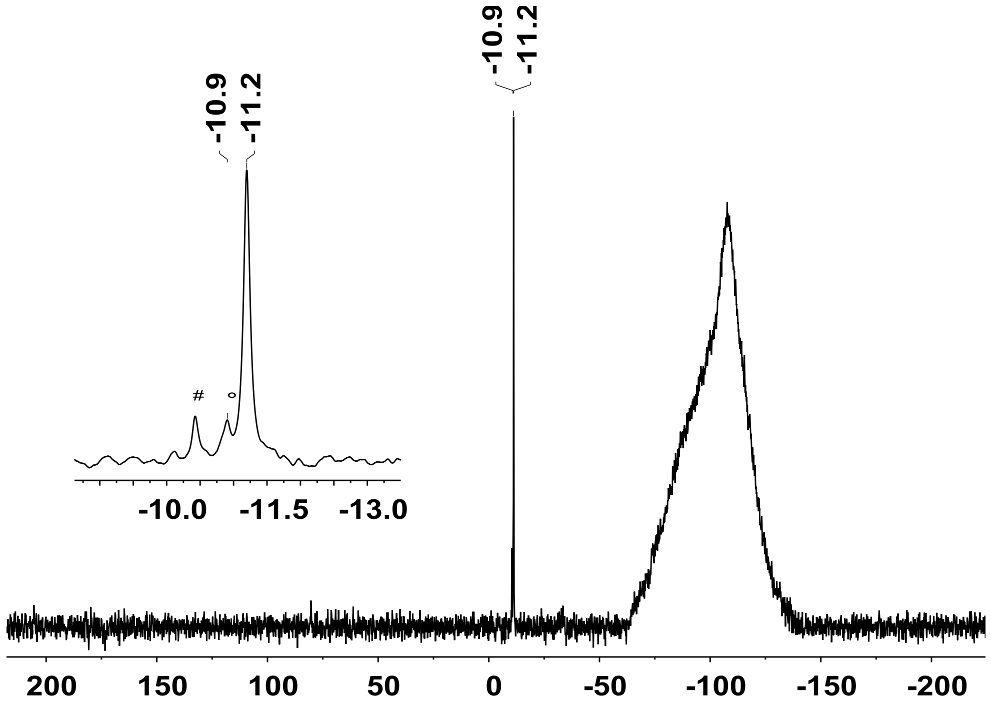

Figure S 84 – $^{29}Si\{^{1}H\}$ NMR spectrum (99.31 MHz, 305.0 K, C_6D_6) of siloxanes **12**(naph,Ch=O) (second diastereomer, # impurities).

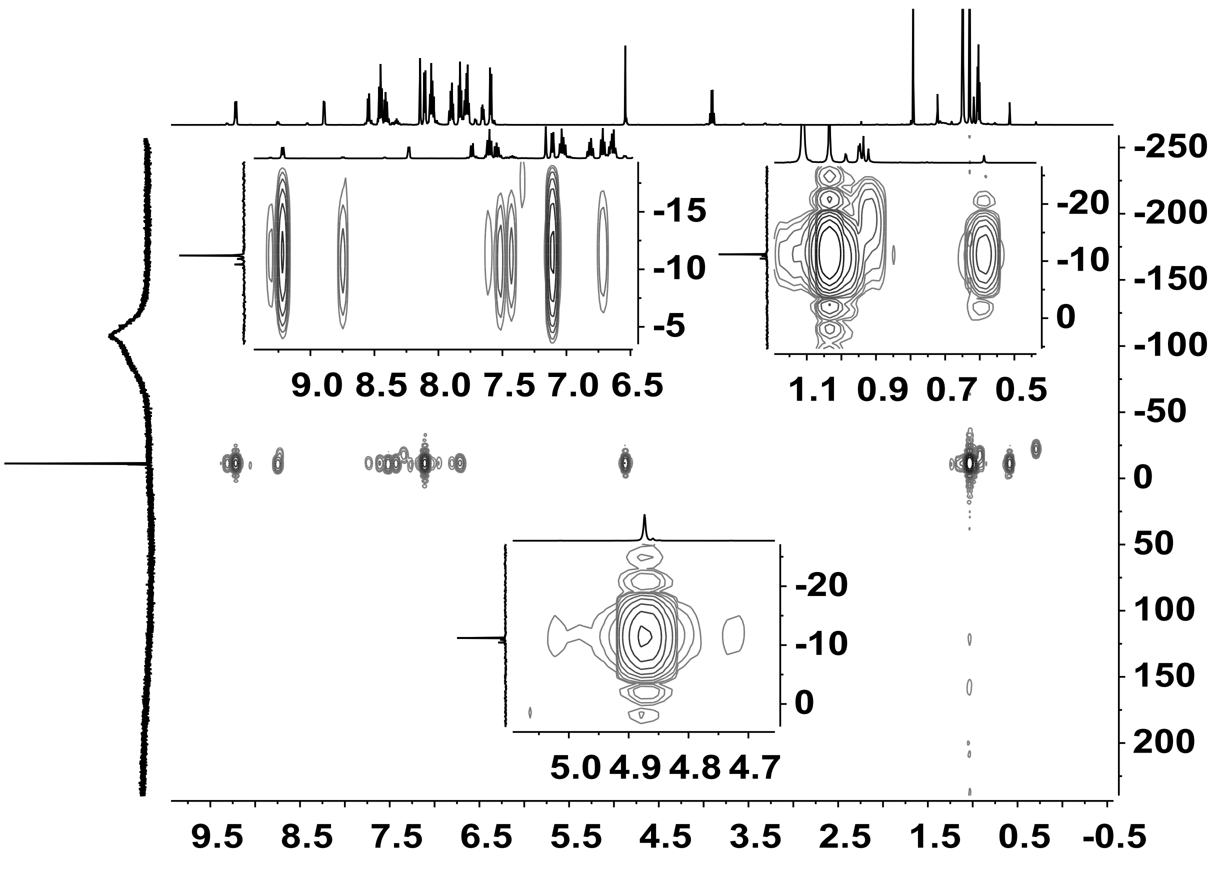

Figure S 85 – $^{1}H/^{29}Si$ HMBC NMR spectrum (499.87 MHz, 304.9 K, C_6D_6) of siloxanes **12** (# impurities).

Reduction of siloxanes 12

The siloxanes **12** were dissolved in 2-20 mL diethylether and di-*iso*-butylaluminiumhydride (1 M in *n*-hexane) was added at r.t.. The mixture was stirred for 16 h and afterwards quenched by addition of 1-20 mL 1 M hydrochloric acid. The two phasic mixture was stirred for 20-60 min, the phases were separated and the product was extracted from the aqueous layer (3 x 5-20 mL Et_2O). After removal of the solvent, the product was purified via column chromatography or preparative TLC (eluent PE). Details see
Table S1 **Entry 1**: Reaction in 20 mL Et_2O, work up with 20 mL 1 M hydrochloric acid. **Entry 2**: Reaction in 4 mL Et_2O, work up with 3 mL 1 M hydrochloric acid. **Entry 3**: Reaction in 2 mL Et_2O, work up with 1 mL 1 M hydrochloric acid.

Table S1 – Batches of the reduction of siloxanes **12** to the corresponding (-)-silanes **3a**, **4a** and **9** ($[\alpha]_D$ measured in Et_2O).

**Entry**	**Comp.**	**1.0 equiv. siloxane**	**2.0-2.5 equiv. DIBAL-H**	**Yield**	**$[\alpha]_D$ (-)-silane**	**ee (-)-silane**
**1**	**12**(Ch=O)	993 mg (1.87 mmol)	4.70 ml (4.69 mmol)	561 mg (82 %)	-11° (0.02 mol L^{-1})	64 %
**2**	**12**(Ch=S)	174 mg (318.79 µmol)	0.64 mL (637.58 µmol)	97 mg (80 %)	-11° (0.05 mol L^{-1})	64 %
**3**	**12**(naph,Ch=O)	158 mg (303.98 µmol)	0.76 mL (759.94 µmol)	75 mg (69 %)	-15° (0.04 mol L^{-1})	54 %

Chiral Memory

A Schlenk tube was charged with 1.0 equiv. of silane **(-)-3a**, **(-)-4a** or **(+)-9** and a second Schlenk tube was charged with 1.0 equiv. of trityl borate $[Ph_3C][B(C_6F_5)_4]$. The solids were dissolved in DCM or chlorobenzene, respectively. The silane was cooled to the temperature indicated in Table S2 and trityl borate was added. The mixture was stirred for the time indicated in Table S2. Then, sodium triethyl borohydride in toluene was added and the mixture was stirred overnight. The solvent was removed and the residue was suspended in petroleum ether. The mixture was filtrated through a thin layer of silica, the solvent was removed and the crude product was purified via preparative TLC (eluent petroleum ether). After the purification, the formation of silanes was confirmed by NMR spectroscopy and then their optical rotation was measured and their ee was determined via chiral HPLC (Table S3)

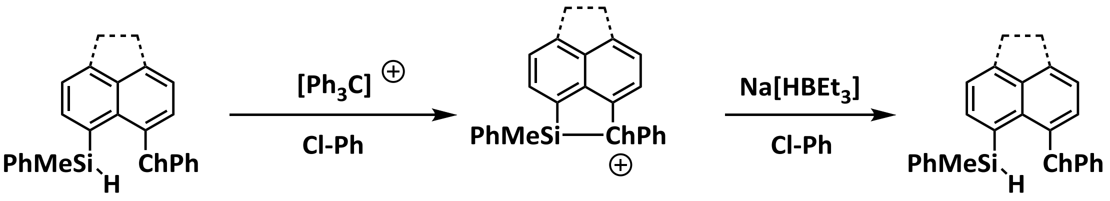

(-)-3a, (-)-4a, (+)-9 **13a, 13c, 14a** **(-)-3a, (-)-4a, (+)-9**

Table S2 – Batches, reaction conditions and yields of the chiral memory experiments.

Comp.	Silane	Trityl borate	Et_3B-H (1 M in toluene)	Conditions	Yield
**(+)-9**	106.6 µmol, 38 mg	106.6 µmol, 98 mg	159.9 µmol, 0.15 mL	Cl-Ph, r.t., 30 min	24 %
**(-)-3a**	177.3 µmol, 65 mg	173.5 µmol, 160 mg	266.0 µmol, 0.27 mL	Cl-Ph, -40 °C, 30 min	14 %
**(-)-3a**	180.1 µmol, 66 mg	180.1 µmol, 166 mg	270.1 µmol, 0.27 mL	DCM, -80 °C, 20 min	0 %
**(-)-4a**	252.5 µmol, 97 mg	252.5 µmol, 233 mg	328.2 µmol, 0.33 mL	Cl-Ph, -40 °C, 15 min	14 %

Table S3 – Results of the optical rotation measured in Et_2O and the chiral HPLC analysis of the chiral memory experiments-

Silane	$[\alpha]_D$ start	ee start	$[\alpha]_D$ end	ee end
**(+)-9**	+17° (0.04 $molL^{-1}$)	84 %	0° (0.004 $molL^{-1}$)	-
**(-)-3a**	-11° (0.02 $molL^{-1}$)	54 %	-4° (0.005 $molL^{-1}$)	32 %
**(-)-3a**	-11° (0.02 $molL^{-1}$)	54 %	Decomposition	decomposition
**(-)-4a**	-10° (0.05 $molL^{-1}$)	64 %	-9° (0.006 $molL^{-1}$)	64 %

1.5 Data from X-ray diffraction analysis of compounds 3a, 4a and 4b

Data were recorded on a Bruker CCD area detector. Structures were refined anisotropically using the program SHELXL-97.[S5] Pertinent data are summarized in Tables S4-6. CCDC-2011380, CCDC-2011381, CCDC-2011382 contain the supplementary crystallographic data for this paper. These data can be obtained free of charge from The Cambridge Crystallographic Data Centre. The Director, CCDC, 12 Union Road, Cambridge CB2 1EZ, UK [Fax: (internat.) +44-1223/336-033; E-mail: deposit@ccdc.cam.ac.uk].

Table S4.

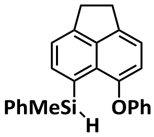

Empirical formula	C25 H22 O Si	
Formula weight	366.51	
Temperature	130(2) K	
Wavelength	0.71073 Å	
Crystal system	Triclinic	
Space group	P-1	
Unit cell dimensions	a = 8.5366(3) Å	a = 93.0590(16)°.
	b = 9.6243(3) Å	b = 108.3412(16)°.
	c = 12.6333(4) Å	g = 91.6942(16)°.
Volume	982.63(6) Å^3	
Z	2	
Density (calculated)	1.239 Mg/m^3	
Absorption coefficient	0.131 mm^{-1}	
F(000)	388	
Crystal size	0.400 x 0.300 x 0.080 mm^3	
Theta range for data collection	1.702 to 32.029°	
Index ranges	-12<=h<=12, -14<=k<=14, -18<=l<=18	
Reflections collected	29138	
Independent reflections	6839 (R(int) = 0.0263)	
Observed reflections (I > 2(I))	5592	
Completeness to theta = 32.029°	100.0 %	
Absorption correction	Semi-empirical from equivalents	
Max. and min. transmission	1.0000 and 0.9178	
Refinement method	Full-matrix least-squares on F^2	
Data / restraints / parameters	6839 / 0 / 270	
Goodness-of-fit on F^2	1.015	
Final R indices (I>2sigma(I))	R1 = 0.0454, wR2 = 0.1168	
R indices (all data)	R1 = 0.0583, wR2 = 0.1249	
Extinction coefficient	n/a	
Largest diff. peak and hole	0.450 and -0.255 e.Å^{-3}	

Table S5

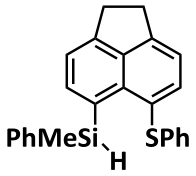

Empirical formula	C25 H22 S Si	
Formula weight	382.57	
Temperature	130(2) K	
Wavelength	0.71073 Å	
Crystal system	Monoclinic	
Space group	Cc	
Unit cell dimensions	a = 10.0944(6) Å	a = 90°.
	b = 12.9115(8) Å	b = 91.183(3)°.
	c = 30.6744(17) Å	g = 90°.
Volume	3997.1(4) $Å^3$	
Z	8	
Density (calculated)	1.271 Mg/m^3	
Absorption coefficient	0.229 mm^{-1}	
F(000)	1616	
Crystal size	0.220 x 0.220 x 0.180 mm^3	
Theta range for data collection	1.328 to 30.034°	
Index ranges	-14<=h<=14, -18<=k<=18, -43<=l<=43	
Reflections collected	38800	
Independent reflections	11514 (R(int) = 0.0468)	
Observed reflections (I > 2(I))	8195	
Completeness to theta = 30.034°	100.0 %	
Absorption correction	Semi-empirical from equivalents	
Max. and min. transmission	1.0000 and 0.8984	
Refinement method	Full-matrix least-squares on F^2	
Data / restraints / parameters	11514 / 2 / 517	
Goodness-of-fit on F^2	1.028	
Final R indices (I>2sigma(I))	R1 = 0.0594, wR2 = 0.1169	
R indices (all data)	R1 = 0.0932, wR2 = 0.1295	
Absolute structure parameter	0.25(12)	
Extinction coefficient	n/a	
Largest diff. peak and hole	0.437 and -0.573 e.$Å^{-3}$	

Table S6

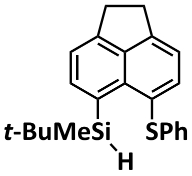

Empirical formula	C23 H26 S Si	
Formula weight	362.59	
Temperature	100(2) K	
Wavelength	0.71073 Å	
Crystal system	Triclinic	
Space group	P-1	
Unit cell dimensions	a = 9.3083(4) Å	a = 83.2067(14)°.
	b = 9.9308(4) Å	b = 72.0268(13)°.
	c = 11.2694(5) Å	g = 82.5637(13)°.
Volume	979.17(7) $Å^3$	
Z	2	
Density (calculated)	1.230 Mg/m^3	
Absorption coefficient	0.229 mm^{-1}	
F(000)	388	
Crystal size	0.400 x 0.350 x 0.300 mm^3	
Theta range for data collection	1.906 to 40.249°	
Index ranges	-16<=h<=16, -18<=k<=18, -20<=l<=20	
Reflections collected	83778	
Independent reflections	12317 (R(int) = 0.0170)	
Observed reflections (I > 2(I))	10986	
Completeness to theta = 40.249°	100.0 %	
Absorption correction	Semi-empirical from equivalents	
Max. and min. transmission	1.0000 and 0.9688	
Refinement method	Full-matrix least-squares on F^2	
Data / restraints / parameters	12317 / 0 / 234	
Goodness-of-fit on F^2	1.013	
Final R indices (I>2sigma(I))	R1 = 0.0291, wR2 = 0.0843	
R indices (all data)	R1 = 0.0338, wR2 = 0.0891	
Extinction coefficient	n/a	
Largest diff. peak and hole	0.892 and -0.539 e.$Å^{-3}$	

2. Computational Details

All quantum chemical calculations were carried out using the Gaussian09 package.[S7]
The molecular structure optimizations were performed using the M06-2X functional[S8] along with the def2-TZVP basis set for the elements Te, Se, S, Si, O, C, H and using the corresponding pseudopotential for Te.[S9] Every stationary point was identified by a subsequent frequency calculation either as minimum (Number of imaginary frequencies (NIMAG): 0) or transition state (NIMAG: 1). The SCF energies (E(SCF)) and the absolute computed Gibbs free energies at T = 298.15 K and p = 0.101 MPa (1 atm) in the gas phase (G298) are given in Table S7 for all optimized molecular structures. The optimized molecular structures of all compounds of interest are given as cartesian coordinates in the structure file (Computed_Molecular_structures.xyz).

Table S7. Calculated absolute energies, E(SCF), and free enthalpies at 298 K, G^{298} for the compounds of interest (at M06-2X/def2-TZVP). "Cation isodes" and "silane isodes" are silyl cations and silanes used for the calculation of the isodesmic reactions given in Scheme 5.

Compound	E(SCF) [a.u.]	ZPVE [kJ mol^{-1}]	G(298) [a.u.]
Ace-OPh-SiMePh cation **13a**	-1329.77950	1023	-1329.44362
Ace-OPh-SiMePh cation isodes	-1329.74123	1021	-1329.40743
Ace-OPh-SiMePh silane isodes	-1330.58763	1040	-1330.24734
Ace-OPh-SiMePh silane **3a**	-1330.58994	1044	-1330.24496
Ace-OPh-SiMe(tBu) cation **13b**	-1255.96866	1101	-1255.60243
Ace-OPh-SiMe(tBu) cation isodes	-1255.92539	1101	-1255.56205
Ace-OPh-SiMe(tBu) silane **3b**	-1256.77680	1125	-1256.40153
Ace-OPh-SiMe(tBu) silane isodes	-1256.77925	1122	-1256.40580
Ace-SPh-SiMePh cation cis *cis*-**13c**	-1652.75595	1015	-1652.42261
Ace-SPh-SiMePh cation trans *trans*-**13c**	-1652.75408	1016	-1652.42098
Ace-SPh-SiMePh cation TS **TS**(cis/trans)	-1652.72635	1014	-1652.39463
Ace-SPh-SiMePh cation isodes	-1652.70662	1013	-1652.37807
Ace-SPh-SiMePh silane anti trans **4a**	-1653.54956	1035	-1653.21101
Ace-SPh-SiMePh silane syn trans **4a**	-1653.55002	1034	-1653.21193
Ace-SPh-SiMePh silane isodes	-1653.55383	1033	-1653.21756
Ace-SPh-SiMePh nitrilium FBN cis **18a**	-2076.51162	1262	-2076.09706
Ace-SPh-SiMePh nitrilium FBN trans **18a**	-2076.50867	1261	-2076.09805
Ace-SPh-SiMePh nitrilium FBN TS **18a**	-2076.49394	1259	-2076.08296
Ace-SPh- SiMe(tBu) cation cis *cis*-**13d**	-1578.94321	1097	-1578.57823
Ace-SPh- SiMe(tBu) cation trans *trans*-**13d**	-1578.94676	1096	-1578.58323
Ace-SPh- SiMe(tBu) cation TS **TS**(cis/trans)	-1578.91799	1091	-1578.55627
Ace-SPh- SiMe(tBu) cation isodes	-1578.89082	1092	-1578.53221
Ace-SPh- SiMe(tBu) silane anti trans **4b**	-1579.73833	1116	-1579.36799
Ace-SPh- SiMe(tBu) silane syn trans **4b**	-1579.73856	1117	-1579.36797
Ace-SPh- SiMe(tBu) silane isodes	-1579.74524	1115	-1579.37627
Ace-SPh-SiMe(tBu) nitrilium FBN trans **18**b	-2002.69516	1345	-2002.24919
Ace-SePh-SiMePh cation cis **13e**	-3656.14276	1011	-3655.81108
Ace-SePh-SiMePh cation trans **13e**	-3656.14078	1011	-3655.81172
Ace-SePh-SiMePh cation TS (cis/trans)	-3656.10479	1009	-3655.77569
Ace-SePh-SiMePh cation isodes	-3656.09273	1009	-3655.76621
Ace-SePh-SiMePh silane syn cis **6**	-3656.93769	1031	-3656.59975
Ace-SePh-SiMePh silane isodes	-3656.94023	1030	-3656.60664
Ace-TeMes-SiMePh cation cis **13f**	-1640.43108	1227	-1640.02345
Ace-TeMes-SiMePh cation trans **13f**	-1640.42899	1228	-1640.02396
Ace-TeMes-SiMePh cation TS (cis/trans)	-1640.38931	1225	-1639.98506
Ace-TeMes-SiMePh cation isodes	-1640.38199	1226	-1639.98074
Ace-TeMes-SiMePh silane syn cis **7**	-1641.22026	1249	-1640.80474
Ace-TeMes-SiMePh silane isodes	-1641.22896	1245	-1640.81978
Naph-SPh-SiMePh cation cis *cis*-**14a**	-1575.33717	923	-1575.03619
Naph-SPh-SiMePh cation trans *trans*-**14a**	-1575.33548	922	-1575.03658
Naph-SPh-SiMePh cation TS **TS**(cis/trans)	-1575.30856	921	-1575.00973
Naph-SPh-SiMePh cation isodes	-1575.27879	920	-1574.98213
Naph-SPh-SiMePh silane **9a**	-1576.12915	942	-1575.82367
Naph-SPh-SiMePh silane isodes	-1576.13507	942	-1575.83113
Naph-SePhSiMe(Ph) cation cis **14b**	-3578.72361	920	-3578.42464
Naph-SePhSiMe(Ph) cation trans **14b**	-3578.72140	920	-3578.42414
Naph-SePhSiMe(Ph) cation TS (cis/trans)	-3578.68612	917	-3578.39036
Naph-SePhSiMe(Ph) silane trans **10a**	-3579.51389	938	-3579.21043
Naph-SePhSiMe(Ph) cation isodes	-3578.66720	918	-3578.37233
Naph-SePhSiMe(Ph) silane isodes	-3579.52069	939	-3579.21792
Naph-SePhSiMe(tBu) cation cis **14c**	-3504.91025	998	-3504.58197
Naph-SePhSiMe(tBu) cation trans **14c**	-3504.91346	997	-3504.58691

Naph-SePhSiMe(tBu) cation TS (cis/trans)	-3504.87755	997	-3504.55059
Naph-SePhSiMe(tBu) silane trans **10b**	-3505.70214	1021	-3505.36523
Naph-SePhSiMe(tBu) cation isodes	-3504.84972	998	-3504.52240
Naph-SePhSiMe(tBu) silane isodes	-3505.71310	1022	-3505.37762

For the assignment of the IR bands of the *syn*- and *anti*-structures of silanes **x-x** (SPh, R = Me, *t*-Bu, Ph), their molecular structures were calculated at the M06-2X/Def2-TZVP level of theory at T = 298.15 K and p = 0.101 MPa (1 atm) in the gas phase with a subsequent frequency analysis. To account for anharmonicity effects, a scaling factor for the Si – H vibration was determined from the correlation of the computed data and the experimental results for a representative collection of silanes (Figure S86). The scaling factor is given by the scope of the line of best fit which is 0.9619 ± 0.0030.

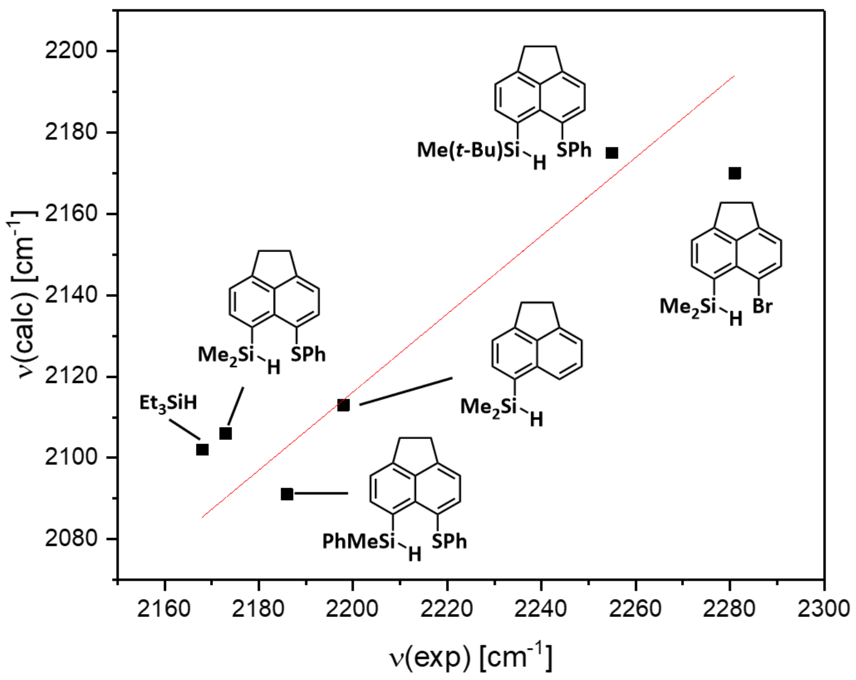

Figure S 86 – Correlation of the calculated wavenumber obtained at M06-2X/Def2-TZVP of the ν(Si – H) vibration and the experimental value for silanes **4a,b**, 5-bromo-6-dimethylsilyl acenaphthene, 5-dimethylsilyl acenaphthene, 5-phenylsulfanyl-6-dimethylsilylacenaphthene and triethylsilane.

References

[S1] N. Lühmann, *PhD thesis*, *University of Oldenburg* **2011**.

[S2] J. Beckmann, T. G. Do, S. Grabowsky, E. Hupf, E. Lork, S. Mebs, *Z. Anorg. Allg. Chem*. **2013**, 2233.

[S3] N. Lühmann, H. Hirao, S. Shaik, T. Müller, *Organometallics* **2011**, *30*, 4087-4096.

[S4] J. C. W. Chien, W. M. Tsai, M. D. Rausch, *J. Am. Chem. Soc.* **1991**, *113*, 8570-8571.

[S5] G. M. Sheldrick, *University of Göttingen*, Germany, **2014**. G. M. Sheldrick, *Acta Crystallogr. Sect. C* **2015**, *71*, 3.

[S7] M. J. T. Frisch, G. W.; Cheeseman, J. R.; Scalmani, G.; Caricato, M.; Hratchian, H. P.; Li, X.; Barone, V.; Bloino, J.; Zheng, G.; Vreven, T.; Montgomery, J. A.; Petersson, G., G. E. S. A.; Scuseria, H. B.; Nakatsuji, H.; Izmaylov, A. F.; Martin, R. L.;, J. L. P. Sonnenberg, J. E.; Heyd, J. J.; Brothers, E.; Ogliaro, F.; Bearpark, M.; Robb,, B. K. M. A.; Mennucci, K. N.; Staroverov, V. N.; Kobayashi, R.; Normand, J.;, A. G. Rendell, R.; Zakrzewski, V. G.; Hada, M.; Ehara, M.; Toyota, K.; Fukuda, and J. I. R.; Hasegawa, M.; Nakajima, T.; Honda, Y.; Kitao, O.; Nakai, H., *Gaussian 09*.

[S8] Y. Zhao, N. E. Schultz and D. G. Truhlar, *J. Chem. Theory Comput.* **2006**, *2*, 364.

[S9] (a) Krishnan, R.; Blinkley, J. S.; Seeger, R.; Pople, J.A. *J. Chem. Phys.* **1980**, *72*, 650; (b) McLean, A. D.; Chandler, G. S*. J. Chem. Phys.* **1980**, *72*, 5639; (c) Curtiss, L. A.; McGrath, M. P.; Blandeau, J-P.; Davis, N. E.; Binning, R. C.; Radom, Jr. L. *J. Chem. Phys.* **1995**, *103*, 6104; (d) Weigend, F.; Ahlrichs, R. *Phys. Chem. Chem. Phys.* **2005**, 3297
